# Genomic analyses of high‐grade neuroendocrine gynecological malignancies reveal a unique mutational landscape and therapeutic vulnerabilities

**DOI:** 10.1002/1878-0261.13057

**Published:** 2021-07-22

**Authors:** Haider Mahdi, Amy Joehlin‐Price, Esther Elishaev, Afshin Dowlati, Ata Abbas

**Affiliations:** ^1^ Gynecologic Oncology Division Cleveland Clinic OH USA; ^2^ Department of Pathology Cleveland Clinic OH USA; ^3^ Department of Pathology Magee‐Womens Hospital Pittsburgh PA USA; ^4^ Division of Hematology and Oncology Department of Medicine Case Western Reserve University Cleveland OH USA; ^5^ University Hospitals Seidman Cancer Center Cleveland OH USA; ^6^ Developmental Therapeutics Program Case Comprehensive Cancer Center Case Western Reserve University School of Medicine Cleveland OH USA; ^7^ Present address: Department of Obstetrics, Gynecology & Reproductive Sciences UPMC Magee‐Womens Hospital Pittsburgh PA USA

**Keywords:** CDK4/6 inhibitors, gynecologic neuroendocrine carcinoma, immunotherapy targets, PARP inhibitors, RB1, YAP1

## Abstract

High‐grade neuroendocrine carcinoma of gynecologic origin (NEC‐GYN) is a highly aggressive cancer that often affects young women. The clinical management of NEC‐GYN is typically extrapolated from its counterpart, small cell carcinoma of the lung (SCLC), but, unfortunately, available therapies have limited benefit. In our NEC‐GYN cohort, median progression‐free survival (PFS) and overall survival (OS) were 1 and 12 months, respectively, indicating the highly lethal nature of this cancer. Our comprehensive genomic analyses unveiled that NEC‐GYN harbors a higher mutational burden with distinct mutational landscapes from SCLC. We identified 14 cancer driver genes, including the most frequently altered *KMT2C* (100%), *KNL1* (100%), *NCOR2* (100%), and *CCDC6* (93%) genes. Transcriptomic analysis identified several novel gene fusions; astonishingly, the *MALAT1* lincRNA gene was found in ˜ 20% of all fusion events in NEC‐GYN. Furthermore, NEC‐GYN exhibited a highly immunosuppressive state, intact RB1 expression, and was uniquely enriched with the *YAP1* ^high^ molecular subtype. Our study identifies several potential therapeutic targets and suggests an urgent need to re‐evaluate the treatment options for NEC‐GYN.

## Graphical abstract

The neuroendocrine carcinoma of gynecologic origin (NEC‐GYN) is a deadly cancer that often affects young women. Treatment of NEC‐GYN is typically extrapolated from small cell lung cancer (SCLC), but, unfortunately, available treatments are inadequate. We report that NEC‐GYN is genomically distinct from SCLC, identify several potential therapeutic targets, and suggest an urgent need to re‐evaluate the treatment options for NEC‐GYN.

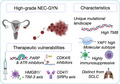

**Abbreviations**
BWA: Burrows–Wheeler alignment
CNV: copy number variation
COMPASS: complex proteins associated with Set1
DDR: DNA damage response
FDR: false discovery rate
FFPE: formalin‐fixed paraffin‐embedded
FIGO: International Federation of Gynecology and Obstetrics (Fédération Internationale de Gynécologie et d'Obstétrique, French)
HPV: human papillomavirus
IHC: immunohistochemistry
lincRNA: long intergenic noncoding RNA
MAF: mutation annotation format
NEC‐GYN: neuroendocrine carcinoma of gynecologic origin
OS: overall survival
PFS: progression‐free survival
RECIST: response evaluation criteria in solid tumors
SCLC: small cell lung carcinoma
SNP: single nucleotide polymorphism
TCGA: The Cancer Genome Atlas
TMB: tumor mutational burden
VCF: variant call format
VEP: variant effect predictor
WES: whole‐exome sequencing
XHMM: eXome hidden Markov model

## 1 Introduction

High‐grade NECs of the cervix are rare, aggressive cancers accounting for about 1–1.5% of all cervical cancer [1, 2]. Unlike the common type, squamous cell carcinoma, patients with cervical NEC are more likely to present with advanced or metastatic disease, resulting in poor prognosis. The 5‐year survival is up to 36% for early‐stage disease, but advanced‐stage disease has < 10% survival, with relapse rates exceeding 90% [1, 2, 3, 4]. These cancers are likely to have a vascular invasion and nodal or visceral metastasis. Unfortunately, NEC of the cervix affects young women with a median age of 37 [1, 2, 3, 4]. High‐grade NECs of other gynecologic origins are even rarer and share similar aggressive behavior and poor outcomes [3]. Furthermore, no prospective data are available to guide therapy in NEC‐GYN. Therefore, an urgency exists in understanding the underlying pathobiology of NEC‐GYN with hopes of developing better therapeutic options.

Current treatment considerations and guidelines for NEC‐GYN are mostly extrapolated from studies conducted in small cell lung cancer (SCLC). In patients with advanced‐stage metastatic disease, standard therapy includes chemotherapy with platinum and etoposide [3, 4, 5]. In patients with recurrent disease, data to guide treatment decisions are entirely absent but again inferred from SCLC, using agents such as topotecan and paclitaxel. However, these regimens are both toxic and have limited activity. Most recently, our group and others have described subgroups of SCLC that may have different pathobiology and potentially distinct therapeutic vulnerabilities [6, 7]. Therefore, we sought to comprehensively investigate NEC‐GYN genomics to understand their oncologic drivers and determine their similarities and differences with SCLC to improve their clinical management.

## 2 Materials and methods

### 2.1 Tumor samples, next‐generation sequencing, and public database

The study was approved by the institutional review board committee. The methods in this retrospective study were conformed to the standards set by the Declaration of Helsinki. Inclusion in this study required a pathologic diagnosis at the time of surgery as small cell or large cell neuroendocrine carcinoma or a diagnosis of combined neuroendocrine carcinoma (having both small cell and large cell histologies) or neuroendocrine features in conjunction with another more common histology. Additionally, slides and blocks with sufficient tissue for sequencing were required. Response Evaluation Criteria in Solid Tumors (RECIST) 1.1 criteria were used to assess response to treatment and disease progression. PFS and OS were calculated with KM curves. All the samples were re‐evaluated by an expert pathologist who marked the regions to dissect tissues for nucleic acid isolation. We successfully sequenced 15 samples from 12 patients, including three samples at two different time points. Whole‐exome sequencing (WES) of 14 samples and stranded paired‐end RNA‐seq of 13 samples, including 12 samples with matched WES, were performed at Novogene Corporation Inc. (Sacramento, CA) using 150‐bp paired‐end format on a NovaSeq 6000 (Illumina, San Diego, CA, USA) sequencer.

Data for frequently altered genes in SCLC were obtained from cBioPortal (https://www.cbioportal.org/ and https://www.cbioportal.org/sclc). Cervical and Ovarian cancer TCGA transcriptomic data were downloaded from the LinkedOmics portal (http://www.linkedomics.org/). Transcriptomic data from healthy human tissues were downloaded from GTEx (https://www.gtexportal.org/).

### 2.2 Whole‐exome sequencing (WES)

DNA was isolated from FFPE slides (area was marked by a pathologist) using an established FFPE DNA isolation pipeline at Novogene, Inc., and DNA QC was checked before starting the library preparation. One μg genomic DNA was used for the WES library preparation. Sequencing libraries were generated using Agilent SureSelect Human All Exon kit (Agilent Technologies, Santa Clara, CA, USA) following the manufacturer's recommendations, and index codes were added to each sample. Briefly, fragmentation was carried out by the hydrodynamic shearing system (Covaris, Woburn, MA, USA) to generate 180–280 bp fragments. Remaining overhangs were converted into blunt ends via exonuclease/polymerase activities, and enzymes were removed. After adenylation of 3' ends of DNA fragments, adapter oligonucleotides were ligated. DNA fragments with ligated adapter molecules on both ends were selectively enriched in a PCR. After PCR, the library was hybridized with the liquid phase with a biotin‐labeled probe, after which streptomycin‐coated magnetic beads are used to capture the exons of genes. Captured libraries were enriched in a PCR to add index tags to prepare for hybridization. Products were purified using the AMPure XP system (Beckman Coulter, Beverly, MA, USA) and quantified using the Agilent high sensitivity DNA assay on the Agilent Bioanalyzer 2100 system. Sequencing was performed using 150‐bp paired‐end format on a NovaSeq 6000 (Illumina) sequencer.

### 2.3 WES analyses, variant calling, and CNV analysis

Sequencing quality was checked using fastqc (https://www.bioinformatics.babraham.ac.uk/projects/fastqc/), and Trim Galore (http://www.bioinformatics.babraham.ac.uk/projects/trim_galore/) was used for adapter trimming. We first generated the reference genome (hs38DH.fa) using GRCh38+ALT+decoy+HLA and then created the BWA index for mapping. Sequence reads were mapped with GRCh38 using BWA‐MEM with default parameters to generate unsorted alignments (BAM) with ALT contigs aware mapping quality [8]. Following GATK best practice [9, 10], PCR duplicates were removed from sorted BAM files, and RG IDs were added using Picard tools (GitHub Repository http://broadinstitute.github.io/picard/). Subsequent realignment and base recalibration were performed using gatk4 (v 4.1.4.1). Databases of known polymorphic sites (dbsnp_146.hg38.vcf, Mills_and_1000G_gold_standard.indels.hg38.vcf, and Homo_sapiens_assembly38.known_indels.vcf) were used to exclude regions around known polymorphisms. Variants were then called using GATK4 HaplotypeCaller with default parameters.

Variant call format files were converted to MAF by mapping each variant to only one of all possible gene isoforms using the vcf2maf package (v 1.6.17) (https://github.com/ckandoth/vcf2maf). Ensembl Variant Effect Predictor (VEP) was used to determine the effect of variants (SNPs, insertions, deletions, CNVs, or structural variants) on genes, transcripts, and protein sequence, as well as regulatory regions [11]. VEP is CLIA‐compliant and uses HGVS variant format and Sequence Ontology nomenclature for variant effects. We used ExAC_nonTCGA.r0.3.1.sites.vep.vcf (germline variants called across thousands of normal samples excluding TCGA) for VEP (v 99.0) to filter variants. MAF files were further curated for missense mutations using SIFT [12] and PolyPhen‐2 [13], and only deleterious missense mutations were kept.

Copy number variations were identified using xhmm (eXome hidden Markov model, v 1.0) [14]. First, the depth of coverage was calculated using gatk (v 3.8) with default parameters. The mean coverage for each exon interval for each sample was extracted and merged into a single sample‐by‐target matrix. GC content of each coding exons was calculated using GATK to exclude all exons with more than 90% or < 10% GC content in the human reference sequence. Using Plink/Seq, a list of targets with low complexity was created based on the repeat‐masked sequence fraction. Using XHMM 'matrix' command, the read‐depth matrix was processed, and extreme GC content and low complexity lists were filtered out. PCA was ran to determine the strongest independent ways (principal components) in which the data vary to normalize mean‐centered data using this information. Next, we calculated the *z*‐score of the per‐sample read depth by centering relative to all target depths in that sample to remove any targets left with very high variance. Then, we used prenormalized read depths to remove the same targets and samples that were removed during the normalization process. This matrix was used for annotation purposes in the subsequent CNV discovery and genotyping steps. CNVs were called using the hidden Markov model (HMM) Viterbi algorithm, and each called CNV was quantitatively genotyped using HMM forward–backward algorithm. XHMM output file (.xcnv) only contains chromosomal coordinates for deletion (DEL) and duplication (DUP). To get genes that fall under the DEL and DUP regions, we first prepared a bed file from.xcnv file using biomart [15] (r package, v 2.44.1). Next, we used genomicranges [16] (r package, v 1.40.0) and Homo.sapiens (r package, v 1.3.1, https://doi.org/10.18129/B9.bioc.Homo.sapiens) to get genes that fall under CNVs after converting hg38 coordinates to hg19 using the ucsc liftover tool.

### 2.4 Mutational signatures, driver gene identification, and TMB comparison

To manage MAF files and visualize WES data, maftools [17] (r package, v 2.2.10) was used in the R environment. A copy number table of DEL and DUP was generated from CNV data obtained from XHMM analysis and used along with the clinical annotation data table for generating various figures in maftools. MAF summary, transition and transversion mutations, somatic interactions, Oncoplots, and lollipop plots for amino acid changes were plotted using maftools. Oncoplots function was used to plot SNV and CNV alterations together as 'multihit', if they happened to hit a given gene (a complete list of CNV data is included in Table S2).

Cancer driver genes were detected based on the positional clustering method using the 'oncodrive' function of maftools. The oncodrive function is based on OncodriveCLUST algorithm [18] that measures genes' bias toward large mutation clustering. It uses a background model composed of coding‐silent mutations that are not under selective pressure and, therefore, provide the baseline clustering of somatic mutations. In principle, most of the variants in cancer‐causing genes are enriched at a few specific hotspots; on the basis of these positions, OncodriveCLUST can identify cancer genes. A minimum of 5 mutations per gene cutoff was used, and the *P*‐value was calculated by *z*‐score. Oncogenic signaling pathways were detected using the 'OncogenicPathways' function of maftools.

Tumor mutational burden (TMB) was calculated and compared against TCGA cohorts using the 'tcgaCompare' function of maftools. Mutation loads from 33 TCGA cohorts were used and compared with NEC‐GYN TMB [19]. To plot mutational signature of DNA damage response (DDR) genes, we used a comprehensive DNA damage repair gene list maintained by Wood Laboratory at MD Anderson (https://www.mdanderson.org/documents/Labs/Wood‐Laboratory/human‐dna‐repair‐genes.html, last curated on June 10^th^, 2020), and the MAF file was subset for the DDR genes to use for Oncoplots. Potentially druggable gene categories and drug–gene interactions were detected using *drugInteractions* function of maftools. The *drugInteractions* uses drug–gene interactions and gene druggability information compiled from Drug Gene Interaction database [20] (http://www.dgidb.org).

### 2.5 RNA‐sequencing and analyses

RNA was extracted from FFPE sections using the miRNeasy FFPE kit (Qiagen, Germantown, MD, USA) according to the manufacturer's protocol. RNA quality was assessed via the Agilent 2100 Bioanalyzer (Agilent Technologies). Strand‐specific RNA‐seq library was prepared using NEBNext Ultra II Directional RNA Library Prep Kit (NEB, Ipswich, MA, USA) according to the manufacturer's protocols. Briefly, the RNA is fragmented randomly by adding a fragmentation buffer; then, the first‐strand cDNA is synthesized by using random hexamer primer, after which a custom second‐strand synthesis buffer (Illumina), dNTPs, RNase H, and DNA polymerase I are added to initiate the second‐strand synthesis. Terminal repair and sequencing adapter ligation were performed, followed by size selection and PCR enrichment. Double‐stranded cDNA libraries were purified using AMPure XP beads (Beckman Coulter) and quantified using the Agilent high sensitivity DNA assay on the Agilent Bioanalyzer 2100. Sequencing was performed using 150‐bp paired‐end format on a NovaSeq 6000 (Illumina) sequencer.

RNA‐sequencing quality was checked by running fastqc, and trimgalore was used for adapter and quality trimming. RNA‐seq reads were mapped against hg38 using star [21] (v 2.7.0e) aligner with default parameters. deseq2 [22] analysis with an adjusted *P*‐value < 0.001 was used to get a list of differentially expressed genes (DEGs). Top 5000 significant DEGs (sorted by *P*‐adj values) were used for unsupervised hierarchical clustering (NEC‐GYN vs SCLC). Pathway analysis (GO biological process) was performed on enrichr (https://amp.pharm.mssm.edu/Enrichr) database [23]. rsem [24] (v 1.3.2) analyses were performed to calculate FPKM and TPM values with default parameters.

### 2.6 Gene fusion and immune cell gene signature analyses

Novel and known somatic fusion genes were detected using fusioncatcher [25] (v 1.20) using default parameters. fusioncatcher is a powerful tool for finding somatic fusion genes in paired‐end RNA‐sequencing data. The fusion junctions were validated by using four different methods employing Bowtie, BLAT, STAR, and Bowtie2 aligners, and sequence analysis for ORFs (open reading frames) was also performed. Possible false positive and readthrough fusions were excluded.

The 'imsig' (r package, v 1.0.0) was used for immune cell gene signatures for profiling tumor microenvironment [26]. ImSig uses a set of immune gene signatures generated by a network‐based deconvolution approach for seven immune cell types. Using ImSig algorithm with default parameter (correlation threshold, *r* = 0.7, over 75% genes’ overlap was observed), a table of the relative abundance of immune cells across samples was generated. This table was used to plot the relative abundance of various immune cells.

### 2.7 Human papillomavirus (HPV) detection

Human papillomavirus was detected by utilizing hpvdetector [27] tool using default parameters. hpvdetector is a robust and precise tool for detecting HPV in tumor samples using WES and RNA‐seq platforms. hpvdetector utilized a custom‐made reference genome that contains human chromosomes and annotated genome of 143 pseudochromosomes of various HPV types. First, paired‐end WES FASTQ files were used to identify the presence of HPV types. Next, RNA‐seq FASTQ files were used to confirm the HPV gene expression in RNA‐seq data using default parameters of hpvdetector tool.

### 2.8 Immunohistochemistry (IHC)

Immunohistochemistry was performed as described previously [6]. RB1 (Cell Signaling, Danvers, MA, USA, cat # 9309), YAP1 (Cell Signaling, cat # 14074), and H3M4me1 (Abcam, Cambridge, MA, USA, cat # ab8895) antibodies were used. Staining was graded on a scale from 0 to 3 (no, weak, moderate, and strong). The final IHC score was calculated as the (staining intensity) x (percentage IHC positive tumor cells), yielding a final scoring range of 0–300. Scoring was performed by a gynecologic pathologist who was blinded to the outcome data.

### 2.9 Statistics

Two‐tailed Mann–Whitney *U*‐tests were used to calculate *P* values. For box plots, box and whisker graphs were plotted using either the Tukey method or minimum to maximum values. The middle line in the box indicates the median, whiskers indicate the highest and lowest values within 1.5 × IQR (interquartile distance between the 25th and 75th percentiles) up and down from the box, and dots plot values > 1.5 × IQR up and down from the box. In other plots, either whiskers were plotted down to the minimum and up to the maximum value and individual value as a point superimposed on the graph or mean with SD was plotted.

## 3 Results

### 3.1 Patient characteristics, treatments, and sequencing

Twenty‐seven patients were diagnosed with high‐grade NEC‐GYN at Cleveland Clinic between 1998 and 2018. Of them, we were able to retrieve sufficient samples in 16 specimens that represented 13 patients, including three samples at two different time points. Sequencing on one patient was unsuccessful. Thus, fifteen FFPE tissue samples from 12 patients including three samples at two different time points were included (see Table 1 for demographic and clinicopathologic annotations and Table S1 for individual patients’ characteristics). Clinical diagnoses were made at the time of surgery based on histomorphology and expression of at least one immunohistochemical marker of neuroendocrine differentiation. All patient’s tumors, at least at a one‐time point, were reported to express synaptophysin. Nine patients also at least focally expressed chromogranin. The majority of patients presented with advanced‐stage disease, FIGO stage IIIC‐IV (75%). Sites of origin included the cervix (58%), ovary (25%), and endometrium (17%). 58% of the patients received cisplatin/etoposide at the time of diagnosis, and second‐line treatments at recurrence/progression were topotecan (23%), liposomal doxorubicin +/− bevacizumab (23%), immunotherapy (nivolumab +/− ipilimumab) (15.4%), and others (Table 1, Table S1). The median number of prior lines of therapy was 1. Median PFS and OS were 1 and 12 months, respectively (Table 1). Whole‐exome sequencing (WES) of 14 samples and stranded paired‐end RNA‐sequencing of 13 samples, including 12 samples with matched WES, were performed. The mean coverage for WES was 280.7× (range, 240.8–330×), and the average number of stranded paired‐end reads for RNA‐seq was 35.7 million (range, 23.1–46.3 million).

**Table 1:** Patients’ clinical and pathologic characteristics and treatment.

Age, years (range)	Median	59 (35–73)
Race	W	10 (83%)
	AA	0
	Unknown	2 (17%)
Stage at diagnosis, *n* (%)	I	2 (17%)
	III	3 (25%)
	IVB	6 (50%)
	Recurrent	1 (8%)
Histology, *n* (%)	Small cell	5 (42%)
	Large cell	5 (42%)
	High grade	1 (8%)
	Combined/mixed	1 (8%)
Site of origin, *n* (%)	Cervix	7 (58%)
	Ovary	3 (25%)
	Endometrial	2 (17%)
Location of tissue, *n* (%)	Primary site	8 (57%)
	Metastatic/recurrent site	6 (43%)
Treatment regimen at diagnosis, *n* (%)	Cisplatin/etoposide	7 (58%)
	Carboplatin/paclitaxel	1 (8%)
	Carboplatin/paclitaxel/bevacizumab	1 (8%)
	Chemoradiation	1 (8%)
	No treatment	2 (17%)
Treatment at recurrence/progression (second line), *n* (%)	Topotecan	3 (23%)
	Immunotherapy (nivolumab +/− ipilimumab)	2 (15.4%)
	Liposomal doxorubicin +/− bevacizumab	3 (23%)
	Carboplatin/gemcitabine	1 (7.6%)
	Etoposide	1 (7.6%)
	Weekly paclitaxel	1 (7.6%)
	Sunitinib	1 (7.6%)
	Carboplatin/paclitaxel	1(7.6%)
Progression‐free survival (months)		1 (0–16)
Overall survival (months)		12 (6–101)
Site of recurrence/metastasis, *n* (%)	Peritoneal	7 (41.2%)
	Brain	3 (17.6%)
	Lung	4 (23.5%)
	Supraclavicular nodes	2 (11.8%)
	Spinal/bone	1 (5.9%)

### 3.2 Mutational profile and tumor mutation burden in NEC‐GYN

**Fig. 1:**
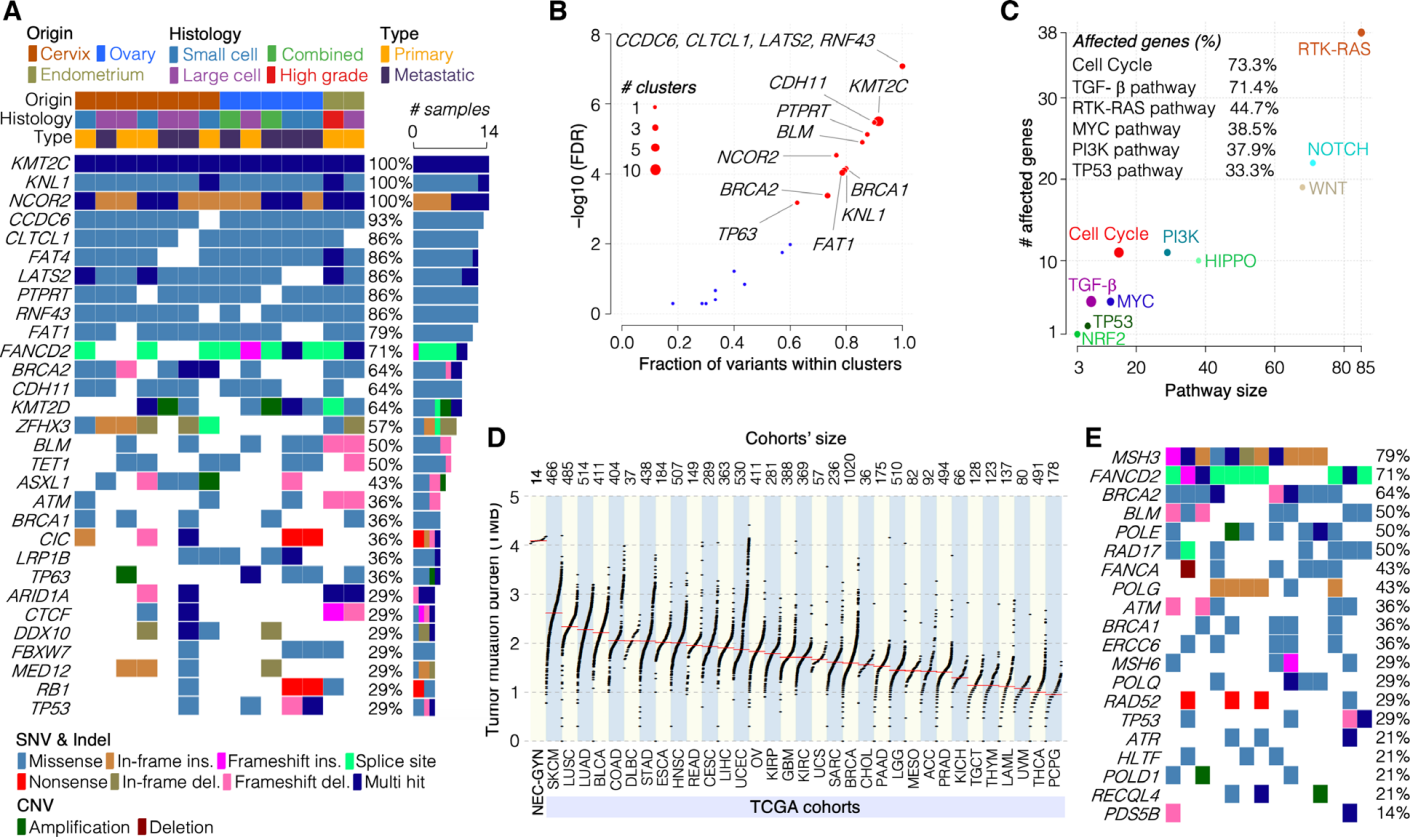
Unique mutational landscape in NEC‐GYN. (A) Frequently altered genes in NEC of gynecologic origins detected by GATK HaplotypeCaller and XHMM. Cohort’s clinical features are given on the top, and color codes representing various SNVs, Indel, and CNVs are given at the bottom. A ‘Combined’ histology represents the presence of both small cell and large cell NEC in a tumor. (B) A scatter plot showing cancer driver genes (FDR < 0.01) detected by OncodriveCLUST algorithm using positional clustering method. (C) Oncogenic signaling pathways associated with mutated genes in NEC‐GYN are illustrated in a scatter plot. Percentage of genes altered in various oncogenic pathways is given. (D) Comparison of TMB in NEC‐GYN with TCGA representing 33 tumors’ cohorts. The cohorts’ size is given on the top. (E) Mutation profile of DNA damage repair genes in NEC‐GYN cohort detected by GATK HaplotypeCaller and XHMM. Please refer to Fig. 1A for color codes representing mutation types.

We discovered a unique mutational landscape in our NEC‐GYN cohort (Fig. 1, Figs S1–S3, Table S2). The WES analyses detected mutations in *KMT2C*, *KNL1*, and *NCOR2* genes in all the tumor samples (100%) (Fig. 1A, Figs S4–S6). Cervical NEC samples had a significantly higher number of mutations within individual genes compared with other NEC‐GYN tumors (Fig. S7). The altered genes that were common between tumors from three gynecologic sites, surprisingly, enriched in the Hippo signaling pathway (Fig. S8). Using the oncodriveCLUST algorithm, we identified 14 cancer driver genes at a false discovery rate (FDR) of 0.01 (Fig. 1B, Table S3). The oncodriveCLUST algorithm measures genes' bias toward large mutation clustering and identifies specific hotspots where most of the variants in cancer‐causing genes are enriched. The frequent driver genes were *CCDC6* (13 mutations in one cluster, 93% mutation frequency), *LATS2* (15 mutations in 2 clusters, 86%), and *CLTCL1* and *RNF43* (18 and 17 mutations, respectively, in 3 clusters, 86%) (Table S3). Oncogenic pathway analysis revealed that the highest proportion of mutated genes were found in the cell cycle (73.3%), TGF‐β (71.4%), RTK‐RAS (44.7%), MYC (38.5), and PI3K (37.9%) pathways (Fig. 1C, Figs S9 and S10). Next, we compared the tumor mutation burden (TMB) of our cohort with 33 TCGA cohorts of various tumors. Surprisingly, NEC‐GYN demonstrated the highest TMB compared to all TCGA cohorts examined (Fig. 1D). Furthermore, we observed frequent mutations in various DNA damage response (DDR) pathway genes including *MSH3* (79%), *FANCD2* (71%), *BRCA2* (64%), and *BLM* (50%) (Fig. 1E), corroborating high TMB in NEC‐GYN. Moreover, drug–gene interaction analysis using GDIdb identified clinically actionable (*KMT2C*, *MAP3K1*) and other potentially druggable targets (Fig. S11). Since our cohort includes specimens from three patients at two different time points (all metastatic, see Table S1), we compared these tumors individually. We found that an ovarian small cell NEC exhibited mutations in additional genes and multiple hits in previously mutated genes at recurrence (Fig. S12). As KMT2C was the top mutated gene in our cohort and responsible for maintaining the H3K4me1 mark at enhancer regions, we performed IHC for H3K4me1 but did not observe any subtle change (Fig. S13). The available literature, indicating KMT2C loss modulates H3K4me1 levels at enhancer regions in a context‐dependent manner [8, 9, 10], suggests that future mechanistic studies are needed to investigate its impact on genome‐wide transcription.

### 3.3 Recurrent gene fusions, unique transcriptomic signatures, and immunosuppressive tumor microenvironment in NEC‐GYN

**Fig. 2:**
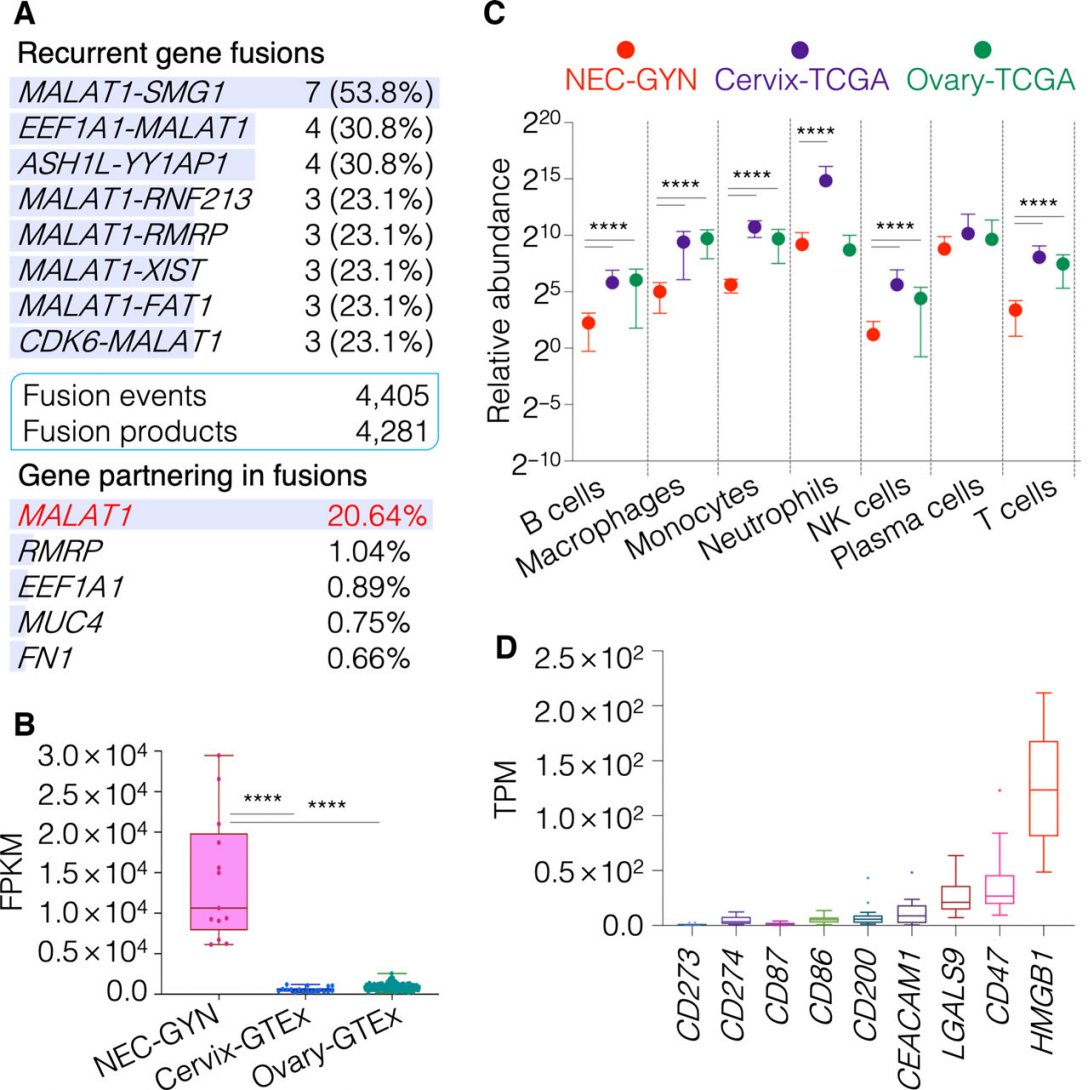
Transcriptional signature and tumor immune microenvironment in NEC‐GYN. (A) Recurrent gene fusions detected by using FusionCatcher in NEC‐GYN. A total of 4405 fusion events were observed across NEC‐GYN cohort that resulted in 4281 unique fusion products (see Table S4 for full list). Genes partnering in total fusion events (%) are given. (B) Expression profile of *MALAT1* gene in NEC‐GYN (*n* = 13), normal cervix (*n* = 18), and normal ovary (*n* = 171) tissues (mean with SD, ****P ≤ 0.0001 by two‐tailed Mann–Whitney *U*‐test). (C) Comparison of relative abundance of immune cells in NEC‐GYN (*n* = 13), Cervix‐TCGA (*n* = 304), and Ovary‐TCGA (*n* = 303) based on network‐based deconvolution (ImSig) analysis (mean with SD, ****P ≤ 0.0001 by two‐tailed Mann–Whitney *U*‐test). (D) Relative expression levels of various immune checkpoint genes in NEC‐GYN (*n* = 13, box plot by Tukey method).

Using transcriptomic data, we discovered several recurrent gene fusions, including *MALAT1‐SMG* (53.8%), *EEF1A1‐MALAT1* (30.8%), and *ASH1L‐YY1AP1* (30.8%) (Fig. 2A, Table S4). Remarkably, the *MALAT1* gene partnered in > 20% of all the fusion events (Fig. 2A) and demonstrated extremely high expression in NEC‐GYN compared to healthy cervix and ovary (Fig. 2B, Fig. S14). Comparing transcriptomic profiles of NEC‐GYN with TCGA cervical squamous cell and epithelial ovarian cancer, the non‐neuroendocrine counterparts of the NEC‐GYN, chromatin assembly and nucleosome organization were the top GO functions for significantly overexpressed genes in NEC‐GYN (Figs S15 and S16). Remarkably, underexpressed genes in NEC‐GYN were enriched for protein modification and catabolic processes and neutrophil‐mediated immunity (Figs S15 and S16).

The tumor immune microenvironment is crucial in determining immunotherapy outcomes. Using transcriptomic data, we analyzed immune cell gene signatures for profiling tumor microenvironment. Our data revealed significantly lower immune cell infiltrations in NEC‐GYN compared to the ovarian or cervical nonendocrine cohorts, except plasma cells (Fig. 2C). Remarkably, expression of HLA‐A, HLA‐B, and HLA‐C was significantly lower in NEC‐GYN compared to ovarian and cervical cancer (*P*‐value < 0.0001), further corroborating immunosuppressive nature of NEC‐GYN (Fig. S17). We then looked at the expression of various immune checkpoint genes and observed a high expression of *HMGB1*, followed by *CD47* and *LGALS9*, and low expression of *CD274* (PD‐L1) and *CD273* (PD‐L2) (Fig. 2D).

### 3.4 NEC‐GYN exhibits a distinct mutational and transcriptomic profile from SCLC

**Fig. 3:**
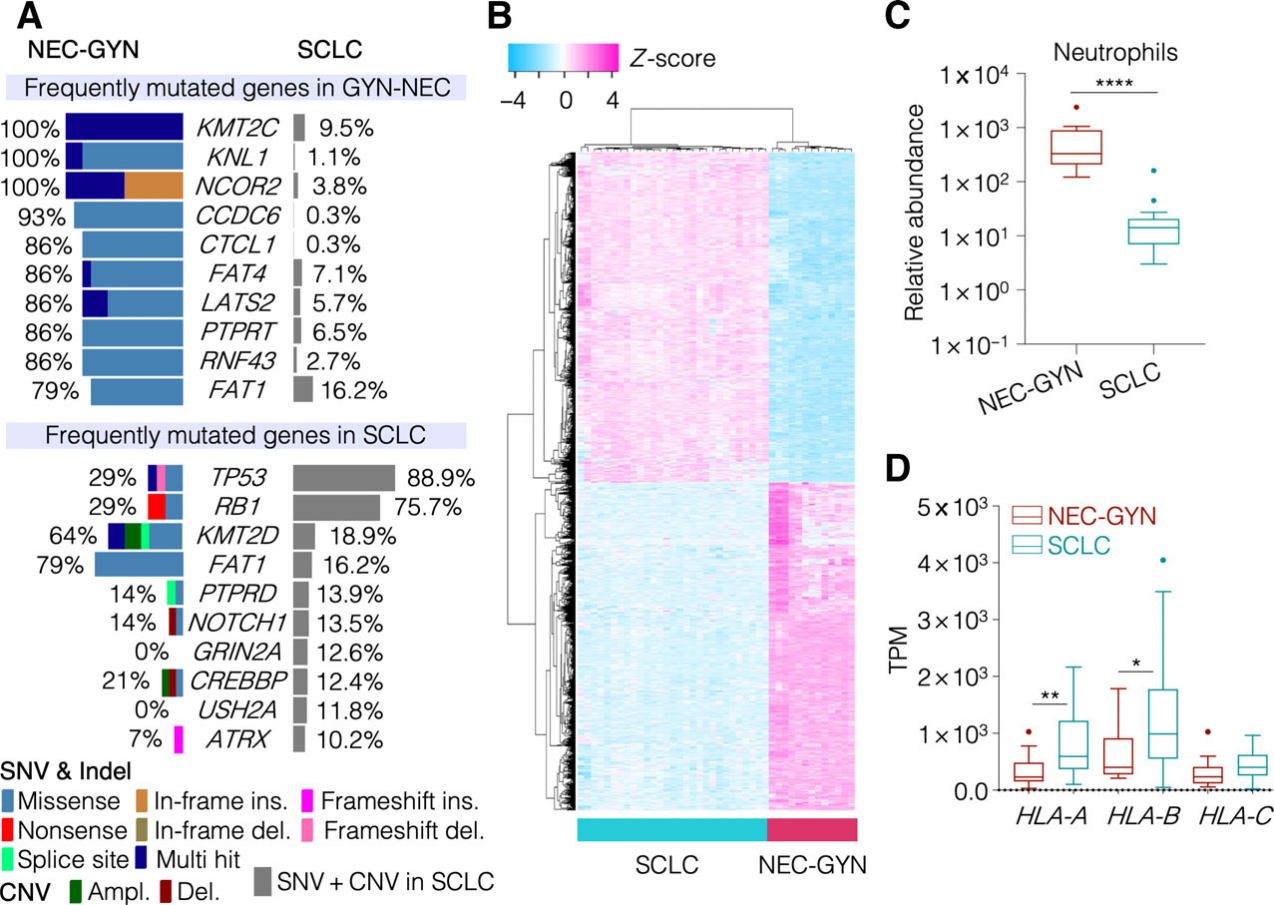
Comprehensive genomic and transcriptomic analyses between NEC‐GYN and SCLC. (A) Comparison of frequently mutated genes (top 10 genes from both NEC‐GYN and SCLC) between NEC‐GYN and SCLC cohorts. (B) A heatmap representing distinct transcriptional signatures between NEC‐GYN and SCLC cohorts. DEGs were sorted by *P*‐adj values, and top 5000 genes were used for unsupervised hierarchical clustering. (C) Comparison of relative abundance of neutrophils (ImSig analysis) between NYC‐GYN (*n* = 13) and SCLC (*n* = 29) cohorts (Tukey, ****P ≤ 0.0001 by two‐tailed Mann–Whitney *U*‐test). (D) Expression patterns of HLA class‐I genes in NEC‐GYN (*n* = 13) and SCLC (*n* = 29) cohorts (Tukey, *P = 0.0262, **P = 0.0028 by two‐tailed Mann–Whitney *U*‐test).

Since NEC‐GYN tumors are traditionally considered similar to SCLC and all treatment recommendations are extrapolated from SCLC, the prototypical high‐grade neuroendocrine cancer, we compared our results with SCLC genomic mutation data obtained from cBioPortal, including data from a cohort of 90 SCLC patients from our laboratory. Astonishingly, the NEC‐GYN mutation profile did not match SCLC as the most frequently mutated genes in NEC‐GYN were among the least mutated in SCLC (Fig. 3A). In SCLC, *TP53* and *RB1* genes are frequently mutated (88.9% and 75.7% respectively); however, *TP53* and *RB1* mutations in our NEC‐GYN cohort were significantly lower (29%) (Fig. 3A). We also looked at only small cell NEC of GYN (*n* = 5) and found that only one patient (with two specimens at different time points) had mutations in *TP53* and *RB1* genes. Compared to SCLC, NEC‐GYN showed highly distinct transcriptomic patterns, as represented by the top 5000 DEGs (*P*‐adj > 0.01) (Fig. 3B, Figs S18 and S19). Compared to the immune microenvironment of SCLC, only neutrophil infiltration was significantly higher in NEC‐GYN (*P* ≤ 0.0001) (Fig. 3C, Fig. S20); however, *HLA‐A* and *HLA‐B* expression were significantly lower in our cohort (Fig. 3D) demonstrating lower antigen presentation capacity.

### 3.5 NEC‐GYN is enriched with *YAP1* molecular subtype and expresses intact RB1 protein

**Fig. 4:**
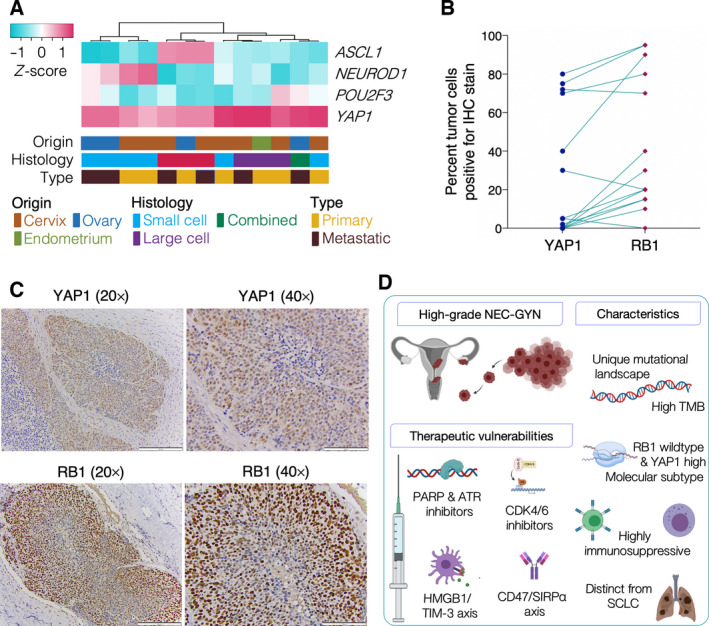
NEC‐GYN molecular subtypes and expression of YAP1 and RB1 proteins. (A) Molecular subtypes of NEC‐GYN defined by the expression of four key transcriptional regulators, *ASCL1, NEUROD1, FOU2F3,* and *YAP1*. (B) A graph correlating YAP1 and RB1 proteins expression by IHC in NEC‐GYN (*n* = 14), and (C) the representative IHC images showing YAP1 and RB1 in a neuroendocrine large cell carcinoma of cervix (scale bars in 20× and 40× images represent 200 and 100 μm, respectively). (D) A cartoon depicting origin of NEC‐GYN, unique characteristics, and therapeutic opportunities.

Since SCLC is typically classified into four molecular subtypes, including *ASCL1* (the largest group), *NEUROD1*, *POU2F3*, and a small group represented by *YAP1* [7], we used similar approach to investigate the molecular subtypes in our cohort. Surprisingly, most of the NEC‐GYN tumors, while still clustering into four groups, represented the *YAP1* high, a chemoresistant molecular subtype (Fig. 4A). This finding led us to evaluate the YAP1 protein expression in NEC‐GYN tumors using immunohistochemistry (IHC). Since *RB1* gene mutation frequency was lower in NEC‐GYN compared to SCLC, we also performed RB1 IHC and compared them with YAP1 expression level (Fig. 4B,C, Fig. S21A). Remarkably, high expression of YAP1 correlated with high RB1 level; however, tumors with a moderate RB1 expression exhibited very weak or no YAP1 protein expression (Fig. 4B).

Next, we investigated the HPV status in the NEC‐GYN cohort using WES data and reconfirmed them using RNA‐seq data. We found that four out of seven patients have positive HPV expression (57%) in NEC of cervical origin, including three patients were positive for HPV18 and one was positive for HPV16. Surprisingly, all the three HPV18 positive tumors exhibited no YAP1 protein expression (Fig. S21B). We also compared the HPV status of NEC‐Cervix with RB1 protein expression level. All the HPV‐positive tumors were expressing lower RB1 protein compared to HPV‐negative NEC‐Cervix (Fig. S21C).

## 4 Discussion

Comprehensive genomic studies are limited in NEC‐GYN. A pilot study with WES data of 5 neuroendocrine cervix cases reported recurrent mutation in *ATRX*, *EBRR4*, and AKT/mTOR signaling pathway genes [28]. Another study of NEC of the cervix reported mutation in *PIK3CA* (18%), *KRAS* (14%), and *TP53* (11%) genes using a limited gene‐targeted panel [29]. A study of 10 cervical NEC cases found frequent mutations in *TP53* (40%) and *PIK3CA* (30%) genes, again using a targeted gene panel [30]. More recently, Hillman *et al*. [31] reported recurrent mutations in *PIK3CA* (26.7%) and *KMT2C* (20%) genes along with deletion in regions containing the *PTEN* gene (33%). Remarkably, no transcriptomic data are available for NEC‐GYN. Our study is the first comprehensive study with matched genomic and transcriptomic data to the best of our knowledge. Given the rarity of this highly malignant tumor, our cohort with even a small sample size provides a novel opportunity to understand the pathobiology of NEC‐GYN. However, collaborative efforts are needed to perform multicenter comprehensive genomic and clinical studies to manage this deadly cancer effectively.

Our data demonstrated a unique mutational landscape in NEC‐GYN with frequent mutations in COMPASS family members (*KMT2C*, 100%; and *KMT2D*, 64%) involved in epigenetic regulation of enhancers and their loss promotes tumorigenesis [32]. Surprisingly, we observed frequent mutations in various DDR genes (Fig. 1E) that possibly explain the incidence of very high TMB in our cohort compared to 33 TCGA cohorts. Providentially, DDR gene mutations confer therapeutic vulnerabilities in cancers [33, 34, 35]. A case report demonstrated promising results using PARP inhibitor in a small cell carcinoma of the cervix patient with somatic *BRCA2* gene mutation [36]. PARP inhibitor, rucaparib, treatment that resulted in 15‐month PFS and symptomatic improvement was noteworthy for such a disease with very high mortality. A recent study evaluating PARP1 expression using IHC in pathological specimens of high‐grade NEC of cervix reported PARP1 positivity for 91% of samples [37]. In our NEC‐GYN cohort, the mutation rate in *BRCA2* (64%) and other genes involved in the DDR pathway was remarkably high (Fig. 1E). Our data strongly suggest using PARP and other inhibitors targeting the DDR pathway in NEC‐GYN. Furthermore, mutations in the cancer driver gene, *CCDC6* (93%), suggest potential benefit from PARP inhibitor treatment [38].

Transcriptional dysregulation is a hallmark of cancer, and oncogenic gene fusions are observed across several cancer types [39]. NEC‐GYN transcriptomic signatures revealed modulation in chromatin assembly and nucleosome organization (Figs S15 and S16). These transcriptomic data are consistent with the NEC‐GYN mutational profile as *KMT2C*, *KMT2D*, and other epigenetic genes were frequently mutated. Surprisingly, gene fusions in NEC‐GYN were mostly partnered with the *MALAT1* gene (Fig. 2A). MALAT1 is one of the best‐characterized lincRNA and a potentially viable druggable target [40]. Transcriptomic‐based tumor immune microenvironment analysis allows us to understand the abundance of the immune cells in tumor tissues. The presence of significantly lower immune cell infiltrations in our cohort than their non‐neuroendocrine counterparts (Fig. 2C) explains its immunosuppressive nature and perhaps the minimal effect of anti‐PD1/PD‐L1 immunotherapies in NEC‐GYN [41]. It is important to note that high TMB is often associated with the inflamed phenotype (high immune cell infiltration) and increased neoantigen presentation. In contrast, NEC‐GYN exhibits low immune cell infiltration partially due to significantly reduced expression of HLA class‐I genes (Fig. S17) that may have compromised neoantigen presentation. Remarkably, other novel immunotherapeutic targets such as *HMGB1* and *CD47* expression are far greater compared to the *CD274* gene (codes for PD‐L1) (Fig. 2D). HMGB1, Gal‐9, CEACAM1, and PS are the four well‐known ligands for TIM‐3, a negative regulator of Type 1 immunity expressed on various immune cells. CD47 is a ligand for SIRPα receptor present on macrophages and involved in ‘don’t eat me’ signaling. Future clinical studies are needed to evaluate HMGB1/TIM‐3 and CD47/SIRPα axes for potential immunotherapy targets, alone or in combination with chemotherapy, in NEC‐GYN.

Treatment options for NEC‐GYN are mostly inferred from studies conducted in SCLC. Astoundingly, the mutational landscape and transcriptional signatures are quite different in NEC‐GYN compared to SCLC (Fig. 3). SCLC is characterized by a very high *TP53* and *RB1* mutation rate that is believed to be responsible for the small cell phenotype. Conversely, *TP53* and *RB1* mutations in NEC‐GYN were significantly lower (29%) then SCLC and did not distinctly correlate with their histology (Figs 3A and 1A). Unexpectedly, NEC‐GYN showed a high expression of *YAP1*, which represents a small molecular subtype in SCLC. YAP1 is a component of the Hippo pathway responsible for multidrug resistance [42]. This unique YAP1 high molecular subtype possibly maintains NYC‐GYN's chemorefractory nature that further facilitates immunosuppressive tumor microenvironment [43]. Lower antigen presentation capacity (Fig. 3D) also aids in immunosuppressive tumor microenvironment in NEC‐GYN, and higher infiltration of neutrophils (Fig. 3C) can be associated with poor clinical outcome [44].

A meta‐analysis by Castel *et al*. and earlier reports have suggested the association of neuroendocrine carcinoma of cervix with HPV [45, 46, 47]. In our cohort, only 57% of NEC of cervix patients were HPV‐positive. A recent study showed that hyperactivation of YAP1 without HPV infection can cause cervical carcinogenesis [48]. Our data showing a negative correlation of HPV positivity and YAP1 protein expression suggest that HPV can be dispensable for NEC of cervix. In consistence with the published literature, HPV does lower the RB1 protein expression in NEC of cervix. However, the overall high expression of RB1 protein with limited genomic mutation in this gene can be exploited for clinical intervention.

## 5 Conclusion

Our data demonstrate a unique mutational landscape in NEC‐GYN with a remarkably high TMB and suggest novel multimodality therapeutic measures to address this highly fatal cancer (Fig. 4D). Frequent mutations of DDR genes encourage trials of PARP and ATR inhibitor‐based therapies and further warrant investigation of potential germline mutations as young women are mostly affected by this malignancy. Our finding of frequent *RB1* wild‐type status correlating high RB1 protein expression suggests a potential therapeutic vulnerability to CDK 4/6 inhibitors [6]. Overall, our data provide a framework for future studies of NEC‐GYN in prospective clinical settings to improve outcomes via the use of targeted therapies.

## Conflicts of interest

The authors declare no conflicts of interest.

## Author contributions

HM, AD, and AA conceptualized the study. AJP and EE performed pathological evaluation. AA performed bioinformatic data analyses and interpretations. HM and AD provided clinical interpretation. HM and AA drafted the manuscript. HM and AA supervised the study. All authors critically revised the manuscript and gave final approval.

### Peer Review

The peer review history for this article is available at https://publons.com/publon/10.1002/1878‐0261.13057.

## Supporting information


**Fig. S1**. Mutational profile of neuroendocrine carcinoma of gynecologic origin (NEC‐GYN) was detected by GATK Haplotype Caller.
**Fig. S2**. A) Percentages of various CNV classes in NEC‐GYN (left) and transition & transversion mutations (right); and B) percentages of various CNV classes in various tumor sample in our cohort.
**Fig. S3**. Mutually exclusive or co‐occurring set of genes.
**Fig. S4**. Lollipop plot showing the mutations (amino acid changes) in A) *KMT2C*; and B) *KNL1* gene in NEC‐GYN.
**Fig. S5**. Lollipop plot showing the mutations (amino acid changes) in A) *NCOR2*; B) *BRCA1*; and C) *BRCA2* gene in NEC‐GYN.
**Fig. S6**. Lollipop plot showing the mutations (amino acid changes) in A) *TP53*; and B) *RB1* gene. *TP53* and *RB1* genes are highly mutated in SCLC but moderately mutated in our NEC‐GYN cohort. Lollipop plot showing the mutations (amino acid changes) in C) *CDH11*; and D) *KMT2D* gene.
**Fig. S7**. Cervical NEC samples showing higher number of mutations within individual genes compared to Ovary and Endometrial NEC.
**Fig. S8**. Venn diagram representing common mutated genes between Cervical, Ovarian, and Endometrial NEC (top 20 frequently mutated genes from each of three groups were used).
**Fig. S9**. Oncogenic pathways affected in NEC‐GYN. A) RTK‐RAS pathway; and B) Cell‐Cycle pathway.
**Fig. S10**. Oncogenic pathways affected in NEC‐GYN.
**Fig. S11**. Various potentially druggable targets were identified by drug–gene interactions analysis using GDIdb.
**Fig. S12**. Mutational landscapes of the specimen obtained from the same patients.
**Fig. S13**. A) Representative H3K4me1 IHC images (20x and 40x) of an ovarian high‐grade NEC. Twelve out of fourteen specimen exhibit strong staining. B) Representative H3K4me1 IHC images (20x and 40x) of an endometrial high‐grade NEC. Tow out of fourteen specimens show strong staining in 70‐80% tumor cells and week to moderate staining in 20‐30% cells.
**Fig. S14**. A) MALAT1 expression levels across various normal human tissues from GETx. TPM value of MALAT1 in NEC‐GYN (shown in the box) is much higher than all of the GETx normal tissues. B) MALAT1 expression level in NEC‐GYN (n=13) compared to SCLC (n=29) and TCGA cohorts of cervical (n=304) and ovarian (n=303) cancers (Tukey, **P = 0.0012, ****P > 0.0001 by two‐tailed Mann–Whitney U‐test).
**Fig. S15**. GO analysis of differentially expressed genes in neuroendocrine carcinoma of cervix (n=8) from NEC‐GYN cohort compared to cervical cancer from TCGA (n=304).
**Fig. S16**. GO analysis of differentially expressed genes in neuroendocrine carcinoma of ovary (n=4) from NEC‐GYN cohort compared to ovarian cancer from TCGA (n=303).
**Fig. S17**. Comparison of HLA class‐I (HLA‐A, HLA‐B, and HLA‐C) genes expression in NEC‐GYN (n=13), cervical (TCGA, n=304), and ovarian (TCGA, n=303) cancers (Tukey, ****P > 0.0001 by two‐tailed Mann–Whitney U‐test).
**Fig. S18**. Comparison of NEC‐GYN (n=13) with SCLC (n=29).
**Fig. S19**. GO analysis of differentially expressed genes in NEC‐GYN (n=13) compared to SCLC (n=29, p‐adj <0.001, fold change >2).
**Fig. S20**. Comparison of relative abundance of immune cells in NEC‐GYN (n=13) and SCLC (n=29) tumors based on network‐based deconvolution (ImSig) analysis (mean with SD, ****P ≤ 0.0001 by two‐tailed Mann–Whitney U‐test).
**Fig. S21**. A) Representative YAP1 and RB1 IHC images (20x and 40x) of a cervix small cell neuroendocrine carcinoma. B) Correlation of *YAP1* RNA level (TPM, transcript per million) with protein expression (IHC) and HPV status. C) Correlation of RB1 expression (IHC) with HPV.

**Table S1**. Demographic and clinicopathologic characteristics of the patients.

**Table S2**. Copy number variations (CNV) detected by XHMM.

**Table S3**. List of cancer driver genes detected based on positional clustering.

**Table S4**. List of fusion genes.

## Data Availability

The datasets generated during the current study are available in the SRA and GEO repositories with the following accession numbers. SRA accession number for WES: PRJNA661624. GEO accession number for RNA‐seq: GSE157601.
