# Supplementary material for: Genomic analyses of high‐grade neuroendocrine gynecological malignancies reveal a unique mutational landscape and therapeutic vulnerabilities
Source: Mol Oncol. 2021 Jul 22;15(12):3545–58. doi: 10.1002/1878-0261.13057 (PMC8637558; doi:10.1002/1878-0261.13057)
Supplement: Supplementary file 1 — Fig. S1. Mutational profile of neuroendocrine carcinoma of gynecologic origin (NEC‐GYN) was detected by GATK Haplotype Caller. Fig. S2. A) Percentages of various CNV classes in NEC‐GYN (left) and transition & transversion mutations (right); and B) percentages of various CNV classes in various tumor sample in our cohort. Fig. S3. Mutually exclusive or co‐occurring set of genes. Fig. S4. Lollipop plot showing the mutations (amino acid changes) in A) KMT2C; and B) KNL1 gene in NEC‐GYN. Fig. S5. Lollipop plot showing the mutations (amino acid changes) in A) NCOR2; B) BRCA1; and C) BRCA2 gene in NEC‐GYN. Fig. S6. Lollipop plot showing the mutations (amino acid changes) in A) TP53; and B) RB1 gene. TP53 and RB1 genes are highly mutated in SCLC but moderately mutated in our NEC‐GYN cohort. Lollipop plot showing the mutations (amino acid changes) in C) CDH11; and D) KMT2D gene. Fig. S7. Cervical NEC samples showing higher number of mutations within individual genes compared to Ovary and Endometrial NEC. Fig. S8. Venn diagram representing common mutated genes between Cervical, Ovarian, and Endometrial NEC (top 20 frequently mutated genes from each of three groups were used). Fig. S9. Oncogenic pathways affected in NEC‐GYN. A) RTK‐RAS pathway; and B) Cell‐Cycle pathway. Fig. S10. Oncogenic pathways affected in NEC‐GYN. Fig. S11. Various potentially druggable targets were identified by drug–gene interactions analysis using GDIdb. Fig. S12. Mutational landscapes of the specimen obtained from the same patients. Fig. S13. A) Representative H3K4me1 IHC images (20x and 40x) of an ovarian high‐grade NEC. Twelve out of fourteen specimen exhibit strong staining. B) Representative H3K4me1 IHC images (20x and 40x) of an endometrial high‐grade NEC. Tow out of fourteen specimens show strong staining in 70‐80% tumor cells and week to moderate staining in 20‐30% cells. Fig. S14. A) MALAT1 expression levels across various normal human tissues from GETx. TPM value of MALAT1 in NEC‐GYN (shown [file MOL2-15-3545-s004.pdf]

Supplementary Figures

Genomic analyses of high-grade neuroendocrine gynecological malignancies reveal a unique mutational landscape and therapeutic vulnerabilities

Haider Mahdi[1,$,#], Amy Joehlin-Price[2], Esther Elishaev[3], Afshin Dowlati[4,5,6], Ata Abbas[4,6,#]

[1]Gynecologic Oncology Division, Cleveland Clinic, Cleveland, OH 44195, USA

[2]Department of Pathology, Cleveland Clinic, Cleveland, OH 44195, USA

[3]Department of Pathology, Magee-Womens Hospital, Pittsburgh, PA 15213, USA

[4]Division of Hematology and Oncology, Department of Medicine, Case Western Reserve University, Cleveland, OH 44106, USA

[5]University Hospitals Seidman Cancer Center, Cleveland, OH 44106, USA

[6]Developmental Therapeutics Program, Case Comprehensive Cancer Center, Case Western Reserve University School of Medicine, Cleveland, OH 44116, USA

[$]Current address: Department of Obstetrics, Gynecology & Reproductive Sciences, UPMC Magee-Womens Hospital, Pittsburgh, PA 15213, USA

[#]Corresponding authors:

Haider Mahdi, MD, Magee Womens Hospital and Magee Womens Research Institute, University of Pittsburgh Medical Center, Pittsburgh, PA 15213. Phone: 216-339-1191; Email: mahdihs@upmc.edu

Ata Abbas, MS, PhD, Case Western Reserve University, 2103 Cornell Road, WRB 3-301, Cleveland, OH 44106. Email: ata.abbas@case.edu

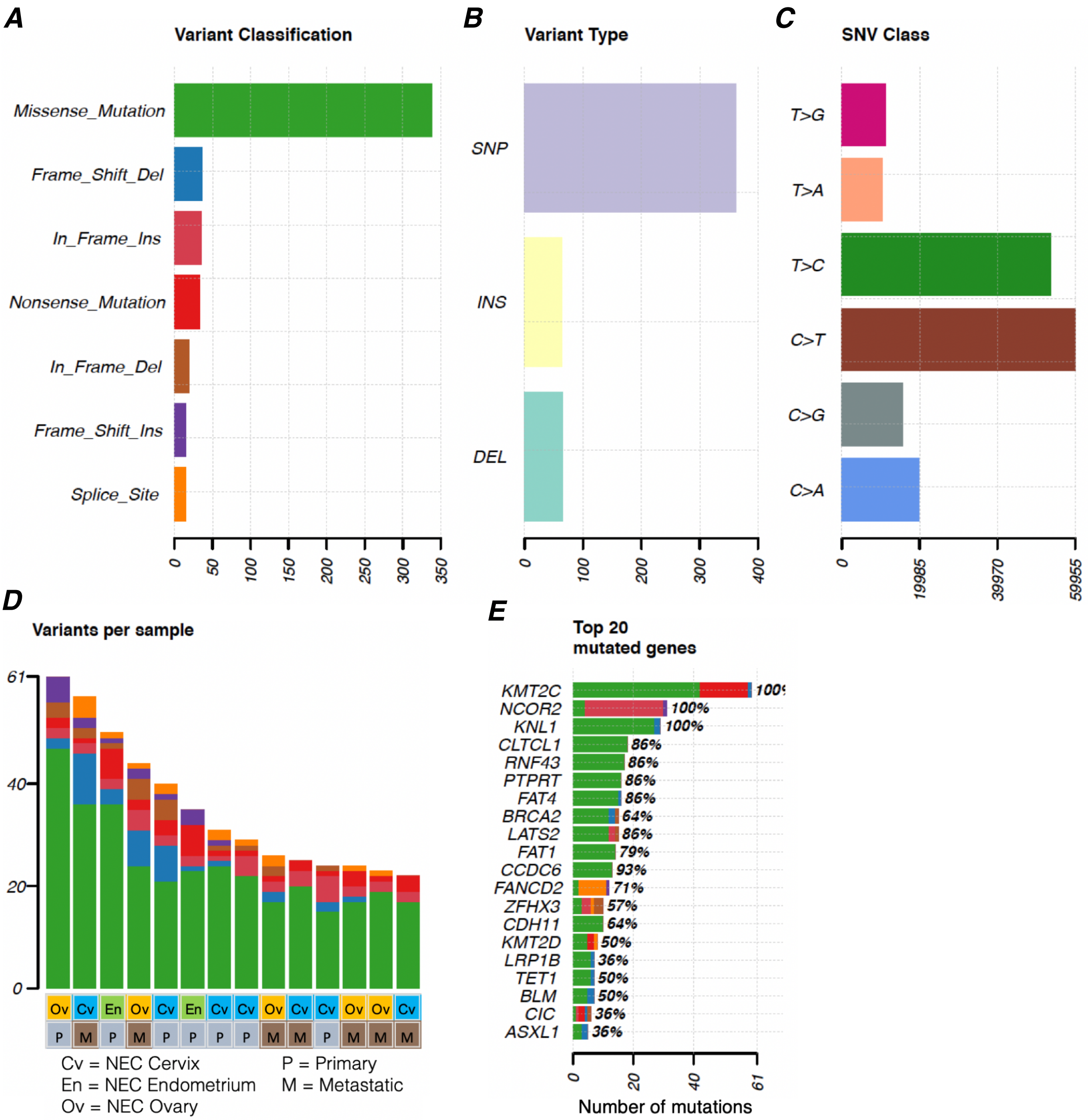

Supplementary Fig. 1. Mutational profile of neuroendocrine carcinoma of gynecologic origin (NEC-GYN) was detected by GATK Haplotype Caller. Figure panels representing **A)** various variants classifications; **B)** variant type; **C)** SNV class; **D)** numbers of variants in each sample; and **E)** frequently mutated genes in our NEC-GYN cohort.

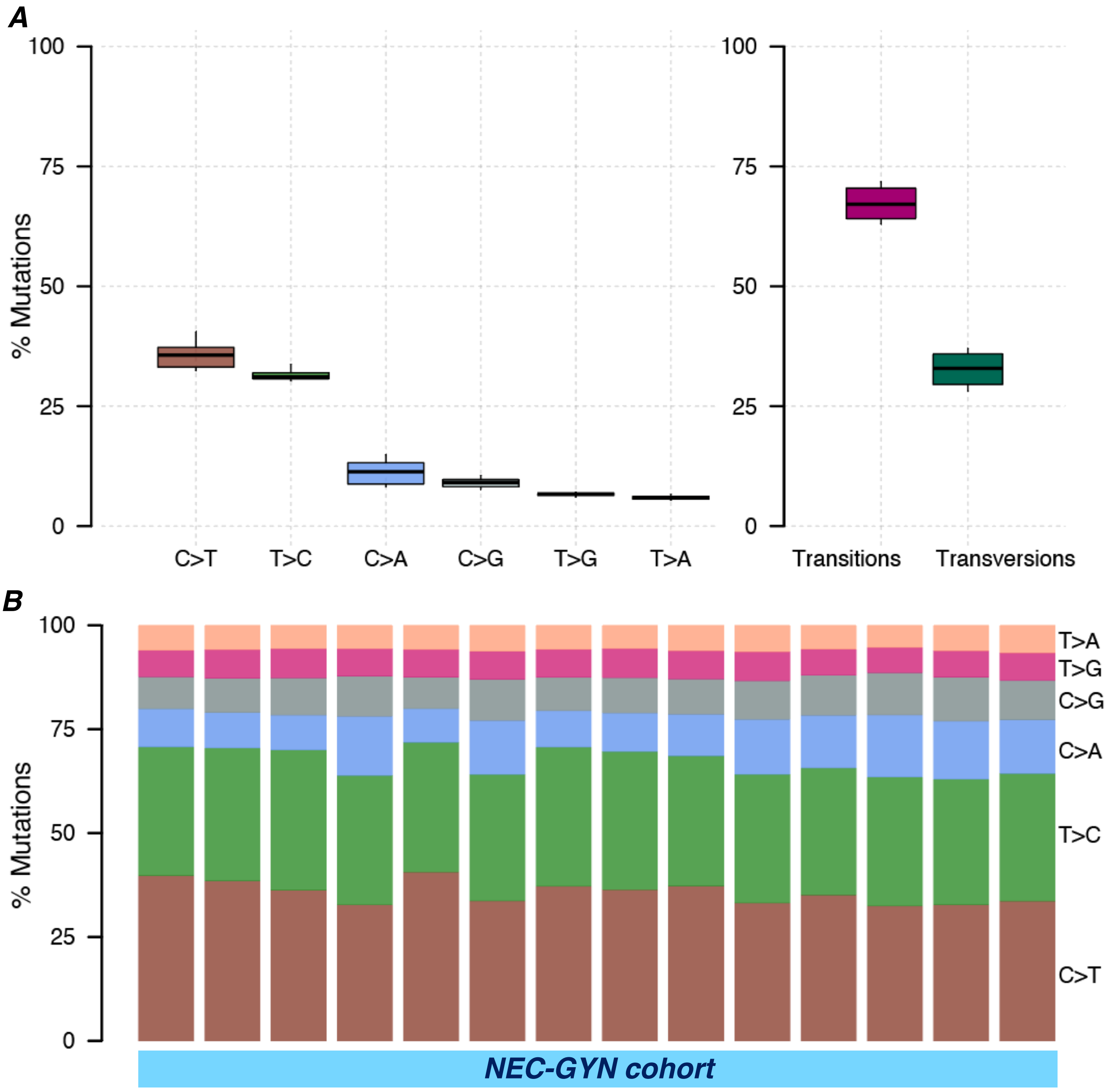

Supplementary Fig. 2. A) Percentages of various CNV classes in NEC-GYN (left) and transition & transversion mutations (right); and **B)** percentages of various CNV classes in various tumor sample in our cohort.

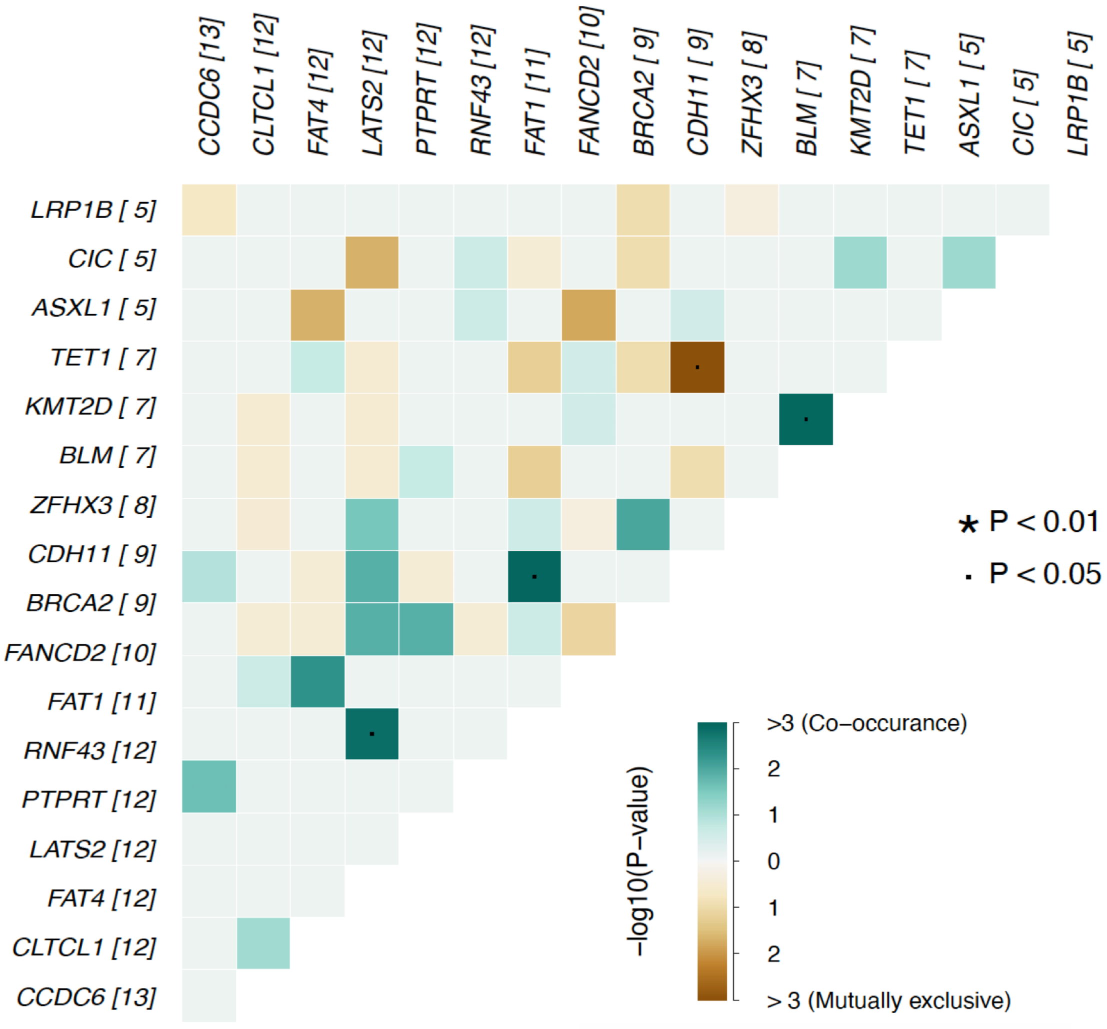

Supplementary Fig. 3. Mutually exclusive or co-occurring set of genes. Numbers in parenthesis indicate for the given gene mutated in total tumor samples out of 14 (cohort size). Pair-wise Fisher's Exact test was used to detect significant pair of genes.

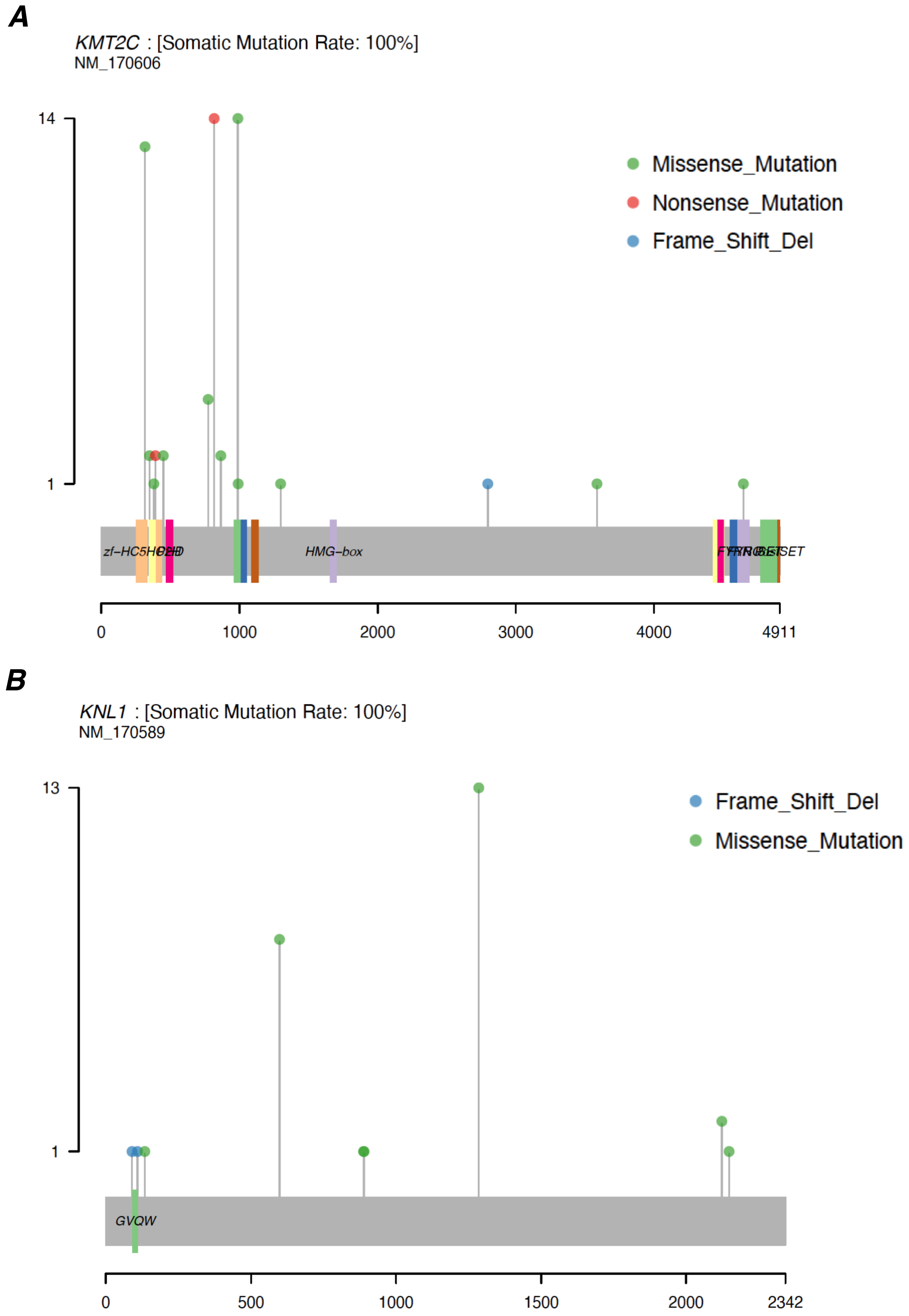

Supplementary Fig. 4. Lollipop plot showing the mutations (amino acid changes) in **A**) *KMT2C*; and **B**) *KNL1* gene in NEC-GYN.

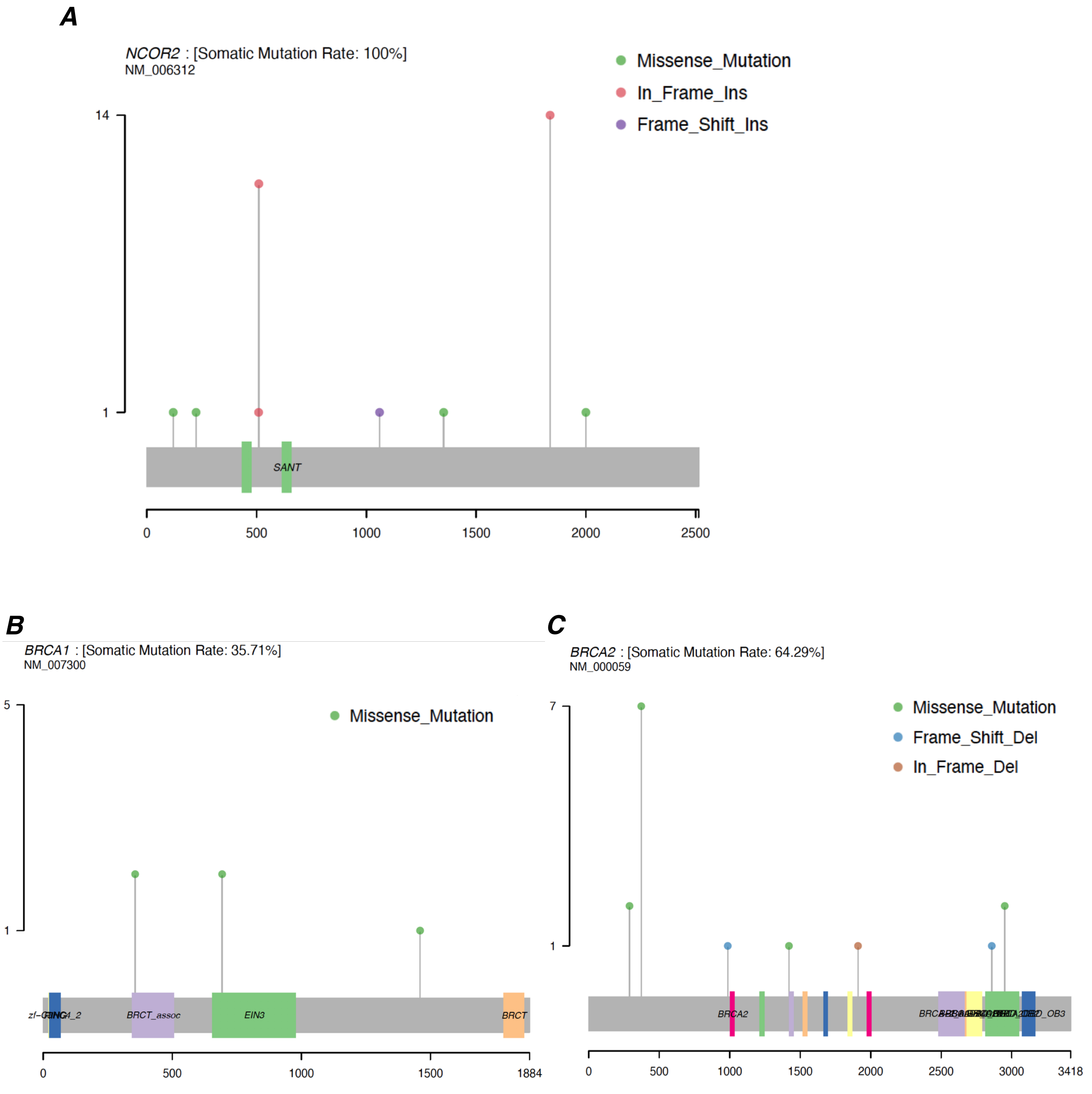

Supplementary Fig. 5. Lollipop plot showing the mutations (amino acid changes) in **A**) *NCOR2*; **B**) *BRCA1*; and **C**) *BRCA2* gene in NEC-GYN.

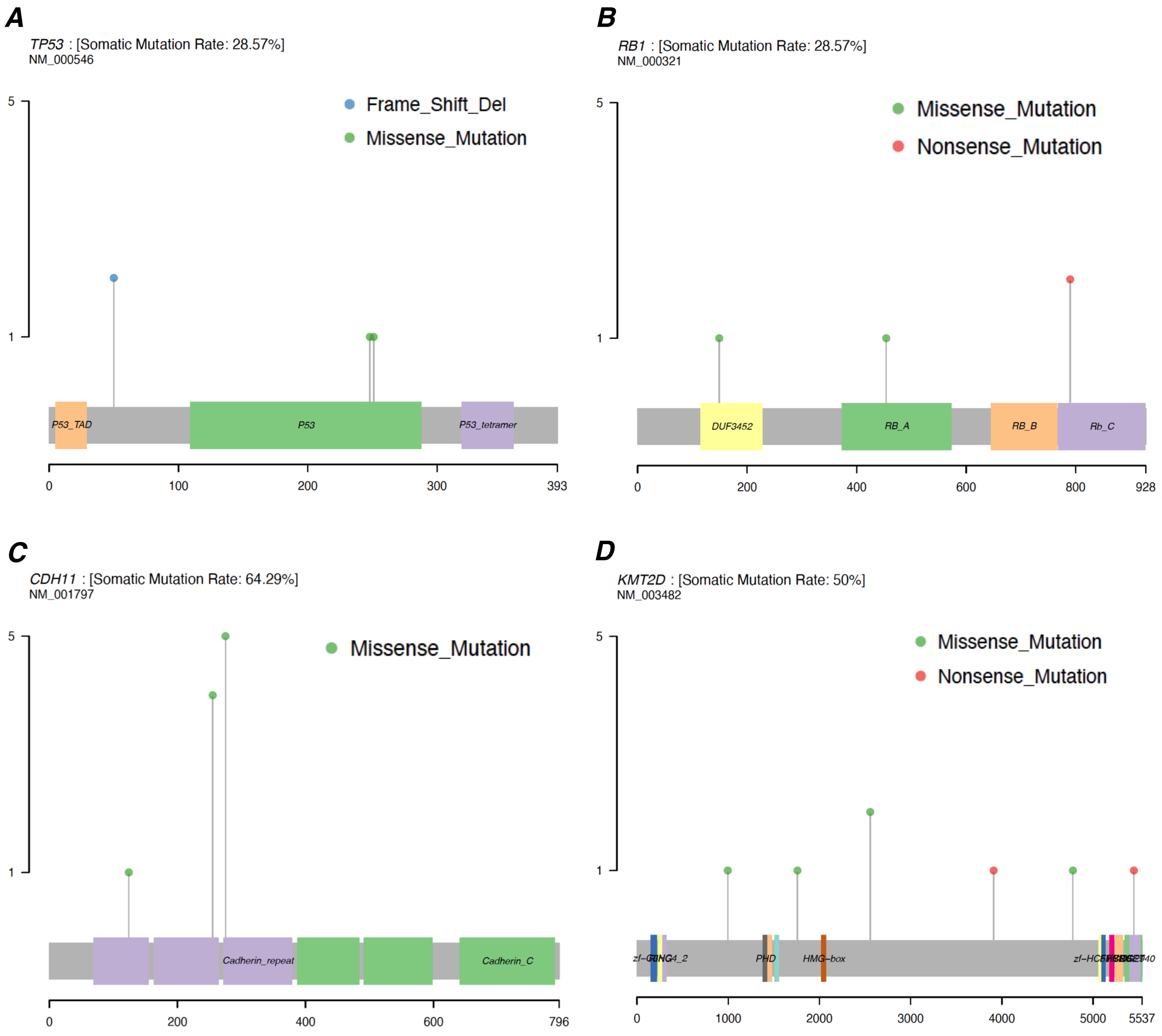

Supplementary Fig. 6. Lollipop plot showing the mutations (amino acid changes) in **A**) *TP53*; and **B**) *RB1* gene. *TP53* and *RB1* genes are highly mutated in SCLC but moderately mutated in our NEC-GYN cohort. Lollipop plot showing the mutations (amino acid changes) in **C**) *CDH11*; and **D**) *KMT2D* gene.

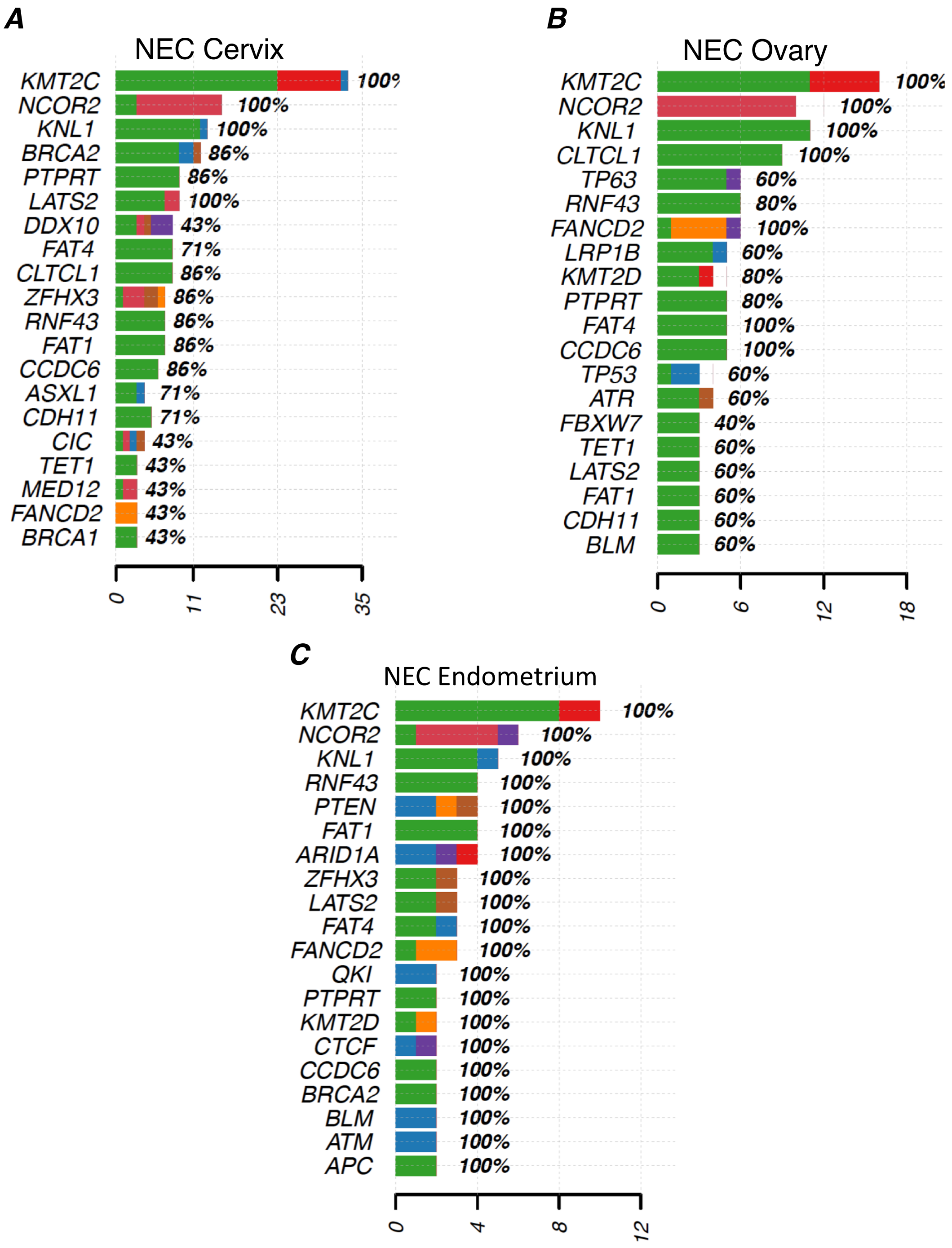

Supplementary Fig. 7. Cervical NEC samples showing higher number of mutations within individual genes compared to Ovary and Endometrial NEC. Frequently mutated genes in **A**) cervical; **B**) ovarian; and **C**) endometrial carcinoma. X-axis representing number of mutations. See Supplementary Fig.1 for color code for various variants.

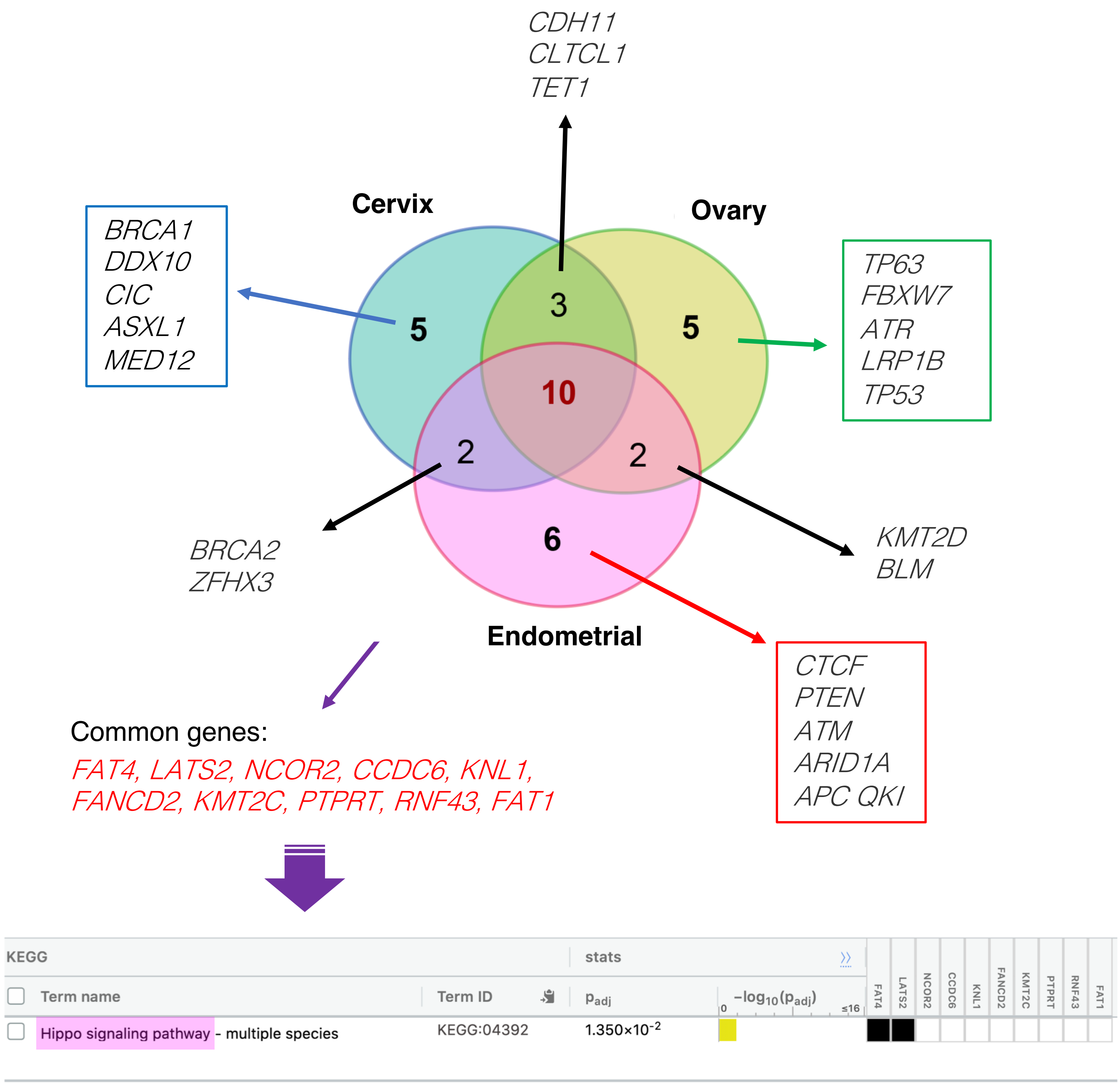

Supplementary Fig. 8. Venn diagram representing common mutated genes between Cervical, Ovarian, and Endometrial NEC (top 20 frequently mutated genes from each of three groups were used). The altered genes that were common between tumors from three gynecologic sites are significantly enriched for the Hippo signaling pathway (bottom).

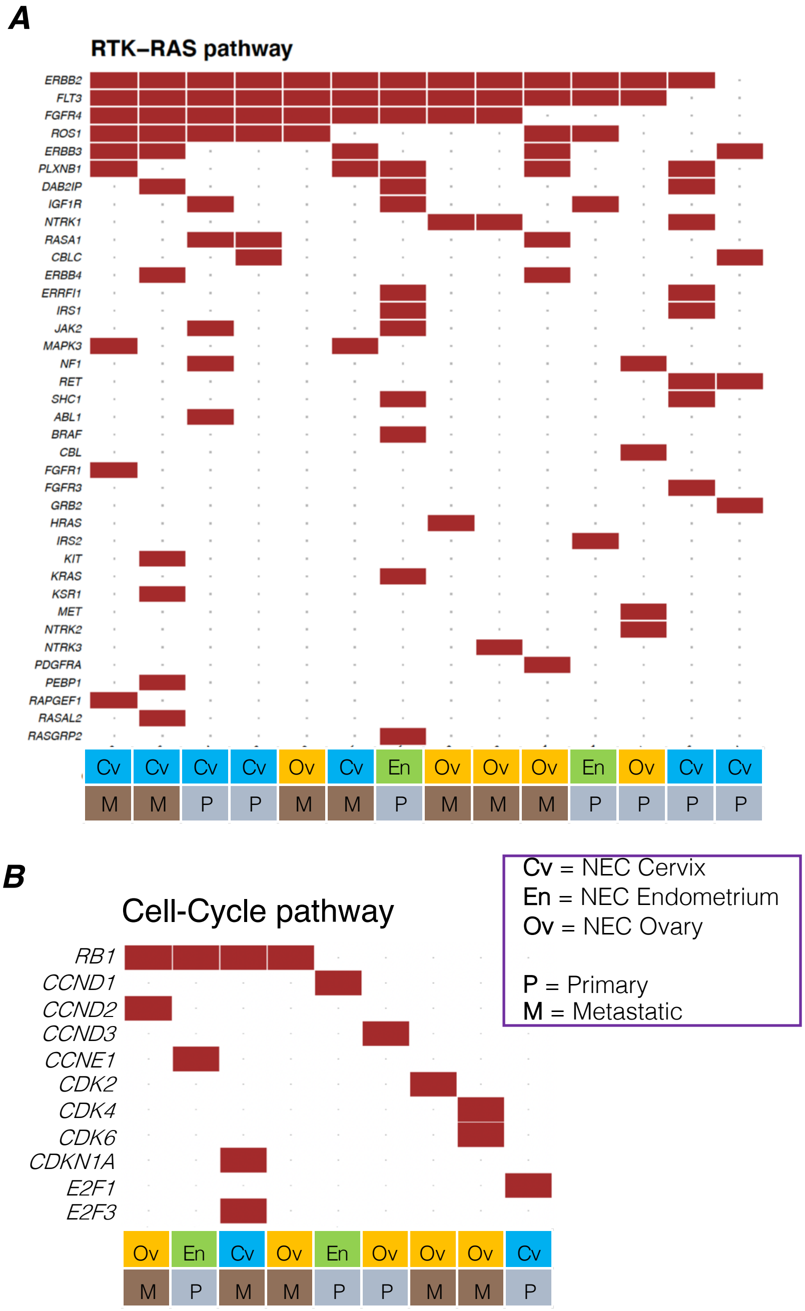

Supplementary Fig. 9. Oncogenic pathways affected in NEC-GYN. **A**) RTK-RAS pathway; and **B**) Cell-Cycle pathway.

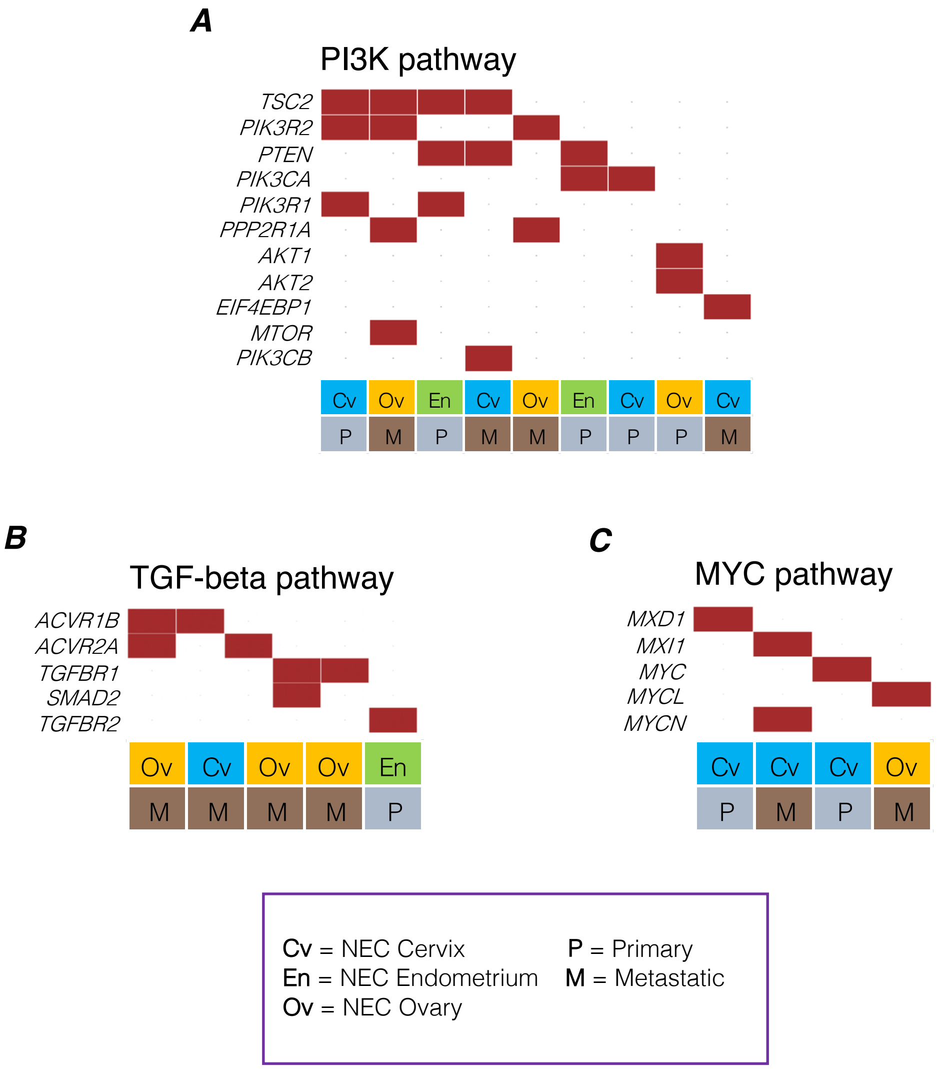

Supplementary Fig. 10. Oncogenic pathways affected in NEC-GYN. **A**) PI3K pathway; **B**) TGF-beta; and **C**) MYC pathway.

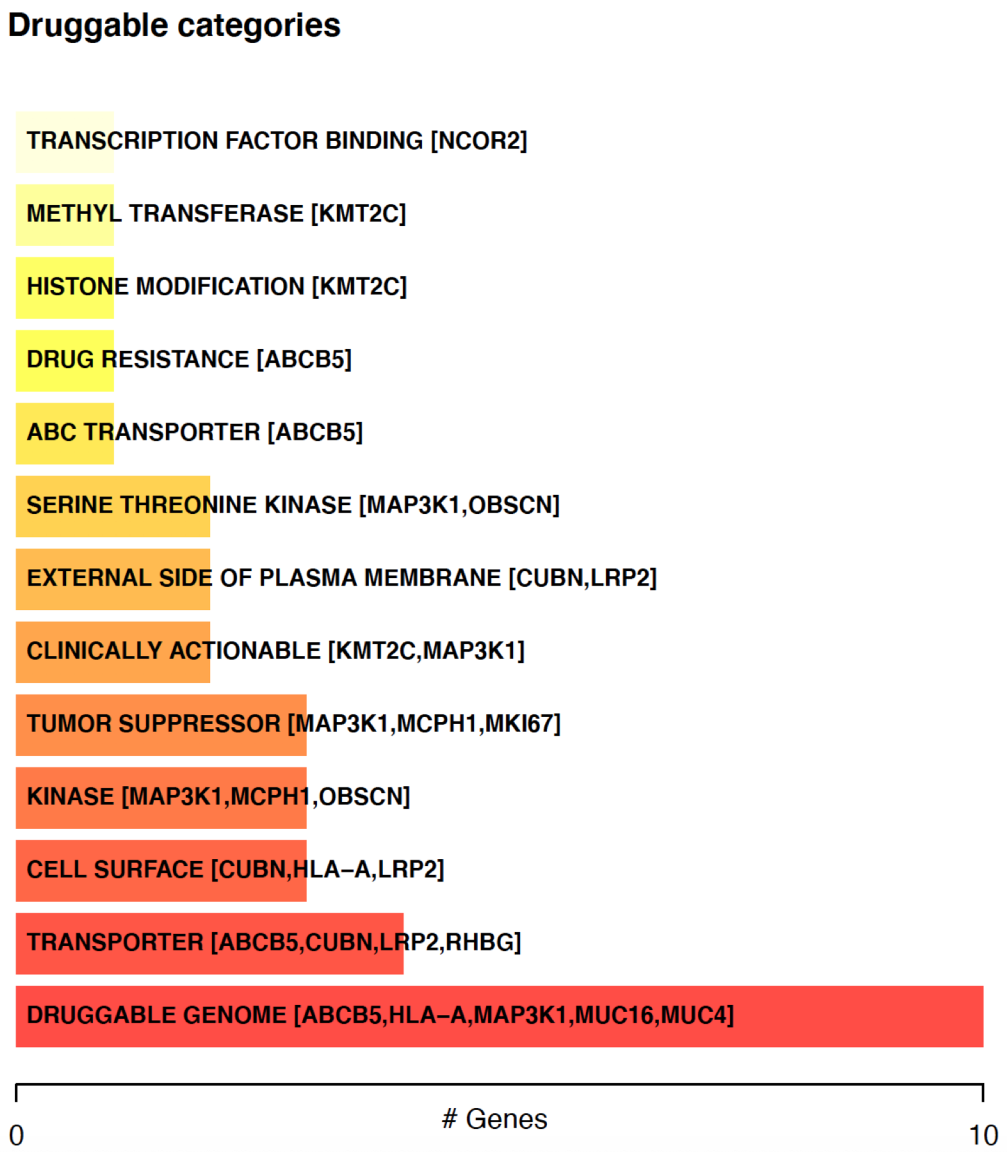

Supplementary Fig. 11. Various potentially druggable targets were identified by drug-gene interactions analysis using GDIdb.

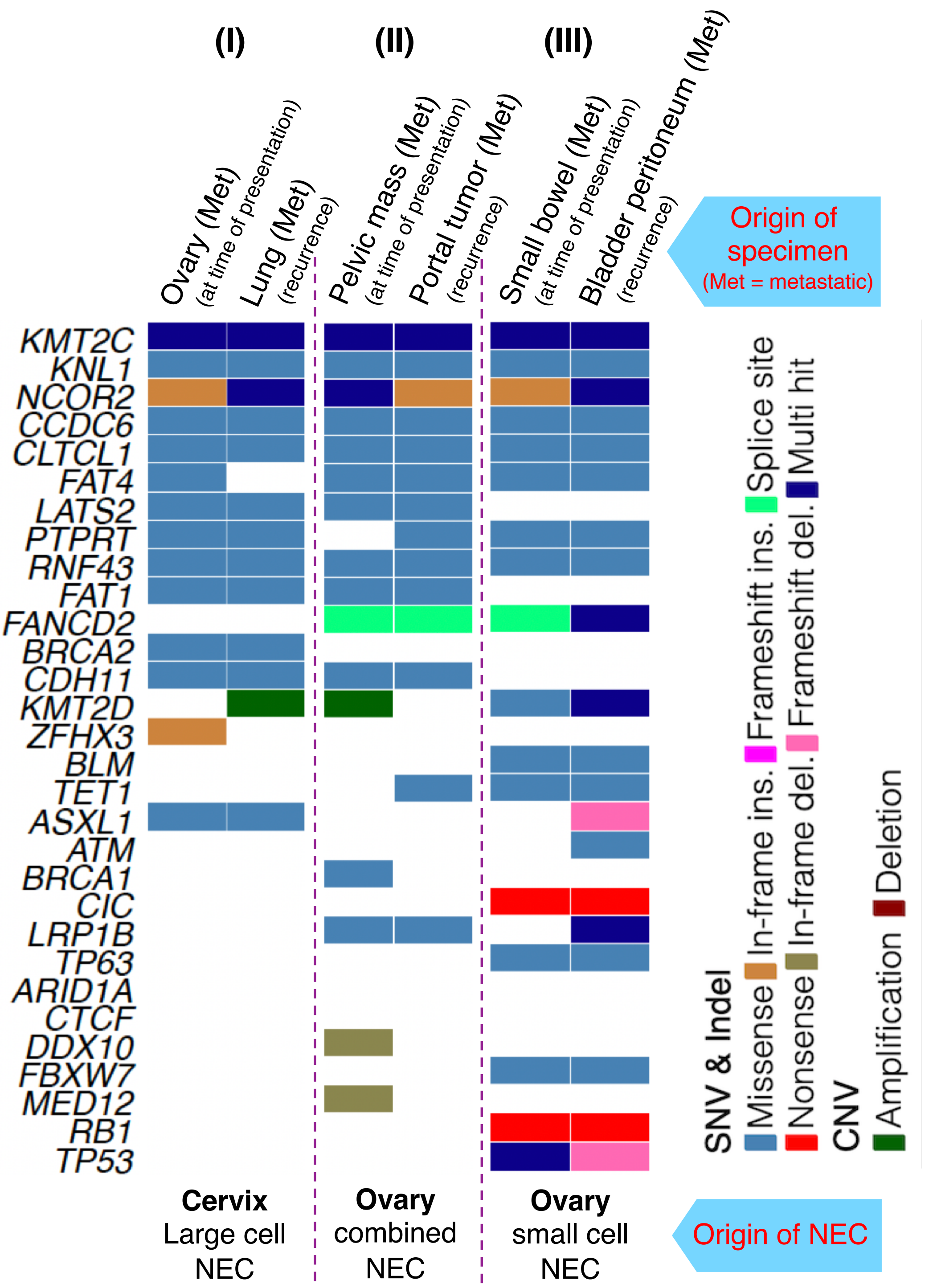

Supplementary Fig. 12. Mutational landscapes of the specimen obtained from the same patients. Patient III (ovarian NEC, small cell histology) presenting mutations in new genes (*ASXL1*, *ATM*, and *LRP1B*) and additional mutations (multi hit) in the same genes (*NCOR2*, *FANCD2*, and *KMT2D*) after recurrence.

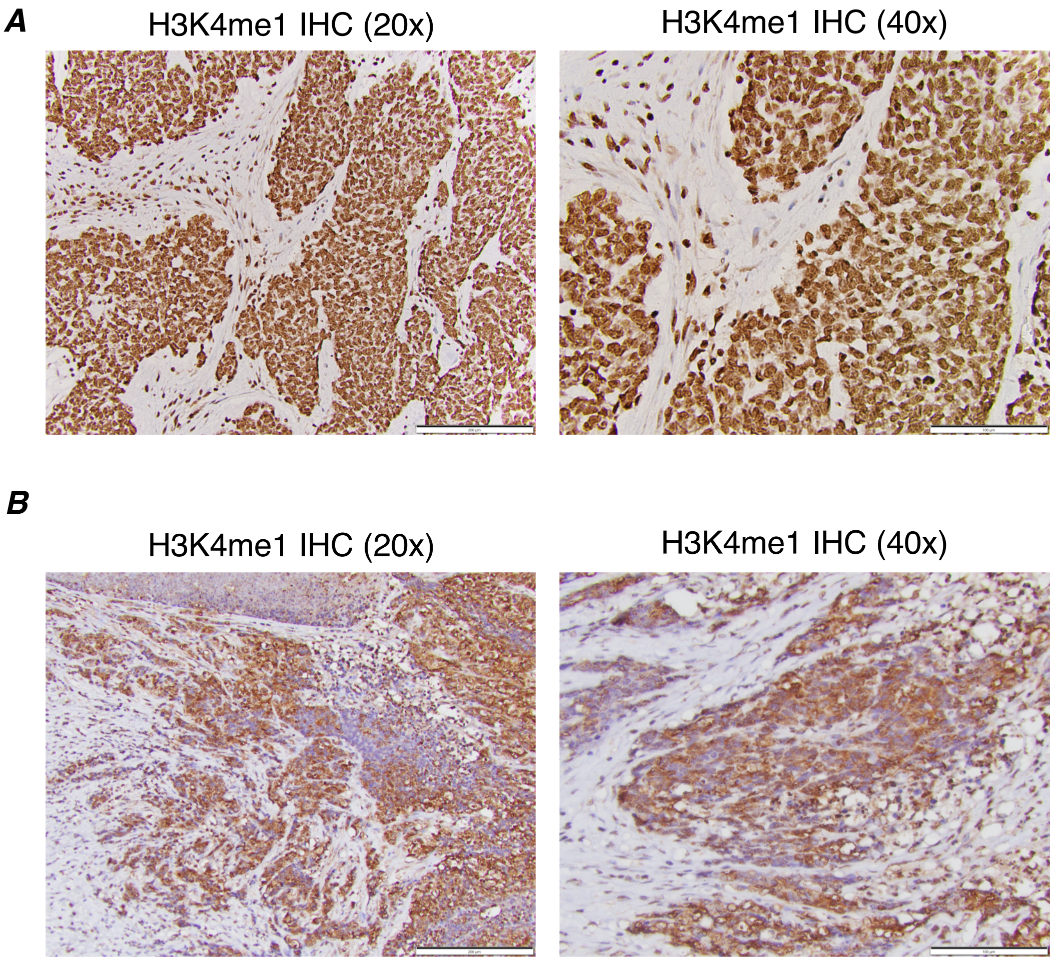

Supplementary Fig. 13. A) Representative H3K4me1 IHC images (20x and 40x) of an ovarian high-grade NEC. Twelve out of fourteen specimen exhibit strong staining. **B)** Representative H3K4me1 IHC images (20x and 40x) of a endometrial high-grade NEC. Tow out of fourteen specimens show strong staining in 70-80% tumor cells and week to moderate staining in 20-30% cells.

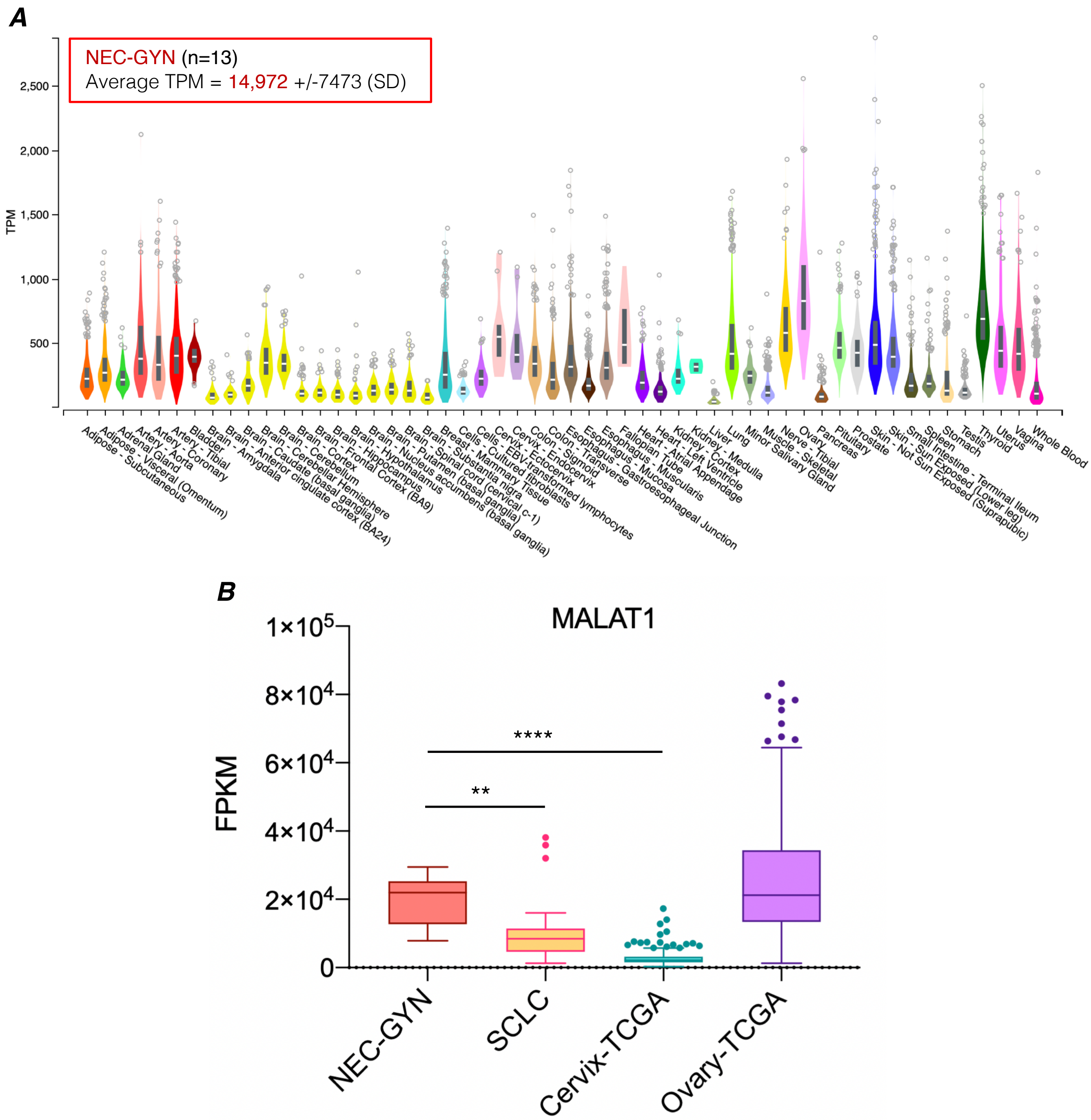

Supplementary Fig. 14. A) MALAT1 expression levels across various normal human tissues from GETx. TPM value of MALAT1 in NEC-GYN (shown in the box) is much higher than all of the GETx normal tissues. **B**) MALAT1 expression level in NEC-GYN (n=13) compared to SCLC (n=29) and TCGA cohorts of cervical (n=304) and ovarian (n=303) cancers (Tukey, **P = 0.0012, ****P > 0.0001 by two-tailed Mann–Whitney U test).

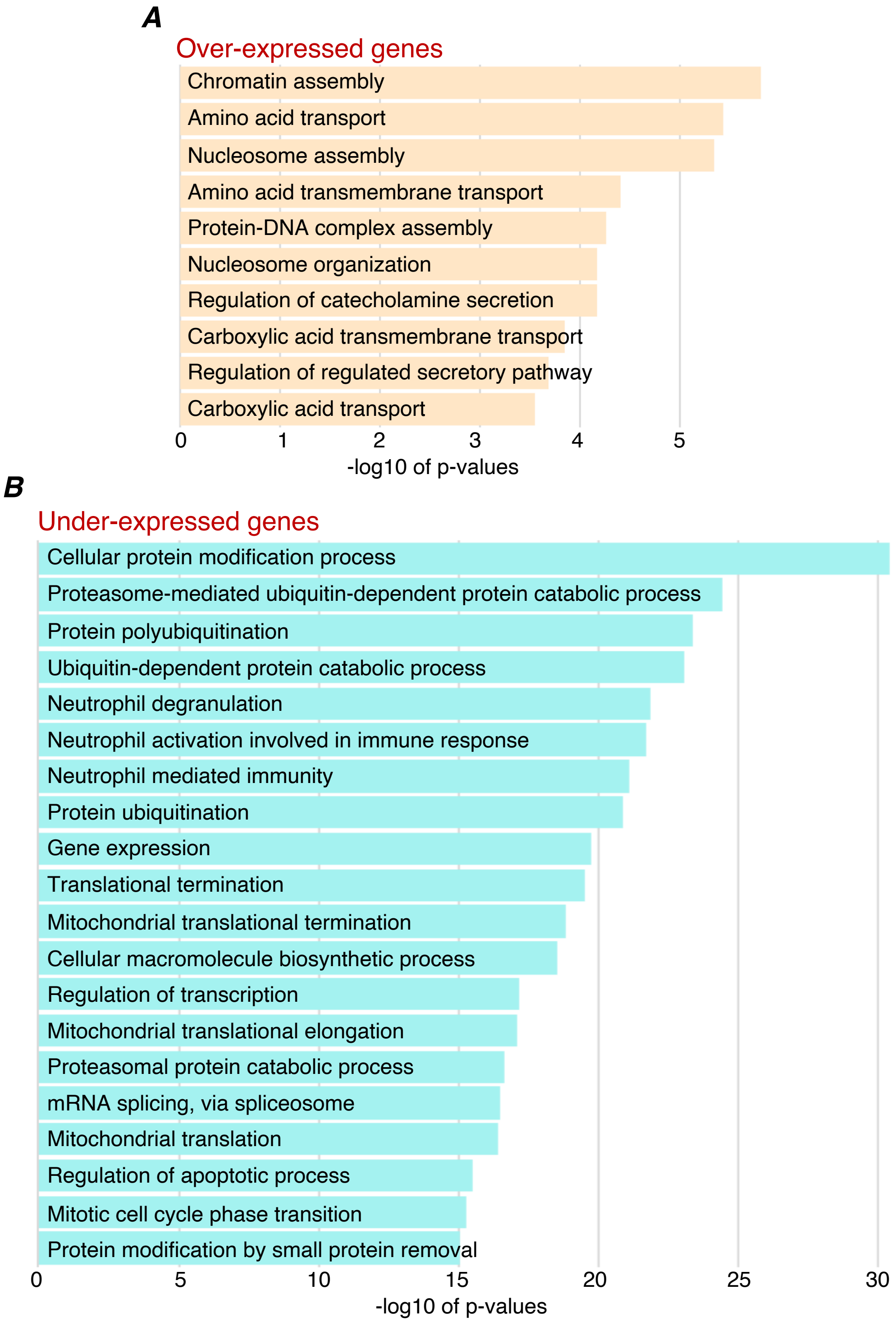

Supplementary Fig. 15. GO analysis of differentially expressed genes in neuroendocrine carcinoma of cervix (n=8) from NEC-GYN cohort compared to cervical cancer from TCGA (n=304). Significantly enriched pathways in **A**) over-expressed genes (4-folds cutoff); and **B**) under-expressed genes (10-folds cutoff).

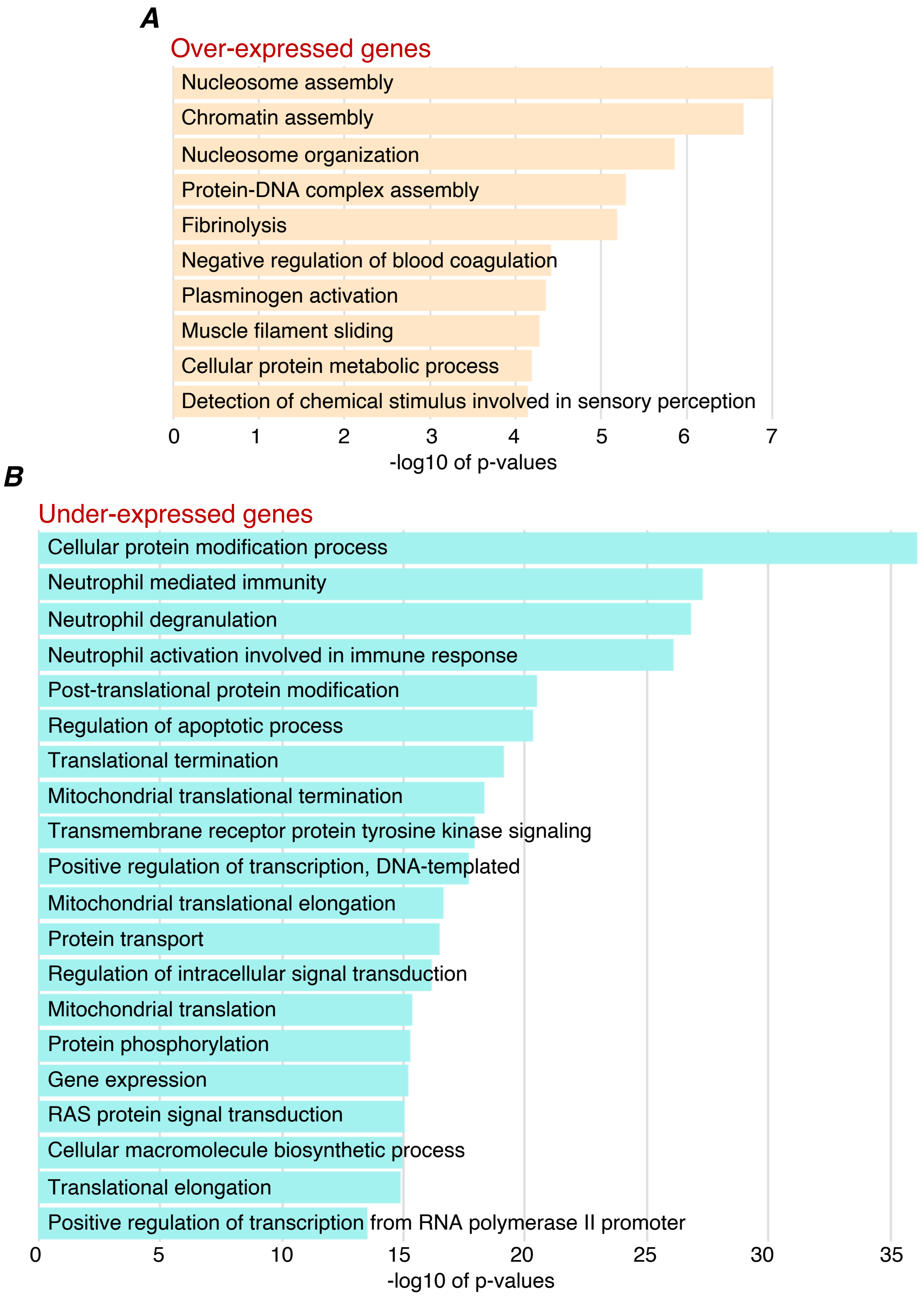

Supplementary Fig. 16. GO analysis of differentially expressed genes in neuroendocrine carcinoma of ovary (n=4) from NEC-GYN cohort compared to ovarian cancer from TCGA (n=303). Significantly enriched pathways in **A**) over-expressed genes (4-folds cutoff); and **B**) under-expressed genes (10-folds cutoff).

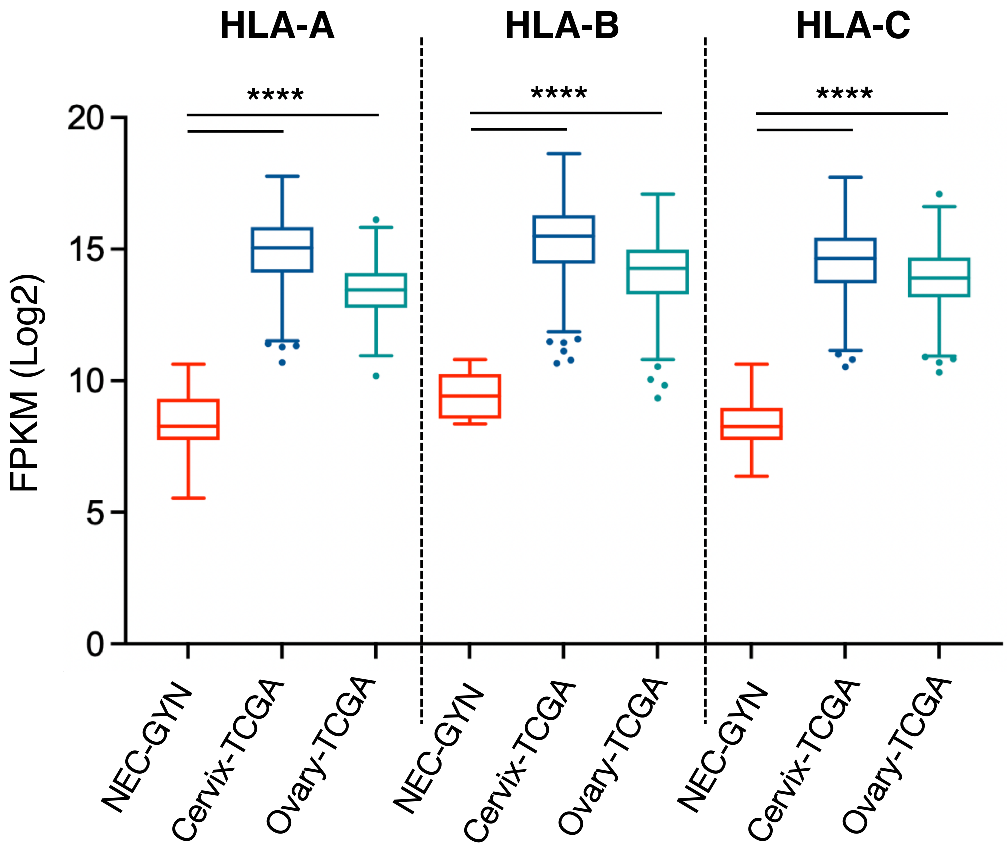

Supplementary Fig. 17. Comparison of HLA class I (HLA-A, HLA-B, and HLA-C) genes expression in NEC-GYN (n=13), cervical (TCGA, n=304), and ovarian (TCGA, n=303) cancers (Tukey, ****P > 0.0001 by two-tailed Mann–Whitney U test).

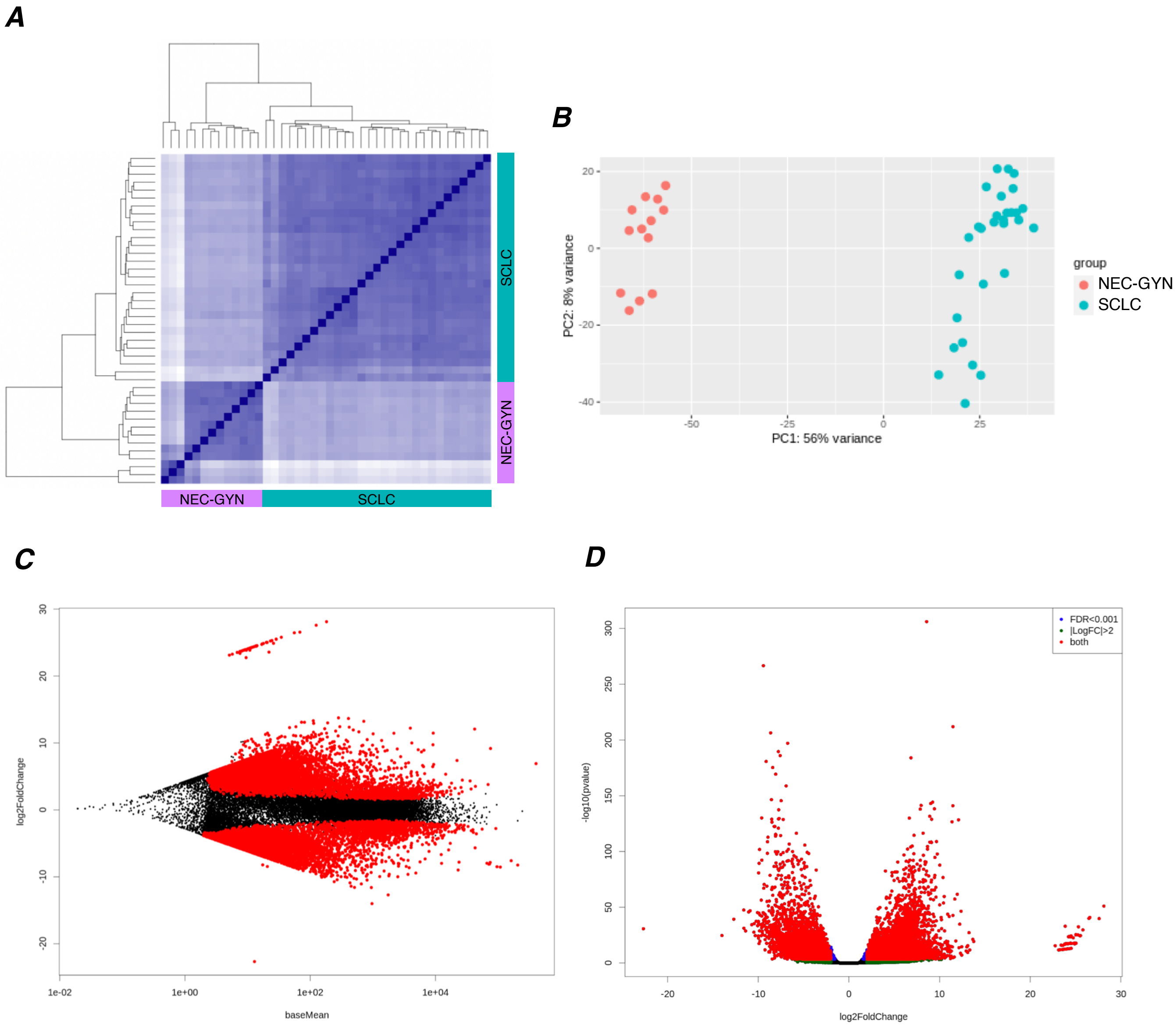

Supplementary Fig. 18. Comparison of NEC-GYN (n=13) with SCLC (n=29). **A**) Samples clustering; and **B**) PCA plot are shown. Differential gene expressions in NEC-GYN vs SCLC are represented by **C**) MA plot; and **D**) Volcano plot.

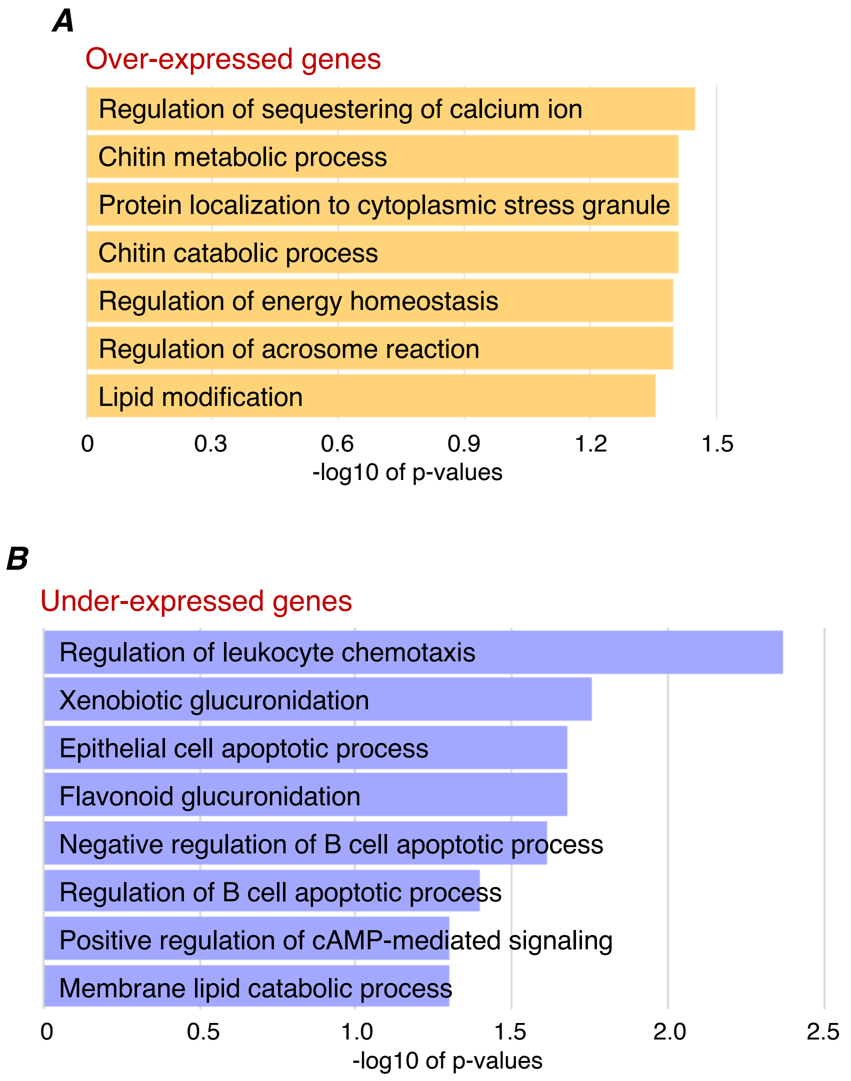

Supplementary Fig. 19. GO analysis of differentially expressed genes in NEC-GYN (n=13) compared to SCLC (n=29, p-adj <0.001, fold-change >2). Significantly enriched pathways in **A**) over-expressed genes; and **B**) under-expressed genes.

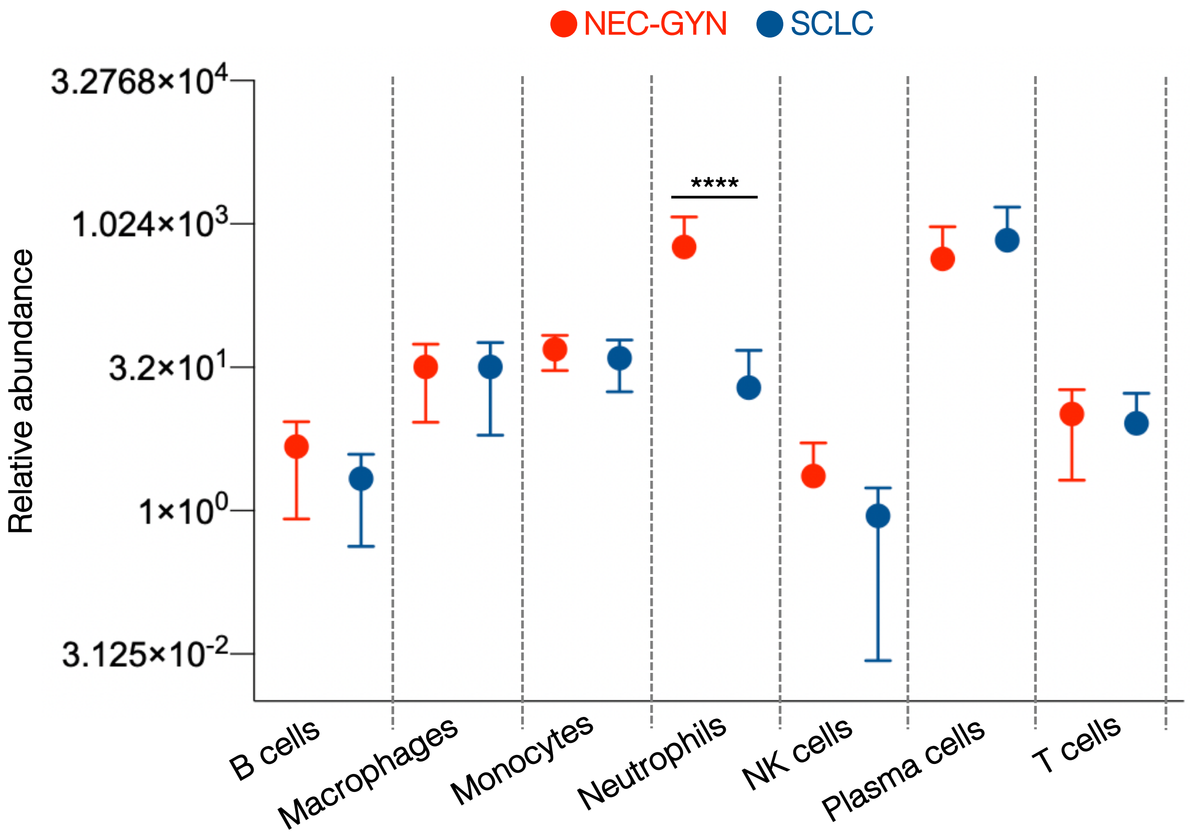

Supplementary Fig. 20. Comparison of relative abundance of immune cells in NEC-GYN (n=13) and SCLC (n=29) tumors based on network-based deconvolution (ImSig) analysis (mean with SD, ****P ≤ 0.0001 by two-tailed Mann–Whitney U test).

A

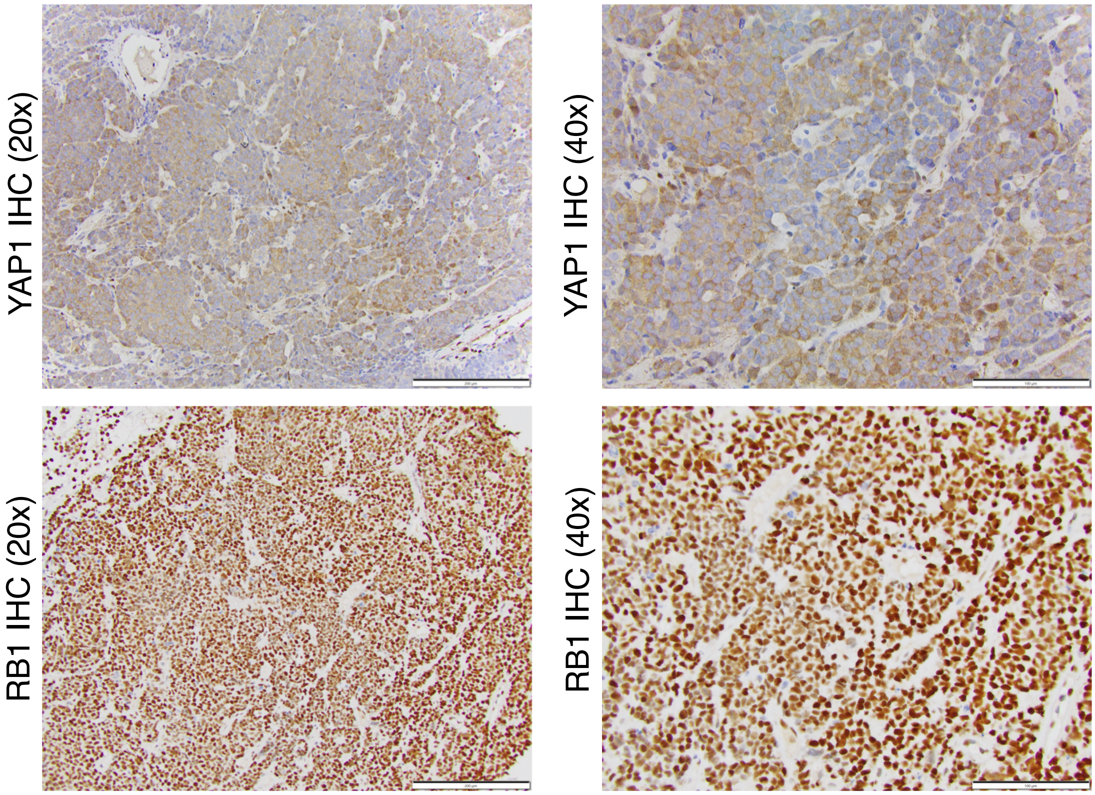

B

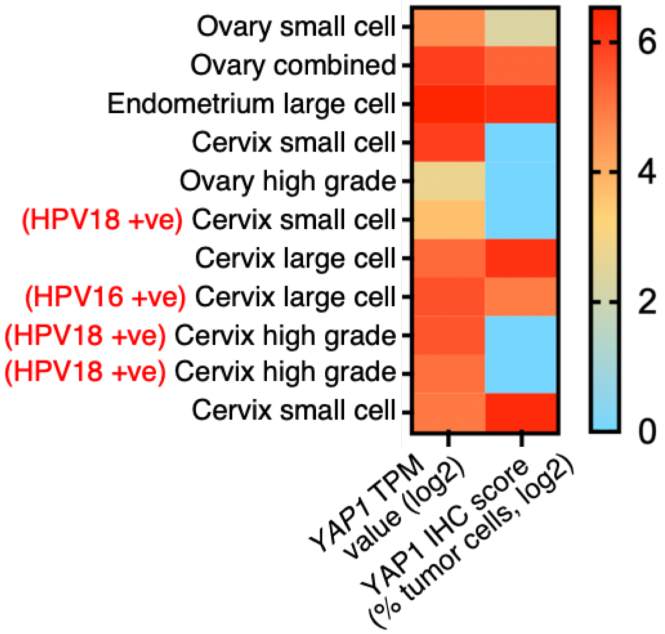

C

RB1 IHC (% tumor cells)	NEC-Cervix (tumor #)	HPV status
70-90+	2	Negative
40-65	1	Negative
10-35	4	Positive

Supplementary Fig. 21. A) Representative YAP1 and RB1 IHC images (20x and 40x) of a cervix small cell neuroendocrine carcinoma. **B)** Correlation of *YAP1* RNA level (TPM, transcript per million) with protein expression (IHC) and HPV status. **C)** Correlation of RB1 expression (IHC) with HPV.
