# Supplementary material for: Genomic analyses of high‐grade neuroendocrine gynecological malignancies reveal a unique mutational landscape and therapeutic vulnerabilities
Source: Mol Oncol. 2021 Jul 22;15(12):3545–58. doi: 10.1002/1878-0261.13057 (PMC8637558; doi:10.1002/1878-0261.13057)
Supplement: Supplementary file 3 — Table S2. Copy number variations (CNV) detected by XHMM. [file MOL2-15-3545-s003.pdf]

Supplementary Table 2. Copy number variations (CNV) detected by XHMM
(Table is sorted by chromosome intervals)

SAMPLE_ID	SAMPLE	CNV	INTERVAL	AFFECTED GENES
TKHS190002641	NEC Ovary	DUP	chr1:10376545-10419749	*KIF1B, PGD*
TKHS190002641	NEC Ovary	DUP	chr1:109739833-109985172	*AHCYL1, CSF1, GSTM3, EPS8L3*
TKHS190002657	NEC Ovary	DEL	chr1:11502678-11787493	*MAD2L2, FBXO2, FBXO6, DRAXIN, MTHFR, AGTRAP, DISP3, FBXO44*
TKHS190002657	NEC Ovary	DEL	chr1:11858214-12207020	*MIR4632, NPPB, PLOD1, MIIP, TNFRSF1B, KIAA2013, TNFRSF8, MFN2*
TKHS190002646	NEC Cervix	DUP	chr1:11956917-11999095	*PLOD1, MFN2*
TKHS190002641	NEC Ovary	DUP	chr1:12774868-12893474	*HNRNPCL1, PRAMEF10, PRAMEF12, PRAMEF4, PRAMEF11, HNRNPCL3, PRAMEF1, PRAMEF2*
TKHS190002646	NEC Cervix	DUP	chr1:1309086-1629566	*ACAP3, PUSL1, MIB2, MRPL20-AS1, DVL1, ATAD3C, SSU72, TMEM240, ANKRD65, MXRA8, INTS11, AURKAIP1, MRPL20, ATAD3A, TMEM88B, FNDC10, VWA1, CPTP, CCNL2, TAS1R3, ATAD3B*
TKHS190002653	NEC Ovary	DEL	chr1:1310634-1336515	*PUSL1, DVL1, INTS11, CPTP, TAS1R3*
TKHS190002657	NEC Ovary	DEL	chr1:1315404-1387582	*DVL1, MXRA8, INTS11, AURKAIP1, CPTP, CCNL2, TAS1R3*
TKHS190002657	NEC Ovary	DEL	chr1:1436807-1545002	*ATAD3C, SSU72, TMEM240, ATAD3A, VWA1, ATAD3B*
TKHS190002655	NEC Cervix	DEL	chr1:145918432-145995561	*NBPF10, NBPF20, TXNIP, LIX1L, ANKRD34A, POLR3GL, PEX11B, RBM8A*
TKHS190002656	NEC Cervix	DUP	chr1:147252623-147271005	*NA, NA, NA, CHD1L*
TKHS190002657	NEC Ovary	DEL	chr1:150497530-150556793	*MIR4257, FALEC, ECM1, ADAMTSL4, TARS2*
TKHS190002657	NEC Ovary	DEL	chr1:152622834-152884773	*C1orf68, LCE4A, LCE2B, LCE1A, LCE1B, LCE1C, LCE1D, LCE1E, LCE1F, LCE2A, LCE2C, LCE2D, LCE3A, SMCP, KPRP, LCE6A*
TKHS190002644	NEC Cervix	DUP	chr1:153310611-153360838	*PGLYRP3, PGLYRP4, S100A9*
TKHS190002644	NEC Cervix	DUP	chr1:155023117-155058474	*DCST1-AS1, DCST2, DCST1, ADAM15*
TKHS190002644	NEC Cervix	DUP	chr1:155175386-155192843	*GBAP1, MTX1, THBS3*
TKHS190002647	NEC Endometrium	DEL	chr1:155202812-155241112	*GBA, GBAP1, MTX1, THBS3*
TKHS190002643	NEC Cervix	DUP	chr1:155203086-155240717	*GBA, GBAP1, MTX1, THBS3*
TKHS190002641	NEC Ovary	DUP	chr1:156566390-156652966	*IQGAP3, NAXE, TTC24, GPATCH4, HAPLN2, BCAN*
TKHS190002647	NEC Endometrium	DEL	chr1:156726906-156768239	*HDGF, RRNAD1, PRCC, MRPL24, ISG20L2*
TKHS190002645	NEC Endometrium	DUP	chr1:15727014-15738245	*PLEKHM2, SLC25A34*
TKHS190002644	NEC Cervix	DUP	chr1:161001020-161124462	*TSTD1, KLHDC9, ARHGAP30, NIT1, F11R, PFDN2, USF1, NECTIN4, DEDD*
TKHS190002644	NEC Cervix	DUP	chr1:161166848-161202480	*NDUFS2, PPOX, B4GALT3, ADAMTS4*
TKHS190002657	NEC Ovary	DEL	chr1:16125215-16207210	*ARHGEF19, EPHA2*
TKHS190002650	NEC Ovary	DUP	chr1:16129434-16206543	*ARHGEF19, EPHA2*
TKHS190002643	NEC Cervix	DEL	chr1:16922019-16946991	*CROCC*
TKHS190002657	NEC Ovary	DEL	chr1:16987046-16996164	*ATP13A2*
TKHS190002642	NEC Ovary	DUP	chr1:171781968-173050962	*MIR3120, DNM3OS, DNM3, FASLG, SUCO, EEF1AKNMT, PIGC, TNFSF18, C1orf105*
TKHS190002657	NEC Ovary	DEL	chr1:175093990-175128746	*TNN*

TKHS190002646	NEC Cervix	DUP	chr1:1752904-1804581	*GNB1, NADK*
TKHS190002641	NEC Ovary	DUP	chr1:1752904-3816370	*LOC100129534, NA, TTC34, MIR4251, RER1, TNFRSF14-AS1, TPRG1L, PRXL2B, ACTRT2, CCDC27, CALML6, MEGF6, FAAP20, GABRD, ARHGEF16, GNB1, TMEM52, HES5, SMIM1, PRDM16-DT, WRAP73, PEX10, PANK4, PRKCZ, TP73-AS1, LRRC47, PRDM16, SKI, NADK, MIR551A, TP73, MMEL1, MORN1, CFAP74, TNFRSF14, PLCH2, CEP104*
TKHS190002656	NEC Cervix	DUP	chr1:179639839-180080661	*FAM163A, TDRD5, TOR1AIP2, TOR1AIP1, CEP350*
TKHS190002653	NEC Ovary	DEL	chr1:1923367-1959209	*CFAP74*
TKHS190002646	NEC Cervix	DUP	chr1:1923367-2229735	*FAAP20, GABRD, PRKCZ, SKI, CFAP74*
TKHS190002641	NEC Ovary	DUP	chr1:19267300-19304386	*LOC100506730, AKR7A3, AKR7L, AKR7A2*
TKHS190002646	NEC Cervix	DUP	chr1:19269272-19304386	*LOC100506730, AKR7A3, AKR7L, AKR7A2*
TKHS190002657	NEC Ovary	DEL	chr1:19270748-19327412	*LOC100506730, AKR7A3, AKR7L, SLC66A1, AKR7A2*
TKHS190002656	NEC Cervix	DUP	chr1:193081364-193212477	*MIR1278, GLRX2, RO60, CDC73, B3GALT2*
TKHS190002654	NEC Cervix	DEL	chr1:200857754-200913629	*CAMSAP2, GPR25, INAVA*
TKHS190002653	NEC Ovary	DUP	chr1:201009266-201110269	*KIF21B, CACNA1S*
TKHS190002642	NEC Ovary	DUP	chr1:201075495-201110269	*CACNA1S*
TKHS190002641	NEC Ovary	DEL	chr1:203020793-203217133	*CHI3L1, CHIT1, ADORA1, MYBPH, MYOG, PPFIA4, TMEM183A*
TKHS190002642	NEC Ovary	DUP	chr1:203048227-203225870	*CHI3L1, CHIT1, ADORA1, MYBPH, MYOG, PPFIA4*
TKHS190002647	NEC Endometrium	DEL	chr1:203165056-203503720	*CHI3L1, CHIT1, ADORA1, FMOD, OPTC, MYBPH, PRELP, LINC01136, BTG2*
TKHS190002653	NEC Ovary	DUP	chr1:203165261-203217133	*CHI3L1, CHIT1, ADORA1, MYBPH*
TKHS190002654	NEC Cervix	DUP	chr1:20342432-20354263	*VWA5B1*
TKHS190002644	NEC Cervix	DUP	chr1:204433316-204450017	*PIK3C2B*
TKHS190002647	NEC Endometrium	DEL	chr1:205256330-205531378	*LEMD1-AS1, MIR135B, CDK18, KLHDC8A, NUAK2, LEMD1, TMCC2*
TKHS190002654	NEC Cervix	DEL	chr1:205845325-206012649	*SLC26A9, LOC284581*
TKHS190002644	NEC Cervix	DUP	chr1:205928827-205935820	*SLC26A9*
TKHS190002642	NEC Ovary	DUP	chr1:208027243-208060837	*PLXNA2*
TKHS190002656	NEC Cervix	DUP	chr1:211987886-212080750	*MIR3122, INTS7, DTL*
TKHS190002657	NEC Ovary	DEL	chr1:21219955-21290198	*ECE1*
TKHS190002656	NEC Cervix	DUP	chr1:212796517-212951818	*TATDN3, SPATA45, FLVCR1, FLVCR1-DT, VASH2*
TKHS190002656	NEC Cervix	DUP	chr1:220073967-220145653	*BPNT1, RNU5F-1, MIR194-1, MIR215, IARS2*
TKHS190002656	NEC Cervix	DUP	chr1:228103134-228350057	*MRPL55, IBA57, GUK1, GJC2, OBSCN-AS1, C1orf35, OBSCN*
TKHS190002648	NEC Cervix	DEL	chr1:228277505-228338372	*OBSCN*
TKHS190002650	NEC Ovary	DUP	chr1:228277505-228375824	*OBSCN*
TKHS190002657	NEC Ovary	DEL	chr1:22896411-22965338	*EPHB2*
TKHS190002656	NEC Cervix	DUP	chr1:246889966-246945827	*ZNF670-ZNF695, AHCTF1, ZNF695*
TKHS190002653	NEC Ovary	DEL	chr1:2480183-2511683	*PANK4, PLCH2*

TKHS190002657	NEC Ovary	DEL	chr1:26543162-26573361	*MIR1976, RPS6KA1*
TKHS190002650	NEC Ovary	DUP	chr1:32161947-32224121	*DCDC2B, TXLNA, KPNA6, IQCC, TMEM234, CCDC28B, EIF3I*
TKHS190002650	NEC Ovary	DUP	chr1:32274198-32651322	*FAM229A, HDAC1, ZBTB8OS, LCK, BSDC1, RBBP4, MARCKSL1, ZBTB8A, ZBTB8B, TSSK3*
TKHS190002650	NEC Ovary	DUP	chr1:32769880-35014888	*MIR3605, TMEM35B, SMIM12, AZIN2, TMEM54, CSMD2, GJB4, HMGB4, RNF19B, A3GALT2, ZNF362, PHC2, AK2, FNDC5, GJA4, GJB3, GJB5, HPCA, CSMD2-AS1, TRIM62, KIAA1522, DLGAP3, S100PBP, LOC653160, ZSCAN20, C1orf94, YARS1, ZMYM6*
TKHS190002655	NEC Cervix	DEL	chr1:33096707-33368622	*MIR3605, AZIN2, A3GALT2, ZNF362, PHC2, TRIM62*
TKHS190002657	NEC Ovary	DEL	chr1:33527196-33583830	*CSMD2*
TKHS190002653	NEC Ovary	DEL	chr1:3414560-3512128	*MEGF6, ARHGEF16, PRDM16*
TKHS190002650	NEC Ovary	DUP	chr1:35449867-35716321	*C1orf216, NCDN, TFAP2E, PSMB2, KIAA0319L*
TKHS190002650	NEC Ovary	DUP	chr1:36008690-36355433	*COL8A2, AGO3, TRAPPC3, TEKT2, ADPRHL2, EVA1B, MAP7D1, SH3D21, STK40, THRAP3*
TKHS190002655	NEC Cervix	DEL	chr1:36172464-36291546	*MAP7D1, THRAP3*
TKHS190002657	NEC Ovary	DEL	chr1:36319260-36355433	*EVA1B, SH3D21, STK40*
TKHS190002646	NEC Cervix	DUP	chr1:3634997-6701800	*LINC01134, MIR4252, MIR4689, NA, ACOT7, PHF13, CCDC27, DFFB, ICMT, CHD5, NPHP4, LINC01777, ZBTB48, C1orf174, GPR153, SMIM1, RNF207, HES3, WRAP73, HES2, DNAJC11, AJAP1, TP73-AS1, PLEKHG5, LRRC47, RPL22, TP73, LINC01346, NOL9, TAS1R1, ESPN, KCNAB2, TNFRSF25, THAP3, CEP104, KLHL21*
TKHS190002650	NEC Ovary	DUP	chr1:36463806-37483611	*MIR4255, CSF3R, GRIK3, MRPS15, LINC01137, ZC3H12A*
TKHS190002650	NEC Ovary	DUP	chr1:37989548-38016420	*SF3A3, FHL3, UTP11*
TKHS190002641	NEC Ovary	DUP	chr1:3857604-3900186	*DFFB, C1orf174*
TKHS190002650	NEC Ovary	DUP	chr1:39626507-39969568	*PPIE, HEYL, MYCL, HPCAL4, TRIT1, OXCT2, BMP8B, NT5C1A, MFSD2A*
TKHS190002650	NEC Ovary	DUP	chr1:40222574-40641925	*ZMPSTE24, TMCO2, ZNF684, COL9A2, ZFP69, RLF, SMAP2, EXO5, ZFP69B, RIMS3*
TKHS190002657	NEC Ovary	DEL	chr1:40301182-40374223	*COL9A2, SMAP2*
TKHS190002641	NEC Ovary	DUP	chr1:43272723-43313425	*TMEM125, C1orf210, TIE1*
TKHS190002657	NEC Ovary	DEL	chr1:43272723-43352772	*TMEM125, C1orf210, MPL, TIE1*
TKHS190002646	NEC Cervix	DUP	chr1:43307142-43338720	*MPL, TIE1*
TKHS190002657	NEC Ovary	DEL	chr1:43420153-43622003	*SZT2, PTPRF, HYI*
TKHS190002650	NEC Ovary	DUP	chr1:43578810-43622003	*PTPRF*
TKHS190002657	NEC Ovary	DEL	chr1:43992153-44248038	*KLF17, CCDC24, DMAP1, SLC6A9, ERI3*
TKHS190002646	NEC Cervix	DUP	chr1:44118908-44218755	*KLF17, DMAP1*
TKHS190002646	NEC Cervix	DUP	chr1:44760280-44762451	*KIF2C*
TKHS190002657	NEC Ovary	DEL	chr1:44802955-44832341	*PLK3, BTBD19, TCTEX1D4, PTCH2*
TKHS190002657	NEC Ovary	DEL	chr1:45621281-45623909	*CCDC17*
TKHS190002657	NEC Ovary	DEL	chr1:46032178-46036066	*MAST2*
TKHS190002657	NEC Ovary	DEL	chr1:46410398-46562843	*MKNK1-AS1, DMBX1, KNCN, FAAH, FAAHP1, MKNK1*
TKHS190002657	NEC Ovary	DEL	chr1:46799082-46941433	*CYP4A11, CYP4B1, CYP4Z2P*

TKHS190002657	NEC Ovary	DEL	chr1:47142108-47187427	*LINC00853, PDZK1IP1, CYP4A22*
TKHS190002657	NEC Ovary	DEL	chr1:51294391-51321825	*TTC39A*
TKHS190002657	NEC Ovary	DEL	chr1:52353173-52362746	*CC2D1B*
TKHS190002654	NEC Cervix	DUP	chr1:53047858-53134429	*PODN, SCP2, SLC1A7*
TKHS190002657	NEC Ovary	DEL	chr1:54757734-54887119	*LEXM, DHCR24, PARS2, TTC22*
TKHS190002657	NEC Ovary	DEL	chr1:5863265-6142605	*CHD5, NPHP4, KCNAB2*
TKHS190002641	NEC Ovary	DUP	chr1:5863265-6701800	*MIR4252, ACOT7, PHF13, ICMT, CHD5, NPHP4, ZBTB48, GPR153, RNF207, HES3, HES2, DNAJC11, PLEKHG5, RPL22, NOL9, TAS1R1, ESPN, KCNAB2, TNFRSF25, THAP3, KLHL21*
TKHS190002657	NEC Ovary	DEL	chr1:6245110-6385666	*ACOT7, GPR153, HES3*
TKHS190002646	NEC Cervix	DUP	chr1:8945887-9129027	*SLC2A7, SLC2A5, CA6, GPR157*
TKHS190002657	NEC Ovary	DEL	chr1:8945887-9264869	*SLC2A7, MIR34A, SLC2A5, CA6, GPR157, H6PD*
TKHS190002641	NEC Ovary	DUP	chr1:9104419-9264869	*MIR34A, GPR157, H6PD*
TKHS190002641	NEC Ovary	DUP	chr1:925942-1649637	*ACAP3, UBE2J2, PUSL1, B3GALT6, MIB2, SAMD11, MRPL20-AS1, DVL1, ATAD3C, LINC01342, TTLL10, NOC2L, SSU72, KLHL17, TMEM240, AGRN, C1QTNF12, RNF223, MIR200A, MIR200B, ANKRD65, SDF4, MXRA8, INTS11, C1orf159, AURKAIP1, MRPL20, ATAD3A, MIR429, HES4, SCNN1D, TMEM88B, FNDC10, VWA1, TNFRSF4, CPTP, CCNL2, TAS1R3, ATAD3B, PLEKHN1, PERM1, MMP23B, TNFRSF18, ISG15, CDK11B*
TKHS190002657	NEC Ovary	DEL	chr1:948131-1244275	*B3GALT6, LINC01342, TTLL10, NOC2L, KLHL17, AGRN, C1QTNF12, RNF223, MIR200A, MIR200B, SDF4, C1orf159, MIR429, HES4, TNFRSF4, PLEKHN1, PERM1, TNFRSF18, ISG15*
TKHS190002646	NEC Cervix	DUP	chr1:952000-1244275	*B3GALT6, LINC01342, TTLL10, NOC2L, KLHL17, AGRN, C1QTNF12, RNF223, MIR200A, MIR200B, SDF4, C1orf159, MIR429, HES4, TNFRSF4, PLEKHN1, PERM1, TNFRSF18, ISG15*
TKHS190002657	NEC Ovary	DEL	chr1:9611753-9736042	*TMEM201, CLSTN1, PIK3CD, PIK3CD-AS1*
TKHS190002653	NEC Ovary	DEL	chr1:961293-1244497	*B3GALT6, LINC01342, TTLL10, KLHL17, AGRN, C1QTNF12, RNF223, MIR200A, MIR200B, SDF4, C1orf159, MIR429, HES4, TNFRSF4, PLEKHN1, PERM1, TNFRSF18, ISG15*
TKHS190002643	NEC Cervix	DUP	chr10:102380506-102398085	*NFKB2, GBF1*
TKHS190002647	NEC Endometrium	DUP	chr10:102472854-102475984	*MFSD13A*
TKHS190002643	NEC Cervix	DEL	chr10:122585271-122593598	*DMBT1*
TKHS190002650	NEC Ovary	DEL	chr10:129536241-129957326	*MIR4297, EBF3, MGMT*
TKHS190002650	NEC Ovary	DEL	chr10:131947530-132445779	*DPYSL4, C10orf91, PWWP2B, JAKMIP3, STK32C, PPP2R2D, BNIP3, LRRC27*
TKHS190002657	NEC Ovary	DUP	chr10:132149982-132445779	*DPYSL4, C10orf91, PWWP2B, JAKMIP3, STK32C, LRRC27*
TKHS190002657	NEC Ovary	DUP	chr10:132908468-133160569	*LINC01168, CFAP46, ADGRA1, KNDC1*
TKHS190002657	NEC Ovary	DUP	chr10:133263156-133312866	*ADAM8, TUBGCP2, PRAP1, ZNF511*
TKHS190002650	NEC Ovary	DEL	chr10:133263156-133352440	*ADAM8, TUBGCP2, PRAP1, ZNF511, CALY*
TKHS190002650	NEC Ovary	DEL	chr10:133398435-133532868	*CYP2E1, SPRN, SCART1, MTG1*
TKHS190002656	NEC Cervix	DUP	chr10:14839905-14934520	*HSPA14, DCLRE1C, SUV39H2*
TKHS190002656	NEC Cervix	DUP	chr10:21538833-21759618	*DNAJC1, MLLT10*
TKHS190002656	NEC Cervix	DUP	chr10:22316417-22389312	*COMMD3-BMI1, COMMD3, BMI1, SPAG6*
TKHS190002656	NEC Cervix	DUP	chr10:240056-356506	*ZMYND11, DIP2C*

TKHS190002656	NEC Cervix	DUP	chr10:26770245-26823305	*ABI1*
TKHS190002656	NEC Cervix	DUP	chr10:27145428-27944962	*YME1L1, RAB18, MKX, PTCHD3, LRRC37A6P, ARMC4, MASTL, ACBD5*
TKHS190002645	NEC Endometrium	DEL	chr10:277325-662962	*MIR5699, DIP2C, PRR26*
TKHS190002656	NEC Cervix	DUP	chr10:32048464-32491997	*NA, KIF5B, EPC1*
TKHS190002656	NEC Cervix	DUP	chr10:35032435-35530036	*CREM, CCNY, CUL2*
TKHS190002650	NEC Ovary	DEL	chr10:44931998-45000859	*DEPP1, C10orf25, TMEM72-AS1, TMEM72, ZNF22, RASSF4*
TKHS190002656	NEC Cervix	DUP	chr10:5456087-5653050	*NET1, CALML5, ASB13, CALML3*
TKHS190002650	NEC Ovary	DEL	chr10:71615504-71682572	*CDH23*
TKHS190002654	NEC Cervix	DUP	chr10:71709098-71812890	*C10orf105, CDH23, VSIR*
TKHS190002641	NEC Ovary	DUP	chr11:116787482-116859604	*APOA5, SIK3, APOA1, APOA4, APOC3, ZPR1*
TKHS190002641	NEC Ovary	DUP	chr11:117175906-117203884	*LOC100652768, PAFAH1B2, SIDT2, TAGLN*
TKHS190002646	NEC Cervix	DUP	chr11:117179264-117192014	*SIDT2*
TKHS190002641	NEC Ovary	DUP	chr11:117504924-117839830	*FXYD6-FXYD2, FXYD2, FXYD6, DSCAML1*
TKHS190002641	NEC Ovary	DUP	chr11:118627305-118661990	*TREH, PHLDB1*
TKHS190002643	NEC Cervix	DUP	chr11:119020150-119111629	*HYOU1, DPAGT1, SLC37A4, H2AX, HMBS, TRAPPC4, VPS11, C2CD2L*
TKHS190002641	NEC Ovary	DUP	chr11:119023321-119048080	*HYOU1, SLC37A4, TRAPPC4*
TKHS190002643	NEC Cervix	DUP	chr11:119359024-119420905	*USP2-AS1, THY1, USP2*
TKHS190002643	NEC Cervix	DUP	chr11:12162156-12255750	*MICAL2*
TKHS190002641	NEC Ovary	DUP	chr11:130188869-130209823	*ST14*
TKHS190002657	NEC Ovary	DEL	chr11:130190454-130209823	*ST14*
TKHS190002654	NEC Cervix	DUP	chr11:17398339-17504697	*USH1C, ABCC8*
TKHS190002643	NEC Cervix	DUP	chr11:45847250-45870532	*CRY2*
TKHS190002642	NEC Ovary	DEL	chr11:487420-644654	*MIR210HG, DEAF1, LRRC56, LOC143666, DRD4, LMNTD2, HRAS, IRF7, MIR210, CDHR5, PHRF1, RNH1, SCT, RASSF7, PTDSS2*
TKHS190002643	NEC Cervix	DUP	chr11:61789795-61848284	*MIR1908, FEN1, FADS1, MIR611, TMEM258, FADS2*
TKHS190002643	NEC Cervix	DUP	chr11:62560069-62609696	*EEF1G, EML3, TUT1, MTA2*
TKHS190002643	NEC Cervix	DUP	chr11:62621259-62715379	*HNRNPUL2-BSCL2, LRRN4CL, HNRNPUL2, GANAB, B3GAT3, BSCL2, GNG3, UBXN1, SNORA57, CSKMT, LBHD1, UQCC3, INTS5*
TKHS190002643	NEC Cervix	DUP	chr11:62789023-62807666	*NXF1, TMEM179B, STX5, TMEM223*
TKHS190002643	NEC Cervix	DUP	chr11:64225499-64243876	*FKBP2, DNAJC4, VEGFB, TRPT1, NUDT22*
TKHS190002644	NEC Cervix	DUP	chr11:65107581-65210872	*FAU, SPDYC, TM7SF2, VPS51, MRPL49, ZNHIT2, CAPN1, SYVN1*
TKHS190002643	NEC Cervix	DUP	chr11:65114713-65133601	*FAU, TM7SF2, MRPL49, ZNHIT2, SYVN1*
TKHS190002644	NEC Cervix	DUP	chr11:65526124-65619932	*KCNK7, ZNRD2, FAM89B, ZNRD2-AS1, EHBP1L1, PCNX3, LTBP3, MAP3K11, SCYL1*
TKHS190002642	NEC Ovary	DEL	chr11:65546798-65580259	*ZNRD2, FAM89B, ZNRD2-AS1, EHBP1L1, LTBP3*
TKHS190002642	NEC Ovary	DEL	chr11:65593343-65628703	*KCNK7, PCNX3, MAP3K11*

TKHS190002643	NEC Cervix	DUP	chr11:6604853-6641613	*TPP1, ILK, TAF10, DCHS1*
TKHS190002643	NEC Cervix	DUP	chr11:66565671-66593714	*CCDC87, CTSF, CCS*
TKHS190002644	NEC Cervix	DUP	chr11:66975363-67279519	*GRK2, KDM2A, C11orf86, RHOD, SYT12*
TKHS190002644	NEC Cervix	DUP	chr11:67431368-67442628	*CORO1B, PTPRCAP, RPS6KB2*
TKHS190002644	NEC Cervix	DUP	chr11:67585138-68019255	*ALDH3B1, ALDH3B2, NUDT8, GSTP1, TBX10, DOC2GP, NDUFV1, FAM86C2P, UNC93B1, ACY3*
TKHS190002641	NEC Ovary	DUP	chr11:695609-726981	*DEAF1, TMEM80, EPS8L2*
TKHS190002643	NEC Cervix	DUP	chr11:72757767-72780450	*STARD10, ARAP1*
TKHS190002642	NEC Ovary	DEL	chr11:774008-842508	*LOC171391, CRACR2B, GATD1, CEND1, POLR2L, PIDD1, PNPLA2, RPLP2, SNORA52, SLC25A22, CD151*
TKHS190002643	NEC Cervix	DUP	chr11:8683202-8917466	*AKIP1, RPL27A, SNORA3A, DENND2B, SNORA3B*
TKHS190002648	NEC Cervix	DUP	chr12:103666291-103677611	*STAB2*
TKHS190002653	NEC Ovary	DUP	chr12:108560765-108852678	*ISCU, CORO1C, TMEM119, SSH1, SELPLG, SART3*
TKHS190002653	NEC Ovary	DUP	chr12:109084953-109279930	*ALKBH2, FOXN4, ACACB, UNG, USP30*
TKHS190002648	NEC Cervix	DUP	chr12:109174132-109281799	*FOXN4, ACACB*
TKHS190002648	NEC Cervix	DUP	chr12:109579802-109855769	*MIR4497, MVK, GLTP, TRPV4, FAM222A, FAM222A-AS1*
TKHS190002653	NEC Ovary	DUP	chr12:109596426-109904147	*MIR4497, MVK, GLTP, TRPV4, TCHP, FAM222A, FAM222A-AS1*
TKHS190002653	NEC Ovary	DUP	chr12:111293446-111448302	*SH2B3, PHETA1, CUX2*
TKHS190002657	NEC Ovary	DEL	chr12:111304210-111363463	*PHETA1, CUX2*
TKHS190002648	NEC Cervix	DUP	chr12:113093539-113398846	*SDS, IQCD, DTX1, PLBD2, CFAP73, TPCN1, DDX54, SLC8B1, RASAL1, RITA1*
TKHS190002657	NEC Ovary	DEL	chr12:113162932-113176935	*DDX54*
TKHS190002643	NEC Cervix	DEL	chr12:113165644-113174936	*DDX54*
TKHS190002653	NEC Ovary	DUP	chr12:113172353-113226964	*IQCD, TPCN1, DDX54, RITA1*
TKHS190002653	NEC Ovary	DUP	chr12:117186477-117531013	*FBXO21, KSR2, NOS1*
TKHS190002648	NEC Cervix	DUP	chr12:120093000-120160023	*MIR4498, GCN1, RAB35, BICDL1*
TKHS190002653	NEC Ovary	DUP	chr12:120097245-120457179	*PXN-AS1, MIR4498, GCN1, RAB35, COX6A1, SIRT4, GATC, MSI1, TRIAP1, PLA2G1B, PXN, RPLP0*
TKHS190002653	NEC Ovary	DUP	chr12:120666875-120737988	*MIR4700, ACADS, UNC119B, CABP1, MLEC*
TKHS190002648	NEC Cervix	DUP	chr12:120687297-120769059	*MIR4700, SPPL3, ACADS, UNC119B, MLEC*
TKHS190002648	NEC Cervix	DUP	chr12:120994164-121005093	*C12orf43, HNF1A*
TKHS190002648	NEC Cervix	DUP	chr12:121221913-121250034	*CAMKK2, P2RX4*
TKHS190002653	NEC Ovary	DUP	chr12:121440816-121899889	*TMEM120B, SETD1B, MORN3, HPD, LINC01089, RHOF, PSMD9, KDM2B, ORAI1*
TKHS190002648	NEC Cervix	DUP	chr12:121775062-121849076	*TMEM120B, SETD1B, HPD, LINC01089, RHOF*
TKHS190002653	NEC Ovary	DUP	chr12:121968006-122207388	*WDR66, MLXIP, LRRC43, IL31, BCL7A, B3GNT4*
TKHS190002648	NEC Cervix	DUP	chr12:122022092-122184779	*MLXIP, LRRC43, IL31, BCL7A*
TKHS190002648	NEC Cervix	DUP	chr12:122848796-122858435	*HIP1R*

TKHS190002653	NEC Ovary	DUP	chr12:122858838-122982586	*ABCB9, ARL6IP4, OGFOD2, VPS37B, HIP1R*
TKHS190002648	NEC Cervix	DUP	chr12:122976253-122986829	*ARL6IP4, PITPNM2, OGFOD2*
TKHS190002657	NEC Ovary	DEL	chr12:124340602-124437996	*NCOR2*
TKHS190002648	NEC Cervix	DUP	chr12:124343005-125125024	*MIR5188, BRI3BP, DHX37, AACS, UBC, SCARB1, NCOR2*
TKHS190002653	NEC Ovary	DUP	chr12:124344597-125125024	*MIR5188, BRI3BP, DHX37, AACS, UBC, SCARB1, NCOR2*
TKHS190002653	NEC Ovary	DUP	chr12:128543957-129337817	*TMEM132D, SLC15A4, GLT1D1, TMEM132C*
TKHS190002657	NEC Ovary	DEL	chr12:131843997-131929796	*MMP17, PUS1, ULK1*
TKHS190002648	NEC Cervix	DUP	chr12:132017535-132675517	*LOC100130238, LRCOL1, P2RX2, DDX51, EP400P1, GALNT9, POLE, EP400, FBRSL1, SNORA49, NOC4L*
TKHS190002653	NEC Ovary	DUP	chr12:132681138-132822128	*PGAM5, ANKLE2, GOLGA3, POLE, PXMP2*
TKHS190002642	NEC Ovary	DUP	chr12:157528-226514	*IQSEC3, SLC6A12, SLC6A13*
TKHS190002642	NEC Ovary	DUP	chr12:2504436-2691199	*CACNA1C-AS1, CACNA1C*
TKHS190002653	NEC Ovary	DUP	chr12:2504436-3696999	*NA, CACNA1C-AS1, TSPAN9, FKBP4, FOXM1, ITFG2-AS1, ITFG2, PRMT8, TEAD4, TULP3, CACNA1C, RHNO1, NRIP2, CRACR2A*
TKHS190002653	NEC Ovary	DUP	chr12:27766665-27969516	*MANSC4, PTHLH, KLHL42*
TKHS190002656	NEC Cervix	DEL	chr12:2820086-2827677	*ITFG2, NRIP2*
TKHS190002642	NEC Ovary	DUP	chr12:3021844-3638454	*TSPAN9, PRMT8, TEAD4, CRACR2A*
TKHS190002653	NEC Ovary	DUP	chr12:47717518-47791706	*RAPGEF3, HDAC7, SLC48A1, ENDOU*
TKHS190002648	NEC Cervix	DUP	chr12:47749390-47855801	*RAPGEF3, HDAC7, SLC48A1, VDR*
TKHS190002656	NEC Cervix	DEL	chr12:48716515-48778290	*MIR4701, ADCY6, TEX49, CCNT1*
TKHS190002648	NEC Cervix	DUP	chr12:48716515-48830692	*MIR4701, ADCY6, TEX49, CACNB3, CCNT1, DDX23*
TKHS190002648	NEC Cervix	DUP	chr12:49024578-49054751	*KMT2D*
TKHS190002653	NEC Ovary	DUP	chr12:49024578-49054751	*KMT2D*
TKHS190002656	NEC Cervix	DEL	chr12:49030893-49054140	*KMT2D*
TKHS190002648	NEC Cervix	DUP	chr12:49069734-49632880	*TROAP, LOC100335030, TUBA1B, MCRS1, RHEBL1, KCNH3, PRPF40B, C1QL4, DHH, LMBR1L, PRPH, SPATS2, TUBA1A, DNAJC22, FAM186B, TUBA1C*
TKHS190002653	NEC Ovary	DUP	chr12:49097672-49351502	*TROAP, TUBA1B, C1QL4, LMBR1L, PRPH, TUBA1A, DNAJC22, TUBA1C*
TKHS190002653	NEC Ovary	DUP	chr12:49543915-49634092	*MCRS1, KCNH3, PRPF40B, FAM186B*
TKHS190002656	NEC Cervix	DEL	chr12:49599469-49666207	*PRPF40B, FAM186B, FMNL3*
TKHS190002648	NEC Cervix	DUP	chr12:51993985-52077190	*GRASP, NR4A1, ATG101, ACVR1B*
TKHS190002653	NEC Ovary	DUP	chr12:51993985-52185587	*KRT80, GRASP, NR4A1, ATG101, ACVR1B*
TKHS190002656	NEC Cervix	DEL	chr12:52013687-52287322	*KRT80, GRASP, C12orf80, LINC00592, NR4A1, KRT7, KRT81, KRT86, ATG101*
TKHS190002656	NEC Cervix	DEL	chr12:53052455-53443827	*SP7, TNS2, AMHR2, LOC283335, ZNF740, IGFBP6, ITGB7, PCBP2, CSAD, PFDN5, PRR13, RARG, C12orf10, SP1, AAAS, SOAT2, SPRYD3, MFSD5, ESPL1*
TKHS190002648	NEC Cervix	DUP	chr12:53097718-53161389	*IGFBP6, CSAD, SOAT2*
TKHS190002648	NEC Cervix	DUP	chr12:53285924-53293474	*ESPL1*

TKHS190002642	NEC Ovary	DUP	chr12:53285924-53293474	*ESPL1*
TKHS190002653	NEC Ovary	DUP	chr12:53320565-53442733	*SP7, AMHR2, PCBP2, PRR13, SP1, AAAS*
TKHS190002653	NEC Ovary	DUP	chr12:53485056-53524761	*LOC100652999, ATF7, TARBP2, MAP3K12, NPFF*
TKHS190002653	NEC Ovary	DUP	chr12:53722025-54011381	*HOTAIR, HOXC13-AS, HOXC8, HOXC9, HOXC10, HOXC11, HOXC12, HOXC13, MIR196A2, CALCOCO1*
TKHS190002653	NEC Ovary	DUP	chr12:54362661-54419198	*ZNF385A, ITGA5, GPR84*
TKHS190002656	NEC Cervix	DEL	chr12:54375844-54408235	*ZNF385A, ITGA5*
TKHS190002642	NEC Ovary	DUP	chr12:55636892-55699989	*OR10P1, METTL7B, ITGA7*
TKHS190002653	NEC Ovary	DUP	chr12:55716933-55749882	*BLOC1S1-RDH5, GDF11, BLOC1S1, RDH5, CD63*
TKHS190002656	NEC Cervix	DEL	chr12:55938915-55958607	*DGKA, PMEL*
TKHS190002653	NEC Ovary	DUP	chr12:55967857-56035331	*CDK2, RAB5B, IKZF4, SUOX*
TKHS190002656	NEC Cervix	DEL	chr12:56116788-56161415	*MYL6B, ESYT1, MYL6, RPL41, ZC3H10*
TKHS190002656	NEC Cervix	DEL	chr12:56224857-56254720	*ANKRD52, SLC39A5, NABP2*
TKHS190002653	NEC Ovary	DUP	chr12:57091125-57192970	*LRP1, NAB2, STAT6*
TKHS190002656	NEC Cervix	DEL	chr12:57091125-57269994	*MIR1228, NXPH4, R3HDM2, STAC3, LRP1, NAB2, NDUFA4L2, SHMT2, STAT6*
TKHS190002648	NEC Cervix	DUP	chr12:57141374-57232843	*MIR1228, NXPH4, LRP1, SHMT2*
TKHS190002657	NEC Ovary	DEL	chr12:57169140-57233662	*MIR1228, NXPH4, LRP1, SHMT2*
TKHS190002653	NEC Ovary	DUP	chr12:57221307-57236681	*NXPH4, NDUFA4L2, SHMT2*
TKHS190002642	NEC Ovary	DUP	chr12:57600324-57827123	*AGAP2-AS1, TSFM, CTDSP2, CDK4, AVIL, OS9, ARHGEF25, AGAP2, CYP27B1, DTX3, B4GALNT1, EEF1AKMT3, MIR26A2, METTL1, TSPAN31, SLC26A10, PIP4K2C, MARCHF9*
TKHS190002648	NEC Cervix	DUP	chr12:5967486-6075551	*VWF*
TKHS190002653	NEC Ovary	DUP	chr12:5967486-6493136	*LTBR, MRPL51, TAPBPL, PLEKHG6, SCNN1A, CD27-AS1, VAMP1, TNFRSF1A, VWF, CD9, CD27*
TKHS190002653	NEC Ovary	DUP	chr12:6526891-6563514	*IFFO1, GAPDH, NOP2, NCAPD2*
TKHS190002653	NEC Ovary	DUP	chr12:6695426-6970543	*LRRC23, P3H3, C12orf57, PHB2, DSTNP2, ATN1, PIANP, ENO2, GPR162, GNB3, RPL13P5, LAG3, MIR141, MIR200C, COPS7A, PTMS, PTPN6, SCARNA12, TPI1, USP5, MLF2, CDCA3, SPSB2, CD4*
TKHS190002656	NEC Cervix	DEL	chr12:6863830-6937561	*LRRC23, DSTNP2, ATN1, ENO2, RPL13P5, TPI1, USP5, SPSB2*
TKHS190002656	NEC Cervix	DEL	chr12:7067789-7150689	*C1RL-AS1, C1RL, C1R, C1S, RBP5, CLSTN3*
TKHS190002653	NEC Ovary	DUP	chr12:7090056-7190569	*C1RL-AS1, C1RL, PEX5, C1R, RBP5, CLSTN3*
TKHS190002653	NEC Ovary	DUP	chr12:99146-237290	*IQSEC3, LOC574538, SLC6A12, SLC6A13*
TKHS190002642	NEC Ovary	DUP	chr13:101057939-101083804	*NALCN-AS1, NALCN*
TKHS190002657	NEC Ovary	DUP	chr14:100123539-100343387	*SLC25A29, DEGS2, SLC25A47, MIR345, EVL, WARS1, YY1*
TKHS190002642	NEC Ovary	DUP	chr14:102039412-102050563	*DYNC1H1*
TKHS190002641	NEC Ovary	DEL	chr14:102039412-102229657	*DYNC1H1, HSP90AA1, MOK, WDR20*
TKHS190002642	NEC Ovary	DUP	chr14:102193463-102229657	*MOK, WDR20*
TKHS190002641	NEC Ovary	DEL	chr14:102939610-102964650	*CDC42BPB*

TKHS190002641	NEC Ovary	DEL	chr14:103502882-103574170	*CKB, TRMT61A, KLC1, MARK3, COA8, BAG5*
TKHS190002641	NEC Ovary	DEL	chr14:104092633-105886061	*ZBTB42, MIR203B, MIR4710, SIVA1, AHNAK2, CLBA1, PLD4, ADSS1, CRIP1, CRIP2, LINC00638, ELK2AP, AKT1, PACS2, NUDT14, KIF26A, CEP170B, TEDC1, BRF1, GPR132, JAG2, ASPG, TMEM179, C14orf180, MIR203A, CDCA4, INF2, TEX22, TMEM121, BTBD6, MTA1*
TKHS190002642	NEC Ovary	DUP	chr14:104739655-104746385	*ADSS1*
TKHS190002642	NEC Ovary	DUP	chr14:104930742-105055420	*AHNAK2, CLBA1, PLD4, GPR132, CDCA4*
TKHS190002647	NEC Endometrium	DUP	chr14:105143482-105173261	*NUDT14, JAG2*
TKHS190002657	NEC Ovary	DUP	chr14:105173021-105217800	*NUDT14, BRF1*
TKHS190002642	NEC Ovary	DUP	*chr14:105838401-105888578*	
TKHS190002657	NEC Ovary	DUP	*chr14:105845375-105886061*	
TKHS190002655	NEC Cervix	DUP	chr14:106088122-106324688	*ELK2AP*
TKHS190002653	NEC Ovary	DEL	chr14:20992092-20995951	*METTL17*
TKHS190002642	NEC Ovary	DUP	chr14:23394068-23433732	*MIR208B, MYH6, MYH7*
TKHS190002657	NEC Ovary	DUP	chr14:24094766-24143489	*FITM1, NRL, EMC9, PCK2, PSME1, PSME2, DCAF11*
TKHS190002657	NEC Ovary	DUP	chr14:24147976-24159960	*RNF31*
TKHS190002657	NEC Ovary	DUP	chr14:24181711-24206723	*TM9SF1, TSSK4, IPO4*
TKHS190002657	NEC Ovary	DUP	chr14:24237245-24255517	*TINF2, GMPR2, TGM1*
TKHS190002655	NEC Cervix	DUP	chr14:24573637-24634160	*CTSG, GZMH, GZMB*
TKHS190002657	NEC Ovary	DUP	chr14:64727593-64742455	*PLEKHG3*
TKHS190002641	NEC Ovary	DEL	chr14:73251664-73265507	*PAPLN*
TKHS190002657	NEC Ovary	DUP	chr14:74494031-74505174	*ISCA2, LTBP2*
TKHS190002657	NEC Ovary	DUP	chr14:74521911-74586118	*LTBP2*
TKHS190002657	NEC Ovary	DUP	chr14:74906549-75009188	*PGF, RPS6KL1, EIF2B2*
TKHS190002653	NEC Ovary	DUP	chr15:29101599-29196564	*FAM189A1, APBA2*
TKHS190002655	NEC Cervix	DUP	chr15:40779545-40812902	*DNAJC17, ZFYVE19*
TKHS190002650	NEC Ovary	DEL	chr15:40779545-40812902	*DNAJC17, ZFYVE19*
TKHS190002647	NEC Endometrium	DUP	chr15:41512115-41513709	*LTK*
TKHS190002650	NEC Ovary	DEL	chr15:42675872-42719574	*STARD9*
TKHS190002655	NEC Cervix	DUP	chr15:42675872-42725251	*CDAN1, STARD9*
TKHS190002655	NEC Cervix	DUP	chr15:43601396-43614476	*STRC*
TKHS190002657	NEC Ovary	DUP	chr15:92444636-92949080	*LINC00930, MIR3175, LINC01578, CHD2, C15orf32, FAM174B, ASB9P1, ST8SIA2*
TKHS190002647	NEC Endometrium	DUP	chr16:1448681-1459187	*CLCN7*
TKHS190002641	NEC Ovary	DEL	chr16:1454411-1494616	*CLCN7, PTX4, TELO2*
TKHS190002641	NEC Ovary	DEL	chr16:15016129-15033399	*PDXDC1*
TKHS190002653	NEC Ovary	DUP	chr16:1520602-1542076	*TMEM204, IFT140*

TKHS190002641	NEC Ovary	DEL	chr16:1523518-1674045	*CRAMP1, TMEM204, IFT140*
TKHS190002655	NEC Cervix	DUP	chr16:16033109-16203613	*ABCC6, ABCC1*
TKHS190002645	NEC Endometrium	DEL	chr16:16090405-16141281	*ABCC1*
TKHS190002641	NEC Ovary	DEL	chr16:1944837-1963839	*SNORA64, NDUFB10, SNORA10, RPL3L, RPS2*
TKHS190002657	NEC Ovary	DUP	chr16:1974181-1981179	*TBL3, NOXO1*
TKHS190002646	NEC Cervix	DEL	chr16:1974181-1986028	*TBL3, NOXO1, GFER*
TKHS190002657	NEC Ovary	DUP	chr16:2037757-2057182	*NTHL1, TSC2, SLC9A3R2*
TKHS190002657	NEC Ovary	DUP	chr16:2082436-2094210	*MIR1225, PKD1, TSC2*
TKHS190002646	NEC Cervix	DEL	chr16:2206086-2235572	*E4F1, BRICD5, PGP, MLST8*
TKHS190002655	NEC Cervix	DUP	chr16:22146236-22308225	*VWA3A, EEF2K, POLR3E*
TKHS190002645	NEC Endometrium	DEL	chr16:22184181-22264880	*EEF2K*
TKHS190002657	NEC Ovary	DUP	chr16:2228292-2235572	*E4F1*
TKHS190002643	NEC Cervix	DEL	chr16:23689238-23694927	*ERN2, PLK1*
TKHS190002646	NEC Cervix	DEL	chr16:2460283-2531073	*NTN3, AMDHD2, ATP6V0C, TBC1D24, CEMP1, TEDC2*
TKHS190002653	NEC Ovary	DUP	chr16:2784631-2786547	*PRSS33*
TKHS190002653	NEC Ovary	DUP	chr16:28974780-28986714	*LAT, SPNS1*
TKHS190002656	NEC Cervix	DEL	chr16:3024327-3026781	*THOC6*
TKHS190002653	NEC Ovary	DUP	chr16:30364900-30379534	*SEPTIN1, ZNF48, TBC1D10B, MYLPF*
TKHS190002653	NEC Ovary	DUP	chr16:30526326-30653103	*ZNF689, ZNF785, ZNF688, ZNF747, PRR14, ZNF768, ZNF764*
TKHS190002650	NEC Ovary	DEL	chr16:3067377-3205185	*OR1F1, ZNF205, ZNF213, ZNF205-AS1, ZSCAN10, IL32*
TKHS190002657	NEC Ovary	DUP	chr16:30756355-30761335	*PHKG2, CCDC189*
TKHS190002655	NEC Cervix	DUP	chr16:31277967-31325404	*ITGAM*
TKHS190002650	NEC Ovary	DEL	chr16:3355941-4007558	*MTRNR2L4, TRAP1, ADCY9, CREBBP, ZNF597, DNASE1, NLRC3, CLUAP1, OR2C1, ZSCAN32, C16orf90, ZNF174, NAA60, SLX4*
TKHS190002657	NEC Ovary	DUP	chr16:3563009-3657953	*DNASE1, NLRC3, SLX4*
TKHS190002655	NEC Cervix	DUP	chr16:3657720-3740549	*TRAP1, CREBBP, DNASE1*
TKHS190002653	NEC Ovary	DUP	chr16:4261779-4395339	*CORO7-PAM16, VASN, PAM16, TFAP4, CORO7, GLIS2*
TKHS190002653	NEC Ovary	DUP	chr16:4651963-4698062	*ANKS3, MGRN1, NUDT16L1*
TKHS190002644	NEC Cervix	DEL	chr16:50292676-50314406	*ADCY7*
TKHS190002641	NEC Ovary	DEL	chr16:50292676-50320030	*ADCY7, BRD7*
TKHS190002653	NEC Ovary	DUP	chr16:50548554-50674074	*SNX20, NKD1*
TKHS190002641	NEC Ovary	DEL	chr16:50621602-50712374	*SNX20, NOD2, NKD1*
TKHS190002641	NEC Ovary	DEL	chr16:55777-93325	*MPG, RHBDF1, SNRNP25, NPRL3*
TKHS190002653	NEC Ovary	DUP	chr16:56519746-56748426	*MT1DP, MT1A, MT1B, MT1E, MT1F, MT1G, MT1H, MT1JP, MT1M, MT1L, MT1X, MT2A, MT3, BBS2, MT1IP, MT4, NUP93*

TKHS190002644	NEC Cervix	DEL	chr16:57680265-57724835	*ADGRG3, DRC7*
TKHS190002644	NEC Cervix	DEL	chr16:58487756-58506473	*NDRG4*
TKHS190002657	NEC Ovary	DUP	chr16:62538-85777	*MPG, RHBDF1, NPRL3*
TKHS190002657	NEC Ovary	DUP	chr16:665950-667782	*WDR90*
TKHS190002642	NEC Ovary	DUP	chr16:66847030-66912580	*CDH16, PDP2, CA7*
TKHS190002642	NEC Ovary	DUP	chr16:66940222-66988830	*CES3, CES4A, CES2*
TKHS190002642	NEC Ovary	DUP	chr16:67228355-67294171	*KCTD19, PLEKHG4, TMEM208, FHOD1, SLC9A5*
TKHS190002641	NEC Ovary	DEL	chr16:67877217-67931852	*NRN1L, CTRL, EDC4, PSKH1, PSMB10*
TKHS190002642	NEC Ovary	DUP	chr16:67877217-67931852	*NRN1L, CTRL, EDC4, PSKH1, PSMB10*
TKHS190002657	NEC Ovary	DUP	chr16:67879587-67930318	*NRN1L, CTRL, EDC4, PSKH1*
TKHS190002646	NEC Cervix	DEL	chr16:681438-697405	*STUB1, FBXL16, JMJD8, WDR24*
TKHS190002650	NEC Ovary	DEL	chr16:681793-717477	*STUB1, FBXL16, JMJD8, METRN, WDR24*
TKHS190002657	NEC Ovary	DUP	chr16:681793-730655	*STUB1, CCDC78, FBXL16, JMJD8, CIAO3, ANTKMT, METRN, WDR24, HAGHL*
TKHS190002641	NEC Ovary	DEL	chr16:69929448-69939940	*WWP2, MIR140*
TKHS190002642	NEC Ovary	DUP	chr16:69929448-69939940	*WWP2, MIR140*
TKHS190002641	NEC Ovary	DEL	chr16:70676881-70698811	*VAC14, MTSS2*
TKHS190002641	NEC Ovary	DEL	chr16:89722531-89739553	*FANCA, ZNF276*
TKHS190002641	NEC Ovary	DEL	chr16:89895990-89932679	*TUBB3, TCF25, MC1R*
TKHS190002657	NEC Ovary	DUP	chr17:1056035-1058867	*ABR*
TKHS190002642	NEC Ovary	DEL	chr17:1478087-1492487	*MYO1C*
TKHS190002643	NEC Cervix	DUP	chr17:1771029-1801024	*SMYD4, SERPINF1*
TKHS190002643	NEC Cervix	DUP	chr17:19341238-19362721	*MIR1180, B9D1*
TKHS190002654	NEC Cervix	DUP	chr17:19384462-19416175	*MFAP4, RNF112*
TKHS190002654	NEC Cervix	DUP	chr17:21287991-21304553	*MAP2K3*
TKHS190002642	NEC Ovary	DEL	chr17:2692571-2704564	*CLUH*
TKHS190002650	NEC Ovary	DEL	chr17:28554220-28575532	*ALDOC, PIGS*
TKHS190002643	NEC Cervix	DUP	chr17:28710871-28741987	*NA, TLCD1, PROCA1, SNORD4B, SNORD4A, SNORD42B, SNORD42A, NEK8, RPL23A, RAB34*
TKHS190002644	NEC Cervix	DUP	chr17:28735240-28749577	*NEK8, TRAF4*
TKHS190002655	NEC Cervix	DUP	chr17:35983801-36090058	*CCL15-CCL14, CCL3, CCL14, CCL15, CCL18, CCL23*
TKHS190002650	NEC Ovary	DEL	chr17:3953347-4034292	*ZZEF1, ATP2A3*
TKHS190002657	NEC Ovary	DUP	chr17:40021955-40031620	*SNORD124, MED24*
TKHS190002643	NEC Cervix	DUP	chr17:40035722-40097403	*THRA, NR1D1, MED24*
TKHS190002647	NEC Endometrium	DEL	chr17:42552934-42565047	*HSD17B1, COASY*

TKHS190002641	NEC Ovary	DUP	chr17:4718612-4796296	*VMO1, GLTPD2, MED11, ARRB2, PSMB6, CXCL16, ZMYND15, TM4SF5*
TKHS190002650	NEC Ovary	DEL	chr17:4720393-4887790	*VMO1, GLTPD2, MED11, ARRB2, MINK1, PLD2, PSMB6, CXCL16, ZMYND15, TM4SF5*
TKHS190002641	NEC Ovary	DUP	chr17:4893286-4902494	*C17orf107, CHRNE, MINK1*
TKHS190002650	NEC Ovary	DEL	chr17:4902217-4981831	*C17orf107, CHRNE, ENO3, CAMTA2, RNF167, GP1BA, PFN1, SLC25A11, SPAG7*
TKHS190002643	NEC Cervix	DUP	chr17:4938154-4946844	*RNF167, PFN1, SLC25A11*
TKHS190002657	NEC Ovary	DUP	chr17:4952025-4960547	*ENO3, SPAG7*
TKHS190002643	NEC Cervix	DUP	chr17:4960022-4981359	*CAMTA2, SPAG7*
TKHS190002643	NEC Cervix	DUP	chr17:50186317-50199590	*COL1A1*
TKHS190002644	NEC Cervix	DUP	chr17:62666757-62681937	*MRC2*
TKHS190002644	NEC Cervix	DUP	chr17:63434402-63479912	*CYB561, ACE*
TKHS190002644	NEC Cervix	DUP	chr17:63824637-63838681	*FTSJ3, PSMC5, SMARCD2*
TKHS190002644	NEC Cervix	DUP	chr17:64001733-64160933	*ERN1, ICAM2, TEX2, SNORA50C, SNORD104, PRR29*
TKHS190002643	NEC Cervix	DUP	chr17:7013666-7017014	*MIR497HG, RNASEK-C17orf49*
TKHS190002650	NEC Ovary	DEL	chr17:7015062-7076152	*MIR497HG, RNASEK-C17orf49, CLEC10A, SLC16A11, SLC16A13, BCL6B, MIR497*
TKHS190002641	NEC Ovary	DUP	chr17:7015774-7043585	*MIR497HG, RNASEK-C17orf49, SLC16A11, SLC16A13, BCL6B, MIR497*
TKHS190002655	NEC Cervix	DUP	chr17:7075360-7076987	*CLEC10A*
TKHS190002642	NEC Ovary	DEL	chr17:7173659-7203593	*DLG4, ASGR1*
TKHS190002641	NEC Ovary	DUP	chr17:7173659-7236767	*DLG4, DVL2, ACADVL, ASGR1, MIR324, PHF23*
TKHS190002654	NEC Cervix	DUP	chr17:7193483-7194495	*DLG4*
TKHS190002650	NEC Ovary	DEL	chr17:7193483-7236767	*DLG4, DVL2, ACADVL, MIR324, PHF23*
TKHS190002643	NEC Cervix	DUP	chr17:7220464-7224552	*ACADVL, MIR324*
TKHS190002643	NEC Cervix	DUP	chr17:7229378-7244635	*GABARAP, DVL2, CTDNEP1, PHF23*
TKHS190002641	NEC Ovary	DUP	chr17:7252918-7437506	*TMEM256-PLSCR3, CLDN7, KCTD11, EIF5A, SPEM2, ELP5, TMEM256, TMEM102, GPS2, TMEM95, SPEM1, YBX2, PLSCR3, NLGN2, SLC2A4, NEURL4, TNK1, ACAP1*
TKHS190002643	NEC Cervix	DUP	chr17:7291093-7314597	*EIF5A, GPS2, YBX2, NEURL4*
TKHS190002641	NEC Ovary	DUP	chr17:7455794-7564864	*SENP3-EIF4A1, CHRNB1, SENP3, TNFSF12-TNFSF13, POLR2A, ZBTB4, SLC35G6, TNFSF13, TNFSF12*
TKHS190002641	NEC Ovary	DUP	chr17:74729256-74771858	*MIR3615, NAT9, RAB37, SLC9A3R1*
TKHS190002644	NEC Cervix	DUP	chr17:74744308-74788870	*MIR3615, NAT9, RAB37, TMEM104, SLC9A3R1*
TKHS190002650	NEC Ovary	DEL	chr17:7481988-7572864	*SENP3-EIF4A1, SENP3, TNFSF12-TNFSF13, POLR2A, ZBTB4, SLC35G6, TNFSF13, TNFSF12*
TKHS190002641	NEC Ovary	DUP	chr17:74872804-74964632	*USH1G, FDXR, FADS6, HID1, OTOP3, OTOP2*
TKHS190002644	NEC Cervix	DUP	chr17:74954138-75021079	*HID1, CDR2L, MRPL58*
TKHS190002644	NEC Cervix	DUP	chr17:75238914-75614360	*LOC100287042, MIR3678, GGA3, TSEN54, GRB2, LLGL2, MRPS7, MIF4GD, CASKIN2, SLC25A19, MYO15B, TMEM94*
TKHS190002641	NEC Ovary	DUP	chr17:75485428-75651265	*SMIM6, TSEN54, LLGL2, CASKIN2, SMIM5, MYO15B, RECQL5, TMEM94*
TKHS190002653	NEC Ovary	DEL	chr17:75491691-75504693	*CASKIN2, TMEM94*

TKHS190002643	NEC Cervix	DUP	chr17:7550120-7587709	*SENP3-EIF4A1, SENP3, SNORA67, TNFSF12-TNFSF13, SNORA48, SNORD10, TNFSF13, TNFSF12, MPDU1, CD68*
TKHS190002644	NEC Cervix	DUP	chr17:75671816-75816912	*MIR4738, GALK1, SAP30BP, H3-3B, ITGB4, UNK*
TKHS190002654	NEC Cervix	DEL	chr17:75706008-75752356	*SAP30BP, ITGB4*
TKHS190002641	NEC Ovary	DUP	chr17:75752174-75828983	*MIR4738, UNC13D, GALK1, H3-3B, ITGB4, UNK*
TKHS190002641	NEC Ovary	DUP	chr17:75874208-75915139	*TRIM65, MRPL38, FBF1, TRIM47*
TKHS190002644	NEC Cervix	DUP	chr17:75949495-76002612	*CDK3, ACOX1*
TKHS190002644	NEC Cervix	DUP	chr17:76079439-76280953	*RNF157-AS1, RNF157, FOXJ1, EXOC7, UBALD2, ZACN, QRICH2*
TKHS190002641	NEC Ovary	DUP	chr17:76097796-76162002	*RNF157-AS1, RNF157, FOXJ1, EXOC7*
TKHS190002641	NEC Ovary	DUP	chr17:76468633-76540215	*CYGB, AANAT, PRCD, RHBDF2*
TKHS190002650	NEC Ovary	DEL	chr17:7661939-7703486	*WRAP53, TP53*
TKHS190002644	NEC Cervix	DUP	chr17:76625819-76643638	*ST6GALNAC1*
TKHS190002644	NEC Cervix	DUP	chr17:78122605-78205753	*C17orf99, TMC6, AFMID, TMC8, TK1, SYNGR2*
TKHS190002641	NEC Ovary	DUP	chr17:7831390-7858824	*CYB5D1, DNAH2, KDM6B, NAA38, TMEM88*
TKHS190002642	NEC Ovary	DEL	chr17:7845555-7855714	*KDM6B, TMEM88*
TKHS190002643	NEC Cervix	DUP	chr17:7930606-7934222	*CNTROB, TRAPPC1*
TKHS190002644	NEC Cervix	DUP	chr17:80140008-80201870	*CARD14, EIF4A3*
TKHS190002644	NEC Cervix	DUP	chr17:80251329-80415821	*RNF213-AS1, SLC26A11, ENDOV, RNF213*
TKHS190002641	NEC Ovary	DUP	chr17:8073657-8150706	*MIR4314, ALOX12B, PER1, ALOXE3, HES7*
TKHS190002644	NEC Cervix	DUP	chr17:81053668-81119580	*BAIAP2, AATK*
TKHS190002641	NEC Ovary	DUP	chr17:81379722-81548028	*LOC100130370, MIR3186, MIR4740, FSCN2, BAHCC1, ACTG1, FAAP100*
TKHS190002650	NEC Ovary	DEL	chr17:8143266-8255691	*NA, MIR4521, LINC00324, PER1, PFAS, BORCS6, VAMP2, CTC1, TMEM107, AURKB*
TKHS190002641	NEC Ovary	DUP	*chr17:81688704-81833852*	
TKHS190002644	NEC Cervix	DUP	chr17:81824499-81941741	*ALYREF, PPP1R27, MYADML2, NPB, MCRIP1, ARHGDIA, MAFG, P4HB, ANAPC11, SIRT7, PYCR1, PCYT2, MAFG-DT*
TKHS190002644	NEC Cervix	DUP	chr17:82017309-82027011	*CENPX, LRRC45, ASPSCR1*
TKHS190002641	NEC Ovary	DUP	chr17:82030065-82091684	*LRRC45, FASN, GPS1, DCXR, RAC3, RFNG, DUS1L*
TKHS190002644	NEC Cervix	DUP	chr17:82033699-82053366	*GPS1, DCXR, RAC3, RFNG*
TKHS190002653	NEC Ovary	DEL	chr17:82081164-82085871	*FASN*
TKHS190002646	NEC Cervix	DUP	chr17:82082009-82084716	*FASN*
TKHS190002641	NEC Ovary	DUP	chr17:8254166-8319394	*ARHGEF15, RANGRF, SLC25A35, PFAS*
TKHS190002644	NEC Cervix	DUP	chr17:82741311-82907821	*FN3K, TBCD, ZNF750*
TKHS190002645	NEC Endometrium	DEL	chr18:12254384-14106409	*LDLRAD4-AS1, MIR4526, MIR5190, PRELID3A, AFG3L2, CIDEA, FAM210A, ZNF519, MC2R, MC5R, CEP192, SPIRE1, PSMG2, PTPN2, LDLRAD4, CEP76, SEH1L, TUBB6, RNMT*
TKHS190002646	NEC Cervix	DUP	chr18:158699-14837289	*LINC01387, LOC100192426, LDLRAD4-AS1, MIR4317, MIR3156-2, MIR4526, MIR3976, MIR5190, DLGAP1-AS4, MYL12B, NDC80, MYL12A, PRELID3A, CETN1, RALBP1, AFG3L2, RAB31, CIDEA, ADCYAP1, FAM210A, APCDD1, LINC00526, ZNF519, RAB12, DLGAP1-AS3, EPB41L3, ANKRD12, MTCL1, SMCHD1, CLUL1, GNAL, DLGAP1-AS5, LAMA1, CYP4F35P, LINC00667,*

				LRRC30, ANKRD62, IMPA2, ANKRD30B, POTEC, LINC00668, MC2R, MC5R, CXADRP3, ANKRD20A5P, NDUFV2, TYMSOS, C18orf61, CEP192, ENOSF1, LINC00470, SPIRE1, PSMG2, TWSG1, CHMP1B, PTPN2, PTPRM, PIEZO2, AKAIN1, CBX3P2, MIR3976HG, TMEM200C, METTL4, DLGAP1-AS1, MPPE1, TGIF1, LOC727896, TYMS, YES1, LDLRAD4, ZBTB14, ARHGAP28, CEP76, COLEC12, SEH1L, EMILIN2, TXNDC2, TUBB6, RNMT, MYOM1, NAPG, USP14, L3MBTL4, VAPA, DLGAP1, LPIN2, THOC1, PPP4R1
TKHS190002646	NEC Cervix	DUP	chr18:21395385-21518234	*GREB1L*
TKHS190002646	NEC Cervix	DUP	chr18:21659170-21857243	*MIR320C1, ABHD3, MIR1-2, MIR133A1, MIB1*
TKHS190002646	NEC Cervix	DUP	chr18:22993721-23753812	*ANKRD29, RMC1, LAMA3, NPC1, RBBP8, TMEM241, RIOK3, CABLES1*
TKHS190002646	NEC Cervix	DUP	chr18:23939223-24179835	*OSBPL1A, TTC39C, CABYR, LAMA3*
TKHS190002645	NEC Endometrium	DEL	chr18:2707563-3215223	*SMCHD1, LOC727896, EMILIN2, MYOM1, LPIN2*
TKHS190002657	NEC Ovary	DEL	chr18:3100320-3176134	*MYOM1*
TKHS190002646	NEC Cervix	DUP	chr18:31074683-31595276	*DSG2-AS1, DSG4, DSC1, DSC2, DSG1, DSG2, DSG3, TTR*
TKHS190002646	NEC Cervix	DUP	chr18:31917578-32393035	*TRAPPC8, MEP1B, RNF138, RNF125, GAREM1*
TKHS190002650	NEC Ovary	DUP	chr18:33245245-34877808	*DTNA, CCDC178, ASXL3, NOL4*
TKHS190002645	NEC Endometrium	DEL	chr18:346295-732965	*CETN1, CLUL1, TYMSOS, ENOSF1, TYMS, YES1, COLEC12*
TKHS190002650	NEC Ovary	DUP	chr18:36501932-36780188	*TPGS2, FHOD3*
TKHS190002645	NEC Endometrium	DUP	chr18:37160200-37321881	*CELF4, KIAA1328*
TKHS190002650	NEC Ovary	DUP	chr18:45912290-45939755	*EPG5*
TKHS190002650	NEC Ovary	DUP	chr18:46456519-46855435	*LOXHD1, ST8SIA5, RNF165, PIAS2*
TKHS190002650	NEC Ovary	DUP	chr18:47059556-48921910	*MIR4743, ZBTB7C, SMAD2, SMAD7, IER3IP1, SKOR2, KATNAL2, HDHD2, CTIF*
TKHS190002650	NEC Ovary	DUP	chr18:50270011-50819702	*SKA1, CXXC1, MBD1, MAPK4, MRO*
TKHS190002645	NEC Endometrium	DEL	chr18:5290858-5433556	*EPB41L3, ZBTB14*
TKHS190002650	NEC Ovary	DUP	chr18:56781533-57476723	*LINC-ROR, WDR7, BOD1L2, ST8SIA3, ONECUT2*
TKHS190002650	NEC Ovary	DUP	chr18:62231194-62575550	*ZCCHC2, RELCH, TNFRSF11A*
TKHS190002645	NEC Endometrium	DEL	chr18:6851034-10801428	*LOC100192426, RALBP1, RAB31, APCDD1, RAB12, ANKRD12, MTCL1, LAMA1, LRRC30, LINC00668, NDUFV2, TWSG1, PTPRM, PIEZO2, ARHGAP28, TXNDC2, NAPG, VAPA, PPP4R1*
TKHS190002657	NEC Ovary	DEL	chr18:6942079-7012138	*LAMA1*
TKHS190002650	NEC Ovary	DUP	chr18:70057742-70642723	*RTTN, SOCS6*
TKHS190002650	NEC Ovary	DUP	chr18:76923071-77268902	*GALR1, MBP, ZNF236*
TKHS190002653	NEC Ovary	DEL	chr18:80034496-80133192	*RBFADN, ADNP2, RBFA*
TKHS190002657	NEC Ovary	DUP	chr19:10093349-10133701	*SNORD105B, DNMT1, P2RY11, PPAN, SNORD105, PPAN-P2RY11, ANGPTL6, EIF3G*
TKHS190002657	NEC Ovary	DUP	chr19:1037625-1043473	*ABCA7, CNN2*
TKHS190002647	NEC Endometrium	DUP	chr19:10459400-10467621	*PDE4A*
TKHS190002657	NEC Ovary	DUP	chr19:1063760-1086006	*ABCA7, ARHGAP45*
TKHS190002641	NEC Ovary	DEL	chr19:10858316-10928410	*CARM1, C19orf38, YIPF2*
TKHS190002657	NEC Ovary	DUP	chr19:10858316-10929702	*CARM1, C19orf38, YIPF2, TIMM29*

TKHS190002646	NEC Cervix	DEL	chr19:10908039-10928410	*CARM1, YIPF2*
TKHS190002657	NEC Ovary	DUP	chr19:11147134-11202483	*SPC24, KANK2, DOCK6*
TKHS190002643	NEC Cervix	DUP	chr19:11166558-11202483	*KANK2, DOCK6*
TKHS190002657	NEC Ovary	DUP	chr19:1256995-1440462	*CIRBP, CIRBP-AS1, EFNA2, GAMT, DAZAP1, NDUFS7, FAM174C, RPS15, PWWP3A, MIDN*
TKHS190002647	NEC Endometrium	DUP	chr19:12701752-12715739	*SNORD41, TNPO2*
TKHS190002657	NEC Ovary	DUP	chr19:12706041-12847687	*RNASEH2A, MAST1, SNORD41, HOOK2, TNPO2, JUNB, GET3, BEST2, PRDX2, TRIR, RTBDN*
TKHS190002643	NEC Cervix	DUP	chr19:12772780-12810208	*RNASEH2A, HOOK2, JUNB, PRDX2*
TKHS190002657	NEC Ovary	DUP	chr19:12880972-13100748	*KLF1, DNASE2, DAND5, FARSA, SYCE2, GCDH, LYL1, NFIX, RAD23A, CALR, GADD45GIP1*
TKHS190002646	NEC Cervix	DEL	chr19:12947848-13090390	*DAND5, NFIX, RAD23A, GADD45GIP1*
TKHS190002657	NEC Ovary	DUP	chr19:13758868-13904913	*LOC284454, C19orf53, NANOS3, MIR181C, MIR181D, ZSWIM4, C19orf57, CCDC130, MRI1*
TKHS190002647	NEC Endometrium	DUP	chr19:13809070-13831050	*ZSWIM4*
TKHS190002647	NEC Endometrium	DUP	chr19:13919130-13930289	*CC2D1A*
TKHS190002657	NEC Ovary	DUP	chr19:13979447-14093237	*MISP3, RLN3, PALM3, PRKACA, RFX1, C19orf67, SAMD1, IL27RA*
TKHS190002657	NEC Ovary	DUP	chr19:14402597-14446561	*DDX39A, PKN1, ADGRE5*
TKHS190002647	NEC Endometrium	DUP	chr19:14952928-15111439	*OR1I1, CCDC105, CASP14, SLC1A6, SYDE1*
TKHS190002647	NEC Endometrium	DUP	chr19:15170331-15192519	*NOTCH3*
TKHS190002657	NEC Ovary	DUP	chr19:15452045-15458655	*RASAL3*
TKHS190002657	NEC Ovary	DUP	chr19:17283225-17333800	*ANKLE1, ANO8, MRPL34, DDA1, ABHD8*
TKHS190002644	NEC Cervix	DUP	chr19:17305831-17333800	*ANO8, MRPL34, DDA1*
TKHS190002647	NEC Endometrium	DUP	chr19:1784819-1791861	*ATP8B3*
TKHS190002657	NEC Ovary	DUP	chr19:18143763-18532814	*MIR3188, MIR3189, IFI30, LRRC25, SSBP4, MAST3, FKBP8, LSM4, JUND, PDE4C, ISYNA1, PIK3R2, PGPEP1, RAB3A, LOC729966, IQCN, ELL, MPV17L2, GDF15*
TKHS190002641	NEC Ovary	DEL	chr19:18193282-18220515	*PDE4C, RAB3A, MPV17L2*
TKHS190002646	NEC Cervix	DEL	chr19:18432973-18465575	*SSBP4, ISYNA1, ELL*
TKHS190002657	NEC Ovary	DUP	chr19:18667577-18788319	*COMP, CRTC1, KLHL26*
TKHS190002657	NEC Ovary	DUP	chr19:18832210-18920909	*CERS1, COPE, GDF1, DDX49, UPF1, HOMER3*
TKHS190002647	NEC Endometrium	DUP	chr19:19120765-19133306	*TMEM161A*
TKHS190002647	NEC Endometrium	DUP	chr19:1918127-1987692	*SCAMP4, CSNK1G2, CSNK1G2-AS1, BTBD2*
TKHS190002647	NEC Endometrium	DUP	chr19:19260813-19268754	*HAPLN4, TM6SF2*
TKHS190002657	NEC Ovary	DUP	chr19:2039613-2426088	*MIR1227, MIR4321, IZUMO4, JSRP1, MOB3A, TIMM13, AMH, MKNK2, TMPRSS9, PEAK3, OAZ1, LSM7, PLEKHJ1, SPPL2B, LINGO3, SF3A2, DOT1L, AP3D1*
TKHS190002650	NEC Ovary	DEL	chr19:2132471-2247022	*MIR1227, PLEKHJ1, SF3A2, DOT1L, AP3D1*
TKHS190002647	NEC Endometrium	DUP	chr19:291285-327989	*MIER2, PLPP2*
TKHS190002654	NEC Cervix	DUP	chr19:2980100-2991984	*TLE6*

TKHS190002657	NEC Ovary	DUP	chr19:3055664-3062927	*TLE5*
TKHS190002647	NEC Endometrium	DUP	chr19:3435083-3539260	*C19orf71, MFSD12, SMIM24, NFIC, FZR1, DOHH*
TKHS190002657	NEC Ovary	DUP	chr19:35262546-35295981	*MAG, LSR, HAMP, USF2*
TKHS190002657	NEC Ovary	DUP	chr19:35449715-35547447	*TMEM147-AS1, TMEM147, GAPDHS, FFAR2, SBSN, KRTDAP, DMKN*
TKHS190002657	NEC Ovary	DUP	chr19:35738282-35752488	*U2AF1L4, PSENEN, LIN37, IGFLR1, KMT2B*
TKHS190002643	NEC Cervix	DUP	chr19:35743809-35759991	*HSPB6, PROSER3, U2AF1L4, PSENEN, LIN37, IGFLR1*
TKHS190002647	NEC Endometrium	DUP	chr19:36016158-36035592	*THAP8, CLIP3*
TKHS190002647	NEC Endometrium	DUP	chr19:3637410-3653589	*PIP5K1C*
TKHS190002657	NEC Ovary	DUP	chr19:3643243-3738663	*PIP5K1C, TJP3*
TKHS190002657	NEC Ovary	DUP	chr19:38616195-38734389	*MAP4K1, CAPN12, EIF3K, ACTN4*
TKHS190002657	NEC Ovary	DUP	chr19:38869943-38907707	*RINL, SIRT2, NFKBIB*
TKHS190002657	NEC Ovary	DUP	chr19:38917724-38922078	*SARS2*
TKHS190002657	NEC Ovary	DUP	chr19:39388339-39392522	*PAF1, MED29*
TKHS190002643	NEC Cervix	DUP	chr19:39388560-39397757	*PAF1, MED29*
TKHS190002645	NEC Endometrium	DUP	chr19:42729123-43194594	*PSG8-AS1, PSG8, PSG1, PSG2, PSG3, PSG4, PSG5, PSG6, PSG7, PSG11, PSG10P*
TKHS190002657	NEC Ovary	DUP	chr19:4408903-4448415	*CHAF1A, MIR4746, UBXN6*
TKHS190002647	NEC Endometrium	DUP	chr19:4428664-4445623	*CHAF1A, UBXN6*
TKHS190002657	NEC Ovary	DUP	chr19:4499491-5040011	*DPP9-AS1, MIR4747, PLIN3, SEMA6B, LRG1, TNFAIP8L1, TICAM1, KDM4B, MIR7-3HG, UHRF1, MIR7-3, PLIN5, FEM1A, MYDGF, ARRDC5, PLIN4, HDGFL2, DPP9*
TKHS190002657	NEC Ovary	DUP	chr19:45406697-45500710	*CD3EAP, PPM1N, ERCC1, FOSB, RTN2*
TKHS190002657	NEC Ovary	DUP	chr19:45759414-46040416	*SIX5, DMPK, DMWD, IRF2BP1, FOXA3, MYPOP, NANOS2, BHMG1, IGFL4, NOVA2, CCDC61, MIR769, RSPH6A, SYMPK, PGLYRP1*
TKHS190002647	NEC Endometrium	DUP	chr19:4651870-4685771	*DPP9-AS1, TNFAIP8L1, MYDGF, DPP9*
TKHS190002641	NEC Ovary	DEL	chr19:4658005-4684862	*DPP9-AS1, MYDGF, DPP9*
TKHS190002653	NEC Ovary	DUP	chr19:474621-519423	*ODF3L2, MADCAM1, TPGS1*
TKHS190002657	NEC Ovary	DUP	chr19:47480958-47514940	*NAPA-AS1, KPTN, NAPA*
TKHS190002657	NEC Ovary	DUP	chr19:48311553-48363531	*EMP3, TMEM143, CCDC114*
TKHS190002657	NEC Ovary	DUP	chr19:48702957-48806717	*FUT1, FUT2, FGF21, MAMSTR, IZUMO1, RASIP1, BCAT2*
TKHS190002641	NEC Ovary	DEL	chr19:4891046-5215412	*MIR4747, KDM4B, UHRF1, PTPRS, ARRDC5*
TKHS190002657	NEC Ovary	DUP	chr19:48965508-48993112	*FTL, GYS1*
TKHS190002657	NEC Ovary	DUP	chr19:49070063-49166215	*HRC, KCNA7, TRPM4, C19orf73, LIN7B, SNRNP70, PPFIA3*
TKHS190002647	NEC Endometrium	DUP	chr19:49171578-49189091	*TRPM4*
TKHS190002657	NEC Ovary	DUP	chr19:49374717-49450939	*GFY, PTH2, CCDC155, DKKL1, PIH1D1, SLC17A7*
TKHS190002657	NEC Ovary	DUP	chr19:49513882-49685037	*MIR5088, FCGRT, PRMT1, IRF3, NOSIP, PRRG2, RCN3, PRR12, SCAF1, RRAS, BCL2L12*
TKHS190002657	NEC Ovary	DUP	chr19:49801390-49836962	*AP2A1, FUZ, MED25*

TKHS190002647	NEC Endometrium	DUP	chr19:49932422-49960935	*MIR4751, SIGLEC11, ATF5*
TKHS190002647	NEC Endometrium	DUP	chr19:50399371-50407415	*POLD1*
TKHS190002657	NEC Ovary	DUP	chr19:50451280-50519415	*FAM71E1, JOSD2, EMC10, MYBPC2, ASPDH, LRRC4B*
TKHS190002657	NEC Ovary	DUP	chr19:50711371-50860127	*LINC01869, GPR32, KLK3, KLK1, SHANK1, KLK15, CLEC11A, SNORD88A, SNORD88B, SNORD88C, C19orf48, ACP4*
TKHS190002657	NEC Ovary	DUP	chr19:51022545-51060093	*KLK11, KLK13, KLK12*
TKHS190002647	NEC Endometrium	DUP	chr19:51643779-51719928	*SIGLEC14, SPACA6, HAS1, MIRLET7E, MIR125A, MIR99B*
TKHS190002657	NEC Ovary	DUP	chr19:55132915-55174999	*DNAAF3, SYT5, TNNI3, TNNT1*
TKHS190002647	NEC Endometrium	DUP	chr19:55236913-55274622	*PPP6R1, HSPBP1*
TKHS190002657	NEC Ovary	DUP	chr19:55437605-55689950	*SBK3, ZNF865, U2AF2, ZNF524, ZNF784, ZNF579, SSC5D, CCDC106, EPN1, ZNF580, ZNF581, NAT14, SBK2, SHISA7, ISOC2, FIZ1, ZNF628*
TKHS190002657	NEC Ovary	DUP	chr19:5654061-5707143	*MICOS13, RPL36, HSD11B1L, SAFB, LONP1*
TKHS190002647	NEC Endometrium	DUP	chr19:5700789-5708403	*LONP1*
TKHS190002657	NEC Ovary	DUP	chr19:5776161-5791141	*PRR22, CATSPERD, DUS3L*
TKHS190002643	NEC Cervix	DUP	chr19:58545425-58547906	*TRIM28*
TKHS190002647	NEC Endometrium	DUP	chr19:6002721-6147675	*LOC100128568, RFX2, ACSBG2*
TKHS190002657	NEC Ovary	DUP	chr19:7468825-7507141	*NA, ARHGEF18, TEX45, PEX11G*
TKHS190002647	NEC Endometrium	DUP	chr19:7539918-7551107	*PNPLA6*
TKHS190002657	NEC Ovary	DUP	chr19:7846543-7981186	*TGFBR3L, TIMM44, EVI5L, ELAVL1, LYPLA2P2, CTXN1, MAP2K7, SNAPC2, LRRC8E, PRR36*
TKHS190002657	NEC Ovary	DUP	chr19:8131554-8251249	*CERS4, FBN3*
TKHS190002647	NEC Endometrium	DUP	chr19:814434-843626	*PRTN3, AZU1, PLPPR3*
TKHS190002657	NEC Ovary	DUP	chr19:8303855-8325096	*KANK3, NDUFA7, CD320, RPS28*
TKHS190002657	NEC Ovary	DUP	chr19:860861-877180	*MED16, CFD*
TKHS190002647	NEC Endometrium	DUP	chr19:8895668-8981138	*MUC16*
TKHS190002641	NEC Ovary	DUP	chr2:176002160-176190142	*HOXD-AS2, HOXD1, HOXD3, HOXD4, HOXD8, HOXD9, HOXD10, HOXD11, HOXD12, HOXD13, EVX2, HAGLR, MIR10B, LNPK*
TKHS190002641	NEC Ovary	DUP	chr2:218356885-218436626	*CATIP, MIR26B, CTDSP1, SLC11A1, VIL1*
TKHS190002657	NEC Ovary	DEL	chr2:218386635-218431042	*MIR26B, CTDSP1, SLC11A1, VIL1*
TKHS190002646	NEC Cervix	DUP	chr2:218404297-218435434	*CTDSP1, VIL1*
TKHS190002654	NEC Cervix	DUP	chr2:218882161-219010085	*LOC100129175, CRYBA2, LINC00608, CFAP65, MIR375, FEV, WNT10A, CDK5R2*
TKHS190002654	NEC Cervix	DUP	chr2:219169823-219230405	*ABCB6, ZFAND2B, SLC23A3, CNPPD1, ANKZF1, ATG9A, RETREG2, NHEJ1*
TKHS190002643	NEC Cervix	DUP	chr2:219210230-219233433	*ABCB6, ANKZF1, ATG9A*
TKHS190002641	NEC Ovary	DUP	chr2:219246000-219309332	*DNAJB2, MIR153-1, PTPRN, TUBA4A, TUBA4B, STK16*
TKHS190002654	NEC Cervix	DUP	chr2:219251565-219451807	*SPEG, DES, DNPEP, DNAJB2, RESP18, MIR153-1, PTPRN, TUBA4A, TUBA4B*
TKHS190002657	NEC Ovary	DEL	chr2:219284632-219298103	*DNAJB2, MIR153-1, PTPRN*
TKHS190002657	NEC Ovary	DEL	chr2:219441558-219641728	*MIR3132, SPEG, STK11IP, TMEM198, OBSL1, GMPPA, INHA, ASIC4, SLC4A3, CHPF*

TKHS190002654	NEC Cervix	DUP	chr2:219479783-219568324	*MIR3132, SPEG, TMEM198, OBSL1, GMPPA, ASIC4, CHPF*
TKHS190002644	NEC Cervix	DEL	chr2:219556454-219568324	*OBSL1*
TKHS190002657	NEC Ovary	DEL	chr2:232456200-232524844	*ALPI, PRSS56, ECEL1*
TKHS190002644	NEC Cervix	DEL	chr2:233457221-233462735	*DGKD*
TKHS190002654	NEC Cervix	DUP	*chr2:233789497-233831540*	
TKHS190002653	NEC Ovary	DUP	chr2:237324774-237381499	*COL6A3*
TKHS190002654	NEC Cervix	DUP	chr2:238238827-238260953	*HES6, PER2*
TKHS190002654	NEC Cervix	DUP	chr2:240572423-241033875	*CAPN10, AGXT, LOC200772, SNED1, GPR35, AQP12A, KIF1A, RNPEPL1, AQP12B, MAB21L4*
TKHS190002644	NEC Cervix	DEL	chr2:240723975-240785100	*KIF1A*
TKHS190002654	NEC Cervix	DUP	chr2:241491061-241508035	*STK25, FARP2*
TKHS190002644	NEC Cervix	DUP	chr2:24820724-24830825	*ADCY3, CENPO*
TKHS190002644	NEC Cervix	DUP	chr2:26476891-26503845	*OTOF*
TKHS190002643	NEC Cervix	DUP	chr2:27035135-27080270	*OST4, EMILIN1, TMEM214, AGBL5*
TKHS190002643	NEC Cervix	DUP	chr2:27131672-27232694	*PREB, TCF23, PRR30, ATRAID, CAD, SLC5A6*
TKHS190002644	NEC Cervix	DUP	chr2:27207258-27234685	*ATRAID, CAD, SLC5A6*
TKHS190002644	NEC Cervix	DUP	chr2:27342826-27380526	*ZNF513, GTF3C2, EIF2B4, SNX17*
TKHS190002644	NEC Cervix	DUP	chr2:27437262-27449031	*KRTCAP3, IFT172, NRBP1*
TKHS190002644	NEC Cervix	DUP	chr2:28602821-28783978	*PLB1, SPDYA, PPP1CB*
TKHS190002644	NEC Cervix	DUP	chr2:28999181-29133816	*TOGARAM2, PCARE, CLIP4*
TKHS190002644	NEC Cervix	DUP	chr2:31372228-31403144	*XDH*
TKHS190002644	NEC Cervix	DUP	chr2:70921098-70985300	*VAX2, ATP6V1B1, ANKRD53*
TKHS190002644	NEC Cervix	DUP	chr2:74432467-74531371	*INO80B-WBP1, MRPL53, LBX2-AS1, DQX1, WBP1, HTRA2, TLX2, AUP1, RTKN, TTC31, MOGS, INO80B, PCGF1, CCDC142, LBX2*
TKHS190002641	NEC Ovary	DEL	chr2:96828567-96986313	*ANKRD23, CNNM3, ANKRD39, FAM178B, SEMA4C*
TKHS190002653	NEC Ovary	DUP	chr2:96863682-96986313	*FAM178B, SEMA4C*
TKHS190002641	NEC Ovary	DEL	chr2:97647067-97760914	*ACTR1B, COX5B, TMEM131, C2orf92, ZAP70*
TKHS190002644	NEC Cervix	DUP	chr2:9845220-9986282	*GRHL1, TAF1B*
TKHS190002654	NEC Cervix	DUP	chr20:10641460-10664014	*JAG1*
TKHS190002654	NEC Cervix	DUP	chr20:1266654-1320435	*FKBP1A-SDCBP2, SDCBP2, SNPH*
TKHS190002657	NEC Ovary	DEL	chr20:17614737-17636729	*RRBP1*
TKHS190002654	NEC Cervix	DUP	chr20:18542296-19886780	*C20orf78, LOC100130264, LINC00653, SEC23B, SCP2D1, LINC00652, SMIM26, RIN2, SLC24A3, DTD1*
TKHS190002654	NEC Cervix	DUP	chr20:1922313-2865495	*SNORD119, TMEM239, MIR1292, NOP56, TMC2, C20orf141, SIRPA, STK35, IDH3B, TGM6, PDYN, CPXM1, EBF4, PTPRA, VPS16, PCED1A, SNRPB, SNORA51, SNORD110, TGM3, ZNF343*
TKHS190002654	NEC Cervix	DUP	chr20:20050470-20640878	*CFAP61, INSM1, CRNKL1, RALGAPA2*
TKHS190002654	NEC Cervix	DUP	chr20:229278-417342	*RBCK1, C20orf96, DEFB129, DEFB132, TRIB3, SOX12, NRSN2, ZCCHC3*

TKHS190002655	NEC Cervix	DUP	chr20:2317068-2463227	*SNORD119, TGM6, SNRPB, TGM3*
TKHS190002654	NEC Cervix	DUP	chr20:24958928-25294292	*LOC284798, VSX1, APMAP, PYGB, ACSS1, CST7, ENTPD6*
TKHS190002654	NEC Cervix	DUP	chr20:3021309-3213226	*UBOX5-AS1, UBOX5, GNRH2, ITPA, OXT, AVP, PTPRA, FASTKD5, MRPS26, DDRGK1, LZTS3*
TKHS190002654	NEC Cervix	DUP	chr20:31368651-31722218	*MCTS2P, MIR3193, HM13-AS1, DEFB118, LINC00028, DEFB119, DEFB121, DEFB122, DEFB123, DEFB124, REM1, ID1, BCL2L1, HM13, COX4I2*
TKHS190002644	NEC Cervix	DEL	chr20:31819581-31845542	*FOXS1, MYLK2*
TKHS190002654	NEC Cervix	DUP	chr20:31819581-32316874	*DUSP15, TSPY26P, CCM2L, TTLL9, FOXS1, POFUT1, HCK, XKR7, PLAGL2, PDRG1, MYLK2, KIF3B, TM9SF4*
TKHS190002654	NEC Cervix	DUP	chr20:32366384-32703458	*NOL4L, NOL4L-DT, COMMD7, ASXL1, C20orf203*
TKHS190002654	NEC Cervix	DUP	chr20:32846618-33302412	*BPIFB6, BPIFA3, BPIFA2, SUN5, BPIFB4, MAPRE1, BPIFA4P, BPIFB3, BPIFA1, BPIFB2, BPIFB1*
TKHS190002655	NEC Cervix	DUP	chr20:33086021-33103014	*BPIFB4*
TKHS190002654	NEC Cervix	DUP	chr20:33408507-33680416	*C20orf144, ACTL10, E2F1, NECAB3, SNTA1, CBFA2T2*
TKHS190002654	NEC Cervix	DUP	chr20:34534628-34709524	*PIGU, TP53INP2, DYNLRB1, MAP1LC3A*
TKHS190002654	NEC Cervix	DUP	chr20:35384624-35669685	*RBM12, CEP250, C20orf173, ERGIC3, UQCC1, SPAG4, GDF5, CPNE1, NFS1*
TKHS190002654	NEC Cervix	DUP	chr20:35931249-36011257	*CNBD2, PHF20, SCAND1*
TKHS190002654	NEC Cervix	DUP	chr20:36195329-36634589	*TGIF2-RAB5IF, MYL9, EPB41L1, DLGAP4, AAR2, RAB5IF, TGIF2, SLA2*
TKHS190002654	NEC Cervix	DUP	chr20:3659909-3889728	*CENPB, HSPA12B, SPEF1, C20orf27, AP5S1, MAVS, GFRA4, SIGLEC1, PANK2, ADAM33, CDC25B*
TKHS190002654	NEC Cervix	DUP	chr20:37732879-38246126	*VSTM2L, CTNNBL1, RPRD1B, TGM2, KIAA1755, TTI1*
TKHS190002654	NEC Cervix	DUP	chr20:38623359-38725114	*SLC32A1, ARHGAP40*
TKHS190002654	NEC Cervix	DUP	chr20:38771559-38962407	*PPP1R16B, DHX35, ACTR5, FAM83D*
TKHS190002650	NEC Ovary	DUP	chr20:41162958-41358542	*ZHX3, PLCG1, LPIN3*
TKHS190002641	NEC Ovary	DEL	chr20:41169457-41412263	*ZHX3, PLCG1, LPIN3, CHD6, EMILIN3*
TKHS190002642	NEC Ovary	DUP	chr20:41172193-41416794	*ZHX3, PLCG1, LPIN3, CHD6, EMILIN3*
TKHS190002650	NEC Ovary	DUP	chr20:43686852-44186705	*GTSF1L, MYBL2, JPH2, TOX2*
TKHS190002650	NEC Ovary	DUP	chr20:45894823-46013796	*MMP9, PLTP, CTSA, ZNF335, PCIF1*
TKHS190002641	NEC Ovary	DEL	chr20:45949485-46013796	*MMP9, ZNF335*
TKHS190002654	NEC Cervix	DUP	chr20:45971210-46387437	*MMP9, SLC35C2, SLC12A5, NCOA5, ELMO2, ZNF335, CDH22, CD40*
TKHS190002641	NEC Ovary	DEL	chr20:46040373-46354965	*SLC35C2, SLC12A5, NCOA5, CDH22, CD40*
TKHS190002650	NEC Ovary	DUP	chr20:47224317-47298947	*ZMYND8*
TKHS190002650	NEC Ovary	DUP	chr20:48625885-48651583	*PREX1*
TKHS190002654	NEC Cervix	DUP	chr20:49874705-50818282	*TRERNA1, CEBPB, RIPOR3, SLC9A8, LINC01270, TMEM189, TMEM189-UBE2V1, BCAS4, RNF114, PTPN1, SNAI1, MIR645, UBE2V1, PARD6B, SPATA2*
TKHS190002653	NEC Ovary	DUP	chr20:51671115-51792352	*ATP9A, SALL4*
TKHS190002654	NEC Cervix	DUP	chr20:57366807-57659697	*NA, CTCFL, PCK1, RBM38, PMEPA1, ZBP1, RAE1*
TKHS190002654	NEC Cervix	DUP	chr20:5863024-5952528	*CHGB, SHLD1, TRMT6, MCM8*
TKHS190002654	NEC Cervix	DUP	chr20:604591-968138	*SLC52A3, SRXN1, RSPO4, ANGPT4, TCF15, FAM110A, SCRT2*

TKHS190002644	NEC Cervix	DEL	chr20:61844668-61929842	*CDH4*
TKHS190002654	NEC Cervix	DUP	chr20:61844668-62327669	*CDH4, MIR1257, ADRM1, HRH3, LSM14B, SS18L1, MTG2, LAMA5, PSMA7, TAF4, OSBPL2*
TKHS190002654	NEC Cervix	DUP	chr20:62346506-62395014	*LAMA5, RPS21, CABLES2*
TKHS190002654	NEC Cervix	DUP	chr20:62465340-62882414	*UCKL1-AS1, MIR941-1, MIR941-3, MIR1914, RGS19, SAMD10, ABHD16B, LKAAEAR1, PRPF6, NPBWR2, C20orf204, MYT1, OPRL1, SOX18, UCKL1, ZNF512B, TCEA2, MIR647, TPD52L2, DNAJC5*
TKHS190002644	NEC Cervix	DEL	chr20:62944739-63278995	*LINC00029, LINC01056, FLJ16779, MIR3196, BHLHE23, NKAIN4, MIR124-3, YTHDF1, GID8, ARFGAP1, SLC17A9, LINC01749, HAR1A, HAR1B, BIRC7*
TKHS190002654	NEC Cervix	DUP	chr20:62944739-63666079	*LINC00029, LINC01056, FLJ16779, MIR4326, MIR3196, RTEL1-TNFRSF6B, CHRNA4, BHLHE23, NKAIN4, EEF1A2, GMEB2, KCNQ2, MIR124-3, STMN3, RTEL1, YTHDF1, GID8, ARFGAP1, PTK6, COL20A1, SLC17A9, LINC01749, SRMS, HAR1A, HAR1B, FNDC11, PPDPF, BIRC7, HELZ2*
TKHS190002644	NEC Cervix	DEL	chr20:63326096-63442531	*CHRNA4, KCNQ2, COL20A1*
TKHS190002654	NEC Cervix	DUP	chr20:63692805-63993485	*UCKL1-AS1, MIR941-1, MIR941-3, MIR1914, RTEL1-TNFRSF6B, ARFRP1, ZBTB46, SAMD10, ABHD16B, PRPF6, RTEL1, LIME1, UCKL1, SLC2A4RG, ZNF512B, MIR647, TPD52L2, DNAJC5, ZGPAT, TNFRSF6B*
TKHS190002654	NEC Cervix	DUP	chr20:64066915-64240448	*RGS19, LKAAEAR1, NPBWR2, MYT1, OPRL1, TCEA2*
TKHS190002642	NEC Ovary	DUP	chr21:30319403-30561289	*MIR4327, KRTAP13-1, KRTAP15-1, KRTAP13-4, KRTAP19-1, KRTAP13-2, KRTAP13-3, KRTAP23-1, KRTAP19-2, KRTAP19-3, KRTAP19-4, KRTAP19-5, KRTAP19-6, KRTAP19-7, KRTAP26-1, KRTAP27-1*
TKHS190002641	NEC Ovary	DEL	chr21:41740838-41859228	*RIPK4, PRDM15*
TKHS190002641	NEC Ovary	DEL	chr21:41918105-42111710	*UMODL1-AS1, C2CD2, ZBTB21, UMODL1*
TKHS190002646	NEC Cervix	DEL	chr21:42271070-42444643	*UBASH3A, TMPRSS3, TFF1, TFF2, TFF3, ABCG1*
TKHS190002641	NEC Ovary	DEL	chr21:42765381-42862394	*WDR4, PDE9A*
TKHS190002641	NEC Ovary	DUP	chr21:44107417-44230089	*ICOSLG, PWP2, GATD3A*
TKHS190002654	NEC Cervix	DUP	chr21:44108984-44711514	*LRRC3-DT, TSPEAR-AS2, ICOSLG, DNMT3L, AIRE, KRTAP12-2, KRTAP12-1, KRTAP10-10, KRTAP10-4, KRTAP10-6, KRTAP10-7, KRTAP10-9, KRTAP10-1, KRTAP10-11, KRTAP10-2, KRTAP10-5, KRTAP10-8, KRTAP10-3, KRTAP12-3, KRTAP12-4, KRTAP10-12, PFKL, TSPEAR, PWP2, TRPM2, CFAP410, LRRC3, GATD3A*
TKHS190002657	NEC Ovary	DEL	chr21:44304275-44339194	*PFKL, CFAP410*
TKHS190002641	NEC Ovary	DUP	chr21:44508869-44638173	*TSPEAR-AS2, KRTAP10-10, KRTAP10-4, KRTAP10-6, KRTAP10-7, KRTAP10-9, KRTAP10-1, KRTAP10-2, KRTAP10-5, KRTAP10-8, KRTAP10-3, TSPEAR*
TKHS190002653	NEC Ovary	DUP	chr21:45981851-46182816	*FTCD, COL6A1, COL6A2, SPATC1L*
TKHS190002654	NEC Cervix	DUP	chr21:45999153-46112824	*COL6A1, COL6A2*
TKHS190002641	NEC Ovary	DEL	chr21:46001253-46275325	*FTCD, MCM3AP-AS1, COL6A1, COL6A2, LSS, SPATC1L, MCM3AP*
TKHS190002646	NEC Cervix	DEL	chr21:46138880-46209625	*FTCD, LSS, SPATC1L*
TKHS190002647	NEC Endometrium	DUP	chr22:20139194-20149640	*ZDHHC8, CCDC188*
TKHS190002654	NEC Cervix	DUP	chr22:21000647-21030351	*TUBA3FP, THAP7-AS1, SLC7A4, THAP7, P2RX6*
TKHS190002647	NEC Endometrium	DUP	chr22:24184766-24231530	*GGT5, SUSD2*
TKHS190002647	NEC Endometrium	DUP	chr22:25768115-26457967	*SEZ6L, ASPHD2, MYO18B, HPS4*
TKHS190002648	NEC Cervix	DEL	chr22:30893638-30935322	*MORC2-AS1, MORC2, OSBP2*
TKHS190002643	NEC Cervix	DUP	chr22:36145473-36266033	*APOL2, APOL4, APOL3, APOL1*
TKHS190002643	NEC Cervix	DUP	chr22:36314145-36518910	*TXN2, MYH9, FOXRED2, EIF3D*
TKHS190002643	NEC Cervix	DUP	chr22:37832958-37886845	*ANKRD54, EIF3L, MIR658, MIR659*

TKHS190002643	NEC Cervix	DUP	chr22:38670884-38729662	*CBY1, TOMM22, GTPBP1, JOSD1*
TKHS190002647	NEC Endometrium	DEL	chr22:42127781-42129185	*NDUFA6-DT, CYP2D6*
TKHS190002647	NEC Endometrium	DUP	chr22:43171819-43231875	*TTLL12, SCUBE1*
TKHS190002643	NEC Cervix	DUP	chr22:45527847-45564992	*FBLN1*
TKHS190002643	NEC Cervix	DUP	chr22:50229404-50248145	*HDAC10, TUBGCP6*
TKHS190002648	NEC Cervix	DEL	chr22:50316174-50423614	*DENND6B, PPP6R2*
TKHS190002647	NEC Endometrium	DUP	chr22:50603223-50740694	*MAPK8IP2, ARSA, ACR, SHANK3*
TKHS190002645	NEC Endometrium	DUP	chr3:100629598-100695001	*ADGRG7*
TKHS190002644	NEC Cervix	DUP	chr3:121911574-122022077	*ILDR1, SLC15A2*
TKHS190002644	NEC Cervix	DUP	chr3:122414536-122577853	*DTX3L, PARP15, KPNA1, WDR5B, PARP9*
TKHS190002644	NEC Cervix	DUP	chr3:122680859-122718958	*PARP14*
TKHS190002645	NEC Endometrium	DUP	chr3:126914680-127034017	*PLXNA1, CHCHD6*
TKHS190002648	NEC Cervix	DEL	chr3:126988594-127030064	*PLXNA1*
TKHS190002653	NEC Ovary	DUP	chr3:128123584-128625653	*DNAJB8, LINC01565, GATA2, DNAJB8-AS1, EEFSEC, RPN1, RUVBL1, LOC90246*
TKHS190002644	NEC Cervix	DUP	chr3:134357705-134365909	*AMOTL2*
TKHS190002644	NEC Cervix	DUP	chr3:136854461-136995556	*NCK1, IL20RB, SLC35G2*
TKHS190002644	NEC Cervix	DEL	chr3:14819072-14922548	*FGD5-AS1, FGD5*
TKHS190002644	NEC Cervix	DUP	chr3:149145354-149221792	*CP, HPS3*
TKHS190002644	NEC Cervix	DUP	chr3:158571301-158734037	*MLF1, LXN, RARRES1, GFM1*
TKHS190002644	NEC Cervix	DUP	chr3:172127028-172820237	*ECT2, GHSR, NCEH1, FNDC3B, TNFSF10*
TKHS190002654	NEC Cervix	DEL	chr3:172506492-172760263	*ECT2, NCEH1, TNFSF10*
TKHS190002648	NEC Cervix	DEL	chr3:184164492-184189277	*AP2M1, DVL3, ABCF3*
TKHS190002656	NEC Cervix	DEL	chr3:184180169-184193452	*AP2M1, ABCF3*
TKHS190002654	NEC Cervix	DUP	chr3:184234266-184236749	*VWA5B2*
TKHS190002644	NEC Cervix	DUP	chr3:184299851-184354314	*CLCN2, FAM131A, EIF4G1, PSMD2, SNORD66*
TKHS190002656	NEC Cervix	DEL	chr3:184308708-184383642	*CLCN2, FAM131A, EIF4G1, POLR2H, PSMD2, SNORD66, THPO, CHRD*
TKHS190002644	NEC Cervix	DUP	chr3:186787127-187236567	*ADIPOQ-AS1, RPL39L, RTP1, EIF4A2, MASP1, RFC4, SNORA63, SNORA4, ST6GAL1, ADIPOQ*
TKHS190002644	NEC Cervix	DUP	chr3:189310386-190429731	*MIR944, CLDN16, TMEM207, TPRG1, P3H2, TP63, CLDN1*
TKHS190002644	NEC Cervix	DEL	chr3:38097744-38122605	*DLEC1*
TKHS190002644	NEC Cervix	DEL	chr3:46989511-47019840	*NRADDP, NBEAL2, SETD2*
TKHS190002657	NEC Ovary	DEL	chr3:47407130-47426169	*SCAP, PTPN23*
TKHS190002644	NEC Cervix	DEL	chr3:48565636-48569760	*COL7A1*
TKHS190002644	NEC Cervix	DEL	chr3:48586074-48593696	*COL7A1*

TKHS190002644	NEC Cervix	DEL	chr3:48605770-48642467	*CELSR3, TMEM89, SLC26A6, UQCRC1*
TKHS190002645	NEC Endometrium	DEL	chr3:49096029-49104733	*QARS1*
TKHS190002645	NEC Endometrium	DEL	chr3:49129839-49132867	*LAMB2*
TKHS190002644	NEC Cervix	DEL	chr3:49530797-49663886	*BSN-DT, DAG1, BSN*
TKHS190002644	NEC Cervix	DEL	chr3:50193265-50257690	*SLC38A3, GNAI2, GNAT1*
TKHS190002657	NEC Ovary	DEL	chr3:50275012-50292466	*LSMEM2, IFRD2, SEMA3B*
TKHS190002644	NEC Cervix	DEL	chr3:50367812-50434429	*CACNA2D2*
TKHS190002654	NEC Cervix	DUP	chr3:50375644-50434429	*CACNA2D2*
TKHS190002644	NEC Cervix	DEL	chr3:51712473-51937736	*IQCF4, IQCF1, GRM2, IQCF2, IQCF5, IQCF3, IQCF6, RRP9*
TKHS190002654	NEC Cervix	DUP	chr3:52278201-52396538	*GLYCTK, DNAH1, MIR135A1, WDR82*
TKHS190002644	NEC Cervix	DEL	chr3:52287094-52396538	*GLYCTK, DNAH1, MIR135A1*
TKHS190002642	NEC Ovary	DUP	chr3:52331148-52361352	*DNAH1*
TKHS190002654	NEC Cervix	DUP	chr3:52435603-52442304	*SEMA3G*
TKHS190002644	NEC Cervix	DEL	chr3:52435603-52529334	*NISCH, STAB1, SEMA3G, NT5DC2, TNNC1*
TKHS190002654	NEC Cervix	DUP	chr3:52470859-52528935	*NISCH, STAB1, NT5DC2*
TKHS190002657	NEC Ovary	DEL	chr3:52484513-52536906	*NISCH, STAB1, SMIM4, NT5DC2*
TKHS190002654	NEC Cervix	DUP	chr3:53495167-53735503	*CACNA1D*
TKHS190002654	NEC Cervix	DUP	chr3:97895959-97967593	*CRYBG3, RIOX2*
TKHS190002654	NEC Cervix	DUP	chr4:139719323-139889306	*MGST2, MAML3*
TKHS190002641	NEC Ovary	DUP	chr4:2898431-2933663	*MFSD10, ADD1*
TKHS190002642	NEC Ovary	DEL	chr4:2928171-2933663	*MFSD10, ADD1*
TKHS190002641	NEC Ovary	DUP	chr4:6287093-6348010	*PPP2R2C, WFS1*
TKHS190002644	NEC Cervix	DUP	chr4:73740659-74099120	*PPBPP2, CXCL1, CXCL2, CXCL3, CXCL8, PF4, PF4V1, PPBP, CXCL6, CXCL5*
TKHS190002643	NEC Cervix	DUP	chr4:7774739-7984893	*AFAP1, ABLIM2, AFAP1-AS1*
TKHS190002654	NEC Cervix	DUP	chr4:8008042-8080802	*ABLIM2*
TKHS190002643	NEC Cervix	DUP	chr4:8366961-8416521	*ACOX3*
TKHS190002641	NEC Ovary	DUP	chr4:932043-1002485	*SLC26A1, DGKQ, GAK, IDUA, TMEM175*
TKHS190002653	NEC Ovary	DUP	chr5:1032152-1074671	*MIR4635, SLC12A7, NKD2*
TKHS190002646	NEC Cervix	DEL	chr5:1032152-1076812	*MIR4635, SLC12A7, NKD2*
TKHS190002641	NEC Ovary	DEL	chr5:1032152-1321819	*MIR4457, MIR4635, SLC12A7, SLC6A19, SLC6A18, TERT, CLPTM1L, NKD2*
TKHS190002642	NEC Ovary	DUP	chr5:116292451-116504944	*COMMD10, SEMA6A*
TKHS190002642	NEC Ovary	DUP	chr5:1210444-1282624	*SLC6A19, SLC6A18, TERT*
TKHS190002646	NEC Cervix	DEL	chr5:1232219-1321819	*MIR4457, SLC6A18, TERT, CLPTM1L*

TKHS190002653	NEC Ovary	DUP	chr5:134115321-134146521	*TCF7*
TKHS190002654	NEC Cervix	DUP	chr5:14479919-14504593	*TRIO*
TKHS190002644	NEC Cervix	DUP	chr5:149003738-149063022	*SH3TC2*
TKHS190002643	NEC Cervix	DUP	chr5:151663455-151676188	*SPARC*
TKHS190002650	NEC Ovary	DUP	chr5:176337062-177096357	*MIR4281, GPRIN1, CLTB, HIGD2A, FGFR4, RNF44, FAF2, ZNF346, EIF4E1B, TSPAN17, ARL10, HK3, SIMC1, NOP16, UIMC1, CDHR2, KIAA1191, SNCB, UNC5A*
TKHS190002641	NEC Ovary	DEL	chr5:176530582-176896159	*MIR4281, GPRIN1, RNF44, EIF4E1B, TSPAN17, HK3, CDHR2, SNCB, UNC5A*
TKHS190002650	NEC Ovary	DUP	chr5:177267562-177441053	*RGS14, LMAN2, F12, PRELID1, GRK6, PRR7-AS1, PFN3, RAB24, NSD1, SLC34A1, MXD3*
TKHS190002648	NEC Cervix	DEL	chr5:177291954-177404664	*RGS14, LMAN2, F12, PRELID1, PFN3, RAB24, NSD1, SLC34A1, MXD3*
TKHS190002641	NEC Ovary	DEL	chr5:177291954-177441053	*RGS14, LMAN2, F12, PRELID1, GRK6, PRR7-AS1, PFN3, RAB24, NSD1, SLC34A1, MXD3*
TKHS190002650	NEC Ovary	DUP	chr5:180619665-180631778	*FLT4*
TKHS190002641	NEC Ovary	DEL	chr5:306602-454051	*PDCD6, EXOC3, EXOC3-AS1, AHRR*
TKHS190002642	NEC Ovary	DUP	chr5:58454583-58459092	*PLK2*
TKHS190002653	NEC Ovary	DUP	chr5:58455539-58459959	*PLK2*
TKHS190002643	NEC Cervix	DEL	chr5:716808-833807	*ZDHHC11*
TKHS190002648	NEC Cervix	DUP	chr6:100393487-100449699	*SIM1*
TKHS190002648	NEC Cervix	DUP	chr6:136557758-137009492	*NHEG1, SLC35D3, MAP3K5, PEX7, IL20RA*
TKHS190002648	NEC Cervix	DUP	chr6:138170662-138512339	*MIR3145, HEBP2, ARFGEF3, NHSL1, PBOV1*
TKHS190002648	NEC Cervix	DUP	chr6:16143699-16328310	*GMPR, MYLIP, ATXN1*
TKHS190002641	NEC Ovary	DUP	chr6:16143699-17110880	*GMPR, MYLIP, STMND1, ATXN1*
TKHS190002648	NEC Cervix	DUP	chr6:167136240-167359412	*CCR6, GPR31, TCP10L2, UNC93A, TCP10, TTLL2*
TKHS190002648	NEC Cervix	DUP	chr6:167951186-168608239	*HGC6.3, DACT2, KIF25, AFDN, SMOC2, FRMD1*
TKHS190002643	NEC Cervix	DUP	chr6:26092685-26368912	*BTN3A2, H1-3, H1-4, H1-6, H2AC8, H2AC7, H2BC5, HFE, H2AC6, H2BC8, H2BC7, H2BC6, H2BC9, H2BC10, H2BC4, H3C4, H3C6, H3C8, H4C4, H4C6, H4C3, H4C8, H4C5, H4C7, H3C7*
TKHS190002641	NEC Ovary	DUP	chr6:27248847-27255131	*PRSS16*
TKHS190002641	NEC Ovary	DUP	*chr6:29440010-31980801*	
TKHS190002646	NEC Cervix	DUP	*chr6:29727957-30075789*	
TKHS190002646	NEC Cervix	DUP	*chr6:30556131-31148728*	
TKHS190002646	NEC Cervix	DUP	*chr6:31540322-31972757*	
TKHS190002648	NEC Cervix	DUP	*chr6:31766245-31792895*	
TKHS190002641	NEC Ovary	DUP	*chr6:32026983-32316414*	
TKHS190002646	NEC Cervix	DUP	*chr6:32039356-32217067*	
TKHS190002643	NEC Cervix	DUP	*chr6:32169966-32188796*	
TKHS190002648	NEC Cervix	DUP	*chr6:32202076-32223928*	
TKHS190002641	NEC Ovary	DUP	chr6:32641972-33711686	*HLA-DQA1, ITPR3, LINC00336, GGNBP1, BAK1, UQCC2*
TKHS190002643	NEC Cervix	DUP	*chr6:32841531-32852502*	
TKHS190002646	NEC Cervix	DUP	chr6:33168955-33575441	*BAK1*

TKHS190002643	NEC Cervix	DUP	*chr6:33201246-33205912*	
TKHS190002643	NEC Cervix	DUP	*chr6:33287614-33290450*	
TKHS190002643	NEC Cervix	DUP	*chr6:33412264-33438919*	
TKHS190002648	NEC Cervix	DUP	chr6:33442889-33687327	*ITPR3, LINC00336, GGNBP1, BAK1*
TKHS190002641	NEC Ovary	DUP	chr6:34527332-34544455	*SPDEF, PACSIN1*
TKHS190002648	NEC Cervix	DUP	chr6:35242622-35462914	*FANCE, SCUBE3, DEF6, PPARD, ZNF76*
TKHS190002641	NEC Ovary	DUP	chr6:35242622-35466345	*FANCE, SCUBE3, DEF6, PPARD, ZNF76*
TKHS190002648	NEC Cervix	DUP	chr6:35480312-35506173	*TEAD3, TULP1*
TKHS190002641	NEC Ovary	DUP	chr6:36052699-36291555	*MAPK14, BRPF3, PNPLA1, MAPK13*
TKHS190002648	NEC Cervix	DUP	chr6:36131271-36330690	*BRPF3, PNPLA1, BNIP5, MAPK13*
TKHS190002641	NEC Ovary	DUP	chr6:37170303-37360574	*TMEM217, PIM1, TBC1D22B, RNF8*
TKHS190002641	NEC Ovary	DUP	chr6:37462829-37696811	*MIR4462, CCDC167, CMTR1, MDGA1*
TKHS190002648	NEC Cervix	DUP	chr6:37484810-37696811	*MIR4462, CCDC167, MDGA1*
TKHS190002641	NEC Ovary	DUP	chr6:39026546-39316947	*DNAH8, GLP1R, SAYSD1, KCNK16, KCNK5, KCNK17*
TKHS190002648	NEC Cervix	DUP	chr6:39887486-41201008	*APOBEC2, ADCY10P1, OARD1, TSPO2, UNC5CL, DAAM2, TREML1, LINC00951, MOCS1, NFYA, TREM2, LRFN2, TDRG1, TREML2*
TKHS190002648	NEC Cervix	DUP	chr6:41335877-41597242	*FOXP4, NCR2*
TKHS190002641	NEC Ovary	DUP	chr6:41336087-41597242	*FOXP4, NCR2*
TKHS190002641	NEC Ovary	DUP	chr6:41653319-42185797	*TOMM6, FRS3, TAF8, USP49, GUCA1A, GUCA1B, PRICKLE4, MDFI, PGC, C6orf132, BYSL, TFEB, CCND3, MED20*
TKHS190002648	NEC Cervix	DUP	chr6:42128672-42185797	*GUCA1A, GUCA1B, C6orf132*
TKHS190002646	NEC Cervix	DUP	chr6:42935537-42966657	*CNPY3, GNMT, PEX6*
TKHS190002641	NEC Ovary	DUP	chr6:42935537-42966657	*CNPY3, GNMT, PEX6*
TKHS190002648	NEC Cervix	DUP	chr6:42962212-42968498	*GNMT, PEX6*
TKHS190002641	NEC Ovary	DUP	chr6:43056307-43521300	*DNPH1, SLC22A7, LRRC73, CUL9, ZNF318, YIPF3, CRIP3, MRPL2, PTK7, DLK2, SRF, TTBK1, ABCC10, KLC4, TJAP1, POLR1C*
TKHS190002648	NEC Cervix	DUP	chr6:43128977-43512563	*DNPH1, SLC22A7, LRRC73, CUL9, ZNF318, YIPF3, CRIP3, PTK7, DLK2, SRF, TTBK1, ABCC10, TJAP1*
TKHS190002646	NEC Cervix	DUP	chr6:43146618-43306124	*DNPH1, SLC22A7, CUL9, CRIP3, PTK7, SRF, TTBK1*
TKHS190002641	NEC Ovary	DUP	chr6:44002681-44287825	*MIR4647, CAPN11, TCTE1, SLC29A1, SPATS1, C6orf223, HSP90AB1, SLC35B2, TMEM151B, NFKBIE, TMEM63B, MRPL14*
TKHS190002646	NEC Cervix	DUP	chr6:44135017-44183235	*CAPN11, TMEM63B*
TKHS190002648	NEC Cervix	DUP	chr6:44146847-44230665	*CAPN11, SLC29A1, TMEM63B*
TKHS190002648	NEC Cervix	DUP	chr6:44273066-44310443	*TCTE1, SPATS1, TMEM151B, AARS2*
TKHS190002641	NEC Ovary	DEL	chr7:100090399-100198582	*MIR4658, STAG3, COPS6, GPC2, CNPY4, MBLAC1, LAMTOR4, MIR106B, MIR25, MIR93, MCM7, MAP11, TAF6, GAL3ST4, AP4M1*
TKHS190002641	NEC Ovary	DEL	chr7:100590174-100606630	*PCOLCE-AS1, FBXO24, PCOLCE*
TKHS190002641	NEC Ovary	DEL	chr7:100755226-100819889	*EPHB4, ZAN*
TKHS190002641	NEC Ovary	DEL	chr7:101211587-101216786	*PLOD3*
TKHS190002653	NEC Ovary	DUP	chr7:101321671-101622200	*COL26A1, IFT22, MYL10*

TKHS190002642	NEC Ovary	DUP	chr7:101553326-101624264	*COL26A1, MYL10*
TKHS190002657	NEC Ovary	DUP	chr7:128807360-128840160	*FLNC, CCDC136*
TKHS190002653	NEC Ovary	DUP	chr7:132174778-132198636	*PLXNA4*
TKHS190002657	NEC Ovary	DUP	chr7:135362784-135422353	*CNOT4*
TKHS190002643	NEC Cervix	DEL	chr7:142065715-142094863	*MGAM*
TKHS190002650	NEC Ovary	DEL	*chr7:142384379-142520138*	
TKHS190002655	NEC Cervix	DUP	*chr7:142384544-142507810*	
TKHS190002657	NEC Ovary	DUP	chr7:149250159-149288691	*ZNF783, LOC155060, ZNF212*
TKHS190002657	NEC Ovary	DUP	chr7:149492859-150340609	*ZBED6CL, ZNF746, ATP6V0E2, ZNF467, SSPO, ATP6V0E2-AS1, RARRES2, ZNF862, ACTR3C, LRRC61, ZNF767P, KRBA1*
TKHS190002641	NEC Ovary	DEL	chr7:149721380-149832923	*ZNF467, SSPO, KRBA1*
TKHS190002646	NEC Cervix	DEL	chr7:149721380-149879677	*ATP6V0E2, ZNF467, SSPO, ATP6V0E2-AS1, ZNF862, KRBA1*
TKHS190002657	NEC Ovary	DUP	chr7:150856471-150977913	*AOC1, KCNH2*
TKHS190002657	NEC Ovary	DUP	chr7:151011788-151239746	*ABCF2, CDK5, FASTK, ABCB8, AGAP3, ASB10, GBX1, ATG9B, NOS3, CHPF2, SLC4A2, SMARCD3, MIR671, TMUB1, ASIC3*
TKHS190002646	NEC Cervix	DEL	chr7:151074399-151079021	*FASTK, SLC4A2*
TKHS190002646	NEC Cervix	DEL	chr7:151222521-151239746	*ABCF2, CHPF2, SMARCD3, MIR671*
TKHS190002657	NEC Ovary	DUP	chr7:154991006-155358179	*HTR5A-AS1, PAXIP1-AS1, PAXIP1, HTR5A, INSIG1*
TKHS190002657	NEC Ovary	DUP	chr7:2524655-2538944	*MIR4648, BRAT1, LFNG*
TKHS190002641	NEC Ovary	DEL	chr7:2649913-2700755	*AMZ1, TTYH3*
TKHS190002650	NEC Ovary	DEL	chr7:3969257-4178586	*SDK1*
TKHS190002657	NEC Ovary	DUP	chr7:4158448-4178586	*SDK1*
TKHS190002657	NEC Ovary	DUP	chr7:44110015-44114261	*MIR4649, AEBP1*
TKHS190002657	NEC Ovary	DUP	chr7:44707582-44801422	*OGDH, PPIA, ZMIZ2*
TKHS190002657	NEC Ovary	DUP	chr7:5489807-5623179	*RNF216, ACTB, FSCN1, MIR589, FBXL18*
TKHS190002641	NEC Ovary	DEL	chr7:5603257-5623179	*RNF216, FSCN1*
TKHS190002657	NEC Ovary	DUP	chr7:56068482-56084244	*SUMF2, PHKG1, PSPH*
TKHS190002643	NEC Cervix	DUP	chr7:5919849-5960864	*RSPH10B, CCZ1*
TKHS190002648	NEC Cervix	DEL	chr7:6139092-6171314	*USP42, CYTH3*
TKHS190002643	NEC Cervix	DEL	chr7:6763928-6805055	*CCZ1B, RSPH10B2*
TKHS190002657	NEC Ovary	DUP	chr7:729663-926644	*ADAP1, SUN1, GET4, DNAAF5*
TKHS190002657	NEC Ovary	DUP	chr7:73303826-73459718	*TRIM50, NSUN5, FZD9, FKBP6, BAZ1B*
TKHS190002641	NEC Ovary	DEL	chr7:74093250-74601297	*LIMK1, RFC2, MIR590, EIF4H, CLIP2, LAT2, GTF2IRD1*
TKHS190002657	NEC Ovary	DUP	chr7:74249739-74254389	*RFC2*
TKHS190002650	NEC Ovary	DUP	chr7:74317547-74538760	*CLIP2, GTF2IRD1*
TKHS190002646	NEC Cervix	DUP	chr7:75007994-75066792	*NA, RCC1L*

TKHS190002642	NEC Ovary	DUP	chr7:87193198-87444975	*TP53TG1, ABCB4, CROT, TMEM243, DMTF1*
TKHS190002642	NEC Ovary	DUP	chr7:90160805-99624180	*TMEM225B, MIR3609, NA, ATP5MF-PTCD1, MIR4652, ARPC1B, LOC101409256, AKAP9, SLC25A13, CDK6, BET1, ARPC1A, CPSF4, PDAP1, COL1A2, CYP51A1, BHLHA15, DLX5, DLX6, DYNC1I1, SAMD9L, ZSCAN25, FAM200A, TMEM130, LMTK2, PEG10, ZKSCAN5, HEPACAM2, FAM133B, BRI3, TECPR1, PTCD1, STEAP2, STEAP1, GNG11, GNGT1, DLX6-AS1, ZNF789, CCZ1P-OR7E38P, LRRD1, NA, KPNA7, ASNS, NPTX2, OCM2, PDK4, ASB4, PEX1, CDK14, PON1, PON2, PON3, ANKIB1, SAMD9, PPP1R9A, VPS50, BAIAP2L1, SDHAF3, SMURF1, MIR489, GATAD1, CASD1, TAC1, MIR591, MIR653, ZNF655, MTERF1, SEM1, TFPI2, CFAP69, CALCR, TRRAP, FZD1, RBM48, ZNF394, MYH16, NA, GTPBP10, KRIT1, BUD31, SGCE, CLDN12, ATP5MF*
TKHS190002641	NEC Ovary	DEL	chr7:98848056-99035716	*MIR3609, TMEM130, SMURF1, TRRAP*
TKHS190002642	NEC Ovary	DUP	chr7:99848166-101216786	*PCOLCE-AS1, SAP25, MIR4653, MIR4658, MUC12, POP7, STAG3, COPS6, AP1S1, MUC17, EPHB4, EPO, PPP1R35, GPC2, NYAP1, CNPY4, MBLAC1, FBXO24, GNB2, PILRB, PILRA, AGFG2, MOGAT3, GJC3, CASTOR3, NAT16, LAMTOR4, C7orf61, UFSP1, LRCH4, MIR106B, MIR25, MIR93, MCM7, ACHE, SPDYE3, SERPINE1, PCOLCE, ACTL6B, SRRT, PMS2P1, ZCWPW1, MAP11, MEPCE, AZGP1, SLC12A9, MOSPD3, GIGYF1, AZGP1P1, CYP3A43, TAF6, TFR2, TRIP6, VGF, ZAN, ZNF3, ZKSCAN1, ZSCAN21, PVRIG, GAL3ST4, OR2AE1, TSC22D4, TRIM56, TRIM4, PLOD3, AP4M1*
TKHS190002648	NEC Cervix	DUP	chr8:101543241-104499519	*MIR3151, BAALC-AS1, MIR5680, CTHRC1, BAALC-AS2, DPYS, DCAF13, LRP12, ODF1, RRM2B, UBR5, AZIN1, ATP6V1C1, KLF10, BAALC, GRHL2, SLC25A32, DCSTAMP, FZD6, NCALD, RIMS2*
TKHS190002648	NEC Cervix	DUP	chr8:108239958-109498771	*TMEM74, EIF3E, ENY2, TRHR, NUDCD1, PKHD1L1, EMC2*
TKHS190002648	NEC Cervix	DUP	chr8:10834666-13115657	*FAM66D, FAM66A, MIR3926-2, MIR3926-1, LOC100506990, LINC00681, DLC1, CTSB, TDH, FDFT1, DEFB109A, DEFB130A, NEIL2, GATA4, XKR6, LOC340357, USP17L2, FAM90A25P, LOC392196, USP17L7, PINX1, TRMT9B, DEFB135, DEFB136, DEFB134, BLK, LOC649352, FAM86B2, MTMR9, MIR598, ZNF705D, FAM90A2P, LOC729732, SOX7, FAM167A, SLC35G5, LINC00208, FAM167A-AS1, FAM86B1, LONRF1*
TKHS190002648	NEC Cervix	DUP	chr8:112247020-113019179	*MIR2053, CSMD3*
TKHS190002643	NEC Cervix	DEL	chr8:11331107-11561452	*TDH, BLK, FAM167A, SLC35G5, FAM167A-AS1*
TKHS190002648	NEC Cervix	DUP	chr8:116657215-119783633	*MIR3610, COLEC10, MAL2, SLC30A8, EXT1, SAMD12, AARD, CCN3, TNFRSF11B, ENPP2, SAMD12-AS1, RAD21, RAD21-AS1, TAF2, UTP23, EIF3H, MED30*
TKHS190002642	NEC Ovary	DUP	chr8:119562857-119638780	*ENPP2*
TKHS190002648	NEC Cervix	DUP	chr8:119853047-120283778	*DEPTOR, COL14A1, DSCC1*
TKHS190002642	NEC Ovary	DUP	chr8:123019195-123231757	*FAM83A-AS1, ZHX1-C8orf76, MIR4663, DERL1, C8orf76, FAM83A, TBC1D31*
TKHS190002642	NEC Ovary	DUP	chr8:123429983-123737334	*FBXO32, ANXA13, KLHL38, WDYHV1*
TKHS190002648	NEC Cervix	DUP	chr8:123973434-124095038	*FER1L6-AS1, FER1L6*
TKHS190002643	NEC Cervix	DEL	chr8:124010594-124095038	*FER1L6-AS1, FER1L6*
TKHS190002642	NEC Ovary	DUP	chr8:124013431-124076325	*FER1L6-AS1, FER1L6*
TKHS190002648	NEC Cervix	DUP	chr8:124547000-125021945	*ZNF572, LINC00964, NDUFB9, SQLE, MTSS1*
TKHS190002642	NEC Ovary	DUP	chr8:124565662-125000071	*ZNF572, LINC00964, NDUFB9, SQLE, MTSS1*
TKHS190002648	NEC Cervix	DUP	chr8:130821342-132087739	*HHLA1, ADCY8, EFR3A, OC90*
TKHS190002643	NEC Cervix	DEL	chr8:132003236-132184367	*HHLA1, EFR3A, KCNQ3, OC90*
TKHS190002648	NEC Cervix	DUP	chr8:132777829-132868227	*PHF20L1, TG*
TKHS190002642	NEC Ovary	DUP	chr8:132836540-133116716	*PHF20L1, SLA, TG*

TKHS190002653	NEC Ovary	DUP	chr8:132844156-133464957	*NDRG1, PHF20L1, ST3GAL1, SLA, TG, CCN4*
TKHS190002643	NEC Cervix	DEL	chr8:138137147-138606452	*COL22A1, FAM135B*
TKHS190002648	NEC Cervix	DUP	chr8:138137147-138844158	*COL22A1, FAM135B*
TKHS190002642	NEC Ovary	DUP	chr8:138138986-138195307	*FAM135B*
TKHS190002648	NEC Cervix	DUP	chr8:140458281-142879813	*LNCOC1, MIR4472-1, PTP4A3, LYPD2, CYP11B1, TSNARE1, DENND3, ARC, AGO2, GML, GPR20, MROH5, THEM6, CHRAC1, LY6K, SLURP1, SLC45A4, PTK2, ADGRB1, LINC00051, LYNX1, LINC01300, PSCA, TRAPPC9, LY6D, JRK*
TKHS190002642	NEC Ovary	DUP	chr8:141150286-143380035	*CDC42P3, LY6E-DT, LNCOC1, MIR4472-1, PTP4A3, RHPN1, TOP1MT, LYPD2, CYP11B1, CYP11B2, TSNARE1, DENND3, ARC, GLI4, GML, GPR20, C8orf31, ZFP41, GPIHBP1, MROH5, LY6E, LY6H, THEM6, LY6K, SLURP1, SLC45A4, ADGRB1, LINC00051, LYNX1, LINC01300, RHPN1-AS1, ZNF696, PSCA, LY6D, JRK*
TKHS190002643	NEC Cervix	DEL	chr8:141217090-143380035	*CDC42P3, LY6E-DT, LNCOC1, MIR4472-1, PTP4A3, RHPN1, TOP1MT, LYPD2, CYP11B1, CYP11B2, TSNARE1, ARC, GLI4, GML, GPR20, C8orf31, ZFP41, GPIHBP1, MROH5, LY6E, LY6H, THEM6, LY6K, SLURP1, SLC45A4, ADGRB1, LINC00051, LYNX1, LINC01300, RHPN1-AS1, ZNF696, PSCA, LY6D, JRK*
TKHS190002648	NEC Cervix	DUP	chr8:143269397-143560771	*RHPN1, TOP1MT, ZC3H3, GLI4, ZFP41, MAFA, RHPN1-AS1, GSDMD, ZNF696*
TKHS190002653	NEC Ovary	DUP	chr8:143558378-143616220	*EEF1D, MROH6, PYCR3, TSTA3, GSDMD, TIGD5, NAPRT*
TKHS190002648	NEC Cervix	DUP	chr8:143577269-144264831	*MIR937, FAM83H-AS1, CCDC166, MIR4664, CYC1, EEF1D, MAPK15, PUF60, SCRIB, OPLAH, ZNF707, BREA2, FAM83H, GRINA, NRBP2, WDR97, SPATC1, HGH1, PLEC, EXOSC4, PYCR3, MIR661, TSTA3, MROH1, SHARPIN, EPPK1, MAF1, PARP10, TIGD5, GPAA1, NAPRT, ZNF623*
TKHS190002642	NEC Ovary	DUP	chr8:143608062-143927992	*MIR937, FAM83H-AS1, CCDC166, MIR4664, MAPK15, PUF60, SCRIB, ZNF707, BREA2, FAM83H, NRBP2, PLEC, PYCR3, TSTA3, EPPK1, ZNF623*
TKHS190002643	NEC Cervix	DEL	chr8:143691073-143932032	*MIR937, FAM83H-AS1, CCDC166, MIR4664, MAPK15, PUF60, SCRIB, ZNF707, BREA2, FAM83H, NRBP2, PLEC, EPPK1*
TKHS190002654	NEC Cervix	DEL	chr8:143716378-143730582	*MAPK15, FAM83H*
TKHS190002653	NEC Ovary	DUP	chr8:143812817-144096029	*MIR937, CYC1, PUF60, SCRIB, OPLAH, GRINA, NRBP2, SPATC1, PLEC, EXOSC4, MIR661, EPPK1, PARP10, GPAA1*
TKHS190002641	NEC Ovary	DEL	chr8:143840706-143932032	*NRBP2, PLEC, EPPK1*
TKHS190002654	NEC Cervix	DEL	chr8:143926977-143946392	*PLEC, MIR661*
TKHS190002643	NEC Cervix	DEL	chr8:143983167-144200541	*CYC1, OPLAH, GRINA, WDR97, SPATC1, HGH1, EXOSC4, MROH1, SHARPIN, MAF1, PARP10, GPAA1*
TKHS190002642	NEC Ovary	DUP	chr8:143983167-144517190	*MIR939, MIR1234, MFSD3, CYC1, ADCK5, BOP1, FBXL6, OPLAH, GPT, GRINA, CPSF1, HSF1, WDR97, TMEM249, SPATC1, TONSL, CYHR1, VPS28, HGH1, EXOSC4, SLC39A4, SCX, MROH1, SLC52A2, SHARPIN, SCRT1, MAF1, PARP10, PPP1R16A, DGAT1, GPAA1, FOXH1, KIFC2, RECQL4*
TKHS190002641	NEC Ovary	DEL	chr8:144264225-144475756	*MIR939, MIR1234, ADCK5, BOP1, FBXL6, CPSF1, HSF1, TMEM249, TONSL, CYHR1, VPS28, HGH1, SLC39A4, SCX, SLC52A2, SCRT1, DGAT1, FOXH1, KIFC2*
TKHS190002648	NEC Cervix	DUP	chr8:144497195-144517190	*MFSD3, GPT, PPP1R16A, RECQL4*
TKHS190002642	NEC Ovary	DUP	chr8:144537721-144946206	*COMMD5, ZNF517, ZNF250, RPL8, ZNF7, ZNF16, ARHGAP39, ZNF34, ZNF251*
TKHS190002648	NEC Cervix	DUP	chr8:144580846-145054157	*ZNF252P, TMED10P1, ZNF252P-AS1, COMMD5, ZNF517, ZNF250, RPL8, C8orf33, ZNF7, ZNF16, ARHGAP39, ZNF34, ZNF251*
TKHS190002653	NEC Ovary	DUP	chr8:144580846-145054157	*ZNF252P, TMED10P1, ZNF252P-AS1, COMMD5, ZNF517, ZNF250, RPL8, C8orf33, ZNF7, ZNF16, ARHGAP39, ZNF34, ZNF251*
TKHS190002648	NEC Cervix	DUP	chr8:17299862-20220402	*NAT2, LOC100128993, FGL1, PSD3, LPL, ASAH1, PCM1, PDGFRL, ATP6V1B2, INTS10, CSGALNACT1, MTUS1, SH2D4A, SLC7A2, SLC18A1, NAT1, MTMR7*
TKHS190002644	NEC Cervix	DEL	chr8:21702805-22211728	*NPM2, LGI3, DMTN, XPO7, GFRA2, HR, SFTPC, FAM160B2, BMP1, NUDT18, REEP4, FGF17, DOK2*
TKHS190002648	NEC Cervix	DUP	chr8:21702805-25410202	*LOC100507156, SORBS3, NPM2, ADAM28, NKX2-6, PEBP4, EGR3, R3HCC1, LGI3, DMTN, XPO7, RHOBTB2, SLC39A14, LOC254896, GFRA2, ADAMDEC1, LOC286059, LOC389641, LOXL2, MIR320A, NEFM, NEFL, NKX3-1, SLC25A37, C8orf58, PIWIL2, PPP2R2A, PPP3CC, HR, BIN3, CCAR2,*

				PDLIM2, SFTPC, FAM160B2, BMP1, POLR3D, STC1, NUDT18, DOCK5, BIN3-IT1, REEP4, ADAM7, TNFRSF10D, TNFRSF10C, TNFRSF10B, TNFRSF10A, FGF17, DOK2, CHMP7, ENTPD4, PHYHIP
TKHS190002643	NEC Cervix	DEL	chr8:22120342-22138793	*HR, REEP4*
TKHS190002653	NEC Ovary	DUP	chr8:22554507-22616248	*SORBS3, C8orf58, CCAR2, PDLIM2*
TKHS190002650	NEC Ovary	DEL	chr8:23005960-23197204	*RHOBTB2, LOC254896, LOC286059, TNFRSF10D, TNFRSF10C, TNFRSF10B, TNFRSF10A*
TKHS190002648	NEC Cervix	DUP	chr8:25861309-29263034	*MIR4287, MIR4288, MIR3622A, MIR3622B, PNMA2, CHRNA2, CLU, ADRA1A, ESCO2, FBXO16, DPYSL2, EPHX2, EXTL3, PTK2B, TRIM35, KIF13B, SCARA5, NUGGC, SCARA3, PNOC, ELP3, PPP2R2A, CCDC25, INTS9, PBK, ZNF395, EBF2, BNIP3L, HMBOX1, FZD3, STMN4*
TKHS190002644	NEC Cervix	DEL	chr8:26770149-27538692	*CHRNA2, ADRA1A, EPHX2, PTK2B, TRIM35, STMN4*
TKHS190002643	NEC Cervix	DEL	chr8:27239259-27525473	*CHRNA2, EPHX2, PTK2B, TRIM35, STMN4*
TKHS190002650	NEC Ovary	DEL	chr8:27397585-27544244	*CHRNA2, EPHX2, PTK2B*
TKHS190002648	NEC Cervix	DUP	chr8:30065935-30545175	*RBPMS-AS1, MIR548O2, DCTN6, RBPMS, LEPROTL1, SARAF, MBOAT4*
TKHS190002648	NEC Cervix	DUP	chr8:32548727-33597515	*NRG1, DUSP26, RNF122, TTI2, MAK16, FUT10*
TKHS190002648	NEC Cervix	DUP	chr8:3284144-4637558	*CSMD1*
TKHS190002648	NEC Cervix	DUP	chr8:37695980-39274350	*ERLIN2, PLPBP, GOT1L1, LETM2, ADRB3, EIF4EBP1, HTRA4, ADAM32, FGFR1, DDHD2, ADGRA2, LSM1, RNF5P1, C8orf86, NSD3, BRF2, PLEKHA2, STAR, TACC1, LOC728024, ZNF703, RAB11FIP1, TM2D2, PLPP5, ADAM9, ASH2L, BAG4*
TKHS190002648	NEC Cervix	DUP	chr8:39987871-40674931	*IDO2, TCIM, ZMAT4*
TKHS190002648	NEC Cervix	DUP	chr8:413080-3052647	*TDRP, ERICH1, CLN8, FBXO25, LOC286083, NA, CSMD1, MIR596, MYOM2, DLGAP2, ARHGEF10, KBTBD11*
TKHS190002648	NEC Cervix	DUP	chr8:41609655-42850997	*AP3M2, CHRNB3, SMIM19, GPAT4, NKX6-3, DKK4, ANK1, IKBKB, PLAT, POLB, THAP1, MIR486-1, SLC20A2, VDAC3, KAT6A, RNF170, CHRNA6*
TKHS190002648	NEC Cervix	DUP	chr8:47279862-49075247	*CEBPD, SPIDR, MCM4, PPDPFL, PRKDC, SNAI2, UBE2V2, EFCAB1*
TKHS190002643	NEC Cervix	DEL	chr8:47779004-47854214	*PRKDC*
TKHS190002644	NEC Cervix	DEL	chr8:52136590-52180312	*ST18*
TKHS190002648	NEC Cervix	DUP	chr8:52137421-52180312	*ST18*
TKHS190002648	NEC Cervix	DUP	chr8:54135284-56316347	*SBF1P1, XKR4, TMEM68, SDR16C5, SNORD54, MRPL15, LYN, MOS, PLAG1, RP1, RPS20, SOX17, CHCHD7, TGS1*
TKHS190002648	NEC Cervix	DUP	chr8:58585898-61607322	*LOC100130298, CLVS1, ASPH, CHD7, RAB2A, CA8, NSMAF, TOX*
TKHS190002648	NEC Cervix	DUP	chr8:65708976-66516076	*LINC00967, RRS1-AS1, ADHFE1, CRH, RRS1, VXN, PDE7A, TRIM55, DNAJC5B, MTFR1*
TKHS190002648	NEC Cervix	DUP	chr8:66612819-66841110	*C8orf44-SGK3, SGK3, PTTG3P, MYBL1, C8orf44, VCPIP1*
TKHS190002648	NEC Cervix	DUP	chr8:67056519-67251450	*ARFGEF1, COPS5, CSPP1*
TKHS190002648	NEC Cervix	DUP	chr8:6747670-7056697	*GS1-24F4.2, DEFA1, DEFA4, DEFA5, DEFA6, DEFB1, DEFT1P, XKR5, DEFA8P, DEFA9P, DEFA10P, AGPAT5, DEFA11P, DEFA1B*
TKHS190002648	NEC Cervix	DUP	chr8:68083240-72055600	*MSC-AS1, LINC01592, NCOA2, C8orf34, EYA1, SULF1, TRAM1, C8orf34-AS1, LACTB2-AS1, XKR9, LACTB2, PRDM14, PREX2, SLCO5A1, TRPA1, MSC*
TKHS190002648	NEC Cervix	DUP	chr8:74825250-75016705	*PI15, CRISPLD1*
TKHS190002648	NEC Cervix	DUP	chr8:80050445-80993227	*MIR5708, PAG1, ZNF704, ZBTB10, TPD52*
TKHS190002654	NEC Cervix	DEL	chr8:86468676-86626080	*RMDN1, CNGB3, CPNE3*
TKHS190002648	NEC Cervix	DUP	chr8:86486484-86579252	*RMDN1, CNGB3, CPNE3*
TKHS190002654	NEC Cervix	DEL	chr8:94372170-94512089	*FSBP, RAD54B, VIRMA*

TKHS190002648	NEC Cervix	DUP	chr8:9720374-9772422	*MIR597, TNKS*
TKHS190002648	NEC Cervix	DUP	chr8:99135015-99442635	*VPS13B*
TKHS190002648	NEC Cervix	DEL	chr9:115052740-115091018	*TNC*
TKHS190002643	NEC Cervix	DUP	chr9:121282452-121321401	*GSN*
TKHS190002648	NEC Cervix	DEL	chr9:121301963-121348149	*STOM, GSN*
TKHS190002644	NEC Cervix	DUP	chr9:121318406-121348149	*STOM, GSN*
TKHS190002648	NEC Cervix	DEL	chr9:122378433-122392544	*PTGS1*
TKHS190002643	NEC Cervix	DUP	chr9:127168679-127212719	*RALGPS1*
TKHS190002648	NEC Cervix	DEL	chr9:127168679-127218769	*RALGPS1*
TKHS190002648	NEC Cervix	DEL	chr9:127407112-127473931	*SNORA65, SLC2A8, RPL12, ZNF79, LRSAM1*
TKHS190002648	NEC Cervix	DEL	chr9:127707032-127896332	*SH2D3C, MIR3960, MIR4672, CDK9, PTRH1, TTC16, ENG, AK1, FPGS, TOR2A, CFAP157, ST6GALNAC6*
TKHS190002657	NEC Ovary	DUP	chr9:127808800-127824974	*ENG, FPGS*
TKHS190002648	NEC Cervix	DEL	chr9:128265788-128525423	*MIR219B, SLC27A4, TRUB2, GLE1, GOLGA2, SWI5, MIR219A2, ODF2, COQ4, CERCAM, URM1*
TKHS190002648	NEC Cervix	DEL	chr9:128604326-128684007	*SET, SPTAN1, WDR34*
TKHS190002648	NEC Cervix	DEL	chr9:128705734-128949243	*LOC100506100, ZER1, ENDOG, DOLK, NUP188, PHYHD1, PKN3, SPOUT1, TBC1D13, LRRC8A, ZDHHC12, KYAT1*
TKHS190002648	NEC Cervix	DEL	chr9:129098249-129108077	*CRAT*
TKHS190002648	NEC Cervix	DEL	chr9:129612347-129807363	*ASB6, TOR1B, NTMT1, C9orf50, PRRX2, PTGES*
TKHS190002648	NEC Cervix	DEL	chr9:129858050-129878440	*USP20*
TKHS190002650	NEC Ovary	DUP	chr9:129868005-129878440	*USP20*
TKHS190002648	NEC Cervix	DEL	*chr9:130407571-130422726*	
TKHS190002648	NEC Cervix	DEL	chr9:131518445-131650242	*POMT1, RAPGEF1, UCK1*
TKHS190002654	NEC Cervix	DUP	chr9:132166832-132241044	*NTNG2*
TKHS190002654	NEC Cervix	DUP	chr9:133514636-133644320	*DBH, MYMK, FAM163B, ADAMTSL2*
TKHS190002646	NEC Cervix	DEL	chr9:133568603-133734173	*DBH-AS1, DBH, SARDH, FAM163B, ADAMTSL2*
TKHS190002654	NEC Cervix	DUP	chr9:134690912-134796901	*COL5A1*
TKHS190002648	NEC Cervix	DEL	chr9:134883302-135522177	*PPP1R26-AS1, OLFM1, C9orf116, C9orf62, FCN1, FCN2, LCN1, LOC401557, MRPS2, PPP1R26*
TKHS190002648	NEC Cervix	DEL	chr9:135742772-136745914	*MIR4673, MIR4674, UBAC1, AGPAT2, ENTR1, NACC2, DIPK1B, CAMSAP1, C9orf163, LCN6, QSOX2, PMPCA, GPSM1, DKFZP434A062, MIR126, LCN10, NOTCH1, EGFL7, INPP5E, KCNT1, CARD9, SNAPC4, SNORA17A, SNORA17B, DNLZ, LHX3, SNHG7, TMEM250, SEC16A*
TKHS190002653	NEC Ovary	DEL	chr9:136899594-137051129	*ABCA2, FUT7, LCN12, PAXX, LCNL1, C9orf139, FBXW5, NPDC1, PTGDS, TRAF2, C8G, CLIC3, ENTPD2*
TKHS190002653	NEC Ovary	DEL	chr9:137158379-137233722	*MIR3621, SLC34A3, NDOR1, TPRN, GRIN1, ANAPC2, CYSRT1, LRRC26, RNF224, RNF208, SSNA1, TMEM203*
TKHS190002657	NEC Ovary	DUP	chr9:137167774-137175837	*MIR3621, GRIN1, ANAPC2, LRRC26*
TKHS190002657	NEC Ovary	DUP	chr9:137233004-137252525	*STPG3-AS1, TUBB4B, SLC34A3, FAM166A, STPG3*
TKHS190002648	NEC Cervix	DEL	chr9:137493013-137577603	*PNPLA7, MRPL41, DPH7*
TKHS190002648	NEC Cervix	DEL	chr9:137957595-138118081	*CACNA1B*

TKHS190002654	NEC Cervix	DEL	chr9:33441990-33466709	*AQP3, NOL6*
TKHS190002644	NEC Cervix	DUP	chr9:33441990-33467262	*AQP3, NOL6*
TKHS190002643	NEC Cervix	DUP	chr9:34636997-34648456	*SIGMAR1, GALT*
TKHS190002643	NEC Cervix	DUP	chr9:35074108-35079524	*FANCG*
TKHS190002643	NEC Cervix	DUP	chr9:35658576-35675567	*CCDC107, CA9, ARHGEF39*
TKHS190002643	NEC Cervix	DUP	chr9:35739628-35750741	*GBA2, RGP1*
TKHS190002654	NEC Cervix	DUP	chrX:103078560-103785768	*RAB40A, TMEM31, BEX3, TCEAL5, MORF4L2-AS1, GLRA4, TCEAL9, RAB9B, PLP1, NXF3, BEX4, TCEAL7, TCEAL4, BEX2, TCEAL3, TCEAL8, TCEAL1, MORF4L2*
TKHS190002648	NEC Cervix	DEL	chrX:152850265-153058544	*PNMA5, PNMA3, NSDHL, ZNF185*
TKHS190002657	NEC Ovary	DUP	chrX:152922720-153797814	*MIR718, MIR3202-2, MIR3202-1, BCAP31, PNCK, TEX28, DNASE1L1, EMD, ABCD1, FLNA, G6PD, OPN1MW, SRPK3, GDI1, SNORA70, HCFC1, IDH3G, IRAK1, L1CAM, ARHGAP4, MECP2, PLXNB3, ATP6AP1, AVPR2, PLXNA3, PDZD4, OPN1LW, RENBP, FAM3A, RPL10, SLC6A8, SSR4, TAZ, NAA10, UBL4A, TMEM187, LAGE3, SLC10A3, TKTL1, IKBKG, FAM50A*
TKHS190002646	NEC Cervix	DEL	chrX:153454394-154018787	*MIR3202-2, MIR3202-1, BCAP31, PNCK, DUSP9, ABCD1, SRPK3, HCFC1, IDH3G, IRAK1, L1CAM, ARHGAP4, ATP2B3, PLXNB3, AVPR2, HAUS7, PDZD4, RENBP, BGN, SLC6A8, SSR4, NAA10, TMEM187, CCNQ*
TKHS190002648	NEC Cervix	DEL	chrX:153457137-153580298	*ATP2B3, HAUS7, BGN*
TKHS190002654	NEC Cervix	DUP	chrX:153808342-153913051	*L1CAM, ARHGAP4, AVPR2, PDZD4*
TKHS190002648	NEC Cervix	DEL	chrX:153905532-154546163	*MIR718, MIR3202-2, MIR3202-1, TEX28, DNASE1L1, EMD, FLNA, G6PD, OPN1MW, GDI1, SNORA70, HCFC1, IRAK1, ARHGAP4, MECP2, ATP6AP1, AVPR2, PLXNA3, OPN1LW, RENBP, FAM3A, RPL10, TAZ, NAA10, UBL4A, TMEM187, LAGE3, SLC10A3, TKTL1, IKBKG, FAM50A*
TKHS190002657	NEC Ovary	DUP	chrX:153918832-154462310	*MIR718, MIR3202-2, MIR3202-1, TEX28, DNASE1L1, EMD, FLNA, OPN1MW, GDI1, SNORA70, HCFC1, IRAK1, ARHGAP4, MECP2, ATP6AP1, PLXNA3, OPN1LW, RENBP, RPL10, TAZ, NAA10, TMEM187, TKTL1, FAM50A*
TKHS190002641	NEC Ovary	DEL	chrX:154325339-154380019	*EMD, FLNA, TKTL1*
TKHS190002646	NEC Cervix	DEL	chrX:154325339-154463519	*DNASE1L1, EMD, FLNA, GDI1, SNORA70, ATP6AP1, PLXNA3, RPL10, TAZ, TKTL1, FAM50A*
TKHS190002643	NEC Cervix	DUP	chrX:154435139-154442428	*GDI1, ATP6AP1*
TKHS190002657	NEC Ovary	DUP	chrX:154468823-154546163	*G6PD, PLXNA3, FAM3A, UBL4A, LAGE3, SLC10A3, IKBKG*
TKHS190002654	NEC Cervix	DUP	chrX:15517929-15537305	*BMX*
TKHS190002654	NEC Cervix	DUP	chrX:40049916-40076962	*BCOR*
TKHS190002643	NEC Cervix	DUP	chrX:47199048-47241699	*CDK16, UBA1, USP11, INE1*
TKHS190002646	NEC Cervix	DEL	chrX:48559614-48706627	*TBC1D25, RBM3, WDR13, SUV39H1, WAS*
TKHS190002646	NEC Cervix	DEL	chrX:49065454-49193463	*WDR45, PRAF2, GPKOW, PRICKLE3, PLP2, SYP, MAGIX, CCDC120*
TKHS190002645	NEC Endometrium	DUP	chrX:50285164-50404184	*DGKK, CCNB3*
TKHS190002648	NEC Cervix	DEL	chrX:53193437-53215976	*KDM5C*
TKHS190002648	NEC Cervix	DEL	chrX:71223708-71290791	*GJB1, NONO, BCYRN1, ZMYM3*
TKHS190002650	NEC Ovary	DUP	chrX:71247734-71253255	*BCYRN1, ZMYM3*
TKHS190002654	NEC Cervix	DUP	chrX:72129831-72143574	*RTL5, NHSL2*
TKHS190002646	NEC Cervix	DEL	chrX:9894358-10474750	*CLDN34, CLCN4, SHROOM2, MID1, WWC3*
