# Supplementary material for: Genomic analyses of high‐grade neuroendocrine gynecological malignancies reveal a unique mutational landscape and therapeutic vulnerabilities
Source: Mol Oncol. 2021 Jul 22;15(12):3545–58. doi: 10.1002/1878-0261.13057 (PMC8637558; doi:10.1002/1878-0261.13057)
Supplement: Supplementary file 4 — Table S3. List of cancer driver genes detected based on positional clustering. [file MOL2-15-3545-s001.pdf]

Supplementary Table 3. List of cancer driver genes detected based on positional clustering

Gene	Total Mutations	Mutated Samples	Clusters	Mutations in clusters	Z-score	p-value	FDR	Fraction of mutations in clusters
CCDC6	13	13	1	13	5.54615385	1.46E-08	8.40E-08	1
CLTCL1	18	12	3	18	5.54615385	1.46E-08	8.40E-08	1
LATS2	15	12	2	15	5.54615385	1.46E-08	8.40E-08	1
RNF43	17	12	3	17	5.54615385	1.46E-08	8.40E-08	1
KMT2C	59	14	8	54	4.82907432	6.86E-07	3.15E-06	0.91525424
CDH11	10	9	2	9	4.77692308	8.90E-07	3.41E-06	0.9
PTPRT	16	12	2	14	4.58461539	2.27E-06	7.47E-06	0.875
BLM	7	7	2	6	4.44725275	4.35E-06	1.25E-05	0.85714286
NCOR2	34	14	2	26	4.23278084	1.15E-05	2.95E-05	0.76470588
BRCA1	5	5	2	4	4.00769231	3.07E-05	7.05E-05	0.8
KNL1	29	14	3	23	3.95464191	3.83E-05	8.01E-05	0.79310345
FAT1	14	11	3	11	3.8978022	4.85E-05	9.30E-05	0.78571429
BRCA2	15	9	3	11	3.4948718	0.00023715	0.00041956	0.73333333
TP63	8	4	2	5	3.34835165	0.00040647	0.00066777	0.625
CTCF	5	4	1	3	2.46923077	0.00677019	0.01038096	0.6
TET1	7	7	1	4	2.24945055	0.01224192	0.01759776	0.57142857
TP53	5	4	1	2	1.7	0.04456546	0.06029445	0.4
FAT4	16	12	1	7	1.21923077	0.11137831	0.14231673	0.4375
ASXL1	6	5	1	2	0.93076923	0.17598648	0.21303626	0.33333333
CIC	6	5	1	2	0.41794872	0.33799231	0.38869115	0.33333333
KMT2D	11	7	1	2	0.05164835	0.47940445	0.50119556	0.18181818
LRP1B	7	5	1	2	0.05164835	0.47940445	0.50119556	0.28571429
ZFHX3	10	8	1	3	-0.0094017	0.50375068	0.50375068	0.3
