# Supplementary material for: Genomic analyses of high‐grade neuroendocrine gynecological malignancies reveal a unique mutational landscape and therapeutic vulnerabilities
Source: Mol Oncol. 2021 Jul 22;15(12):3545–58. doi: 10.1002/1878-0261.13057 (PMC8637558; doi:10.1002/1878-0261.13057)
Supplement: Supplementary file 5 — Table S4. List of fusion genes. [file MOL2-15-3545-s005.pdf]

Supplementary Table 4. List of fusion genes

Fusion genes	%
MALAT1--SMG1	53.8
AD000090.1--MALAT1	53.8
AC098591.3--RLIM	46.2
AC099535.1--UBA52	38.5
EEF1A1--MALAT1	30.8
ACTB--MALAT1	30.8
ASH1L--YY1AP1	30.8
DYNC1H1--MALAT1	23.1
AC091807.1--IMPDH1	23.1
AC007621.1--UBE2V1	23.1
MALAT1--TPT1	23.1
XIST--MALAT1	23.1
MALAT1--XIST	23.1
MALAT1--RNF213	23.1
MALAT1--RMRP	23.1
MALAT1--FAT1	23.1
CDK6--MALAT1	23.1
AHNAK--MALAT1	23.1
AC091951.3--HERC2	23.1
ZNF480--ZNF665	15.4
MALAT1--ZNF638	15.4
MALAT1--VPS13A	15.4
MALAT1--TCF4	15.4
MALAT1--SPTBN1	15.4
MALAT1--RPSA	15.4
KAT6A--MALAT1	15.4
HNRNPU--MALAT1	15.4
DST--MALAT1	15.4
CR381653.1--ZNF717	15.4
CLTC--MALAT1	15.4
CCT3--MALAT1	15.4
AC093878.1--CAMSAP1	15.4
AC024270.1--FABP5	15.4
UBC--MALAT1	15.4
SET--LMOD3	15.4
RPPH1--ACTB	15.4
RANBP2--MALAT1	15.4
PARG--AC022400.4	15.4
NEAT1--XIST	15.4
NAP1L1--MALAT1	15.4
MROH1--MALAT1	15.4
MALAT1--VMP1	15.4
MALAT1--TTC3	15.4
MALAT1--TNRC6A	15.4
MALAT1--TAOK1	15.4
MALAT1--SYNE2	15.4
MALAT1--SREBF2	15.4
MALAT1--SMC3	15.4
MALAT1--ROCK1	15.4
MALAT1--RGPD2	15.4
MALAT1--RDX	15.4
MALAT1--RALGAPA1	15.4
MALAT1--POLR2A	15.4
MALAT1--PCM1	15.4
MALAT1--NKTR	15.4
MALAT1--KIAA1328	15.4
MALAT1--JAK2	15.4
MALAT1--JAK1	15.4
MALAT1--HNRNPU	15.4
MALAT1--HNRNPA3	15.4
MALAT1--HNRNPA2B1	15.4
MALAT1--EEF1A1	15.4
MALAT1--CP	15.4
MALAT1--COL1A2	15.4
MALAT1--CKAP5	15.4
MALAT1--CANX	15.4
MALAT1--BIRC6	15.4
MALAT1--ALK	15.4
MALAT1--ACTG1	15.4
MALAT1--ACIN1	15.4
LNPEP--MALAT1	15.4
LINC02241--C1QTNF3-AMACR	15.4
LINC01923--NDUFV1	15.4
IL9R--AL591424.1	15.4
HSP90AA1--MALAT1	15.4
HNRNPH1--MALAT1	15.4
FOXP1--MALAT1	15.4
EEF1A1--NEAT1	15.4
COL3A1--MALAT1	15.4
CNOT4--MALAT1	15.4
CANX--MALAT1	15.4
C1QTNF3-AMACR--LINC02241	15.4
BTN2A2--BTN2A3P	15.4
BRD4--MALAT1	15.4
BMS1P4-AGAP5--TIMM23B	15.4
AL591424.1--IL9R	15.4
AL589743.5--TOMM40	15.4
AL157871.3--NDUFB3	15.4
AC064847.1--ENO3	15.4
ZZEF1--FLNC	7.7
ZZEF1--ACP6	7.7
ZYG11A--KCNH7	7.7
ZSWIM4--INTS3	7.7
ZSCAN29--PHF3	7.7
ZSCAN29--FAM149A	7.7
ZRANB1--RPS14	7.7
ZRANB1--MUC4	7.7
ZPR1--DGKA	7.7
ZNRF3--RREB1	7.7
ZNF91--ZNF525	7.7
ZNF91--CBX5	7.7
ZNF850--AC010624.4	7.7
ZNF841--ZNF721	7.7
ZNF816-ZNF321P--AC023934.1	7.7
ZNF814--RSRP1	7.7
ZNF813--ELL2	7.7
ZNF8--DNM1L	7.7
ZNF790--TMEM176A	7.7
ZNF788P--RCAN3	7.7
ZNF782--OLA1	7.7
ZNF781--ZNF20	7.7
ZNF767P--LARS	7.7
ZNF766--ZNF415	7.7
ZNF763--ZNF442	7.7
ZNF761--ZNF808	7.7
ZNF737--NCOR1	7.7
ZNF721--ZNF716	7.7
ZNF721--TRAF7	7.7
ZNF721--MYH11	7.7
ZNF721--AC010332.2	7.7
ZNF714--YTHDF3	7.7
ZNF71--SIN3A	7.7
ZNF701--ZNF347	7.7
ZNF69--AC022415.2	7.7
ZNF682--PLEKHG5	7.7
ZNF680--ZNF471	7.7
ZNF664--TRMT12	7.7
ZNF664--IPO9	7.7
ZNF664--GCC2	7.7

ZNF66--ACAP2	7.7
ZNF658B--ZNF334	7.7
ZNF654--GNAS	7.7
ZNF644--NPM1	7.7
ZNF641--ASS1	7.7
ZNF638--ZFP30	7.7
ZNF638--TIMP2	7.7
ZNF629--TARS2	7.7
ZNF626--AC021451.1	7.7
ZNF625--C12ORF57	7.7
ZNF623--LCOR	7.7
ZNF609--HSP90B1	7.7
ZNF609--ADCY1	7.7
ZNF608--TMEM260	7.7
ZNF608--MALAT1	7.7
ZNF605--FN1	7.7
ZNF594--TOPORS	7.7
ZNF592--LINC-PINT	7.7
ZNF586--ZNF417	7.7
ZNF584--IFIH1	7.7
ZNF57--AGL	7.7
ZNF561--DNAH7	7.7
ZNF559--MALAT1	7.7
ZNF558--TCF7L1	7.7
ZNF551--ZNF530	7.7
ZNF551--RGP1	7.7
ZNF530--KCNMB3	7.7
ZNF526--PPP1R12B	7.7
ZNF518B--ARL6IP1	7.7
ZNF518A--RNF40	7.7
ZNF460--DNAJC7	7.7
ZNF438--ZBTB20	7.7
ZNF436--MALAT1	7.7
ZNF43--PYCR1	7.7
ZNF428--MIR100HG	7.7
ZNF426--ZFP90	7.7
ZNF426--SETX	7.7
ZNF41--XIST	7.7
ZNF397--ERGIC1	7.7
ZNF383--ZNF420	7.7
ZNF37A--COPS3	7.7
ZNF37A--COG4	7.7
ZNF33A--SYNE2	7.7
ZNF337--ZNF586	7.7
ZNF330--TTC3	7.7
ZNF326--FAM193A	7.7
ZNF281--STIL	7.7
ZNF277--PCM1	7.7
ZNF276--TSPYL5	7.7
ZNF266--ZNF30	7.7
ZNF24--ZNF852	7.7
ZNF236--SCAF8	7.7
ZNF234--CTCFL	7.7
ZNF224--PLSCR1	7.7
ZNF217--ANKS1A	7.7
ZNF208--ZNF267	7.7
ZNF208--HIPK2	7.7
ZNF180--MALAT1	7.7
ZNF169--VPS13C	7.7
ZNF154--ZNF530	7.7
ZNF148--WDR76	7.7
ZNF141--TMEM135	7.7
ZNF141--FXYD5	7.7
ZNF14--IGF2	7.7
ZNF14--HNMT	7.7
ZNF137P--ZNF83	7.7
ZNF121--ZNF473	7.7
ZMYND8--C17ORF64	7.7
ZMYND15--DNAH1	7.7
ZMYND11--MALAT1	7.7
ZMYM4--RBM5	7.7
ZMYM2--MAP4K4	7.7
ZMYM2--MALAT1	7.7
ZMPSTE24--AC087190.3	7.7
ZMIZ2--FBXO36-IT1	7.7
ZMIZ1--XIST	7.7
ZMIZ1--RMRP	7.7
ZMIZ1--LAMA1	7.7
ZMIZ1--CCDC14	7.7
ZKSCAN1--MALAT1	7.7
ZKSCAN1--ASCL1	7.7
ZKSCAN1--AGO1	7.7
ZIM2--SPTBN1	7.7
ZIC5--MIIP	7.7
ZHX2--TIMP2	7.7
ZFYVE16--UTRN	7.7
ZFP92--ZNF629	7.7
ZFP90--ZNF433	7.7
ZFP36L1--ATXN2	7.7
ZFP30--ZNF420	7.7
ZFAND6--TAF15	7.7
ZFAND5--USP47	7.7
ZEB2--IGH@	7.7
ZDHHC11B--ARID2	7.7
ZDBF2--CFAP410	7.7
ZCCHC8--COX7B	7.7
ZC3H7A--FOXD1	7.7
ZC3H14--PSME4	7.7
ZC3H14--CCT7	7.7
ZC3H13--RPS29	7.7
ZC3H13--FOCAD	7.7
ZBTB8OS--TCP11L1	7.7
ZBTB48--MALAT1	7.7
ZBTB26--METTL8	7.7
ZBTB21--DCAF10	7.7
ZBTB20--COL3A1	7.7
ZBTB12BP--ZBTB12	7.7
ZBTB1--DNAH5	7.7
ZBED9--SEL1L	7.7
ZBED6--ZSCAN31	7.7
ZBED6--HIST1H1D	7.7
ZBED6--ERCC5	7.7
Z83843.1--PEG3	7.7
YY1AP1--COL6A2	7.7
YWHAZ--SPTAN1	7.7
YWHAZ--DST	7.7
YWHAG--TMSB4X	7.7
YWHAE--UBR4	7.7
YWHAE--DIDO1	7.7
YWHAE--CFAP44	7.7
YWHAE--ALMS1	7.7
YTHDF3--PISD	7.7
YTHDF3--ITGA9	7.7
YTHDC1--PLXNA3	7.7
YTHDC1--MUC4	7.7
YTHDC1--GOLGB1	7.7
YME1L1--TRAF6	7.7
YME1L1--TBC1D32	7.7
YME1L1--KRAS	7.7
YLPM1--YTHDF3	7.7
YIPF6--STARD10	7.7
YIPF5--HSPD1	7.7
YES1--SURF4	7.7
YES1--DOCK1	7.7
YBX1--GNLY	7.7

YAP1--ZMYM2	7.7
YAP1--AC004951.1	7.7
YAE1--DYNC1LI2	7.7
XRN2--TLN1	7.7
XRN2--MALAT1	7.7
XRCC6--SPATA13	7.7
XPOT--KAT6A	7.7
XPO5--CD63	7.7
XPO5--CALR	7.7
XPO5--ACTB	7.7
XPO1--SPAST	7.7
XPNPEP1--ADRM1	7.7
XIST--ZNF652	7.7
XIST--TP73-AS1	7.7
XIST--SECISBP2	7.7
XIST--PLXNB2	7.7
XIST--PLCG2	7.7
XIST--HMGN1	7.7
XIST--G6PD	7.7
XIST--EPS15	7.7
XIST--EEF1A1	7.7
XIST--CDC42	7.7
XIST--CCT3	7.7
XIST--AC004922.1	7.7
XIAP--EIF3J-DT	7.7
XIAP--BLOC1S5-TXNDC5	7.7
WWTR1--B2M	7.7
WWOX--ZNF267	7.7
WWOX--PSMA1	7.7
WWOX--FN1	7.7
WWOX--ACTG1	7.7
WWC3--SUN2	7.7
WTAP--ACTG1	7.7
WSB2--CACNA2D1	7.7
WRAP73--SKIL	7.7
WNK1--SMARCA2	7.7
WNK1--GTF2I	7.7
WIF1--CPLANE1	7.7
WDSUB1--AKR1B10	7.7
WDR82--PARP3	7.7
WDR74--UTRN	7.7
WDR74--ROCK1	7.7
WDR74--MUC4	7.7
WDR74--HNRNPUL2-BSCL2	7.7

WDR74--FTL	7.7
WDR74--EGR1	7.7
WDR74--DNAJC10	7.7
WDR74--DDX5	7.7
WDR74--AP005263.1	7.7
WDR74--AEBP1	7.7
WDR72--CCDC162P	7.7
WDR64--MIR100HG	7.7
WDR6--KLHL11	7.7
WDR6--GNAS	7.7
WDR6--CNTNAP1	7.7
WDR6--A4GNT	7.7
WDR45B--SILC1	7.7
WDR33--PHLDA1	7.7
WDR26--FOXJ3	7.7
WDR12--HAGHL	7.7
WDFY3--UBE3A	7.7
WDFY3--POLR3F	7.7
WASHC4--GGNBP2	7.7
WAPL--KLF3	7.7
WAC-AS1--MALAT1	7.7
VWF--LONP2	7.7
VWF--BCL2L11	7.7
VWA8--TIMM8A	7.7
VTI1B--GPR157	7.7
VSNL1--PAFAH1B1	7.7
VPS45--PHRF1	7.7
VPS39--GOLGA8R	7.7
VPS37A--NEB	7.7
VPS13C--EEF1A1	7.7
VPS13C--DDX27	7.7
VPS13A--TLE4	7.7
VPS13A--SPDL1	7.7
VPS13A--KIF2A	7.7
VPS13A--FAM193A	7.7
VMP1--CANX	7.7
VMP1--ASXL1	7.7
VMO1--NEB	7.7
VMA21--DNMT1	7.7
VKORC1L1--ARID1B	7.7
VIRMA--ZNF558	7.7
VIM--TMEM38B	7.7
VIM--POGZ	7.7
VIM--PIK3R2	7.7
VIM--MALAT1	7.7

VIM--KMT2C	7.7
VIM--EIF4A1	7.7
VIM--ANP32B	7.7
VIM--AD000090.1	7.7
VIL1--RBM33	7.7
VIL1--MALAT1	7.7
VIL1--CCT5	7.7
VEZT--GNA13	7.7
VCPIP1--SYNJ2BP-COX16	7.7
VCPIP1--KIAA0895	7.7
VCP--LMBR1	7.7
VCP--CCDC47	7.7
VCL--PDGFRA	7.7
VCAN--TAF15	7.7
VCAN--NR4A3	7.7
VAMP3--RAB11FIP5	7.7
VAMP3--CYP17A1	7.7
UTRN--MALAT1	7.7
UTRN--COL1A1	7.7
UTRN--ALKBH7	7.7
USP9X--MAPK8IP3	7.7
USP9X--IBTK	7.7
USP9X--GPR155	7.7
USP8--TMOD3	7.7
USP7--C2CD3	7.7
USP53--UBA52	7.7
USP40--MALAT1	7.7
USP37--ZSWIM5	7.7
USP37--FNDC3A	7.7
USP34--SOS1	7.7
USP34--SMG1	7.7
USP34--NBAS	7.7
USP34--GIGYF1	7.7
USP34--ARID1B	7.7
USP28--YLPM1	7.7
USP28--PTMA	7.7
USP24--GUSB	7.7
USP20--SERPINB6	7.7
USP15--RANBP3	7.7
USP1--PPIA	7.7
USH1C--TRA@	7.7
USF3--FNDC3B	7.7
URI1--PHF14	7.7
UQCRFS1--KHDC4	7.7

UQCRB--TRA@	7.7
UQCRB--PODXL	7.7
UQCC2--MALAT1	7.7
UNG--NAPA	7.7
UNG--DYM	7.7
UNC80--PPP3CB	7.7
UNC80--MALAT1	7.7
UMPS--DISP2	7.7
ULK4--MRAS	7.7
ULK1--ZNF292	7.7
UGT2B15--XIST	7.7
UGDH--DST	7.7
UFM1--CLCN7	7.7
UFC1--HSPD1	7.7
UCP2--AL021155.5	7.7
UCHL5--AL390957.1	7.7
UBXN4--VPS13A	7.7
UBR4--SYMPK	7.7
UBR4--RPS11	7.7
UBR4--RPL26	7.7
UBR4--MALAT1	7.7
UBR4--CHD2	7.7
UBR3--PANK2	7.7
UBR3--CPLANE1	7.7
UBR2--DDX17	7.7
UBIAD1--STK24	7.7
UBE4A--ENPP4	7.7
UBE3A--RANBP2	7.7
UBE3A--AC068580.4	7.7
UBE2Z--MALAT1	7.7
UBE2V1--AC007621.1	7.7
UBE2Q2--PTPRD	7.7
UBE2D3--PHKG2	7.7
UBC--ZZEF1	7.7
UBC--ZFPM1	7.7
UBC--VIM	7.7
UBC--SLC25A3	7.7
UBC--PRRC2B	7.7
UBC--MARS	7.7
UBC--LMAN1	7.7
UBC--FAM214A	7.7
UBC--CIITA	7.7
UBC--CAPZB	7.7
UBC--BAZ2B	7.7
UBC--ADAMTS12	7.7

UBB--SYNPO2	7.7
UBAP1--PLEKHM3	7.7
UBAC1--ZFP36	7.7
UBA52--PDPR	7.7
UBA52--AC099535.1	7.7
UBA3--NEB	7.7
UBA2--LIFR	7.7
UBA1--LRBA	7.7
UBA1--BACH1	7.7
UAP1--PRPF4B	7.7
TYK2--NEFH	7.7
TXNRD1--NF1	7.7
TXNRD1--MYCBP2	7.7
TXNRD1--IFT46	7.7
TXNRD1--FTH1	7.7
TXNL1--OARD1	7.7
TXNIP--MUC4	7.7
TXNIP--IGK@	7.7
TXN--MALAT1	7.7
TXLNA--PRKAG1	7.7
TWSG1--HSPD1	7.7
TUT7--VARS	7.7
TUT4--ADCY9	7.7
TULP3--UNC13B	7.7
TUBGCP4--SLC44A1	7.7
TUBB6--CDV3	7.7
TUBA1C--SMC4	7.7
TUBA1B--CHST11	7.7
TTYH3--IGF2BP1	7.7
TTN--TRA@	7.7
TTN--TCF4	7.7
TTN--SMC2	7.7
TTN--RALY	7.7
TTI2--MIR100HG	7.7
TTF2--FMNL3	7.7
TTC3--RMRP	7.7
TTC28--ULK4	7.7
TTC21B--EPAS1	7.7
TTC14--INSR	7.7
TSPYL1--MAPK8IP3	7.7
TSPAN14--ERCC1	7.7
TSN--VPS13B	7.7
TSIX--PHC1	7.7
TSHZ2--INF2	7.7
TSG101--ATG16L2	7.7

TSEN34--PLCE1	7.7
TSC22D2--XIST	7.7
TSC2--RPL31	7.7
TRPV4--ARHGAP33	7.7
TRPV1--WIZ	7.7
TRNAU1AP--CALR	7.7
TRIT1--VPS13D	7.7
TRIP12--IL13RA1	7.7
TRIP12--FYCO1	7.7
TRIP12--EIF3B	7.7
TRIOBP--UBA1	7.7
TRIOBP--MALAT1	7.7
TRIOBP--IGF1R	7.7
TRIOBP--ARHGAP35	7.7
TRIO--CDKAL1	7.7
TRIO--ATXN2L	7.7
TRIO--AP000295.1	7.7
TRIM52--ARIH2	7.7
TRIM44--ZNF607	7.7
TRIM25--EML4	7.7
TRIM2--FTH1	7.7
TRIM13--ARL3	7.7
TRIL--FN1	7.7
TRIB3--JAG1	7.7
TRG@--DNAH14	7.7
TRAPPC9--GABBR1	7.7
TRAPPC8--RALGAPA1	7.7
TRAPPC8--PPIP5K1	7.7
TRAPPC3--GTF2F1	7.7
TRAPPC10--TCF12	7.7
TRAP1--STT3B	7.7
TRANK1--INO80B-WBP1	7.7
TRAM1--HDAC7	7.7
TRAK2--SLC25A44	7.7
TRAK1--RAD54L	7.7
TRAK1--CSF1R	7.7
TRAIP--SPTAN1	7.7
TRAF3--TRPC4AP	7.7
TRA@--SYNE2	7.7
TRA@--GOSR1	7.7
TRA@--ANO10	7.7
TRA2B--C17ORF113	7.7
TRA2A--MALAT1	7.7
TPT1--PCF11	7.7

TPT1--MCPH1	7.7
TPT1--AAK1	7.7
TPR--MYH9	7.7
TPR--CKAP5	7.7
TPP2--HTT	7.7
TPP2--EHD2	7.7
TPP2--AGL	7.7
TPM3--MALAT1	7.7
TPM3--CCAR1	7.7
TPM2--TAOK1	7.7
TPM2--RCHY1	7.7
TPM1--WDR60	7.7
TPM1--QSER1	7.7
TPI1--TPMT	7.7
TPI1--RMRP	7.7
TPI1--CPD	7.7
TPI1--ANKRD6	7.7
TPGS2--ACTN1	7.7
TPD52L2--VCP	7.7
TPCN1--EDN2	7.7
TP53BP1--DOCK7	7.7
TOR1AIP1--TTBK2	7.7
TOP2B--CELSR3	7.7
TOP2A--WWOX	7.7
TOP1--P2RY11	7.7
TOP1--MYH15	7.7
TOLLIP--AC021087.5	7.7
TOB1--OSGIN1	7.7
TNS3--AKR1C4	7.7
TNS1--WDR70	7.7
TNS1--PDE5A	7.7
TNRC6C--PRSS35	7.7
TNRC6C--MUC4	7.7
TNRC6B--SCP2	7.7
TNRC6B--MALAT1	7.7
TNRC6A--FOXA2	7.7
TNRC6A--ACTR2	7.7
TNRC18--FGD5	7.7
TNPO2--MALAT1	7.7
TNPO1--STAT1	7.7
TNKS2--SCARNA5	7.7
TNKS2--HERC2	7.7
TNK2--MAGI2	7.7
TNIP1--ZMYM4	7.7
TNFRSF21--HIST1H1B	7.7

TNFRSF14--SDAD1	7.7
TNC--NUMA1	7.7
TMTC3--ADCY2	7.7
TMPO--COL1A1	7.7
TMEM59--GANC	7.7
TMEM43--PPP1R15B	7.7
TMEM38B--YME1L1	7.7
TMEM38B--ACTG1	7.7
TMEM259--ARF4	7.7
TMEM229B--RPGRIP1L	7.7
TMEM209--ZNF699	7.7
TMEM184C--MALAT1	7.7
TMEM18--TNKS1BP1	7.7
TMEM176B--COMTD1	7.7
TMEM165--HSPB1	7.7
TMEM161B-AS1--VPS13A	7.7
TMEM131--AC021087.5	7.7
TMEM117--PCLO	7.7
TMEM108--RAB10	7.7
TMEM107--FBXO31	7.7
TMEM107--ARHGAP28	7.7
TMED10--H19	7.7
TMCO6--CTNNAL1	7.7
TMCO4--KAT6A	7.7
TMCO1--IVNS1ABP	7.7
TM9SF3--MALAT1	7.7
TM9SF2--LRIG2	7.7
TM9SF2--CCDC80	7.7
TLN1--CACNA1A	7.7
TLE5--POLR2A	7.7
TLE5--MALAT1	7.7
TLE3--CTSK	7.7
TLCD4-RWDD3--STAB1	7.7
TLCD4--STAB1	7.7
TJAP1--LMF1	7.7
TIRAP--KLF6	7.7
TIMM23B--BMS1P4-AGAP5	7.7
TIMM23B--AC022400.4	7.7
TICRR--PFDN5	7.7
TICRR--PABPC1	7.7
THUMPD3--HTATSF1	7.7
THRAP3--MALAT1	7.7
THOC7--SAT1	7.7
THOC5--MUC16	7.7

THOC1--SGK1	7.7
THBS2--AL445685.3	7.7
THAP6--AC006978.1	7.7
THAP5--WDR45B	7.7
TGIF2-RAB5IF--ZNF672	7.7
TGIF1--MAK16	7.7
TGIF1--FTL	7.7
TGFBR1--PGK1	7.7
TFG--PHACTR4	7.7
TF--MALAT1	7.7
TF--ADGRV1	7.7
TEX261--EIF3A	7.7
TEX2--CCDC6	7.7
TET2--ZNF117	7.7
TET2--TRAPPC10	7.7
TES--TRPC4AP	7.7
TENT5A--EIF2AK1	7.7
TENT4B--FTH1	7.7
TENT4A--OSGEP	7.7
TEAD2--PABPC1	7.7
TDRD3--CSPP1	7.7
TDRD3--ACTG1	7.7
TCTN1--WDR70	7.7
TCP1--LBH	7.7
TCF7--C3ORF62	7.7
TCF4--WBP4	7.7
TCF4--SMG1	7.7
TCF4--ITSN2	7.7
TCF25--PRPF8	7.7
TCEAL4--HDAC7	7.7
TBX2--RB1	7.7
TBX15--EPRS	7.7
TBL1X--PRRC2C	7.7
TBCE--ATP5MC2	7.7
TBCA--DDX5	7.7
TBC1D9B--SQSTM1	7.7
TBC1D9B--MALAT1	7.7
TBC1D9B--ETV6	7.7
TBC1D4--MALAT1	7.7
TBC1D32--LUCAT1	7.7
TASP1--NCOR2	7.7
TASOR2--WRNIP1	7.7
TAS2R63P--TAS2R30	7.7
TARS--TCF3	7.7
TARDBP--KIAA1109	7.7

TAOK1--RHEB	7.7
TAOK1--COL1A1	7.7
TANGO6--MTCH2	7.7
TANC2--NHLRC3	7.7
TANC2--HSPG2	7.7
TAF1D--AL033519.5	7.7
TAF15--VPS13A	7.7
TAF15--SF1	7.7
TAF11--WNK1	7.7
TAF1--UBAP1	7.7
TAB3--MKNK2	7.7
SYTL4--MBIP	7.7
SYT14--INTS10	7.7
SYNRG--CHCHD10	7.7
SYNPO--EIF4A2	7.7
SYNJ2--AL358113.1	7.7
SYNE3--DNAH14	7.7
SYNE2--MALAT1	7.7
SYNE2--LGALS3BP	7.7
SYNE2--HIST1H2AG	7.7
SYNE2--FAM168A	7.7
SYNE1--FTL	7.7
SYNE1--ATP5F1A	7.7
SYCP2--PTPRB	7.7
SWAP70--CDK13	7.7
SUZ12--TRB@	7.7
SUZ12--NF1	7.7
SUZ12--ASCL1	7.7
SUPT6H--RBL2	7.7
SUMO3--NUP153	7.7
SUMO2--IL6ST	7.7
SULF1--ADCY4	7.7
SUFU--ADGRD1	7.7
STXBP5--SHPRH	7.7
STXBP5--LYST	7.7
STX7--XIST	7.7
STX3--SETBP1	7.7
STRN4--SON	7.7
STRADA--ATP2B1	7.7
STN1--ZNF292	7.7
STMN1--USP7	7.7
STMN1--ARHGAP22	7.7
STK4--WWOX	7.7
STK4--CTDSP1	7.7
STK38--RBBP8	7.7

STK36--ZMYM2	7.7
STK32B--UBC	7.7
STK24--EPRS	7.7
STK24--C9ORF85	7.7
STIL--BDP1	7.7
STAT5B--NPEPPS	7.7
STAT1--PEX6	7.7
STAT1--NEAT1	7.7
STAT1--ANP32B	7.7
STARD7--ZNF300	7.7
STARD4--CD46	7.7
STARD3--GEN1	7.7
STARD10--STAG1	7.7
STAMBP--LONP2	7.7
STAG3L5P-PVRIG2P-PILRB--JAM3	7.7
STAG3--GTF2IRD2B	7.7
STAG3--C19MC	7.7
ST8SIA4--MOV10	7.7
ST5--SBF2	7.7
ST3GAL6--SLC16A1-AS1	7.7
ST18--USP34	7.7
ST13--XPNPEP1	7.7
SSR1--AKR1C4	7.7
SSBP3--UBC	7.7
SSBP3--SYBU	7.7
SSBP1--PRRC1	7.7
SSBP1--MGAM	7.7
SRSF9--PDCD4	7.7
SRSF4--MESD	7.7
SRSF4--MALAT1	7.7
SRSF3--RARS2	7.7
SRSF2--CLCN7	7.7
SRSF11--CTCF	7.7
SRSF10--ZNF528	7.7
SRRT--RMRP	7.7
SRRM2--TRMT11	7.7
SRRM2--SLC29A2	7.7
SRRM2--HUWE1	7.7
SRRM2--HGSNAT	7.7
SRRM2--GTF2I	7.7
SRRM2--GAPDH	7.7
SRRM2--COL18A1	7.7
SRRM2--ATM	7.7
SRPRA--FANCI	7.7

SRP72--YTHDF2	7.7
SRGAP1--SH3BP2	7.7
SRC--UNC119	7.7
SQSTM1--PCNT	7.7
SPTBN1--SLC26A8	7.7
SPTBN1--NUP214	7.7
SPTBN1--HELZ2	7.7
SPTBN1--FKTN	7.7
SPTAN1--STXBP5	7.7
SPTAN1--RAB11FIP1	7.7
SPTAN1--CARM1	7.7
SPPL3--XBP1	7.7
SPPL2A--NBPF9	7.7
SPIN4--ALCAM	7.7
SPIN3--GTF2I	7.7
SPICE1--TTC17	7.7
SPG7--EIF4B	7.7
SPG7--COL6A2	7.7
SPEF2--SRP72	7.7
SPECC1L-ADORA2A--PPWD1	7.7
SPECC1L-ADORA2A--ADA2	7.7
SPECC1L--PPWD1	7.7
SPC24--IL20RB	7.7
SPATS2L--HYMAI	7.7
SPATS2--PDGFRL	7.7
SPART--PTPRC	7.7
SPART--GRHL2	7.7
SPARC--SPCS1	7.7
SPARC--NACA4P	7.7
SPARC--EHBP1	7.7
SPAG9--RPL10	7.7
SP3--OLA1	7.7
SP140L--FBRSL1	7.7
SP100--ZNF91	7.7
SP1--XRCC5	7.7
SP1--KARS	7.7
SOX4--RFX6	7.7
SOX4--B3GALNT2	7.7
SOX18--WDR74	7.7
SOS2--WDR36	7.7
SOS1--TGFBR1	7.7
SORL1--MALAT1	7.7
SORCS1--A2M	7.7
SON--SSR1	7.7

SON--PFKL	7.7
SON--EEF1B2	7.7
SOD3--CACNA1H	7.7
SNX9--UQCRB	7.7
SNX6--NCL	7.7
SNX30--HADHB	7.7
SNX27--MALAT1	7.7
SNX25--TPR	7.7
SNX21--ITFG1	7.7
SNX13--DELE1	7.7
SNU13--C12ORF4	7.7
SNU13--AC091060.1	7.7
SNTB1--AC253536.7	7.7
SNRPN--SLC30A9	7.7
SNRNP27--CHSY1	7.7
SNRNP200--MALAT1	7.7
SMURF2--NCAPG2	7.7
SMTN--TANK	7.7
SMPDL3A--PTS	7.7
SMIM7--MALAT1	7.7
SMG1--SNX1	7.7
SMG1--SLC39A10	7.7
SMG1--RSL24D1	7.7
SMG1--PIKFYVE	7.7
SMG1--GTF2I	7.7
SMG1--GSN	7.7
SMG1--DSP	7.7
SMG1--CCL3	7.7
SMG1--CACNA2D1	7.7
SMG1--C2CD5	7.7
SMCHD1--PARG	7.7
SMC6--ZBTB22	7.7
SMC4--SNU13	7.7
SMC4--MUC4	7.7
SMC4--KRCC1	7.7
SMC3--PDCD4	7.7
SMC2--DNAH14	7.7
SMC1A--MDN1	7.7
SMARCC2--RPPH1	7.7
SMARCC2--CLCN2	7.7
SMARCC2--AKR1C3	7.7
SMARCC1--UBC	7.7
SMARCC1--BRWD1	7.7
SMARCA5--MALAT1	7.7
SMARCA4--ZFP36L1	7.7

SMARCA4--RELL1	7.7
SMARCA4--AL031777.3	7.7
SMARCA2--HNRNPA3	7.7
SMARCA1--MALAT1	7.7
SMARCA1--EEF1B2	7.7
SMAD4--SH3GLB2	7.7
SMAD3--AC007114.2	7.7
SMAD1--SPTBN1	7.7
SLTM--RNF141	7.7
SLTM--CNOT8	7.7
SLTM--AC107029.2	7.7
SLMAP--TSC2	7.7
SLC9A7--HYOU1	7.7
SLC9A3R1--SPEN	7.7
SLC8A1--RBM25	7.7
SLC7A6--CNTN3	7.7
SLC7A11--METTL7B	7.7
SLC6A9--SREBF1	7.7
SLC6A15--RASSF3	7.7
SLC4A7--TBK1	7.7
SLC4A7--ANKRD17	7.7
SLC40A1--MLYCD	7.7
SLC40A1--FUBP1	7.7
SLC39A5--FAM234A	7.7
SLC39A10--AC092943.1	7.7
SLC38A9--NUP153	7.7
SLC35F5--MALAT1	7.7
SLC25A46--RMRP	7.7
SLC25A40--GTF3C1	7.7
SLC25A4--VAT1	7.7
SLC25A3--TM9SF2	7.7
SLC25A3--TAPT1	7.7
SLC25A3--ASAP1	7.7
SLC25A16--ATP13A1	7.7
SLC25A13--TENM4	7.7
SLC24A3--LUC7L2	7.7
SLC24A1--MIR4435-2HG	7.7
SLC1A5--ZC3H13	7.7
SLC1A3--EHF	7.7
SLC19A2--PRKDC	7.7
SLC16A1--IGKC	7.7
SLC16A1--IGK@	7.7
SLC16A1--ADGRL1	7.7
SLC12A6--ZEB2	7.7

SLC12A2--IGSF10	7.7
SLC12A2--GOLGA2	7.7
SIPA1L3--LRRN4	7.7
SIPA1L2--TUG1	7.7
SIPA1L1--KLB	7.7
SIK3--MALAT1	7.7
SIDT2--CFL2	7.7
SIAE--DHX9	7.7
SHTN1--RMND5A	7.7
SHROOM4--PPP1R3F	7.7
SHROOM4--NFAT5	7.7
SHROOM3--MALAT1	7.7
SHROOM3--ACTB	7.7
SHPRH--KCNB2	7.7
SHLD2--RANGAP1	7.7
SHLD2--FN1	7.7
SHISA6--PPFIBP2	7.7
SHISA5--MALAT1	7.7
SHISA4--IGF1R	7.7
SHISA2--SPPL2B	7.7
SHC1--GRIK5	7.7
SH3PXD2A--EDIL3	7.7
SH3GLB1--VCAN	7.7
SH3GLB1--E2F1	7.7
SH2D3C--KTN1	7.7
SGSM2--FBXO41	7.7
SFSWAP--RYBP	7.7
SFSWAP--FSIP2	7.7
SFPQ--TPM1	7.7
SFPQ--RBIS	7.7
SFPQ--PRPF6	7.7
SF3B2--CEP350	7.7
SF3B2--ACIN1	7.7
SF3B1--ZNF362	7.7
SF3B1--CELSR1	7.7
SETX--RAC1	7.7
SETDB1--MALAT1	7.7
SETD5--CSGALNACT2	7.7
SETBP1--WDR74	7.7
SET--PRDM2	7.7
SET--MALAT1	7.7
SET--IPO5	7.7
SET--AC019155.1	7.7
SESTD1--CIC	7.7
SESN3--SIK2	7.7

SERPINE2--ATIC	7.7
SERPINA1--MAN1A2	7.7
SERPINA1--EEF2	7.7
SERP1--TMSB4X	7.7
SERP1--ERGIC3	7.7
SERBP1--NT5C	7.7
SEPTIN2--CNOT3	7.7
SEPTIN2--BCOR	7.7
SEPTIN11--KDM4B	7.7
SEPTIN11--FTH1	7.7
SEPHS2--UBQLN2	7.7
SENP6--B4GALT5	7.7
SEMA6A--MYO1E	7.7
SEMA6A--CALCA	7.7
SEMA3C--TNRC6B	7.7
SEMA3C--SMG6	7.7
SELENON--ATRX	7.7
SELENOF--EPS15L1	7.7
SEC63--WWOX	7.7
SEC63--RTN1	7.7
SEC63--BANK1	7.7
SEC61A1--CPT2	7.7
SEC31A--MGAT4A	7.7
SEC31A--COPB2	7.7
SEC24D--MASTL	7.7
SEC23B--PAPOLA	7.7
SEC23B--CPLX2	7.7
SEC23A--ACTA1	7.7
SEC14L1--CASP3	7.7
SEC13--SNX33	7.7
SDHC--IGFN1	7.7
SDF4--RIMS2	7.7
SDF4--REV1	7.7
SCYL1--GTF3C5	7.7
SCN9A--MALAT1	7.7
SCN8A--ILRUN	7.7
SCG3--NAA15	7.7
SCG2--RMRP	7.7
SCG2--NEAT1	7.7
SCFD1--NOP53	7.7
SCD5--AC027097.2	7.7
SCD--FGFR1	7.7
SCD--APOC3	7.7
SCARNA9--ZNF676	7.7
SCARNA7--NPAS2	7.7

SCARNA7--EXOSC10	7.7
SCARNA5--SLC22A17	7.7
SCARNA5--NEAT1	7.7
SCARNA5--AMD1	7.7
SCARNA2--LIMA1	7.7
SCAMP1--CCDC162P	7.7
SCAI--SRRM2	7.7
SCAF4--RALGAPB	7.7
SCAF11--AC117386.2	7.7
SBNO1--NUCB1	7.7
SAT1--RACGAP1	7.7
SAT1--MED8	7.7
SAT1--CREB1	7.7
SASS6--LAMB1	7.7
SART3--SMOC2	7.7
SAP130--B2M	7.7
SAMM50--FAM104B	7.7
SAFB2--PTK2	7.7
SACS--NEAT1	7.7
SACS--MLLT6	7.7
S100A6--STARD13	7.7
S100A11--CAP1	7.7
RUNX1--FAM111B	7.7
RUFY3--NAGA	7.7
RUBCN--FYTTD1	7.7
RTTN--CCN2	7.7
RTN3--H19	7.7
RTL6--C16ORF96	7.7
RTKN--ZNF347	7.7
RTF1--SLC3A2	7.7
RTEL1-TNFRSF6B--TTC3	7.7
RSRC2--MALAT1	7.7
RSL24D1--GJA1	7.7
RSF1--TMED10	7.7
RSF1--KIAA1109	7.7
RSAD2--NEB	7.7
RRP8--MALAT1	7.7
RRP36--MIR205HG	7.7
RRP15--CRKL	7.7
RRBP1--SMARCA1	7.7
RRBP1--MYDGF	7.7
RRBP1--LCOR	7.7
RRAS--LMAN1	7.7
RPS6KB1--MALAT1	7.7

RPS6KA3--HSP90AA1	7.7
RPS6--PAX5	7.7
RPS4X--RGS3	7.7
RPS3--UQCRC2	7.7
RPS29--MALAT1	7.7
RPS27A--PITPNM3	7.7
RPS24--RMRP	7.7
RPS20--FTSJ3	7.7
RPS18--PTPN21	7.7
RPS17--TBL1XR1	7.7
RPS17--MALAT1	7.7
RPS15--PRPF31	7.7
RPPH1--ZBTB16	7.7
RPPH1--TRAPPC1	7.7
RPPH1--SEPTIN9	7.7
RPPH1--PRKAR1A	7.7
RPPH1--OGDH	7.7
RPPH1--MYH10	7.7
RPPH1--MUC4	7.7
RPPH1--LPXN	7.7
RPPH1--FTL	7.7
RPPH1--DST	7.7
RPPH1--CNOT1	7.7
RPPH1--CEP350	7.7
RPPH1--ARMC8	7.7
RPPH1--AP001267.5	7.7
RPPH1--AC138409.2	7.7
RPN2--HAUS6	7.7
RPN1--MKI67	7.7
RPLP0--MALAT1	7.7
RPL8--WDR74	7.7
RPL8--SOGA1	7.7
RPL5--FMNL2	7.7
RPL38--RMRP	7.7
RPL37A--MALAT1	7.7
RPL37--CRABP2	7.7
RPL35A--YRDC	7.7
RPL34--FAM91A1	7.7
RPL32--MKI67	7.7
RPL32--CD24	7.7
RPL31--IGK@	7.7
RPL31--FTL	7.7
RPL27A--MALAT1	7.7
RPL24--TTC37	7.7
RPL23AP82--DHX9	7.7

RPL23--APEH	7.7
RPL23--AC013549.4	7.7
RPL17--DOCK6	7.7
RPL13A--UBN2	7.7
RPL13A--ANKRD17	7.7
RPL13--ELN	7.7
RPL11--EFCAB14	7.7
RPL11--C21ORF58	7.7
RPL11--BRD4	7.7
RPL11--ATXN7	7.7
RPL11--ARFIP2	7.7
RPL10--UBC	7.7
RPGRIP1L--SLC12A7	7.7
RPGR--TLK1	7.7
RPGR--COX4I1	7.7
RPAP1--CUL3	7.7
ROCK1--SET	7.7
ROBO2--COL1A2	7.7
RO60--HNRNPC	7.7
RNPS1--TTN	7.7
RNPC3--PTPRA	7.7
RNF34--ZEB2	7.7
RNF213--RAD23B	7.7
RNF213--PPP5C	7.7
RNF213--HNRNPA3	7.7
RNF213--ATP2C1	7.7
RNF213--ALKBH5	7.7
RNF213--AD000090.1	7.7
RNF170--EPAS1	7.7
RNF152--UBA6	7.7
RNF149--UBE4B	7.7
RNF133--EFTUD2	7.7
RNF13--GNB1	7.7
RNF123--UBA52	7.7
RNF115--PIAS3	7.7
RNF10--HNRNPA1	7.7
RNASEH2A--MUC5B	7.7
RMRP--ZFAND3	7.7
RMRP--XIST	7.7
RMRP--USP24	7.7
RMRP--UBALD2	7.7
RMRP--TPX2	7.7
RMRP--SUGP2	7.7
RMRP--SMARCAL1	7.7
RMRP--SBNO1	7.7

RMRP--PDS5A	7.7
RMRP--NUP153	7.7
RMRP--MALAT1	7.7
RMRP--HSF1	7.7
RMRP--HEATR1	7.7
RMRP--GPCPD1	7.7
RMRP--GNAS	7.7
RMRP--FTH1	7.7
RMRP--C2CD5	7.7
RMRP--ATP2B1	7.7
RIOK3--SVIL	7.7
RIMS2--VPS13B	7.7
RIMKLB--LAMC1	7.7
RICTOR--AP3B1	7.7
RIC1--GNAS	7.7
RHOU--EBF1	7.7
RHOQ--UROS	7.7
RHOA--TPI1	7.7
RHOA--KNDC1	7.7
RFX7--TUBGCP5	7.7
RFC1--SEC24A	7.7
REV1--SON	7.7
REV1--SH3BGRL	7.7
RET--CDC42BPB	7.7
REST--MALAT1	7.7
RERE--RALGAPB	7.7
RERE--PRRC2A	7.7
RERE--DDX17	7.7
REPS1--IGFBP7	7.7
REPS1--CENPT	7.7
RELN--SETD5	7.7
RECK--PREPL	7.7
REC8--XIST	7.7
RC3H2--RPL8	7.7
RC3H2--HOXC6	7.7
RC3H2--CPSF6	7.7
RC3H1--TMF1	7.7
RC3H1--PPT1	7.7
RC3H1--MALAT1	7.7
RBPMS--ATXN2L	7.7
RBPJ--C8ORF33	7.7
RBMXL1--RPS17	7.7
RBMX2--ENAH	7.7
RBM5--TCERG1	7.7
RBM5--ABI1	7.7

RBM47--NAMPT	7.7
RBM47--HUWE1	7.7
RBM39--MALAT1	7.7
RBM39--HIST1H2BG	7.7
RBM39--G6PD	7.7
RBM38--BBS2	7.7
RBM24--FTH1	7.7
RBM15--TXNIP	7.7
RBM14-RBM4--KIF1C	7.7
RBM12--AP2A1	7.7
RBL1--ANKRD44	7.7
RBFOX2--PAXBP1	7.7
RBFOX2--KCNJ2	7.7
RBFA--MLLT10	7.7
RBBP6--AL645922.1	7.7
RBBP4--TCF4	7.7
RBBP4--GPI	7.7
RBBP4--FTH1	7.7
RB1--EP300	7.7
RASSF6--KAT2B	7.7
RARS2--OAZ1	7.7
RAPH1--NSD3	7.7
RAP1GAP--ATP5F1B	7.7
RANGAP1--CAPN12	7.7
RANBP2--MUC1	7.7
RANBP2--LIPF	7.7
RANBP2--FOXK1	7.7
RAN--MALAT1	7.7
RAN--BTAF1	7.7
RALY--EP300	7.7
RALY--CFL1	7.7
RALGAPA2--PSIP1	7.7
RALGAPA2--LPCAT3	7.7
RALGAPA1--VCL	7.7
RALGAPA1--MAPK7	7.7
RALBP1--THRAP3	7.7
RALB--SPATS2L	7.7
RALA--NAA20	7.7
RAD54L2--FTL	7.7
RAD54L2--DPY19L2	7.7
RAD51D--GTF2I	7.7
RAD51AP1--VHL	7.7
RAD23B--LAMA2	7.7
RAD21--ELOVL5	7.7
RAD21--ADAR	7.7

RAD18--COL1A2	7.7
RAD17--DMTF1	7.7
RAD17--ANO6	7.7
RACK1--STARD9	7.7
RACK1--RMRP	7.7
RACK1--AC108704.2	7.7
RAC1--HSPA9	7.7
RABEP1--WDR74	7.7
RAB8B--NT5C2	7.7
RAB8A--AXIN2	7.7
RAB8A--AC004805.1	7.7
RAB7A--UBC	7.7
RAB7A--ACAD9	7.7
RAB6B--BACH1	7.7
RAB3GAP2--PLIN3	7.7
RAB3GAP2--CES1	7.7
RAB3GAP1--PPP6R2	7.7
RAB2A--VIPAS39	7.7
RAB2A--FBRSL1	7.7
RAB21--PHF20L1	7.7
RAB18--SYNE2	7.7
RAB12--NOP58	7.7
RAB11A--CHD7	7.7
QSOX2--PKP4	7.7
QRICH2--CNOT1	7.7
QKI--SF3B1	7.7
PWWP2A--EIF4G2	7.7
PVT1--MYC	7.7
PUM3--ATP11C	7.7
PUM1--SRRM2	7.7
PTPRS--RAD21	7.7
PTPRN2--CUX1	7.7
PTPRG--MALAT1	7.7
PTPRF--PTCH2	7.7
PTPRF--LSP1	7.7
PTPRF--FLNC	7.7
PTPRC--BTBD2	7.7
PTPRA--MALAT1	7.7
PTPN3--FN1	7.7
PTPN14--SYNM	7.7
PTPN12--ZNF236	7.7
PTPN11--CREBBP	7.7
PTP4A2--SRSF10	7.7
PTOV1--AC063952.2	7.7
PTMS--PSMC4	7.7

PTMS--P4HA2	7.7
PTMS--GTF2A1L	7.7
PTMA--NEFM	7.7
PTMA--NEAT1	7.7
PTMA--MALAT1	7.7
PTMA--KIF21A	7.7
PTMA--CDC25A	7.7
PTK7--PRCP	7.7
PTGES3--FTH1	7.7
PTCH2--FTL	7.7
PTCH1--ECPAS	7.7
PTCH1--DMGDH	7.7
PTBP3--GAL	7.7
PTBP2--IGHMBP2	7.7
PTBP1--SH2B1	7.7
PTBP1--COPZ1	7.7
PTAR1--EIF2B1	7.7
PSMG4--STAG1	7.7
PSME4--RPL14	7.7
PSME4--MALAT1	7.7
PSME3--RPL37A	7.7
PSME1--SELENOT	7.7
PSME1--MAPK1IP1L	7.7
PSMD2--PRR34-AS1	7.7
PSMD2--GAP43	7.7
PSMD13--ZNF664	7.7
PSMD12--AHNAK	7.7
PSMC4--ATP13A3	7.7
PSMB4--CTCF	7.7
PSMA3--STIL	7.7
PSMA3--FREM2	7.7
PSIP1--SAR1A	7.7
PSIP1--IGF2	7.7
PSIP1--CCDC171	7.7
PSD3--TUBB	7.7
PSAT1--UBR4	7.7
PSAP--NPLOC4	7.7
PSAP--KRT7	7.7
PSAP--FCHSD2	7.7
PRRT1--PPM1G	7.7
PRRG3--RBM6	7.7
PRRC2C--ZC3H7A	7.7
PRRC2C--TBC1D14	7.7
PRRC2C--SMC5	7.7
PRRC2C--RP1	7.7

PRRC2C--NFAT5	7.7
PRRC2C--MALAT1	7.7
PRRC2C--ENTPD4	7.7
PRRC2C--DNAJA1	7.7
PRRC2C--CYP4B1	7.7
PRRC2C--CHST11	7.7
PRRC2B--NEFM	7.7
PRRC2B--MALAT1	7.7
PRRC2A--STOM	7.7
PRRC2A--MALAT1	7.7
PRR5-ARHGAP8--TIMP1	7.7
PRPSAP1--HECTD1	7.7
PRPF8--RNF38	7.7
PRPF8--RIF1	7.7
PRPF8--PPARG	7.7
PRPF8--MALAT1	7.7
PRPF4B--SERPINA1	7.7
PRPF38B--TRB@	7.7
PRPF3--ZMYND8	7.7
PROCR--RHOB	7.7
PROCA1--PDCD10	7.7
PROC--NR2F2	7.7
PRNP--ZBTB22	7.7
PRNP--SYNPO2	7.7
PRMT7--RPPH1	7.7
PRMT5--PTCH2	7.7
PRKDC--MALAT1	7.7
PRKDC--ACTG1	7.7
PRKCSH--RNF145	7.7
PRKAB2--STARD7	7.7
PRKAB2--HNRNPC	7.7
PRIM1--UGGT1	7.7
PRICKLE2--BPNT1	7.7
PREPL--IGK@	7.7
PREP--FAM172A	7.7
PRELID2--MALAT1	7.7
PRDM2--EIF5	7.7
PQBP1--TALDO1	7.7
PPWD1--ESD	7.7
PPRC1--PPP4R3A	7.7
PPP4R3B--SETD4	7.7
PPP4R3A--CYP4F11	7.7
PPP4R1--UQCRFS1	7.7
PPP2R3B--MROH1	7.7

PPP2R1A--PKP4	7.7
PPP2R1A--CCDC82	7.7
PPP1R3B--SERPINH1	7.7
PPP1R12C--PLBD1	7.7
PPP1R12C--DDX39B	7.7
PPP1R12B--SS18L1	7.7
PPP1CC--SUPT16H	7.7
PPP1CA--RP1L1	7.7
PPM1L--NLRP3	7.7
PPM1L--EFHD2	7.7
PPM1G--HMGCS1	7.7
PPM1G--ABCC2	7.7
PPIP5K2--CDK16	7.7
PPIP5K1--LGR4	7.7
PPIL3--MTA2	7.7
PPIH--YBX1	7.7
PPIG--PDPK1	7.7
PPIG--B3GALNT1	7.7
PPIG--AL627309.5	7.7
PPIB--SLC35G1	7.7
PPIB--LAMB3	7.7
PPIA--RRBP1	7.7
PPFIBP1--WDFY3	7.7
PPARG--ACTG1	7.7
POU2F1--SPIN1	7.7
POSTN--CLIP4	7.7
POSTN--C1R	7.7
POMT2--RCC1	7.7
POM121C--RBAK	7.7
POLR3G--AARS	7.7
POLR3E--ERGIC1	7.7
POLR3A--P4HA2	7.7
POLR2E--WDR74	7.7
POLR2B--AP002373.1	7.7
POLR2A--KPNB1	7.7
POLR2A--KANK3	7.7
POLR1B--RAP1GAP2	7.7
POLR1A--COMMD9	7.7
POLG--BRWD1	7.7
POLD2--COL1A2	7.7
POLA1--NEB	7.7
POGZ--RNF111	7.7
POGK--EXO1	7.7
PNPT1--SCARNA9	7.7
PNISR--PTAR1	7.7

PLXND1--GFM1	7.7
PLXNB3--GTF2I	7.7
PLXNB2--RPPH1	7.7
PLXNB2--PTPRN2	7.7
PLXNB2--NUAK2	7.7
PLXNB1--PRPF8	7.7
PLXNA1--UBC	7.7
PLXNA1--NOP9	7.7
PLSCR1--SERPINH1	7.7
PLPP5--DDX5	7.7
PLP2--MUC4	7.7
PLOD1--ZNF91	7.7
PLOD1--PARP3	7.7
PLLP--MALAT1	7.7
PLEKHO1--COL11A1	7.7
PLEKHM3--FAS	7.7
PLEKHH1--VMP1	7.7
PLEKHG3--PHF2	7.7
PLEC--CHSY1	7.7
PLCL1--SSR1	7.7
PLCG1--KIF26A	7.7
PLA2G2A--ZNF766	7.7
PKP3--MALAT1	7.7
PKP3--EIF4G2	7.7
PKP1--BET1L	7.7
PKD1--ARHGAP26	7.7
PITRM1--CCT6A	7.7
PITRM1--AL358933.1	7.7
PITPNM2--PSMD14	7.7
PITPNA--ZNF281	7.7
PITPNA--EEF1A1	7.7
PIP5K1C--TUBB	7.7
PINK1-AS--TM9SF2	7.7
PIKFYVE--DOCK6	7.7
PIK3C3--WDR74	7.7
PIK3C2G--CDC42BPA	7.7
PIK3C2B--CALR	7.7
PIGW--MACC1	7.7
PIGS--APH1A	7.7
PIGB--TCF4	7.7
PIF1--PLK1	7.7
PIEZO1--MAZ	7.7
PICALM--SYTL2	7.7
PICALM--MYH9	7.7
PIBF1--SPIDR	7.7

PIBF1--KLF5	7.7
PIAS3--NUP98	7.7
PHTF2--PTPN12	7.7
PHTF1--NDUFV1	7.7
PHPT1--BIRC6	7.7
PHLDA1--DVL1	7.7
PHKB--PHF3	7.7
PHKB--KIAA1958	7.7
PHIP--TUBB	7.7
PHIP--SPTBN1	7.7
PHIP--PAWR	7.7
PHIP--COL24A1	7.7
PHGDH--ZNF469	7.7
PHF3--TUBA1B	7.7
PHF20--LARP4	7.7
PHF19--P2RX7	7.7
PHF14--VPS13D	7.7
PHF14--NCOR1	7.7
PHF13--MTA3	7.7
PHF12--DNAH5	7.7
PHC3--CAT	7.7
PHACTR1--MSN	7.7
PGRMC2--STMN2	7.7
PGM3--VPS13A	7.7
PGM2--SMG1	7.7
PGM2--BRWD1	7.7
PGK1--ZRANB3	7.7
PGD--FAM117B	7.7
PGBD1--ZNF276	7.7
PEX26--ACADVL	7.7
PEX2--TUBB	7.7
PEX19--SMG1	7.7
PERM1--YTHDF2	7.7
PELI2--PTCD3	7.7
PEG3--HNRNPU	7.7
PDXDC1--HTT	7.7
PDS5B--CHST11	7.7
PDS5A--SERPINI1	7.7
PDS5A--NIN	7.7
PDLIM2--BECN1	7.7
PDK2--SYNJ1	7.7
PDIA6--ARFGEF2	7.7
PDIA3--HR	7.7
PDHB--GPX2	7.7
PDE8A--SCAF8	7.7

PDE7A--TPM2	7.7
PDE5A--NLRC4	7.7
PDCD2L--AK2	7.7
PCSK5--NBR1	7.7
PCNX4--PHF8	7.7
PCNX4--MUC4	7.7
PCNX4--CNNM4	7.7
PCNP--SERPINF1	7.7
PCNA--NEAT1	7.7
PCMTD1--PTPRG	7.7
PCMT1--PKP4	7.7
PCM1--STT3A	7.7
PCM1--RHOXF2B	7.7
PCM1--EEF1A1	7.7
PCLO--ZCCHC14	7.7
PCLO--SMARCAD1	7.7
PCLO--CACNA2D1	7.7
PCLO--AC124312.3	7.7
PCID2--SETX	7.7
PCGF5--EGR1	7.7
PCED1A--PHLPP1	7.7
PCBP2--MKLN1	7.7
PCBP1--EZH1	7.7
PCBD1--MAST3	7.7
PC--MDM2	7.7
PBXIP1--PDCD11	7.7
PBX1--PTMS	7.7
PAXIP1--RSF1	7.7
PAXIP1--NSMAF	7.7
PAWR--SGCA	7.7
PATJ--OAS3	7.7
PATJ--MALAT1	7.7
PASK--EFHD2	7.7
PARVA--TEAD1	7.7
PARP9--CUL4B	7.7
PARP16--SDHAF1	7.7
PARP14--RMRP	7.7
PARP14--PPP2CB	7.7
PARP1--EDRF1	7.7
PARG--BMS1	7.7
PARD3--SGMS1	7.7
PAPOLA--SYNE2	7.7
PANK3--MUC4	7.7
PANK3--GKAP1	7.7
PAN2--KLK11	7.7
PAM--AKAP1	7.7
PALLD--TAGLN	7.7
PALLD--SLC35B4	7.7
PALLD--SH3BP5L	7.7
PAICS--MEGF8	7.7
PAFAH1B2--CD276	7.7
PAFAH1B1--NSUN5	7.7
PACRGL--CLTC	7.7
PABPC4--LDHA	7.7
PABPC4--CCT5	7.7
PABPC1--TMEM106C	7.7
PABPC1--SENP7	7.7
PABPC1--MALAT1	7.7
PABPC1--GANAB	7.7
PABPC1--GAK	7.7
PA2G4--S1PR3	7.7
PA2G4--MALAT1	7.7
OTUD7B--IER3IP1	7.7
OSTC--LIG3	7.7
OSGIN2--COL6A3	7.7
OSGIN1--CHD6	7.7
OSBPL9--FAF1	7.7
OSBPL8--NAP1L1	7.7
OS9--VPS13D	7.7
OS9--TBCK	7.7
OS9--TAF7L	7.7
ORAI2--CNNM4	7.7
OR7H1P--OR7E55P	7.7
OR2A14--OR2A15P	7.7
ONECUT2--SHROOM3	7.7
ONECUT2--ADAM9	7.7
OMA1--ITGAV	7.7
OIP5-AS1--DIS3	7.7
OGT--SMG7	7.7
OGN--COL1A2	7.7
OGDHL--GOLM1	7.7
OGDH--PSMD7	7.7
OGA--COL1A1	7.7
ODF2L--CP	7.7
OBSCN--CHGB	7.7
OAZ2--NOL10	7.7
NYNRIN--KIAA1549	7.7
NXPE3--TMEM106A	7.7
NVL--TUBB	7.7
NVL--MALAT1	7.7
NUTM2A-AS1--MUC4	7.7
NUP62--ACTB	7.7
NUP58--DBI	7.7
NUP214--TTN-AS1	7.7
NUP214--DHX9	7.7
NUP210--ADAMTS1	7.7
NUP205--STMP1	7.7
NUP155--MALAT1	7.7
NUP153--DGCR8	7.7
NUP153--AL513165.2	7.7
NUP133--YTHDC1	7.7
NUMA1--ZNF483	7.7
NUFIP2--EIF4G1	7.7
NUF2--ZMYND8	7.7
NUCKS1--SCAMP5	7.7
NUCKS1--LETM1	7.7
NUCKS1--CPD	7.7
NUCKS1--ATP8B2	7.7
NTRK2--FAT1	7.7
NTHL1--TRIM25	7.7
NT5DC2--SNRPB	7.7
NT5C3A--TSPYL4	7.7
NT5C3A--ALG8	7.7
NSMF--DNMT1	7.7
NSF--MBNL1	7.7
NSD3--CAMSAP2	7.7
NSD1--ZFP36L1	7.7
NSD1--RPL14	7.7
NSD1--MALAT1	7.7
NRDE2--RMRP	7.7
NRDC--ZNF644	7.7
NRCAM--NEB	7.7
NR6A1--ATP5F1B	7.7
NR4A1--ACTG1	7.7
NR2F2--SV2B	7.7
NR2F2--DAZAP2	7.7
NR2F1-AS1--ULK4	7.7
NQO1--NADK	7.7
NQO1--HSPA8	7.7
NQO1--BBS5	7.7
NPTN--ESYT1	7.7
NPM1--PAX8	7.7
NPM1--LINC01193	7.7
NPM1--BRWD1	7.7
NPLOC4--RAI1	7.7

NPIPB5--ZNF347	7.7
NPHP4--MALAT1	7.7
NPEPL1--EEF1A1	7.7
NPC1--AHSA2P	7.7
NPAT--USP9X	7.7
NOTUM--RERE	7.7
NOTCH3--CACNA1A	7.7
NOTCH2--SMG1	7.7
NOTCH2--MUC4	7.7
NOTCH2--FTL	7.7
NORAD--ZNF551	7.7
NOL9--AC138811.2	7.7
NOC2L--NUP62	7.7
NMNAT3--RNF213	7.7
NMNAT1--AC044839.1	7.7
NME6--GAS6	7.7
NLRP12--ZC3H13	7.7
NKIRAS2--NEB	7.7
NKD1--NSMAF	7.7
NISCH--SMG1	7.7
NIPBL-DT--TUT4	7.7
NIPBL--VPS13C	7.7
NIPBL--ATP2A2	7.7
NIBAN1--DSP	7.7
NHP2--RPL30	7.7
NHLRC3--NIPBL	7.7
NHLRC2--NAGK	7.7
NFX1--LDLR	7.7
NFX1--AUXG01000058.1	7.7
NFKBIZ--UTP15	7.7
NFIB--PSIP1	7.7
NFIA--TBC1D25	7.7
NFIA--SH3BP4	7.7
NFE2L2--IGKC	7.7
NFE2L2--ABCD3	7.7
NFE2L1--XIST	7.7
NFAT5--UTRN	7.7
NFAT5--MALAT1	7.7
NFAT5--EIF3K	7.7
NF1--PACSIN2	7.7
NF1--LPP	7.7
NEURL4--NDUFC1	7.7
NES--TOR1AIP2	7.7
NEO1--AC124312.3	7.7

NEMF--FGFR2	7.7
NEK4--PEBP1	7.7
NEFL--KAT6A	7.7
NEDD1--TNRC6B	7.7
NEBL--MED13L	7.7
NEB--XIST	7.7
NEB--USP31	7.7
NEB--NIPBL	7.7
NEB--MALAT1	7.7
NEB--ETV5	7.7
NEB--BAG2	7.7
NEAT1--USP34	7.7
NEAT1--UHMK1	7.7
NEAT1--UCHL1	7.7
NEAT1--SPATA6	7.7
NEAT1--RNF213	7.7
NEAT1--RMRP	7.7
NEAT1--RBBP4	7.7
NEAT1--RARRES1	7.7
NEAT1--PTPN22	7.7
NEAT1--PHF3	7.7
NEAT1--NCOA2	7.7
NEAT1--MUC4	7.7
NEAT1--MAP3K7	7.7
NEAT1--LUC7L3	7.7
NEAT1--LDHA	7.7
NEAT1--KPNB1	7.7
NEAT1--KAT6B	7.7
NEAT1--HMGXB4	7.7
NEAT1--GTF2I	7.7
NEAT1--GART	7.7
NEAT1--FTL	7.7
NEAT1--CNPY4	7.7
NEAT1--CDK12	7.7
NEAT1--CCNT1	7.7
NEAT1--ABCD3	7.7
NDUFS5--CSE1L	7.7
NDUFS1--SCN9A	7.7
NDUFS1--RSPO4	7.7
NDUFS1--DMXL2	7.7
NDUFB5--GLUD1	7.7
NDUFB3--AL157871.3	7.7
NDUFA6--OSBPL6	7.7
NDUFA10--HDAC4	7.7
NDST1--SESN2	7.7

NDRG1--DPP8	7.7
NDNF--COPS6	7.7
NDFIP1--LMNB1	7.7
NDE1--RC3H2	7.7
NCOR2--SRGAP2	7.7
NCOR2--EP400	7.7
NCOR1--PLEC	7.7
NCOR1--PIK3CA	7.7
NCOA6--PSPH	7.7
NCOA3--SUSD6	7.7
NCOA3--ARHGAP29	7.7
NCOA3--ADD1	7.7
NCOA1--STK38L	7.7
NCL--PHB2	7.7
NCL--PALM3	7.7
NCL--PABPC4	7.7
NCL--MMP15	7.7
NCL--KIF1C	7.7
NCL--ACTG1	7.7
NCKAP1--MALAT1	7.7
NCKAP1--CLCN6	7.7
NCF1B--NCF1	7.7
NCAPH2--RNF213	7.7
NCAPD2--ACTB	7.7
NBPF9--PRSS23	7.7
NBPF9--NEAT1	7.7
NBPF8--NEAT1	7.7
NBPF3--SCAF11	7.7
NBPF13P--AC239859.1	7.7
NBN--EVI2B	7.7
NBEAL1--NEAT1	7.7
NAV2--EPB41L5	7.7
NAV2--CHST10	7.7
NAT10--ZNF808	7.7
NAT10--RCAN1	7.7
NAT10--PCNX1	7.7
NARF--YLPM1	7.7
NAPG--TRIM34	7.7
NAP1L1--TMCO6	7.7
NAP1L1--MARK1	7.7
NAMPT--NEAT1	7.7
NAGPA--GFM1	7.7
NACA--COL5A2	7.7
NAA50--DSP	7.7
NAA16--TAB2	7.7

N4BP2--UHRF2	7.7
N4BP1--MSL3	7.7
MYOM3--SFT2D3	7.7
MYO9B--ZNF385A	7.7
MYO9B--IBTK	7.7
MYO6--TMSB4X	7.7
MYO6--DAPK1	7.7
MYO6--APLP2	7.7
MYO5C--NEBL	7.7
MYO3B--ISYNA1	7.7
MYO1F--AARS2	7.7
MYO1D--MNAT1	7.7
MYO1B--CDK2	7.7
MYO19--MALAT1	7.7
MYO10--SH3GLB1	7.7
MYLK--ZBTB7A	7.7
MYLK--AC092445.1	7.7
MYL12A--PSAP	7.7
MYH9--SOX13	7.7
MYH9--GFOD2	7.7
MYH3--TNNT3	7.7
MYH11--TTC3	7.7
MYH11--SCARNA5	7.7
MYCN--LINC01128	7.7
MYCBP2--NAGK	7.7
MYC--MYH11	7.7
MYBL2--PPP6R1	7.7
MYBBP1A--PRPF8	7.7
MYADM--F5	7.7
MVP--OCIAD1	7.7
MVK--TSKU	7.7
MUC5B--SRRM2	7.7
MUC5B--MARCH8	7.7
MUC5B--CEP57	7.7
MUC4--WWOX	7.7
MUC4--TMSB4X	7.7
MUC4--MAPK13	7.7
MUC4--DDX47	7.7
MUC4--CIT	7.7
MUC4--CALR	7.7
MUC4--ADAM10	7.7
MUC16--TPR	7.7
MUC16--NEAT1	7.7
MUC16--KIAA0100	7.7
MUC16--FUS	7.7

MTREX--USP9X	7.7
MTOR--SYP	7.7
MTMR4--DNAAF2	7.7
MTMR3--HIST1H2AE	7.7
MTMR2--TLE5	7.7
MTMR10--STT3B	7.7
MTMR10--AARS2	7.7
MTHFD2--KANSL3	7.7
MTHFD1--ANO10	7.7
MTFMT--MALAT1	7.7
MTERF4--RPL10A	7.7
MTCH2--CNTNAP4	7.7
MSRB3--FMO5	7.7
MSN--MALAT1	7.7
MSN--APLN	7.7
MSMO1--FTH1	7.7
MSI2--IFT57	7.7
MSI2--DCAF7	7.7
MSH6--REV3L	7.7
MSH6--AL021155.5	7.7
MSH3--PKM	7.7
MSANTD4--ADGRV1	7.7
MRTFA--XPNPEP3	7.7
MRPS27--PIK3C2B	7.7
MRPS26--FXYD6-FXYD2	7.7
MRPS25--HOMER3	7.7
MRPS18A--LLPH-DT	7.7
MRPL45--PPIG	7.7
MRPL39--COL1A2	7.7
MRPL32--MALAT1	7.7
MRPL1--GDI1	7.7
MRFAP1L1--NIPA1	7.7
MREG--SCG2	7.7
MRC1--COMMD2	7.7
MPRIP--CSRP1	7.7
MPP6--QSER1	7.7
MPP6--BBX	7.7
MPC2--MALAT1	7.7
MORF4L1--MALAT1	7.7
MORC4--MALAT1	7.7
MORC3--TTN	7.7
MORC3--HMGN2	7.7
MON2--FTL	7.7
MOB3A--EIF4G3	7.7

MOB1B--EXOC4	7.7
MOAP1--ANP32C	7.7
MNT--MUC4	7.7
MMADHC--DNMT3A	7.7
MLLT3--MALAT1	7.7
MLLT10--CREBZF	7.7
MLH3--CNN3	7.7
MKRN1--NINL	7.7
MKLN1--RPS17	7.7
MKLN1--MALAT1	7.7
MKI67--SAP130	7.7
MKI67--PTPRK	7.7
MKI67--NUCKS1	7.7
MITF--GLIS2	7.7
MIR100HG--PABPC1	7.7
MINDY2--MAPK6	7.7
MICALL1--MALAT1	7.7
MICAL3--MALAT1	7.7
MIAT--SGSH	7.7
MGRN1--FBXO45	7.7
MGA--NSF	7.7
MGA--MALAT1	7.7
MFSD6--WNK1	7.7
MFSD4B--GCNT1	7.7
MFN1--CRYBG3	7.7
MFHAS1--NIPBL	7.7
METTL21A--NEDD4	7.7
MET--KIF21A	7.7
MEP1A--TAF4B	7.7
MEIOB--TLN2	7.7
MEGF10--XIST	7.7
MED28--FREM2	7.7
MED13L--GOT1	7.7
MED12--SERPINA1	7.7
MED12--LINC00632	7.7
MECP2--RYR2	7.7
MEAF6--TFAP2A	7.7
MDN1--AKAP9	7.7
MDM4--CTCF	7.7
MDM2--HSPA9	7.7
MDM2--CWF19L2	7.7
MDM1--EIF4G2	7.7
MDK--SOGA1	7.7
MDC1--LARGE1	7.7
MDC1--GTF2I	7.7

MCTP2--ZNF292	7.7
MCOLN3--MALAT1	7.7
MCM9--GJB3	7.7
MCM7--AC019117.3	7.7
MCM6--PASK	7.7
MCM3--TRG@	7.7
MCL1--ABCC2	7.7
MCL1--AATF	7.7
MCFD2--RMRP	7.7
MCCC2--DIS3L2	7.7
MCCC1--INTS1	7.7
MBTD1--PTP4A2	7.7
MBP--ODC1	7.7
MBD1--PCDHGA10	7.7
MAX--DTWD1	7.7
MAU2--IDH1	7.7
MATR3--FOXP1	7.7
MASTL--RNF213	7.7
MARS--RMRP	7.7
MARCKS--DDX5	7.7
MARCH6--HSBP1	7.7
MAPKAPK2--PCID2	7.7
MAPK1IP1L--PEX19	7.7
MAP7D2--NOL12	7.7
MAP4K4--PDK2	7.7
MAP3K4--MIR9-3HG	7.7
MAP3K4--MALAT1	7.7
MAP3K13--AVPR1A	7.7
MAP3K10--LINC01833	7.7
MANEA--NINL	7.7
MAN2B1--AC005332.6	7.7
MAN2A2--APEH	7.7
MAN2A1--WDR6	7.7
MAN2A1--RICTOR	7.7
MAN2A1--MALAT1	7.7
MAN1B1--MFSD14B	7.7
MAN1A2--RPS8	7.7
MAN1A2--KNL1	7.7
MAML1--SALL1	7.7
MAML1--IL6ST	7.7
MALT1--ARHGAP35	7.7
MALRD1--AL845552.1	7.7
MALAT1--ZZEF1	7.7
MALAT1--ZNF93	7.7
MALAT1--ZNF708	7.7

MALAT1--ZNF347	7.7
MALAT1--ZNF33A	7.7
MALAT1--ZNF24	7.7
MALAT1--ZNF143	7.7
MALAT1--ZMIZ2	7.7
MALAT1--ZFHX3	7.7
MALAT1--ZFC3H1	7.7
MALAT1--ZEB2	7.7
MALAT1--ZCCHC7	7.7
MALAT1--ZC3HAV1	7.7
MALAT1--ZBTB20	7.7
MALAT1--YWHAZ	7.7
MALAT1--YTHDF3	7.7
MALAT1--YIPF3	7.7
MALAT1--YBX1	7.7
MALAT1--XRN1	7.7
MALAT1--XRCC6	7.7
MALAT1--XRCC5	7.7
MALAT1--XPO6	7.7
MALAT1--XPO1	7.7
MALAT1--WWOX	7.7
MALAT1--WTAP	7.7
MALAT1--WSB1	7.7
MALAT1--WNK1	7.7
MALAT1--WDFY3	7.7
MALAT1--WASHC4	7.7
MALAT1--WASH5P	7.7
MALAT1--WAPL	7.7
MALAT1--VWA8	7.7
MALAT1--VPS8	7.7
MALAT1--VPS50	7.7
MALAT1--VPS13C	7.7
MALAT1--VCAN	7.7
MALAT1--VAPB	7.7
MALAT1--USP7	7.7
MALAT1--USF2	7.7
MALAT1--UQCRB	7.7
MALAT1--UQCC1	7.7
MALAT1--ULK1	7.7
MALAT1--UBR5	7.7
MALAT1--UBR4	7.7
MALAT1--UBE2R2	7.7
MALAT1--UBAP2L	7.7
MALAT1--UBAP2	7.7
MALAT1--TXNRD1	7.7

MALAT1--TUT7	7.7
MALAT1--TTN	7.7
MALAT1--TTC21B	7.7
MALAT1--TTC19	7.7
MALAT1--TTC14	7.7
MALAT1--TRAM1	7.7
MALAT1--TRAK1	7.7
MALAT1--TRA@	7.7
MALAT1--TPD52	7.7
MALAT1--TOPBP1	7.7
MALAT1--TOP1	7.7
MALAT1--TNS3	7.7
MALAT1--TMEM131	7.7
MALAT1--TMED5	7.7
MALAT1--TMED2	7.7
MALAT1--TMCO6	7.7
MALAT1--TMBIM6	7.7
MALAT1--TM4SF1-AS1	7.7
MALAT1--TJP1	7.7
MALAT1--TF	7.7
MALAT1--TET3	7.7
MALAT1--TCERG1	7.7
MALAT1--TC2N	7.7
MALAT1--TAS2R30	7.7
MALAT1--TANGO2	7.7
MALAT1--TALDO1	7.7
MALAT1--TAF6	7.7
MALAT1--TAF1D	7.7
MALAT1--TAF15	7.7
MALAT1--SYT13	7.7
MALAT1--SYNE1	7.7
MALAT1--SYAP1	7.7
MALAT1--SUZ12	7.7
MALAT1--SUPT5H	7.7
MALAT1--STRN4	7.7
MALAT1--STAT1	7.7
MALAT1--STAMBP	7.7
MALAT1--ST5	7.7
MALAT1--SRSF11	7.7
MALAT1--SRSF10	7.7
MALAT1--SRSF1	7.7
MALAT1--SRRM2	7.7
MALAT1--SQSTM1	7.7
MALAT1--SPTAN1	7.7
MALAT1--SPG7	7.7

MALAT1--SPG11	7.7
MALAT1--SPEN	7.7
MALAT1--SPARC	7.7
MALAT1--SPAG9	7.7
MALAT1--SON	7.7
MALAT1--SOCS4	7.7
MALAT1--SNX29	7.7
MALAT1--SMG5	7.7
MALAT1--SMCHD1	7.7
MALAT1--SMC5	7.7
MALAT1--SLC44A3	7.7
MALAT1--SLC44A1	7.7
MALAT1--SLC40A1	7.7
MALAT1--SLC39A11	7.7
MALAT1--SLC29A1	7.7
MALAT1--SLC25A37	7.7
MALAT1--SLC25A22	7.7
MALAT1--SLC17A5	7.7
MALAT1--SIPA1L1	7.7
MALAT1--SIN3B	7.7
MALAT1--SGPL1	7.7
MALAT1--SF1	7.7
MALAT1--SETD5	7.7
MALAT1--SET	7.7
MALAT1--SERINC1	7.7
MALAT1--SERBP1	7.7
MALAT1--SEMA4B	7.7
MALAT1--SEL1L3	7.7
MALAT1--SEC11C	7.7
MALAT1--SDHC	7.7
MALAT1--SCRN1	7.7
MALAT1--SCN8A	7.7
MALAT1--SCARNA7	7.7
MALAT1--SCARNA13	7.7
MALAT1--SCAPER	7.7
MALAT1--SAMD4B	7.7
MALAT1--SAFB	7.7
MALAT1--RTTN	7.7
MALAT1--RTCA-AS1	7.7
MALAT1--RSRC1	7.7
MALAT1--RRBP1	7.7
MALAT1--RPS9	7.7
MALAT1--RPS8	7.7
MALAT1--RPS27	7.7
MALAT1--RPS11	7.7

MALAT1--RPS10-NUDT3	7.7
MALAT1--RPRD2	7.7
MALAT1--RPN2	7.7
MALAT1--RPLP1	7.7
MALAT1--RPL5	7.7
MALAT1--RPL35A	7.7
MALAT1--RPL32	7.7
MALAT1--RPL22	7.7
MALAT1--ROBO1	7.7
MALAT1--RNF10	7.7
MALAT1--RN7SL151P	7.7
MALAT1--RN7SL1	7.7
MALAT1--RMND5B	7.7
MALAT1--RIPOR2	7.7
MALAT1--RIMS2	7.7
MALAT1--RIBC1	7.7
MALAT1--RHOBTB3	7.7
MALAT1--REV3L	7.7
MALAT1--RETREG3	7.7
MALAT1--RERE	7.7
MALAT1--REPS1	7.7
MALAT1--REL	7.7
MALAT1--RCAN3	7.7
MALAT1--RBM41	7.7
MALAT1--RBM26	7.7
MALAT1--RBL2	7.7
MALAT1--RBBP4	7.7
MALAT1--RASA1	7.7
MALAT1--RAPGEF4	7.7
MALAT1--RAPGEF2	7.7
MALAT1--RAF1	7.7
MALAT1--RAD21	7.7
MALAT1--RAB5A	7.7
MALAT1--RAB3IP	7.7
MALAT1--RAB22A	7.7
MALAT1--PXK	7.7
MALAT1--PTCH2	7.7
MALAT1--PTCD3	7.7
MALAT1--PTBP2	7.7
MALAT1--PSME3	7.7
MALAT1--PSMD11	7.7
MALAT1--PSIP1	7.7
MALAT1--PRRC2B	7.7
MALAT1--PRR11	7.7

MALAT1--PROX1	7.7
MALAT1--PRKACA	7.7
MALAT1--PRKAB1	7.7
MALAT1--PRIM1	7.7
MALAT1--PPP4R3B	7.7
MALAT1--PPP1CB	7.7
MALAT1--PPIL4	7.7
MALAT1--PNN	7.7
MALAT1--PMS1	7.7
MALAT1--PLSCR1	7.7
MALAT1--PLOD2	7.7
MALAT1--PLEKHA2	7.7
MALAT1--PLEC	7.7
MALAT1--PLCG2	7.7
MALAT1--PLA2G4B	7.7
MALAT1--PKP4	7.7
MALAT1--PITPNB	7.7
MALAT1--PI4KA	7.7
MALAT1--PHKA2	7.7
MALAT1--PGM5	7.7
MALAT1--PGM3	7.7
MALAT1--PFN2	7.7
MALAT1--PEX13	7.7
MALAT1--PDS5B	7.7
MALAT1--PDIA3	7.7
MALAT1--PDHB	7.7
MALAT1--PCLO	7.7
MALAT1--PBX3	7.7
MALAT1--PARP14	7.7
MALAT1--PAPOLA	7.7
MALAT1--PANK2	7.7
MALAT1--PABPC1L	7.7
MALAT1--PABPC1	7.7
MALAT1--OXNAD1	7.7
MALAT1--OTULIN	7.7
MALAT1--OSBPL3	7.7
MALAT1--OSBPL1A	7.7
MALAT1--ORC3	7.7
MALAT1--ONECUT2	7.7
MALAT1--NUP153	7.7
MALAT1--NSD2	7.7
MALAT1--NRDC	7.7
MALAT1--NQO1	7.7
MALAT1--NPM1	7.7
MALAT1--NPIPB5	7.7

MALAT1--NPEPPS	7.7
MALAT1--NOTCH2	7.7
MALAT1--NOS3	7.7
MALAT1--NORAD	7.7
MALAT1--NHLRC2	7.7
MALAT1--NF1	7.7
MALAT1--NET1	7.7
MALAT1--NES	7.7
MALAT1--NEK9	7.7
MALAT1--NEBL	7.7
MALAT1--NEB	7.7
MALAT1--NCOA3	7.7
MALAT1--NCLN	7.7
MALAT1--NCL	7.7
MALAT1--NACA	7.7
MALAT1--N4BP2	7.7
MALAT1--MYRF	7.7
MALAT1--MYO9A	7.7
MALAT1--MYL6	7.7
MALAT1--MUC4	7.7
MALAT1--MUC16	7.7
MALAT1--MUC1	7.7
MALAT1--MRC2	7.7
MALAT1--MORF4L2	7.7
MALAT1--MOB1A	7.7
MALAT1--MLX	7.7
MALAT1--MKI67	7.7
MALAT1--MIOS	7.7
MALAT1--MGAT4A	7.7
MALAT1--MED23	7.7
MALAT1--MED15	7.7
MALAT1--MED13L	7.7
MALAT1--MED13	7.7
MALAT1--MBTD1	7.7
MALAT1--MBNL2	7.7
MALAT1--MAVS	7.7
MALAT1--MAT2A	7.7
MALAT1--MAST2	7.7
MALAT1--MAP4K4	7.7
MALAT1--MAP2K4	7.7
MALAT1--MALT1	7.7
MALAT1--LUM	7.7
MALAT1--LUC7L3	7.7
MALAT1--LRP8	7.7
MALAT1--LRBA	7.7

MALAT1--LMO7	7.7
MALAT1--LINC00910	7.7
MALAT1--LEO1	7.7
MALAT1--LDLR	7.7
MALAT1--LDHA	7.7
MALAT1--LARS2	7.7
MALAT1--LAMB1	7.7
MALAT1--LAMA3	7.7
MALAT1--LAMA1	7.7
MALAT1--KRT7	7.7
MALAT1--KRT18	7.7
MALAT1--KPNB1	7.7
MALAT1--KMT2C	7.7
MALAT1--KMT2A	7.7
MALAT1--KIF11	7.7
MALAT1--KIAA2012	7.7
MALAT1--KIAA1841	7.7
MALAT1--KHDRBS1	7.7
MALAT1--KDM3B	7.7
MALAT1--KCNQ4	7.7
MALAT1--KCNQ1OT1	7.7
MALAT1--KCNK6	7.7
MALAT1--KAT6B	7.7
MALAT1--KANSL1	7.7
MALAT1--JUP	7.7
MALAT1--IQGAP1	7.7
MALAT1--IPO7	7.7
MALAT1--INTS2	7.7
MALAT1--INPP1	7.7
MALAT1--ING1	7.7
MALAT1--IL6ST	7.7
MALAT1--IGF1R	7.7
MALAT1--IFT122	7.7
MALAT1--HSPD1	7.7
MALAT1--HSPA9	7.7
MALAT1--HSPA4	7.7
MALAT1--HSP90AA1	7.7
MALAT1--HRG	7.7
MALAT1--HNRNPR	7.7
MALAT1--HNRNPH1	7.7
MALAT1--HNRNPC	7.7
MALAT1--HMGN2	7.7
MALAT1--HMGA1	7.7
MALAT1--HMCN1	7.7
MALAT1--HELZ	7.7

MALAT1--HDLBP	7.7
MALAT1--HCFC2	7.7
MALAT1--GTF3C4	7.7
MALAT1--GRN	7.7
MALAT1--GPBP1	7.7
MALAT1--GPATCH2L	7.7
MALAT1--GOLGA8A	7.7
MALAT1--GOLGA3	7.7
MALAT1--GMNN	7.7
MALAT1--GCLC	7.7
MALAT1--GART	7.7
MALAT1--GAPDH	7.7
MALAT1--GALT	7.7
MALAT1--GABPB2	7.7
MALAT1--FUS	7.7
MALAT1--FTH1	7.7
MALAT1--FRS2	7.7
MALAT1--FREM2	7.7
MALAT1--FOXP1	7.7
MALAT1--FNIP2	7.7
MALAT1--FMNL2	7.7
MALAT1--FLT4	7.7
MALAT1--FEM1A	7.7
MALAT1--FBXW2	7.7
MALAT1--FBXL3	7.7
MALAT1--FBXL13	7.7
MALAT1--FAM95C	7.7
MALAT1--FAM13A	7.7
MALAT1--EXTL3	7.7
MALAT1--EXOC3	7.7
MALAT1--ETV6	7.7
MALAT1--ETS2	7.7
MALAT1--ETF1	7.7
MALAT1--ERGIC2	7.7
MALAT1--ERBIN	7.7
MALAT1--EPRS	7.7
MALAT1--EP400	7.7
MALAT1--ENTPD4	7.7
MALAT1--ENPP2	7.7
MALAT1--EML4	7.7
MALAT1--ELP2	7.7
MALAT1--EIF4G3	7.7
MALAT1--EIF4G2	7.7
MALAT1--EIF4B	7.7
MALAT1--EIF4A3	7.7

MALAT1--ECT2	7.7
MALAT1--EBF1	7.7
MALAT1--DYRK1A	7.7
MALAT1--DOCK7	7.7
MALAT1--DHCR24	7.7
MALAT1--DGKH	7.7
MALAT1--DERL2	7.7
MALAT1--DEPP1	7.7
MALAT1--CUL9	7.7
MALAT1--CUL1	7.7
MALAT1--CTR9	7.7
MALAT1--CTPS1	7.7
MALAT1--CTDSP2	7.7
MALAT1--CSE1L	7.7
MALAT1--CRIPAK	7.7
MALAT1--CREB3L2	7.7
MALAT1--CPSF6	7.7
MALAT1--COPA	7.7
MALAT1--COL6A3	7.7
MALAT1--COA1	7.7
MALAT1--CNTRL	7.7
MALAT1--CNOT1	7.7
MALAT1--CNKSR2	7.7
MALAT1--CLSTN3	7.7
MALAT1--CLCN7	7.7
MALAT1--CHD2	7.7
MALAT1--CFAP36	7.7
MALAT1--CEP192	7.7
MALAT1--CENPF	7.7
MALAT1--CELSR2	7.7
MALAT1--CELF2-AS2	7.7
MALAT1--CDKL2	7.7
MALAT1--CDH11	7.7
MALAT1--CDH1	7.7
MALAT1--CDC42BPA	7.7
MALAT1--CD63	7.7
MALAT1--CD24	7.7
MALAT1--CCT6A	7.7
MALAT1--CCND3	7.7
MALAT1--CCDC191	7.7
MALAT1--CBLB	7.7
MALAT1--CARD8	7.7
MALAT1--CAPRIN1	7.7
MALAT1--CAMSAP1	7.7
MALAT1--CALD1	7.7

MALAT1--CADM1	7.7
MALAT1--C3ORF80	7.7
MALAT1--C3AR1	7.7
MALAT1--C15ORF40	7.7
MALAT1--BTN3A2	7.7
MALAT1--BTAF1	7.7
MALAT1--BMP2K	7.7
MALAT1--BIVM-ERCC5	7.7
MALAT1--BCLAF1	7.7
MALAT1--BACH2	7.7
MALAT1--ATXN1	7.7
MALAT1--ATP8B1	7.7
MALAT1--ATP13A3	7.7
MALAT1--ATP11A	7.7
MALAT1--ATM	7.7
MALAT1--ASPM	7.7
MALAT1--ARID4A	7.7
MALAT1--ARID2	7.7
MALAT1--ARHGEF7	7.7
MALAT1--ARHGEF12	7.7
MALAT1--ARHGAP1	7.7
MALAT1--API5	7.7
MALAT1--APC	7.7
MALAT1--AP001888.1	7.7
MALAT1--ANP32D	7.7
MALAT1--ANKRD18A	7.7
MALAT1--ANAPC1	7.7
MALAT1--ALDH5A1	7.7
MALAT1--ALDH1A1	7.7
MALAT1--AL512356.1	7.7
MALAT1--AL360020.1	7.7
MALAT1--AL157400.3	7.7
MALAT1--AL139353.1	7.7
MALAT1--AL136295.4	7.7
MALAT1--AL109811.3	7.7
MALAT1--AL031595.2	7.7
MALAT1--AKR1B10	7.7
MALAT1--AIG1	7.7
MALAT1--AHNAK	7.7
MALAT1--AFMID	7.7
MALAT1--AEBP1	7.7
MALAT1--ADGRV1	7.7
MALAT1--ADGRD1	7.7
MALAT1--ADAM17	7.7
MALAT1--ACTB	7.7

MALAT1--ACP6	7.7
MALAT1--ACACA	7.7
MALAT1--AC245884.4	7.7
MALAT1--AC245033.1	7.7
MALAT1--AC118549.1	7.7
MALAT1--AC109517.1	7.7
MALAT1--AC106741.1	7.7
MALAT1--AC100830.1	7.7
MALAT1--AC090227.2	7.7
MALAT1--AC023509.1	7.7
MALAT1--AC021087.5	7.7
MALAT1--AC012676.5	7.7
MALAT1--AC006148.1	7.7
MALAT1--ABHD2	7.7
MALAT1--ABCB1	7.7
MALAT1--ABCA5	7.7
MALAT1--ABCA3	7.7
MALAT1--ABCA1	7.7
MALAT1--A1CF	7.7
MAGI3--AHNAK	7.7
MACF1--UMPS	7.7
MACF1--TXNIP	7.7
MACF1--THRAP3	7.7
MACF1--KDM4A	7.7
MACF1--INPP5B	7.7
MACF1--GTF2I	7.7
MACF1--GDI1	7.7
MACF1--FANCI	7.7
MACF1--FAM120A	7.7
MACF1--EFCAB14	7.7
MACF1--AHNAK	7.7
M6PR--XIST	7.7
LXN--CLOCK	7.7
LUC7L3--RBM15	7.7
LUC7L3--MALAT1	7.7
LUC7L3--HSPA8	7.7
LTBP3--CPXM1	7.7
LTA4H--SLC35E1	7.7
LTA4H--HSPB1	7.7
LSP1--UBASH3B	7.7
LSM14B--ZNF638	7.7
LSAMP--MADD	7.7
LRRIQ1--ZNF664	7.7
LRRCC1--TBL1XR1	7.7
LRRC57--CEP170B	7.7

LRRC52-AS1--GOLGA6L2	7.7
LRRC3B--TAB2	7.7
LRRC37A4P--MALAT1	7.7
LRPPRC--MALAT1	7.7
LRP10--PCNX1	7.7
LRP1--HERC1	7.7
LRMP--PLEC	7.7
LRIG2--MED9	7.7
LRCH3--MALAT1	7.7
LRATD2--NDUFS8	7.7
LPIN1--ALB	7.7
LPGAT1--TPD52	7.7
LOX--RACGAP1	7.7
LONRF1--MLYCD	7.7
LONP2--SYT13	7.7
LONP2--STRIP1	7.7
LONP1--ATP6V1G2-DDX39B	7.7
LNPEP--JPT2	7.7
LMNB2--LAS1L	7.7
LMAN1--STX3	7.7
LINC02234--VPS13B	7.7
LINC02145--CHD9	7.7
LINC00863--NUTM2A	7.7
LINC00630--BAZ2A	7.7
LINC00476--DDX5	7.7
LINC00115--ZNF596	7.7
LIN54--AC048338.2	7.7
LIMCH1--FXYD2	7.7
LIMCH1--CTDSP2	7.7
LILRB5--LILRP1	7.7
LIG3--OSTC	7.7
LIG3--IGKC	7.7
LIG3--IGK@	7.7
LIG3--GOLGA4	7.7
LIG3--AHCYL1	7.7
LHFPL6--NUP98	7.7
LGR4--ITPA	7.7
LGALS8--MALAT1	7.7
LGALS8--LINC01876	7.7
LGALS3BP--NCSTN	7.7
LGALS1--UBAC2	7.7
LFNG--SPECC1L-ADORA2A	7.7
LEPROTL1--SMG1	7.7
LENG8--SERPINB6	7.7

LENG8--MYCL	7.7
LDLR--PRODH2	7.7
LCP1--CENPJ	7.7
LCORL--CTNNB1	7.7
LBR--EIF4A2	7.7
LATS1--AL021546.1	7.7
LASP1--HSPD1	7.7
LARP4B--FTH1	7.7
LARP4B--AP3B2	7.7
LARP1--RPPH1	7.7
LARP1--ENO2	7.7
LARGE2--NSD1	7.7
LAPTM4A--SZT2	7.7
LAMC3--SLC12A4	7.7
LAMC3--KIF23	7.7
LAMC1--TGFBR2	7.7
LAMC1--PICALM	7.7
LAMB1--MALAT1	7.7
LAMB1--HIST1H1D	7.7
LAMB1--DQX1	7.7
LAMA1--VWDE	7.7
LAMA1--SIN3B	7.7
KTN1--PSMA3	7.7
KRT8--MYO6	7.7
KRT18--NEBL	7.7
KRT18--MALAT1	7.7
KRT18--H6PD	7.7
KRT18--BBS9	7.7
KRT15--DHX9	7.7
KPNB1--WDR74	7.7
KPNB1--VPS54	7.7
KPNB1--SND1	7.7
KPNB1--NSUN4	7.7
KPNB1--NEK4	7.7
KPNB1--MALAT1	7.7
KPNB1--GNA12	7.7
KPNB1--CAPN7	7.7
KPNB1--ARID1A	7.7
KPNB1--AKAP13	7.7
KPNA6--MGAT4B	7.7
KPNA2--AC005833.1	7.7
KNSTRN--GUF1	7.7
KNDC1--GPAT4	7.7
KMT5C--COL1A2	7.7
KMT5B--PDGFC	7.7

KMT2E--SPON2	7.7
KMT2E--IGK@	7.7
KMT2E--CACNA2D1	7.7
KMT2D--PKN2	7.7
KMT2D--GPI	7.7
KMT2C--TTLL7	7.7
KMT2C--RUFY3	7.7
KMT2C--MALAT1	7.7
KMT2C--CAST	7.7
KMT2A--SMARCA2	7.7
KMT2A--RAB7A	7.7
KLK12--CRY1	7.7
KLHL9--RGS7	7.7
KLHL7--RNF43	7.7
KLHL7--IRF3	7.7
KLHL42--MALAT1	7.7
KLHL28--MUC4	7.7
KLHDC3--MYO1B	7.7
KLHDC2--SMG1	7.7
KLF7--PPIB	7.7
KLF6--MALAT1	7.7
KLC2--ZNF470	7.7
KIFC1--RNF213	7.7
KIF3B--HERC2	7.7
KIF3A--HSPD1	7.7
KIF2A--HNRNPU	7.7
KIF21A--TLK1	7.7
KIF21A--NRAS	7.7
KIF21A--KIAA1522	7.7
KIF1C--PIGT	7.7
KIF1B--RPPH1	7.7
KIF1B--CTNNBIP1	7.7
KIF1A--MED13	7.7
KIF11--MUC4	7.7
KIAA2026--DSP	7.7
KIAA1671--MALAT1	7.7
KIAA1549--MALAT1	7.7
KIAA1324--TNFRSF11B	7.7
KIAA1324--TFG	7.7
KIAA1211L--B4GALT1	7.7
KIAA1109--TUG1	7.7
KIAA1109--SCFD1	7.7
KIAA1109--MAP1B	7.7
KIAA0895L--PTPRA	7.7
KIAA0895--KIF21A	7.7

KIAA0556--MALAT1	7.7
KIAA0355--KCTD20	7.7
KIAA0100--XIST	7.7
KIAA0100--MALAT1	7.7
KIAA0100--DHCR24	7.7
KHDC4--SOX12	7.7
KDR--SFMBT1	7.7
KDM6A--VCAN	7.7
KDM6A--VANGL2	7.7
KDM5A--PI4KA	7.7
KDM5A--MALAT1	7.7
KDM1A--CNOT7	7.7
KCTD10--SBF2	7.7
KCNQ1OT1--TTN	7.7
KCNQ1OT1--MAP4K4	7.7
KCNMA1--NAGK	7.7
KCNE4--FNDC3A	7.7
KCMF1--PTPRM	7.7
KBTBD8--DSP	7.7
KAT6B--TBL1XR1	7.7
KAT6B--PLEKHO2	7.7
KAT6A--AP1G1	7.7
KANSL1--ARID1A	7.7
JUN--VDAC3	7.7
JPT2--HMCN1	7.7
JMJD1C--P4HA1	7.7
JAK1--PAPSS1	7.7
JAK1--LPAR6	7.7
JAK1--APCDD1	7.7
JAG1--STK36	7.7
IWS1--USP40	7.7
IWS1--PRRC2C	7.7
IVNS1ABP--GLUL	7.7
IVNS1ABP--DEUP1	7.7
ITSN2--TMEM127	7.7
ITSN1--UBC	7.7
ITPR3--SIGLEC1	7.7
ITPR3--ESYT1	7.7
ITPR2--RABGAP1L	7.7
ITPR2--FGFR1OP2	7.7
ITPR1--ARL8B	7.7
ITM2B--RMRP	7.7
ITM2B--ACTB	7.7
ITGB4--DIP2A	7.7
ITGAV--ZFPM2	7.7

ITGAV--MALAT1	7.7
ITGA7--AC004951.1	7.7
ITGA6--TSHZ1	7.7
ITGA4--UBR5	7.7
ITGA1--PISD	7.7
ITGA1--MPHOSPH9	7.7
ISL1--FXR1	7.7
IRF1--SMG1	7.7
IRAK1--SCARNA2	7.7
IQGAP3--SUZ12	7.7
IQGAP1--SIN3A	7.7
IQGAP1--IFI16	7.7
IQGAP1--FTL	7.7
IQCN--DTNA	7.7
IQCA1--TPR	7.7
IQCA1--CMTR1	7.7
IPO9--ATXN10	7.7
IPO5--DLG1	7.7
IPO5--COL1A2	7.7
INVS--FLVCR1	7.7
INTS8--KIAA1522	7.7
INTS7--RMRP	7.7
INTS3--HSP90B1	7.7
INTS10--MALAT1	7.7
INTS1--GALNS	7.7
INO80D--MCM3	7.7
ING5--MALAT1	7.7
IMPAD1--HLCS	7.7
IMMT--PCYT1B	7.7
ILVBL--MKI67	7.7
ILRUN--RBM20	7.7
ILRUN--BICD1	7.7
ILKAP--NF1	7.7
ILF3--SEC24B	7.7
ILF3--FTL	7.7
ILF3--DHX15	7.7
IL6ST--ZFP62	7.7
IL33--ZNF562	7.7
IL20RB--MALAT1	7.7
IL1R1--RPS4X	7.7
IL13RA1--WNK1	7.7
IL10RA--VIM	7.7
IGKV1-5--RBMS1	7.7
IGK@--RBM14	7.7
IGK@--LSP1	7.7

IGK@--HIST1H2BD	7.7
IGK@--HGD	7.7
IGK@--H19	7.7
IGK@--DUX4	7.7
IGK@--COL1A2	7.7
IGK@--CNTRL	7.7
IGK@--CDK6	7.7
IGK@--AC010976.1	7.7
IGHV3-30--CD44	7.7
IGHG1--UGGT2	7.7
IGHG1--SDK1	7.7
IGH@--WWOX	7.7
IGH@--MALT1	7.7
IGH@--CD44	7.7
IGH@--BCL11A	7.7
IGH@--AL137139.2	7.7
IGFN1--APEH	7.7
IGFN1--AL133243.3	7.7
IGFBP2--UBC	7.7
IGF2R--UNC13B	7.7
IGF2R--AK2	7.7
IFT74--BRIP1	7.7
IFT172--AHNAK	7.7
IFNGR2--MALAT1	7.7
IFNAR1--POLR2C	7.7
IFITM3--YAP1	7.7
IFITM1--METAP2	7.7
IDH2--POMP	7.7
IDH2--MAP4	7.7
IDH2--HTATIP2	7.7
IDH1--MALAT1	7.7
IDH1--HECTD4	7.7
ID1--NAP1L1	7.7
ICMT--MALAT1	7.7
ICE2--SLC43A1	7.7
ICE1--SNF8	7.7
IBTK--MALAT1	7.7
IBTK--INHBA	7.7
IBTK--GRN	7.7
IARS2--MALAT1	7.7
IARS--MALAT1	7.7
HYOU1--CASZ1	7.7
HUWE1--SP110	7.7
HUWE1--MALAT1	7.7
HUWE1--LMNB2	7.7

HUWE1--CDCA5	7.7
HUWE1--AK2	7.7
HUWE1--AC106785.1	7.7
HTATSF1--NCOR2	7.7
HSPG2--RPL8	7.7
HSPG2--MALAT1	7.7
HSPE1-MOB4--HNRNPLL	7.7
HSPD1--UMPS	7.7
HSPD1--NEAT1	7.7
HSPD1--HERC2	7.7
HSPD1--H19	7.7
HSPD1--DSP	7.7
HSPD1--COLGALT1	7.7
HSPA9--SCMH1	7.7
HSPA9--PTPRF	7.7
HSPA9--MATR3	7.7
HSPA9--MALAT1	7.7
HSPA8--PFKM	7.7
HSPA8--PDIA4	7.7
HSPA8--LTBR	7.7
HSPA5--LMNA	7.7
HSPA5--DST	7.7
HSPA5--ARFGAP1	7.7
HSPA4--HNRNPU	7.7
HSPA1B--DIAPH1	7.7
HSPA1A--RPS6KC1	7.7
HSP90B1--TGOLN2	7.7
HSP90B1--MALAT1	7.7
HSP90B1--CDYL	7.7
HSP90B1--BCR	7.7
HSP90AB1--MAP1B	7.7
HSP90AB1--MALAT1	7.7
HSP90AB1--FN1	7.7
HSP90AA1--SYNE2	7.7
HSP90AA1--BCL6	7.7
HSDL2--TMEM86A	7.7
HSDL1--EIF2A	7.7
HSD17B4--UBR4	7.7
HSD17B4--RARS	7.7
HSD17B4--PUS7L	7.7
HSD11B2--SSH1	7.7
HPF1--AC122713.1	7.7
HP1BP3--DZANK1	7.7
HP1BP3--DLGAP5	7.7

HOXD8--RNF213	7.7
HOXD8--HIPK2	7.7
HOOK3--SLC16A1	7.7
HOOK3--LINC00632	7.7
HNRNPUL2-BSCL2--PTPRF	7.7
HNRNPUL2-BSCL2--KANSL1	7.7
HNRNPUL1--GALNT7	7.7
HNRNPUL1--AC021087.5	7.7
HNRNPU--UBC	7.7
HNRNPU--TANC1	7.7
HNRNPU--SSR1	7.7
HNRNPU--SLC25A6	7.7
HNRNPU--RC3H1	7.7
HNRNPU--CNTNAP2	7.7
HNRNPU--AEBP1	7.7
HNRNPU--AC016588.2	7.7
HNRNPR--ITPKB	7.7
HNRNPL--AFG3L1P	7.7
HNRNPK--CYBA	7.7
HNRNPH3--MFN1	7.7
HNRNPH3--AL162171.3	7.7
HNRNPH1--WDR59	7.7
HNRNPH1--ODF2L	7.7
HNRNPH1--MPHOSPH9	7.7
HNRNPH1--IL6ST	7.7
HNRNPH1--CLIC1	7.7
HNRNPH1--ANKRA2	7.7
HNRNPF--CD2AP	7.7
HNRNPDL--SRRM2	7.7
HNRNPDL--ONECUT2	7.7
HNRNPDL--CSNK1D	7.7
HNRNPD--DDX1	7.7
HNRNPC--RALGAPA2	7.7
HNRNPC--MALAT1	7.7
HNRNPC--ICE2	7.7
HNRNPC--HERPUD1	7.7
HNRNPA3--ADHFE1	7.7
HNRNPA2B1--ZFYVE26	7.7
HNRNPA2B1--WWOX	7.7
HNRNPA2B1--SPTAN1	7.7
HNRNPA2B1--SCN8A	7.7
HNRNPA2B1--RMRP	7.7
HNRNPA2B1--MALAT1	7.7

HNRNPA2B1--DENND4B	7.7
HNRNPA2B1--AC093512.2	7.7
HNRNPA1--PRKAA2	7.7
HNRNPA1--GFPT1	7.7
HMGN1--PCSK7	7.7
HMGN1--EEF1A1	7.7
HMGN1--COL5A2	7.7
HMGN1--APP	7.7
HMGCS1--KMT2C	7.7
HMGB3--WDR83	7.7
HMGB2--PHF14	7.7
HMGB2--COL1A2	7.7
HMGB1--UBE4B	7.7
HMGB1--FCN1	7.7
HMGB1--AC008725.1	7.7
HMCN1--VIM	7.7
HMCN1--MTCH2	7.7
HMCN1--HMGB3	7.7
HMCN1--COL5A2	7.7
HLTF--PTK2	7.7
HLTF--GTF3C1	7.7
HLCS--MUC16	7.7
HK1--MALAT1	7.7
HIST1H4L--MPRIP	7.7
HIST1H3C--ZNF720	7.7
HIST1H2BJ--TPM2	7.7
HIST1H2BH--PRSS16	7.7
HIST1H2BF--ZNF592	7.7
HIST1H2BC--ZNRF1	7.7
HIST1H2AL--ISYNA1	7.7
HIST1H2AB--TPR	7.7
HIST1H1E--PSAP	7.7
HIST1H1E--POLR2A	7.7
HIST1H1E--KHNYN	7.7
HIPK3--ATP2A2	7.7
HIPK2--MALAT1	7.7
HIPK1--TTBK2	7.7
HIPK1--NEAT1	7.7
HIPK1--HSP90AB1	7.7
HIGD2A--VPS9D1-AS1	7.7
HIF1A--CACNA1C	7.7
HID1--ADAR	7.7
HGS--SCG5	7.7
HERC4--GALE	7.7

HERC2--SIGMAR1	7.7
HERC2--PROX1	7.7
HERC2--MALAT1	7.7
HERC2--IGK@	7.7
HERC2--ATXN2	7.7
HERC2--APP	7.7
HERC2--AP4E1	7.7
HERC2--AL138966.2	7.7
HERC1--MALAT1	7.7
HERC1--HELLPAR	7.7
HELZ--TPI1	7.7
HELZ--RNMT	7.7
HELLPAR--TPM3	7.7
HELB--CDC6	7.7
HECTD4--PTCH1	7.7
HECTD4--MALAT1	7.7
HECTD1--URB1	7.7
HECTD1--TLK1	7.7
HECTD1--MALAT1	7.7
HECTD1--AC004805.1	7.7
HDLBP--MAX	7.7
HDGFL2--MIGA2	7.7
HDGF--RBM3	7.7
HDC--TTC14	7.7
HDAC2--ZNF141	7.7
HDAC2--NSD3	7.7
HDAC1--SMARCA4	7.7
HDAC1--PRORP	7.7
HDAC1--KPNA6	7.7
HCCS--SCARNA5	7.7
HBP1--CDK6	7.7
HAUS1--REL	7.7
HASPIN--ELOVL7	7.7
HASPIN--AKAP9	7.7
HAS2--ZNF572	7.7
HADH--NKD1	7.7
HACD4--RSBN1	7.7
HACD3--AC245033.1	7.7
H3F3A--SPEN	7.7
H1F0--HELLS	7.7
H1F0--ALDH7A1	7.7
H19--ZNF639	7.7
H19--ZNF496	7.7
H19--NUP98	7.7
H19--MTHFD1	7.7

H19--MTF1	7.7
H19--MSI2	7.7
H19--HIVEP1	7.7
GUCY1A2--XIST	7.7
GUCD1--ATRAID	7.7
GTPBP1--MOV10	7.7
GTF3C4--RPS11	7.7
GTF3C2--DDX23	7.7
GTF3C1--ACADVL	7.7
GTF2I--PAWR	7.7
GTF2I--NEK9	7.7
GTF2I--MALAT1	7.7
GTF2I--HDLBP	7.7
GTF2I--APH1A	7.7
GSTP1--MALAT1	7.7
GSR--SMG1	7.7
GSPT1--RPS4X	7.7
GSE1--TAF1	7.7
GSE1--MTMR3	7.7
GSE1--MALAT1	7.7
GRSF1--EEF1D	7.7
GRK2--TCN2	7.7
GRIPAP1--GSTK1	7.7
GRHPR--C1QC	7.7
GRAMD2B--USP24	7.7
GPR65--XIST	7.7
GPR108--FN1	7.7
GPI--STAB2	7.7
GPI--EEF2	7.7
GPC6--PRRC2C	7.7
GPBP1L1--DDX58	7.7
GPBP1--COMMD10	7.7
GPATCH2--DDX21	7.7
GOSR2--RANBP2	7.7
GORASP2--BLVRA	7.7
GON4L--SDR9C7	7.7
GOLM1--SDHB	7.7
GOLM1--DA750114	7.7
GOLGB1--MALAT1	7.7
GOLGB1--ITGA3	7.7
GOLGA6L10--XIST	7.7
GOLGA4--LRRFIP2	7.7
GOLGA3--APPBP2	7.7
GOLGA3--AC104472.3	7.7
GNPTAB--MALAT1	7.7

GNPNAT1--FMNL2	7.7
GNL3L--PPIP5K2	7.7
GNL3L--FTL	7.7
GNL3--DSG2	7.7
GNE--RNF213	7.7
GNE--EDEM3	7.7
GNB4--CTNND1	7.7
GNB1--NOP56	7.7
GNB1--AL031777.3	7.7
GNAS--SMG1	7.7
GNAS--AD000090.1	7.7
GMNN--NID1	7.7
GLUL--XPO6	7.7
GLUL--DOCK10	7.7
GLUL--CEP350	7.7
GLRX2--ATXN7L3B	7.7
GLG1--KIAA1324	7.7
GLB1L--EEF2	7.7
GJA1--MALAT1	7.7
GJA1--CD74	7.7
GINS1--MALAT1	7.7
GIGYF2--MALAT1	7.7
GGA1--EP300	7.7
GFPT1--UBA1	7.7
GFPT1--LAPTM4A	7.7
GFM1--MALAT1	7.7
GDPD3--GNB1	7.7
GDNF--CCT3	7.7
GDI2--RPL8	7.7
GDI2--MMP24OS	7.7
GDI2--CSDE1	7.7
GDI2--C21ORF91	7.7
GDI1--DCP2	7.7
GDF7--EEF1A1	7.7
GDE1--AHNAK	7.7
GDAP1--TM9SF2	7.7
GCSH--RANBP2	7.7
GCN1--NDUFB11	7.7
GCN1--MALAT1	7.7
GCLC--SMG1	7.7
GBF1--SENP2	7.7
GBF1--AC004797.1	7.7
GBA2--FHL1	7.7
GBA--LINC00205	7.7
GBA--AC005534.1	7.7

GASK1B--GPI	7.7
GAS7--PSD3	7.7
GAS5--RERE	7.7
GARS--LRP10	7.7
GARS--LINC00472	7.7
GAPVD1--RNF167	7.7
GAPDH--XRCC6	7.7
GAPDH--MALAT1	7.7
GALNT3--PRRC2B	7.7
GALNT2--ABHD3	7.7
GALNT18--ANKRD17	7.7
GALNT13--GDE1	7.7
GALM--KPNB1	7.7
GALC--NEAT1	7.7
GABRA3--TGFBR3	7.7
GABPB1-AS1--NEB	7.7
GAB2--ARRDC3	7.7
G3BP1--MYH10	7.7
FYTTD1--LRCH3	7.7
FXR1--SLC29A1	7.7
FXR1--RMRP	7.7
FXR1--NUTF2	7.7
FXR1--CCDC59	7.7
FUS--RTBDN	7.7
FUS--NXF1	7.7
FUS--AL021155.5	7.7
FUBP1--USP33	7.7
FUBP1--MALAT1	7.7
FTX--MUC4	7.7
FTSJ1--PCM1	7.7
FTL--RPL11	7.7
FTL--RN7SL1	7.7
FTL--RBM6	7.7
FTL--MALAT1	7.7
FTL--DPYSL2	7.7
FTH1--WASHC5	7.7
FTH1--WAC	7.7
FTH1--VEGFA	7.7
FTH1--PRLHR	7.7
FTH1--PAH	7.7
FTH1--NEAT1	7.7
FTH1--NDEL1	7.7
FTH1--MRM3	7.7
FTH1--MDM2	7.7
FTH1--MALAT1	7.7

FTH1--LHPP	7.7
FTH1--HMGN2	7.7
FTH1--HIPK2	7.7
FTH1--HECTD3	7.7
FTH1--COA3	7.7
FTH1--CHST15	7.7
FTH1--CACNA2D1	7.7
FTH1--BDP1	7.7
FTH1--ADAM15	7.7
FSTL1--MALAT1	7.7
FST--MALAT1	7.7
FRMD3--XRCC6	7.7
FRK--MALAT1	7.7
FRG1BP--AL161457.2	7.7
FRG1--AL161457.2	7.7
FREM2--PPL	7.7
FPR2--DDX3X	7.7
FOXRED2--VWF	7.7
FOXP1--ZNFX1	7.7
FOXP1--RNF213	7.7
FOXP1--RABEP1	7.7
FOXP1--GOLGA4	7.7
FOXP1--EGR1	7.7
FOXM1--ZC3H13	7.7
FOXK2--ZNF652	7.7
FOXJ3--RMRP	7.7
FOS--MRTFA	7.7
FO538757.1--AC009533.1	7.7
FNDC3B--ROBO1	7.7
FNDC3A--DGKH	7.7
FNDC3A--CD2AP	7.7
FNDC3A--ACTB	7.7
FNBP1L--MALAT1	7.7
FN1--ZFAT	7.7
FN1--WDR74	7.7
FN1--VDAC2	7.7
FN1--UGDH-AS1	7.7
FN1--UBXN7	7.7
FN1--SART3	7.7
FN1--PABPC1	7.7
FN1--NPM1	7.7
FN1--IGF1R	7.7
FN1--HNRNPK	7.7
FN1--FNTA	7.7

FN1--FBXW2	7.7
FN1--ERBIN	7.7
FN1--CPED1	7.7
FN1--CAST	7.7
FMR1--TASOR2	7.7
FMR1--IREB2	7.7
FMC1-LUC7L2--ZNF189	7.7
FLOT1--MON1B	7.7
FLNC--CHGB	7.7
FLNA--MALAT1	7.7
FLNA--HIST1H2BH	7.7
FLCN--KIAA2026	7.7
FKBP14--ZBED1	7.7
FGG--SLC38A10	7.7
FGFR2--ZMYND11	7.7
FGFR1OP--IPO5	7.7
FGFR1--UBC	7.7
FGD1--MALAT1	7.7
FEN1--HIST1H2AM	7.7
FEM1B--RET	7.7
FEM1B--MALAT1	7.7
FDPS--PARP14	7.7
FCHSD2--TMEM106B	7.7
FCF1--CHCHD5	7.7
FCF1--AC009220.3	7.7
FBXW11--SEMA3C	7.7
FBXO34--MUC4	7.7
FBXO28--ANGEL2	7.7
FBXL3--CDK4	7.7
FBXL18--YBX3	7.7
FBXL18--UTRN	7.7
FBXL18--F5	7.7
FBRSL1--MALAT1	7.7
FBN1--FOXP1	7.7
FBLN1--PI4KA	7.7
FBLN1--MALAT1	7.7
FAT3--BAZ1A	7.7
FAT1--SLC38A2	7.7
FAT1--RAPGEF2	7.7
FAT1--CADPS	7.7
FAT1--ANKRD17	7.7
FASTKD2--LNPEP	7.7
FASN--MALAT1	7.7
FASN--DSP	7.7
FARP2--NUP155	7.7

FARP1--ZC3HAV1	7.7
FAP--RMRP	7.7
FANCI--ZNF396	7.7
FANCA--CNOT1	7.7
FAM50A--MALAT1	7.7
FAM50A--GOLM1	7.7
FAM50A--BOLA3-AS1	7.7
FAM47DP--FAM47C	7.7
FAM32A--CPLX1	7.7
FAM210B--CNOT1	7.7
FAM20C--MUC4	7.7
FAM182B--AC209154.1	7.7
FAM126A--MALAT1	7.7
FAM120B--STXBP5L	7.7
FAM120B--PSEN1	7.7
FAM120B--AP4B1	7.7
FAM118B--PRRC2A	7.7
FAM117A--AGMO	7.7
FAM110B--LEMD3	7.7
FAM104B--FZD7	7.7
FAM102A--SMG1	7.7
FAF1--SSBP2	7.7
FAF1--PPP1CB	7.7
F5--RPN1	7.7
F3--RIMS1	7.7
F2--DDX17	7.7
F12--IDH1	7.7
EZR--FLNA	7.7
EYA4--PCID2	7.7
EXTL3--MALAT1	7.7
EXTL2--KIAA0100	7.7
EXOC2--MALAT1	7.7
EXOC2--DUSP22	7.7
EXOC2--ANKH	7.7
EVC--SCARNA5	7.7
ETV6--MALAT1	7.7
ETV6--EEF1A1	7.7
ETV6--ATIC	7.7
ETS2--AL391380.1	7.7
ETNK1--LRRC14	7.7
ESCO1--DDX46	7.7
ESCO1--B3GALNT2	7.7
ERRFI1--SAMD9	7.7
ERRFI1--PAICS	7.7
ERRFI1--ANKRD11	7.7

ERO1A--SLC38A3	7.7
ERO1A--KIF14	7.7
ERLIN1--SCARB2	7.7
ERCC6L2--MALAT1	7.7
ERCC6L2--HECTD1	7.7
ERBIN--EID1	7.7
ERBB4--PERM1	7.7
ERBB3--MTHFD1	7.7
ERBB3--FZD6	7.7
ERAL1--WASHC4	7.7
EPSTI1--IGK@	7.7
EPS15--FAF1	7.7
EPM2AIP1--MALAT1	7.7
EPCAM--PCDHGA11	7.7
EPCAM--NR2C2	7.7
EPCAM--MALAT1	7.7
EPB41L5--RMRP	7.7
EPB41L5--APOC1	7.7
EPB41L3--SRXN1	7.7
EPB41L3--MALAT1	7.7
EPB41L3--INTS7	7.7
EP400--ATP9A	7.7
EP300--TCEA3	7.7
EP300--SOS1-IT1	7.7
EP300--GNA13	7.7
EP300--ERO1B	7.7
ENTPD6--AC005534.1	7.7
ENTPD1--MALAT1	7.7
ENPP2--ADGRV1	7.7
ENPP1--COQ8A	7.7
ENO1--ST3GAL2	7.7
ENO1--PALM2-AKAP2	7.7
ENO1--DENND5A	7.7
ENGASE--MALAT1	7.7
ENC1--CTSD	7.7
ENAH--SP100	7.7
ENAH--PRKDC	7.7
EMP3--GSN	7.7
EMP2--PHIP	7.7
EMP1--ATN1	7.7
EML5--EIF3D	7.7
EML4--POSTN	7.7
EML3--PLAA	7.7
EMC1--MALAT1	7.7
ELOA--EEF1A1	7.7

ELMO2--FMNL2	7.7
ELMO1--IRF2BP2	7.7
ELL2--AGAP1	7.7
ELK3--ATL3	7.7
ELF3--RAI1	7.7
ELAC2--PRKAA2	7.7
EIF5B--SPTBN1	7.7
EIF5B--CKAP2L	7.7
EIF5--MALAT1	7.7
EIF4G3--RBM26	7.7
EIF4G3--ARID1B	7.7
EIF4G1--GABPB1-IT1	7.7
EIF4ENIF1--NECTIN1	7.7
EIF4B--XRCC6	7.7
EIF4B--LCAT	7.7
EIF4B--ABCG1	7.7
EIF4A2--XIST	7.7
EIF4A2--HSPH1	7.7
EIF4A2--GMPR2	7.7
EIF4A2--CACNA2D1	7.7
EIF4A2--C19ORF44	7.7
EIF4A1--SEC14L1	7.7
EIF4A1--MALAT1	7.7
EIF4A1--KRT8	7.7
EIF4A1--DUSP6	7.7
EIF3H--TRPS1	7.7
EIF3H--SKI	7.7
EIF3E--XIST	7.7
EIF3B--MALAT1	7.7
EIF3A--ZNF292	7.7
EIF3A--SLTM	7.7
EIF3A--SLC12A7	7.7
EIF3A--MALAT1	7.7
EIF2S3--HSP90AA1	7.7
EIF2AK4--AC016876.2	7.7
EIF2AK1--HNRNPUL1	7.7
EIF2AK1--HMGB1	7.7
EIF2A--OTOF	7.7
EHMT1--ZDBF2	7.7
EHBP1L1--LHFPL4	7.7
EHBP1L1--CD276	7.7
EGFR--MALAT1	7.7
EFHD2--SIPA1L1	7.7
EFEMP1--MTO1	7.7
EFCAB14--C14ORF93	7.7

EEF2--MALAT1	7.7
EEF2--KIFAP3	7.7
EEF1B2--TMED4	7.7
EEF1AKMT1--COL4A1	7.7
EEF1A1--ZNF133	7.7
EEF1A1--XIST	7.7
EEF1A1--WDR74	7.7
EEF1A1--VPS13A	7.7
EEF1A1--UBE3C	7.7
EEF1A1--SYT7	7.7
EEF1A1--RPPH1	7.7
EEF1A1--RMRP	7.7
EEF1A1--PIEZO1	7.7
EEF1A1--MYH9	7.7
EEF1A1--MIR100HG	7.7
EEF1A1--MED13	7.7
EEF1A1--FOXP1	7.7
EEF1A1--FN1	7.7
EEF1A1--FLNA	7.7
EEF1A1--F10	7.7
EEF1A1--EPC1	7.7
EEF1A1--BAZ2B	7.7
EEF1A1--ACOT9	7.7
EED--UBC	7.7
EDRF1--AHNAK	7.7
EDF1--SREBF2	7.7
EDC3--XIST	7.7
ECPAS--ATP2A2	7.7
ECM1--ATN1	7.7
ECD--POMK	7.7
ECD--NT5E	7.7
EBNA1BP2--WBP1L	7.7
EBF1--MALAT1	7.7
EBF1--GNAS	7.7
EBF1--CNN2	7.7
EBF1--API5	7.7
EBAG9--SMG1	7.7
DZIP1L--NCKAP1	7.7
DYRK2--AL590666.2	7.7
DYRK1A--EFHC1	7.7
DYNLL1--SYNPO	7.7
DYNC1LI2--DFFA	7.7
DYNC1LI2--CAVIN1	7.7
DYNC1I1--COL3A1	7.7
DYNC1H1--WFDC2	7.7

DYNC1H1--RNPS1	7.7
DYNC1H1--NEB	7.7
DYNC1H1--ISCA1	7.7
DYNC1H1--ENAH	7.7
DYNC1H1--CASC3	7.7
DYNC1H1--AXIN2	7.7
DYNC1H1--APP	7.7
DVL3--MALAT1	7.7
DVL3--COL1A2	7.7
DUXAP8--XIST	7.7
DUSP22--KDM6A	7.7
DUOX1--BLOC1S5-TXNDC5	7.7
DTNB--CHM	7.7
DTNA--SERPINA1	7.7
DTNA--PTK2	7.7
DSTYK--SERINC5	7.7
DST--TMEM230	7.7
DST--RMRP	7.7
DST--MAZ	7.7
DST--AK2	7.7
DSP--ZXDC	7.7
DSP--YY1AP1	7.7
DSP--RMRP	7.7
DSP--MALAT1	7.7
DSP--ETV6	7.7
DSP--ATG4B	7.7
DSP--AHNAK	7.7
DSG2--CASC19	7.7
DROSHA--ELK3	7.7
DRG1--HIST1H4E	7.7
DRAXIN--NDUFS2	7.7
DRAIC--NCAPG2	7.7
DPYSL2--UROD	7.7
DPY19L1--IGH@	7.7
DPP3--MALAT1	7.7
DPH3--ANKRD36	7.7
DOP1A--MDH2	7.7
DOP1A--MALAT1	7.7
DOCK7--ZMIZ1	7.7
DOCK7--PEAK1	7.7
DOCK10--EPB41L4B	7.7
DOCK1--MALAT1	7.7
DOCK1--HSPA8	7.7
DOCK1--ETV1	7.7

DOCK1--AKR1E2	7.7
DNTTIP2--DYRK2	7.7
DNMT1--MAP3K4	7.7
DNM3--MALAT1	7.7
DNER--OAT	7.7
DNASE1L1--ZNF428	7.7
DNAJC5--TOM1L2	7.7
DNAJC5--MALAT1	7.7
DNAJC3--MUC20	7.7
DNAJC21--MTPAP	7.7
DNAJC2--COX20	7.7
DNAJC19--MALAT1	7.7
DNAJB6--OSGIN2	7.7
DNAJA3--MALAT1	7.7
DNAH5--HCFC2	7.7
DNAH11--EEF1A1	7.7
DNA2--CHD9	7.7
DMXL2--MALAT1	7.7
DMXL1--TPD52L2	7.7
DLST--FKBP11	7.7
DLL3--KANSL1	7.7
DLGAP5--EMC1	7.7
DLG1--PUS10	7.7
DLG1--N4BP1	7.7
DLG1--DDX24	7.7
DLEU2--NINL	7.7
DLD--PRKAA2	7.7
DLD--MSI2	7.7
DLD--BAZ2B	7.7
DLAT--METTL21A	7.7
DLAT--CEMIP2	7.7
DKC1--SAMD5	7.7
DKC1--ITPRID2	7.7
DKC1--AP000553.5	7.7
DIP2C--MALAT1	7.7
DICER1--UBE2I	7.7
DICER1--KMT2A	7.7
DIAPH3--MALAT1	7.7
DIAPH1--IGH@	7.7
DIABLO--GRHL2	7.7
DHX57--GPN3	7.7
DHX40--EIF4A2	7.7
DHX35--NDRG1	7.7
DHX30--VWF	7.7
DHX15--DAPK3	7.7

DHRS13--STIL	7.7
DGCR8--MCM7	7.7
DESI1--ZNF30	7.7
DEPDC5--FTL	7.7
DEPDC1B--ELOVL7	7.7
DEPDC1B--AL157935.2	7.7
DEPDC1--CRTC2	7.7
DENR--MALAT1	7.7
DENR--AL450263.2	7.7
DENND5B--COL5A1	7.7
DENND5A--CBLB	7.7
DENND3--AC124312.3	7.7
DELE1--SASH1	7.7
DEK--SMG1	7.7
DEK--MALAT1	7.7
DEK--GLUL	7.7
DDX60L--IGK@	7.7
DDX6--SSBP1	7.7
DDX6--PASK	7.7
DDX54--UBXN7	7.7
DDX5--RBM14	7.7
DDX5--DARS	7.7
DDX5--COL6A3	7.7
DDX3Y--DDX3P1	7.7
DDX3X--MALAT1	7.7
DDX3X--AC023509.1	7.7
DDX39B--ARHGAP12	7.7
DDX27--TRIO	7.7
DDX23--E4F1	7.7
DDX21--MALAT1	7.7
DDX19A--SNRNP200	7.7
DDX17--RALGAPA2	7.7
DDX17--MALAT1	7.7
DDX17--HBB	7.7
DDX11--PRDM15	7.7
DDR1--YLPM1	7.7
DDR1--RIMS2	7.7
DDOST--KRT8	7.7
DDHD2--NEB	7.7
DDHD1--MAPKAPK5	7.7
DDB1--XIST	7.7
DDB1--CYP17A1	7.7
DCUN1D1--MUC4	7.7
DCTPP1--C21ORF91	7.7
DCTN1--DPY19L4	7.7

DCP1A--RPLP1	7.7
DCLK2--MALAT1	7.7
DCHS2--ZSWIM2	7.7
DCHS1--KLHL24	7.7
DCDC1--PTCH2	7.7
DCDC1--ADAMTS1	7.7
DCBLD2--SYNCRIP	7.7
DCBLD2--CGNL1	7.7
DCAF6--SEC61A1	7.7
DCAF16--EIF4G1	7.7
DCAF13--PTMA	7.7
DBT--ARAP1	7.7
DBI--FHAD1	7.7
DAPK1--PIK3R3	7.7
DAPK1--AKAP9	7.7
DAP3--MON2	7.7
DAP--SELENOF	7.7
DAG1--EIF3E	7.7
DAG1--CCNC	7.7
DAAM1--CLCN7	7.7
CYP17A1--MALAT1	7.7
CYLD--SERPINA1	7.7
CYFIP1--MALAT1	7.7
CYB5A--SEPTIN9	7.7
CXCL8--NAMPT	7.7
CXADR--DHCR24	7.7
CUX1--AKR1C3	7.7
CTSK--SUV39H2	7.7
CTPS1--MALAT1	7.7
CTNND1--ZNF577	7.7
CTNNB1--ARF5	7.7
CTH--ASPM	7.7
CTDSPL2--DDX6	7.7
CTCF--GRHL2	7.7
CTCF--ATF4	7.7
CSTB--VIM	7.7
CSRP1--XIST	7.7
CSNK1G3--VMP1	7.7
CSNK1G1--DLAT	7.7
CSNK1E--EFHC1	7.7
CSNK1D--RAD21	7.7
CSE1L--SPOCD1	7.7
CSE1L--SMG7	7.7
CSE1L--PTPN1	7.7
CSE1L--MALAT1	7.7

CSDE1--RAN	7.7
CSDE1--NEB	7.7
CSDE1--LGALS3BP	7.7
CSDE1--ATP6V1A	7.7
CS--AGO1	7.7
CROCC--NAPG	7.7
CRIPAK--TNRC18	7.7
CREBBP--PAXIP1-AS2	7.7
CREBBP--LASP1	7.7
CREBBP--FOXP2	7.7
CREBBP--COL1A2	7.7
CREB3L2--HSDL2	7.7
CPSF6--ESPN	7.7
CPSF6--DIP2A	7.7
CPOX--COL1A1	7.7
CPLX2--TMEM50A	7.7
CPLX2--KMT2E	7.7
CPLANE1--MALAT1	7.7
CPED1--RAB1B	7.7
CPD--EFR3A	7.7
COX6B1--TMTC2	7.7
COX6B1--SRRM2	7.7
COX6A1--RPL7L1	7.7
COX6A1--BMP2K	7.7
COX20--BIRC6	7.7
COX11--ASPN	7.7
COX10--PABPC1	7.7
COTL1--ZBTB21	7.7
COQ8A--DVL3	7.7
COPS3--EEF2	7.7
COPA--VPS37D	7.7
COPA--SULF2	7.7
COPA--HSP90B1	7.7
COMMD7--CCDC50	7.7
COLGALT1--AL031777.3	7.7
COLEC12--MALAT1	7.7
COL6A3--MORF4L1	7.7
COL6A3--MAP1B	7.7
COL6A3--LINC02012	7.7
COL4A5--FN1	7.7
COL4A2--MALAT1	7.7
COL3A1--WDR74	7.7
COL3A1--TAX1BP1	7.7
COL3A1--RPLP1	7.7

COL3A1--EGFR	7.7
COL3A1--BCL2	7.7
COL3A1--AC093010.3	7.7
COL1A2--TMBIM6	7.7
COL1A2--RPL36A-HNRNPH2	7.7
COL1A2--PSMA3-AS1	7.7
COL1A2--NISCH	7.7
COL1A2--MTRNR2L12	7.7
COL1A2--MALAT1	7.7
COL1A2--HNRNPH1	7.7
COL1A2--CABIN1	7.7
COL1A1--WDR70	7.7
COL1A1--STT3B	7.7
COL1A1--SHOC2	7.7
COL1A1--RPL15	7.7
COL1A1--LRPPRC	7.7
COL1A1--KLHL42	7.7
COL1A1--HSD17B7	7.7
COL1A1--H19	7.7
COL1A1--EXOSC7	7.7
COL1A1--ENO2	7.7
COL1A1--DDX27	7.7
COL1A1--ASCC3	7.7
COL12A1--TPT1	7.7
COG3--CALR	7.7
COASY--UBR4	7.7
COASY--TAF15	7.7
COA1--KMT2C	7.7
CNPY4--GOLGA8J	7.7
CNPY2--MYSM1	7.7
CNOT6--MAPKAPK2	7.7
CNOT6--LRPAP1	7.7
CNOT1--UBC	7.7
CNOT1--SUGP2	7.7
CNOT1--SMG1	7.7
CNOT1--PHKA2	7.7
CNOT1--KNL1	7.7
CNNM4--MUC4	7.7
CNN3--SON	7.7
CNKSR3--DUSP6	7.7
CNIH4--HOXB3	7.7
CNBP--ACTB	7.7
CMTM6--RBAK	7.7
CMTM4--KMT2D	7.7

CLYBL--IGH@	7.7
CLU--SLIT2	7.7
CLTC--VPS13A	7.7
CLTC--TMEM94	7.7
CLTC--RNF7	7.7
CLTC--PLEC	7.7
CLTC--LMTK2	7.7
CLSPN--ANKRD7	7.7
CLPTM1--MALAT1	7.7
CLPTM1--DUS4L	7.7
CLMP--COL5A2	7.7
CLIP1--CD63	7.7
CLIC4--FBN1	7.7
CLIC4--C1GALT1	7.7
CLDN4--SERPINA1	7.7
CLASP2--B2M	7.7
CKAP5--TPT1	7.7
CKAP5--DST	7.7
CKAP2--MAN2B2	7.7
CIT--BICDL1	7.7
CIITA--SDHA	7.7
CIC--RTL8C	7.7
CIAO1--VASN	7.7
CIAO1--PRPF18	7.7
CHSY1--MALAT1	7.7
CHST11--SEMA6C	7.7
CHST11--MALAT1	7.7
CHST11--HMCN1	7.7
CHST11--FN1	7.7
CHST11--COL6A1	7.7
CHMP5--AC114402.2	7.7
CHMP1A--UBB	7.7
CHML--DTX3L	7.7
CHM--TTLL7	7.7
CHID1--MALAT1	7.7
CHGB--GTF2I	7.7
CHGB--CFAP221	7.7
CHGA--MALAT1	7.7
CHFR--MALAT1	7.7
CHFR--AC011448.1	7.7
CHD9--PSAP	7.7
CHD9--LRRC49	7.7
CHD9--GGA1	7.7
CHD6--COL27A1	7.7
CHD3--MALAT1	7.7

CHD2--PPP4R3B	7.7
CHD1--CDC42	7.7
CHD1--AC000123.3	7.7
CHAF1B--GNAS	7.7
CFLAR--AC068896.1	7.7
CFL1--ARHGAP1	7.7
CFDP1--ZCCHC8	7.7
CFAP70--AL451062.3	7.7
CFAP20--ABCC3	7.7
CERT1--ABCC2	7.7
CEP97--TNFAIP3	7.7
CEP85L--XRCC5	7.7
CEP76--LAPTM4A	7.7
CEP70--CCT8	7.7
CEP350--TLN1	7.7
CEP350--KHSRP	7.7
CEP350--IGH@	7.7
CEP295--TFRC	7.7
CEP290--MALAT1	7.7
CEP250--PAXBP1	7.7
CEP250--MALAT1	7.7
CEP250--CCNT2	7.7
CEP192--DIS3	7.7
CEP131--TACC1	7.7
CEP128--CSNK1D	7.7
CEP120--KDM5C	7.7
CEP104--HERC2	7.7
CENPU--AL355987.2	7.7
CENPO--CPNE1	7.7
CENPJ--ZNF217	7.7
CENPF--PGD	7.7
CENPF--NEO1	7.7
CENPF--CNPY4	7.7
CENPF--AC117386.2	7.7
CEMIP2--RPL27A	7.7
CELSR2--COL1A2	7.7
CDYL--AC091230.1	7.7
CDV3--CARMIL1	7.7
CDR2L--SMAD1	7.7
CDON--AP004607.3	7.7
CDKN2B-AS1--POLR3A	7.7
CDKL5--RGL2	7.7
CDK6--SOS1	7.7
CDK6--KMT2A	7.7
CDK6--AC079594.2	7.7

CDK5RAP2--LUZP1	7.7
CDK5RAP2--HNRNPA3	7.7
CDK2--EEF1A1	7.7
CDK13--MALAT1	7.7
CDH2--FTL	7.7
CDH1--NDUFV1	7.7
CDCP1--ZDHHC7	7.7
CDC42BPG--NACA	7.7
CDC42BPB--LAS1L	7.7
CDC42BPA--RNF213	7.7
CDC42BPA--BMP2K	7.7
CDC27--XIST	7.7
CDC27--PNRC2	7.7
CDC23--MALAT1	7.7
CD81--USP34	7.7
CD81--FTL	7.7
CD55--NAMPT	7.7
CD55--HUWE1	7.7
CD46--OTUD7B	7.7
CD46--HERC2	7.7
CD46--ATP11B	7.7
CD44--RERE	7.7
CD2AP--USP53	7.7
CD2AP--COPG1	7.7
CD24--RHCE	7.7
CD209--MALAT1	7.7
CD164--GDA	7.7
CCT8--SPOCK3	7.7
CCT8--SFPQ	7.7
CCT6A--NCOR1	7.7
CCT5--ZFAND6	7.7
CCT5--KDM4A	7.7
CCT5--BRIX1	7.7
CCT4--SQSTM1	7.7
CCT2--TPT1	7.7
CCSER2--KIDINS220	7.7
CCP110--HMGN2	7.7
CCNT1--MALAT1	7.7
CCNI--SLC25A6	7.7
CCNI--ACTB	7.7
CCNG2--KTN1	7.7
CCNE1--AC027097.2	7.7
CCND3--MALAT1	7.7
CCND3--IGH@	7.7
CCNB1IP1--CAPZB	7.7
CCNB1--NPIPB11	7.7
CCN1--TTN	7.7
CCM2--MALAT1	7.7
CCDC88A--MYO9A	7.7
CCDC88A--ATRX	7.7
CCDC82--RO60	7.7
CCDC186--PHKA2	7.7
CCDC186--MAP4K1	7.7
CCDC174--ARL2-SNX15	7.7
CCDC162P--MUC4	7.7
CCDC15--CUL5	7.7
CCDC14--ZNF148	7.7
CCDC14--DISC1	7.7
CCDC138--CHD7	7.7
CCDC122--MALAT1	7.7
CCDC112--RC3H2	7.7
CCAR2--IFNGR2	7.7
CCAR1--TASOR2	7.7
CCAR1--DMPK	7.7
CBLL1--CASC3	7.7
CBFB--PLEC	7.7
CAST--TCF12	7.7
CAST--PRELID3B	7.7
CASD1--LAMA4	7.7
CARMIL1--SLC17A4	7.7
CARMIL1--PEG10	7.7
CARM1--STK38	7.7
CAPRIN1--PRKD1	7.7
CAPRIN1--ABCC3	7.7
CAPN9--NOTCH2	7.7
CAPN5--FASN	7.7
CANX--TSN	7.7
CAND1--ZNF107	7.7
CAND1--MALAT1	7.7
CAND1--KIF4A	7.7
CALR--STRC	7.7
CALR--PRRC2C	7.7
CALR--PLXNB1	7.7
CALR--PEPD	7.7
CALR--MALAT1	7.7
CALR--CUL4B	7.7
CALM1--ARSG	7.7
CALD1--MGAT4B	7.7
CALCOCO2--VPS28	7.7
CALCA--UTRN	7.7
CALCA--MALAT1	7.7
CALCA--ATRX	7.7
CADPS2--MALAT1	7.7
CAD--GDI1	7.7
CACUL1--METTL9	7.7
CACNA2D1--WDR74	7.7
CACNA2D1--FTL	7.7
CACNA1H--RNF169	7.7
CACNA1D--RPPH1	7.7
C9ORF78--CLTC	7.7
C8ORF33--RREB1	7.7
C6ORF62--RPL30	7.7
C6ORF62--IFI27L2	7.7
C5ORF63--RNF34	7.7
C5--L3MBTL3	7.7
C2CD4B--CEP85	7.7
C2CD3--XIST	7.7
C2CD3--STAT1	7.7
C21ORF58--RACGAP1	7.7
C1RL--REST	7.7
C1QA--STIP1	7.7
C1ORF43--DNAJC2	7.7
C19MC--IGK@	7.7
C18ORF25--CDC5L	7.7
C17ORF80--STOM	7.7
C17ORF80--COL4A2	7.7
C16ORF72--AFG3L2	7.7
C15ORF40--GAPDH	7.7
C12ORF45--MALAT1	7.7
BYSL--MALAT1	7.7
BX322639.1--ZNF99	7.7
BUB3--PPP1R21	7.7
BTF3--SENP7	7.7
BTF3--AL359762.3	7.7
BTBD3--UBC	7.7
BTBD10--COL1A2	7.7
BSN--MALAT1	7.7
BRWD1--SNX9	7.7
BRWD1--OGFRL1	7.7
BRWD1--FAM204A	7.7
BRPF3--KDM2A	7.7
BROX--ADGRD1	7.7
BRI3BP--MALAT1	7.7
BRD8--ILVBL	7.7
BRD7--TSEN54	7.7

BRD7--SCN4B	7.7
BRD2--RRP1B	7.7
BRD2--RNF10	7.7
BRD2--AHNAK	7.7
BRAF--EFS	7.7
BPTF--XIST	7.7
BPTF--SPP1	7.7
BPTF--MALAT1	7.7
BPTF--IGH@	7.7
BOP1--FOXH1	7.7
BOD1L1--RABIF	7.7
BOD1L1--GJA1	7.7
BNIP3L--GRB2	7.7
BMS1--LRTOMT	7.7
BMPR1A--DSP	7.7
BMP7--AL139300.1	7.7
BLOC1S5-TXNDC5--TUG1	7.7
BIRC6--ZNF706	7.7
BIRC6--RBM39	7.7
BIRC6--CAPN15	7.7
BIRC3--BTAF1	7.7
BICRAL--PLCB1	7.7
BICD1--AHNAK	7.7
BDP1--ZFYVE16	7.7
BDP1--TSPAN13	7.7
BCR--DYNC1H1	7.7
BCR--AL078602.1	7.7
BCLAF1--RPS7	7.7
BCL2L2--COL1A2	7.7
BCL2--KDM6B	7.7
BCL11A--ZNF839	7.7
BCKDHB--HMG20B	7.7
BCKDHB--GLS	7.7
BCAR3--EIF3B	7.7
BBX--SLX4	7.7
BBS2--MALAT1	7.7
BAZ2B--ZZEF1	7.7
BAZ2B--MALAT1	7.7
BAZ1B--MALAT1	7.7
BANK1--COL3A1	7.7
BANF1--BRAP	7.7
BAGE2--CU104787.1	7.7
BAG6--TLK1	7.7
BACH2--SERPING1	7.7

BABAM2--ZNF384	7.7
B4GALT5--PARN	7.7
B4GALT5--MALAT1	7.7
B4GALT4--HECTD1	7.7
B4GALT3--SAFB	7.7
B4GALT1--C21ORF58	7.7
B3GNTL1--HEATR5B	7.7
B2M--MGAT4B	7.7
B2M--INTS8	7.7
B2M--AREL1	7.7
AZGP1--TUBB4B	7.7
AVL9--ABCC10	7.7
AURKA--ADCY3	7.7
AUP1--SFN	7.7
ATXN2L--RAB5C	7.7
ATXN2L--CSDE1	7.7
ATXN2--SSR1	7.7
ATXN10--NFE2L1	7.7
ATXN1--ADCY6	7.7
ATRX--SMG1	7.7
ATRX--NPAT	7.7
ATRX--MYL6	7.7
ATRX--MAGT1	7.7
ATRNL1--TNRC6B	7.7
ATRNL1--MALAT1	7.7
ATRNL1--IPO9	7.7
ATRN--HERC2	7.7
ATR--MALAT1	7.7
ATP9A--TTC37	7.7
ATP9A--KCNQ1OT1	7.7
ATP9A--CSTF1	7.7
ATP6V1H--RP1L1	7.7
ATP6V1H--PPIG	7.7
ATP6V1G2-DDX39B--MALAT1	7.7
ATP6V1E1--IPO9	7.7
ATP6V1A--NDUFAF5	7.7
ATP6V0E2--MFSD4B	7.7
ATP6V0A1--RPS6	7.7
ATP6V0A1--MDM4	7.7
ATP5PO--LARS	7.7
ATP5ME--FTH1	7.7
ATP5ME--CKAP2L	7.7
ATP5IF1--CHMP4B	7.7
ATP5F1E--KPNB1	7.7

ATP5F1C--MALAT1	7.7
ATP5F1B--ROCK1	7.7
ATP2C1--AFDN	7.7
ATP2B2--FNBP4	7.7
ATP2B1--MALAT1	7.7
ATP2B1--AC117386.2	7.7
ATP2A2--MALAT1	7.7
ATP2A2--LRPPRC	7.7
ATP2A2--DA750114	7.7
ATP1B1--DYNC1H1	7.7
ATP1B1--CAPNS1	7.7
ATP1A1--YAF2	7.7
ATP1A1--NOTCH2	7.7
ATP1A1--KIDINS220	7.7
ATP13A3--TAX1BP1	7.7
ATP13A3--MALAT1	7.7
ATP13A3--HSP90AB1	7.7
ATP11C--HSPG2	7.7
ATN1--CKS1B	7.7
ATM--EIF3J	7.7
ATM--ACOT9	7.7
ATM--AC120057.2	7.7
ATIC--MALAT1	7.7
ATG16L1--MICU2	7.7
ATF4--ZNF664	7.7
ATF2--PTK2	7.7
ATAD5--MALAT1	7.7
ATAD5--FN1	7.7
ATAD2B--CUL4B	7.7
ATAD2--LDHA	7.7
ATAD2--CLPTM1	7.7
ASXL2--FBLN7	7.7
ASRGL1--PPP2R2C	7.7
ASRGL1--ARL13B	7.7
ASPM--RAB10	7.7
ASPM--MALAT1	7.7
ASPM--FASN	7.7
ASH2L--PTGFRN	7.7
ASH2L--PRKD3	7.7
ASH1L--MALAT1	7.7
ASCL1--AK9	7.7
ASCC3--MALAT1	7.7
ARPC5--SPP1	7.7
ARPC3--ZC3HAV1	7.7
ARPC2--CCAR1	7.7

ARNT--SYNE1	7.7
ARNT--C2CD2L	7.7
ARMCX6--N4BP2L2	7.7
ARMC8--PAFAH1B2	7.7
ARL8B--ITPR1	7.7
ARL6IP5--DTX3L	7.7
ARL1--MALAT1	7.7
ARIH2--TBX21	7.7
ARIH2--SYPL1	7.7
ARIH2--STT3A	7.7
ARID5B--PHF6	7.7
ARID4B--SLK	7.7
ARID4B--CECR2	7.7
ARID2--SETX	7.7
ARID2--SCAF11	7.7
ARID2--SBF2	7.7
ARID1B--APOB	7.7
ARID1A--KRBOX4	7.7
ARHGEF40--MALAT1	7.7
ARHGEF2--TMEM266	7.7
ARHGEF2--MALAT1	7.7
ARHGEF17--HECTD4	7.7
ARHGEF12--RNF103	7.7
ARHGEF12--ITIH3	7.7
ARHGEF11--FAT1	7.7
ARHGEF10L--UBR4	7.7
ARHGEF10L--FN1	7.7
ARHGDIA--SPG7	7.7
ARHGAP5--MALAT1	7.7
ARHGAP35--NRF1	7.7
ARHGAP31--TP53BP1	7.7
ARHGAP31--MALAT1	7.7
ARHGAP29--CDK6	7.7
ARHGAP27--HECTD4	7.7
ARHGAP26--MUC4	7.7
ARHGAP26--CCNA2	7.7
ARHGAP21--VIM	7.7
ARHGAP11A--PLEC	7.7
ARGLU1--POLR2A	7.7
ARFGEF3--MALAT1	7.7
ARFGEF3--FAM102A	7.7
ARFGEF2--CLTC	7.7
ARF3--CALR	7.7
AREL1--XRN2	7.7
AQP3--SUN1	7.7
APRT--MFSD10	7.7
APPL1--ECI1	7.7
APOOL--MALAT1	7.7
APOM--SUPT20H	7.7
APOM--SMARCC2	7.7
APOL2--KMT2C	7.7
APOE--MACF1	7.7
APOBEC3C--NUP133	7.7
APOB--RPPH1	7.7
APOB--OBSCN	7.7
APOB--MALAT1	7.7
APOB--LRP10	7.7
APOA2--SETD2	7.7
APLP2--SP100	7.7
APLP2--CBX5	7.7
APLP1--MALAT1	7.7
API5--NEAT1	7.7
API5--MALAT1	7.7
APCDD1--VAPA	7.7
APCDD1--DLG5	7.7
APCDD1--ASH1L	7.7
APC--RALGAPA1	7.7
APBB2--DNAJC13	7.7
AP3S1--LVRN	7.7
AP004607.3--CDON	7.7
AP001273.2--TSSK4	7.7
AP001273.2--SERINC2	7.7
AP001267.5--MALAT1	7.7
AP000781.2--CLIP1	7.7
AP000646.1--P4HA1	7.7
AP000350.2--KLHL5	7.7
ANXA5--CALM2	7.7
ANXA5--AL445222.1	7.7
ANXA3--NUP107	7.7
ANXA2--ACTG1	7.7
ANP32B--TRA@	7.7
ANP32B--MALAT1	7.7
ANP32A--MTHFD1	7.7
ANP32A--AC093525.7	7.7
ANKS1B--PPT1	7.7
ANKRD50--ZBED5	7.7
ANKRD50--B2M	7.7
ANKRD46--WDR74	7.7
ANKRD36--C11ORF24	7.7
ANKRD28--PTPN13	7.7
ANKRD26--SPATA13	7.7
ANKRD26--RMRP	7.7
ANKRD17--ARHGAP10	7.7
ANKRD12--DNAJC5	7.7
ANKRD11--DCUN1D1	7.7
ANKRD11--CEP192	7.7
ANKRD10--DKC1	7.7
ANKMY1--PRPF8	7.7
ANKIB1--PAM16	7.7
ANKHD1-EIF4EBP3--SYNE2	7.7
ANKHD1-EIF4EBP3--PCNX1	7.7
ANKHD1-EIF4EBP3--FADS1	7.7
ANKHD1--TRA@	7.7
ANKFY1--SEPTIN9	7.7
ANKAR--PMS1	7.7
ANK2--COL3A1	7.7
ANK2--CALD1	7.7
ANK1--BAG5	7.7
ANAPC5--NCEH1	7.7
ANAPC16--AC018521.1	7.7
ANAPC15--OSCP1	7.7
AMZ2--NRIP1	7.7
AMY2B--UBB	7.7
AMBP--ENOPH1	7.7
ALPK3--ZBTB44	7.7
ALMS1--ENOSF1	7.7
ALK--MALAT1	7.7
ALG5--ILF3	7.7
ALG2--MALAT1	7.7
ALDOA--ZBTB20	7.7
ALDH3A2--AMD1	7.7
ALAS1--ZNF638	7.7
AL845552.1--CPED1	7.7
AL671762.1--KTN1	7.7
AL596202.1--ATP2A2	7.7
AL590004.3--RNF123	7.7
AL512637.1--PRIM1	7.7
AL445487.1--KPNA2	7.7
AL445305.1--AP3S1	7.7
AL390728.4--OAS3	7.7
AL365475.1--PUM3	7.7
AL365181.3--CTNNB1	7.7
AL358334.3--PAX5	7.7
AL357075.5--MALAT1	7.7

AL355987.3--PPIL2	7.7
AL355377.1--SMC5	7.7
AL355297.4--FTL	7.7
AL355075.4--ACTB	7.7
AL354809.1--RP1L1	7.7
AL160286.1--MALAT1	7.7
AL160237.3--PCLO	7.7
AL157392.5--AC026464.4	7.7
AL139300.1--VPS37B	7.7
AL137782.1--VIRMA	7.7
AL133500.1--ITGB1	7.7
AL133353.2--ZNF568	7.7
AL121900.2--GJB2	7.7
AL109827.1--ICAM1	7.7
AL109811.3--RAPGEF2	7.7
AL109811.3--DRAP1	7.7
AL049776.1--TSIX	7.7
AL049697.1--COL3A1	7.7
AL035078.4--NDUFV3	7.7
AL031777.3--UPF1	7.7
AL031681.2--STIL	7.7
AL024498.2--TMEM14DP	7.7
AL022311.1--NEAT1	7.7
AL022238.4--DYRK4	7.7
AL021408.1--CCDC50	7.7
AL021155.5--PHIP	7.7
AL021155.5--MAN2B1	7.7
AL021155.5--MALAT1	7.7
AL021155.5--AL513165.2	7.7
AL020996.2--MKI67	7.7
AKT1--DICER1	7.7
AKR1C3--TSHZ1	7.7
AKR1C3--CEP192	7.7
AKR1C2--MALAT1	7.7
AKR1B10--PTGES3	7.7
AKR1A1--SUGP2	7.7
AKNA--SCARNA7	7.7
AKAP9--ZNF692	7.7
AKAP9--PARG	7.7
AKAP9--KPNB1	7.7
AKAP9--GTF2I	7.7
AKAP9--GPX2	7.7
AKAP9--EIF5B	7.7
AKAP9--CNOT4	7.7
AKAP9--CD74	7.7
AKAP8L--TMTC4	7.7
AKAP6--SRRM2	7.7
AKAP17A--DEPTOR	7.7
AKAP13--HSP90B1	7.7
AKAP11--MALAT1	7.7
AHSA2P--NUP98	7.7
AHSA1--ONECUT2	7.7
AHR--MALAT1	7.7
AHNAK--WDR74	7.7
AHNAK--UCKL1	7.7
AHNAK--TAB3	7.7
AHNAK--STON1	7.7
AHNAK--PBX2	7.7
AHNAK--MMAB	7.7
AHNAK--ASH1L	7.7
AHNAK--ARF3	7.7
AHCYL2--AL162417.1	7.7
AGTRAP--GLIPR2	7.7
AGTPBP1--MALAT1	7.7
AGRN--EFHD2	7.7
AGR2--CCNT2	7.7
AGPS--BCL2	7.7
AGPS--AOC1	7.7
AGO4--ANP32E	7.7
AGO2--SMG1	7.7
AGMO--CXCL5	7.7
AGGF1--TMEM176A	7.7
AGFG1--SLC4A1AP	7.7
AGAP1--RPS6KA5	7.7
AGA--NUCKS1	7.7
AFG3L2--TPR	7.7
AFG3L1P--POLR3A	7.7
AFF4--ACTB	7.7
AFDN--MALAT1	7.7
AEBP1--MALAT1	7.7
AEBP1--CASK	7.7
AEBP1--ARL6IP5	7.7
ADSL--SPTAN1	7.7
ADNP2--ZC3H7B	7.7
ADNP--MALAT1	7.7
ADNP--CACNA2D1	7.7
ADM--TMEM67	7.7
ADK--SLC16A9	7.7
ADK--JAK2	7.7
ADK--DOCK1	7.7
ADH5--NR1D1	7.7
ADGRV1--PFKL	7.7
ADGRV1--MALAT1	7.7
ADGRV1--GAB2	7.7
ADGRV1--B2M	7.7
ADGRL2--MALAT1	7.7
ADD2--ZZEF1	7.7
ADD1--CEMIP2	7.7
ADCY9--MMP25-AS1	7.7
ADARB1--SPATC1L	7.7
ADAR--MALAT1	7.7
ADAR--EPHB3	7.7
ADAR--ARIH2	7.7
ADAMTS9--TRIM72	7.7
ADAM9--MALAT1	7.7
ADAM17--SPG11	7.7
ADAM12--ENO1	7.7
AD000090.1--XIST	7.7
AD000090.1--UBC	7.7
AD000090.1--SRRM2	7.7
AD000090.1--SLTM	7.7
AD000090.1--SEC62	7.7
AD000090.1--NCL	7.7
AD000090.1--MUC4	7.7
AD000090.1--MED13	7.7
AD000090.1--HMCN1	7.7
AD000090.1--H19	7.7
AD000090.1--FTL	7.7
AD000090.1--FN1	7.7
AD000090.1--FLNA	7.7
AD000090.1--EEF1A1	7.7
AD000090.1--DYNC1H1	7.7
AD000090.1--DIP2B	7.7
AD000090.1--CSDE1	7.7
AD000090.1--COL1A2	7.7
AD000090.1--COL1A1	7.7
AD000090.1--AHNAK	7.7
ACVR1B--KMT2E	7.7
ACVR1--SCN9A	7.7
ACTN4--GGNBP2	7.7
ACTN4--DTX3	7.7
ACTG1--UNC13B	7.7
ACTG1--PSAP	7.7
ACTG1--PRKDC	7.7

ACTG1--NEAT1	7.7
ACTG1--HERC2	7.7
ACTG1--CHD7	7.7
ACTB--ZDHHC18	7.7
ACTB--ZBTB16	7.7
ACTB--WDR74	7.7
ACTB--RDX	7.7
ACTB--MLLT6	7.7
ACTB--KAT6A	7.7
ACTB--HACL1	7.7
ACTB--ERICH6B	7.7
ACTB--CRTC1	7.7
ACTB--AHR	7.7
ACTA2--USP34	7.7
ACTA2--DGCR2	7.7
ACSM3--TM9SF2	7.7
ACOX2--ANXA11	7.7
ACOT9--SLC9B2	7.7
ACOT8--ACTA2	7.7
ACAT2--IGFBP4	7.7
ACAT1--MALAT1	7.7
ACAT1--AFDN	7.7
ACAP2--RNF213	7.7
ACADVL--TPM2	7.7
ACADS--TAPBP	7.7
ACAD9--MALAT1	7.7
ACAD8--MALAT1	7.7
ACACB--MALAT1	7.7
ACACA--VPS13A	7.7
ACACA--RTN4	7.7
ACAA2--MRPS25	7.7
ACAA2--LRRC45	7.7
AC253572.2--MALAT1	7.7
AC245047.6--AL606490.1	7.7
AC245033.1--MACF1	7.7
AC244197.3--PKD1	7.7
AC239859.5--IGK@	7.7
AC138915.2--IGK@	7.7
AC138409.2--LBR	7.7
AC132217.2--VPS13A	7.7
AC128688.1--INAVA	7.7
AC124312.3--RBM39	7.7
AC124312.3--FTH1	7.7
AC118549.1--PRPF8	7.7

AC110275.1--BCL6	7.7
AC104758.3--GOLGA6L9	7.7
AC104619.3--MALAT1	7.7
AC104581.2--NEB	7.7
AC104046.1--FABP5	7.7
AC104041.1--CCDC162P	7.7
AC100839.2--FRY	7.7
AC098650.1--PPIA	7.7
AC098582.1--ANLN	7.7
AC098483.1--SYNE1	7.7
AC093512.2--MALAT1	7.7
AC092642.1--TXNL4A	7.7
AC092279.1--BAZ2B	7.7
AC091951.1--GOLGA6L2	7.7
AC091551.1--DPYSL2	7.7
AC090360.1--MLLT10	7.7
AC073912.3--NPIPB5	7.7
AC073610.2--ACTG1	7.7
AC073585.2--UBC	7.7
AC067968.1--NFAT5	7.7
AC048338.2--BDP1	7.7
AC036108.2--RPL18	7.7
AC034193.1--GYS1	7.7
AC027644.4--MRPS33	7.7
AC026979.4--NEFM	7.7
AC026362.1--PIGG	7.7
AC024451.3--UBE2N	7.7
AC023934.1--ZNF91	7.7
AC023509.1--RSBN1	7.7
AC022679.1--POLR2J3	7.7
AC022400.4--TIMM23B	7.7
AC022150.4--ZNF816	7.7
AC020661.4--MALAT1	7.7
AC018362.3--SSR2	7.7
AC017015.1--SET	7.7
AC015813.2--MALAT1	7.7
AC015813.1--TPI1	7.7
AC013410.1--HSP90AB1	7.7
AC012254.2--HNRNPA2B1	7.7
AC010197.2--AP002495.1	7.7
AC009951.4--FN1	7.7
AC009220.3--FCF1	7.7

AC009133.1--AC138028.4	7.7
AC009093.10--BANP	7.7
AC009086.2--SMG1	7.7
AC008581.2--IFITM2	7.7
AC007938.3--MALAT1	7.7
AC007608.4--MALAT1	7.7
AC007608.4--KDM5A	7.7
AC007192.1--NOP14	7.7
AC007192.1--COL1A2	7.7
AC006427.2--TAPT1-AS1	7.7
AC006064.6--PPP3R1	7.7
AC006064.6--MALAT1	7.7
AC006064.6--CAMSAP1	7.7
AC006001.3--UBB	7.7
AC005537.1--ATP8B1	7.7
AC005336.2--POLRMT	7.7
AC005154.2--GOLGA8R	7.7
AC005077.3--GTF2IRD1	7.7
AC004951.1--TIA1	7.7
AC004951.1--LINC00243	7.7
AC004951.1--DOCK5	7.7
AC004148.1--DUSP22	7.7
AC000120.1--MUC4	7.7
ABR--YWHAE	7.7
ABI1--NEK3	7.7
ABHD2--UBC	7.7
ABHD12--APOB	7.7
ABCD1--IGK@	7.7
ABCC6--BLOC1S2	7.7
ABCC4--ATP2A2	7.7
ABCC2--TMX2-CTNND1	7.7
ABCC2--SREBF2	7.7
ABCC2--MALAT1	7.7
ABCB6--HACL1	7.7
ABCB1--TRIM25	7.7
ABCA3--MALAT1	7.7
ABCA1--NUDT6	7.7
ABCA1--CNDP2	7.7
AASDH--ARPP19	7.7
AARS--GSN	7.7
AARS--AP000346.2	7.7
